# Every loaf of bread is political – Reflections on collective care responses to Covid-19 in Cape Town

**DOI:** 10.1016/j.socscimed.2024.116881

**Published:** 2024-04-18

**Authors:** Manya van Ryneveld, Helen Schneider, Leanne Brady, Eleanor Whyle

**Affiliations:** a School of Public Health, University of the Western Cape, South Africa; b Health Systems Division, School of Public Health, University of Cape Town, South Africa; c Emergency Medical Services, Western Cape Provincial Department of Health and Wellness, South Africa

## Abstract

Feminist perspectives on care have demonstrated how capitalism undervalues care work. The Covid-19 pandemic highlighted this further, as systems of production and social reproduction became destabilized globally. In many countries, the formal pandemic response fell short of attending to the daily, fundamental care needs of people living through the crisis, especially those compromised by the socio-economic effects of the pandemic. These needs were often attended to at the community level. This article explores a community-led network of care, known as CANs, that emerged in response to the pandemic in Cape Town. It makes three overarching observations. The first is that community-led responses were characterised by a push towards the collectivisation of care work. The second is that this enabled emergent strategies and relational practices of care, centring notions of solidarity, inter-dependence and horizontal exchange of resources and knowledge. Finally, we observed that, although the devaluation of care work limited the recognition and material support extended to CANs, opportunities to re-politicise care work as resistance work emerged. These represent a prefigurative moment in which alternative logics and strategies can transform the vision of our health and care systems, and the notion of community participation in and ownership of those systems.

## 1 Introduction

The Covid-19 pandemic unveiled many contradictions and compromises in care work. In addition to revealing the vulnerability of health care workers – especially those situated on the lower rungs of the health system hierarchy – the pandemic highlighted the extent to which self-organised networks of people located outside of the health system provide essential forms of non-medical care (Llop-Gironés et al., 2021; Loewenson et al., 2020). Prominent examples of this during the pandemic included establishing community kitchens and systems for checking on those in quarantine, sourcing and sewing masks for distribution, setting up public sanitising stations, and combating misinformation in accessible ways, to name just a few.

This care work tended to sit outside of state-led pandemic responses – which generally took a hospital-centred and top-down approach to ensuring compliance with population-wide lockdown restrictions. Across the globe, community-led forms of care and mutual aid have overlapped, supported, and intersected with state-led pandemic responses, yet largely failed to receive the recognition or material support they deserved (O’Dwyer and Silva Souza, 2022). In many settings the tacit assumption was that this kind of care work fell outside the scope of the state’s responsibility – or even capacity – to provide. Where this led to life-threatening gaps in the pandemic response, it was networks of volunteers, community organisations, non-profit and faith-based groups that stepped in.

In Cape Town, where this research is situated, the pandemic response was characterised by extensive local mobilisation through neighbourhood-based networks of care. This research focuses on Cape Town’s “Community Action Networks” (CANs) which played a pivotal role in propping up the demands, mitigating the repercussions and filling the gaps of the state’s pandemic response. The research contributes to a broader literature which calls for an analysis of Covid-19 and the pandemic response through the lens of a feminist politics of care. Understanding the pandemic through a feminist care lens demands a critique of the structural and material conditions that lead to the devaluing and marginalisation of care and care workers (conditions that lay the ground for both the acute and chronic forms of crisis we face today). However, this lens also lends itself to a “politics of the possible” (Vale, 2020) – hinting at alternative futures in which care systems are publicly oriented, democratically organised and embedded in a relational ethic of mutual interdependence and social reproductive solidarity (Razavi et al., 2022; Tronto, 1993; Vale, 2020).

### 1.1 Care through a feminist lens

This research grounds its observations in feminist theories of care that speak to the place of care in an unequal world. This body of scholarship has contributed to our understanding of how care is shaped by the intersecting forces of gender, race, class, and economy. Debates in feminist economics have demonstrated how the capitalist economic order is structured into two spheres – the sphere of the market-based, “productive” economy and the sphere of the non-market-based social reproductive economy, also known as the unpaid care economy (Bhattacharya, 2017; Razavi, 2007). As a system, capitalism is built upon a fundamental contradiction in that the possibility of market-based wealth accumulation within the so-called “productive economy” is dependent upon the unpaid and undervalued labour of the care economy (Folbre, 2014; Fraser, 2016). This contradiction is normalised and obscured by a seemingly “natural” divide between the public, economically productive sphere, and the private, social reproductive sphere, upheld in part by a gendered moral and social order that denotes care work as “women’s work” or a “labour of love” (Tronto, 1993). The obscuring of care and social reproductive labour is further entrenched by the subaltern identities of those who do care work, mapping onto other patriarchal power relations and forces of racial subjugation inherent to capitalist society (Bhattacharya, 2017).

In the current era of neoliberal, financialised capitalism, capitalism’s contradiction manifests as the interlocking crises of care, climate, and economy, producing an underlying, prolonged state of “general crisis” into which moments of major disruption such as the Covid-19 pandemic arrive. In the context of this generalised crisis, systems of social protection such as health systems have been debilitated by structural adjustment and austerity policies. The displacement of responsibility for social reproduction from the collective to the individual, and from the public to the private, or to a thinly stretched, volunteer-based “third sector” of NGOs and community-based organisations, has been normalised (Cardona, 2021; Paremoer, 2023; Patel, 2009). The effects of these crises are mitigated by the care economy, where social reproductive work is extracted out of care workers in a similar way to how natural resources are extracted out of the earth. This concept of “care extractivism” highlights the “intensified commodification and exploitation of the resource labour in social reproduction for the purpose of managing crises situations without burdening the state or the health industry with additional costs and responsibilities” (Wichterich, 2020, p. 122). The state, subsumed by the managerial agenda of “cost-efficiency” and other dictates of neoliberalism, abdicates responsibility for care onto care workers such as community health workers and other community-based volunteers, whose unpaid or underpaid labour subsidises the work of the state (Abramovitz and Zelnick, 2010; Hunter, 2012; Paremoer, 2023; Vale, 2020).

This paper begins from the premise that community-based care responses to the pandemic – including those that are considered “informal”, self-organised and hyper-local in their scope – should be understood and recognised as an essential part of the public health response. In the South African context – where care has been systematically devalued and relegated to the realm of the private, domestic, or third-sector space (Fakier and Cock, 2009; Lund and Budlender, 2008; Patel, 2009) – it is imperative that we conceptualise the pandemic response beyond the imposition of lockdowns, social distancing, quarantine, or the availability of vaccines, hospital beds and oxygen. A broader conceptualisation must consider the equally life sustaining work of those attending to their own and their communities’ survival in times of multi-layered precarity. At the heart of this are communities, households and neighbourhoods and the ongoing, daily labour of – usually – working-class women of colour; mothers, sisters, aunties, and grandmothers, whose bodies and capacity for labour become the “shock absorbers” of the social reproductive crisis produced by capitalism (Fakier and Cock, 2009). Theirs’ is the everyday, life-sustaining work of cooking, feeding, growing, building, nursing, cleaning, fetching, organising – activities that under capitalism, are largely relegated to the economically “unproductive” care economy.

Yet it is this care work makes the state’s pandemic response possible, as it continues to sustain life in the wake of both a life-threatening pandemic and, at times, a life-threatening state response (in the form of lockdown regulations that cut people off from their livelihoods without offering any support in its place, or lockdown regulations enforced through extreme police violence). Much of this care work is characterised by its “everyday-ness” (Jupp, 2022). It is often organised informally, in an ad hoc manner based on the relational and interpersonal practices that characterise daily, ongoing forms of care. This means that, even in moments in which care work is made more visible, it often remains illegible to formal institutions and bureaucracies such as the state and is therefore excluded from or denied recognition and material support.

This paper describes an example of a community-led pandemic response in which care was momentarily displaced from its usual confines within the invisible, feminised, and private, social reproductive sphere – benefitting from collectivisation and receiving recognition in new ways. Relationships were built and strategies emerged out in the open, offering a chance for new social reproductive solidarities between care actors to emerge (Razavi et al., 2022). This was a moment that revealed ways of doing care that emphasise our inter-dependence and the mutuality of our care needs. However, as the pandemic moment dissipated it also became evident how normative frameworks towards care that maintain the status quo of a debilitated and marginalised care economy, remain intractably embedded.

### 1.2 Research methodology

The research methodology informing this paper is based on collaborative feminist research praxis that prioritises the communal production and transmission of diverse knowledges, and attempts to challenge power relations between the researcher and the researched (Benson and Nagar, 2006; Nagar and Ali, 2003). All of the authors were members of their neighbourhood CANs throughout the pandemic period. Three of the authors (MVR, EW, LB) were also involved in various network-wide activities, workshops, and inter-CAN events such as cycle rides or community kitchen visits and exchanges. Aside from providing opportunities to build relationships, these activities enabled us (as both researchers, and CAN members) to document, learn from, and reflect on the experiences of CANs in responding to the pandemic, through participatory “sense-making” methodologies that emerged.

A key data source for this paper is text extracted from the publication *Dala Kitchen* – *More Than a Cookbook*, where many of the reflections, interviews, stories, and narrative artefacts from the network were collated and published. The pieces in the cookbook were gathered and written by various people within the CTT network and represent a wide range of voices from across the city. The process of producing the cookbook reflects the collaborative praxis that shaped this research and is described in further detail below. For the purposes of triangulating data for this paper, the textual data extracted from *Dala Kitchen* was supplemented by additional data gathered by the first author (MVR) including in-depth unstructured interviews with two CAN members conducted in 2022, as well as MVR’s notes, reflections and participant observations collected over the course of her involvement in the network between 2020 and 2021. Informed consent was sought from the two interview participants and ethics approval to conduct this research was received from the University of the Western Cape.

An inductive coding approach was applied to the data, beginning with an *a priori* coding framework drawing from the literature, as well as an insider understanding of the network that resulted from the first author’s positionality as an embedded researcher and CAN member. A preliminary set of codes was used to guide a line-by-line reading of *Dala Kitchen*, which resulted in the extraction of text that was then categorised and grouped according to the coding framework, while also expanding and deepening it further. A simultaneous process of “justification” was conducted in which the authors reflected on the selection and identification of each code, providing a brief explanation of its relevance to the coding framework. This was done in an attempt to make explicit the intentional “bias” inherent to the coding and analysis process. Once coding of the text of *Dala Kitchen* had been completed, the supplementary data underwent the same process of extraction, coding, and justification. The entire coding process was captured within a table from which a thematic analysis followed.

It is important to note that the process of producing *Dala Kitchen* was an act of collective, participatory “sense-making” in which members of this emergent city-wide network were able to share, record, digest and represent our own stories. The findings reported in this paper should therefore be contextualised as the by-product of this process, which was not oriented towards academic outcomes but rather towards the broader political project of providing (and reimagining the provision of) care during the pandemic. *Dala Kitchen* was produced by a group of 15 embedded researcher/activists and CAN members (including the authors), known as the Connecting CANs Fellowship. This group held some of the creative and administrative space needed to connect different nodes across the network, with funding from the School of Public Health, University of the Western Cape, which financed the production and printing of the cookbook, as well as a living wage for some of the fellows. This group oversaw the cookbook project, inviting wider collaboration from across the network and providing the logistical support for the collection of stories, interviews, and materials for the book. *Dala Kitchen* was constructed by hand through a DIY book making process. The project was initiated in October 2020 and ran for approximately 9 months, culminating in the publication of the cookbook in July 2021. We invited anyone from the CTT network to join in a series of workshops held at one of the community kitchens in Gugulethu, where the book was pieced together, page by page, using collage methods inspired by the “Pumfleteers Collective”. The Pumfleteers Collective was founded by architect, Ilze Wolff and artist, Kemang Wa Lehulere in 2016 and “exists to publish interventions into the social imaginary”. In the run up to making Dala Kitchen Ilze Wolff shared various zine-making methodologies and collage techniques based off their “Pumflet” series of zines, which heavily informed the design of the cookbook, as well as the hand-made and collective ethos of its production. Participants were asked to bring artefacts, such as photos, recipes, notes, or anything they felt represented their contributions. These were scanned and included in the book. Interviews with CAN members were conducted by members of the Connecting CANs Fellowship via phone call, WhatsApp messages, and in-person conversations. Some stories were submitted in written form by CANs themselves. The pages went through an editorial process led by MVR, LB, and EW, who edited some of the stories and interviews for language and clarity and decided upon the sequencing of the contents. The book was shared through a series of book launches organised by CANs across the city and distributed for free across the network and beyond, with copies placed in kitchens, bookshelves, and public libraries.

*Dala Kitchen* is an archival and textural consideration of the many hours of labour, collective action and acts of solidarity that constituted the network. The book contains recipes as they are used in the community kitchens – often cooked in hundred litre pots – as well as photographs, drawings, maps, artworks, scans of pressed herbs like *dhania* (coriander), leaves and flowers from community gardens. *Dala Kitchen* represents a wide range of voices, experiences, lessons, and memories drawn from across the network, emphasising the political nature of this work, while also grounding it in the everyday contextual realities of those doing the work. In this sense, *Dala Kitchen* brings together what Houston refers to as “biographical, political, philosophical and place-based meanings” to forge and sustain “alternate knowledges” that are essential to imagining and bringing forth different futures (Barca, 2020; Houston, 2013).

*Dala Kitchen* also joins a legacy of activist cookbooks such as the *Philadelphia People’s Cookbook*, and the *Rural Women’s Assembly Cook-book*, as well as other historical examples such as the Black Panther’s Breakfast Programme, all of which demonstrate that the politicisation of our collective food practices is an important strategy within broader struggles for liberation. As a discursive object, the cookbook is a contested site of gendered expression. Traditionally, cookbooks have often been used to uphold patriarchal gender divides and the naturalisation of “women’s work”. However, in reclaiming the cookbook as a site and tool of political expression, activist cookbooks such as *Dala Kitchen* make the assertion that spaces of social reproduction are also political spaces.

### 1.3 Contextualising the Covid-19 response and the Community Action Networks in Cape Town

The Covid-19 pandemic unfolded onto the backdrop of Cape Town’s deep socio-economic inequality. Lasting legacies of spatial apartheid have kept the city’s neighbourhoods segregated along racial and class lines, imposing high transport costs on workers and low-income residents who mostly live in the peripheries of the city. The National Minimum Wage, brought into law in 2020, is R20,76 an hour, which is insufficient to cover even basic living expenses. According to March 2021 data, there were 1 113 198 people receiving social grants in the Western Cape province, out of a total of 11 450 005 grant beneficiaries across the entire country (Orlandi, 2021). Furthermore, both locally and nationally, the health and social welfare system is marked by systematic encroachment of private sector interests, and generally poor public sector governance and mismanagement. Public social services are heavily overburdened, and the private sector is largely inaccessible to low-income South Africans.

Low-income households in the country face extreme levels of nutrition poverty and household food insecurity, with female-headed households being the most vulnerable (Jacobs, 2010). Food insecurity in South Africa should be understood as an issue of “chronic emergency” – a framing used by Bhan et al. (2020) to highlight how prolonged crisis and precarity is a core feature of the everyday conditions of life facing low-income South African households, even prior to Covid-19. After core expenses such as transport and electricity are subtracted from the monthly wage, the money remaining to secure all other expenses generally falls far short of the cost of a household food basket for a basic nutritious diet (Abrahams et al., 2024). These scenarios were heavily exacerbated during the COVID-19 pandemic (Wills et al., 2020; Wonci, 2020). This is important context for understanding the scale and extent of the problem of household food insecurity to which community-based food aid initiatives responded during the pandemic.

The declaration of a national state of disaster, which enabled the pandemic response to be implemented, took on strong authoritarian tones, employing a top-down approach that was largely decided upon by national government and implemented through hierarchical and heavily medicalised scientific committees and task teams. The pandemic response was initially premised on a national lockdown to give the health system time to prepare for the inevitable surge in hospitalisations, involving the complete closure of all but the most essential services. The closure of non-essential services and the resultant economic shutdown meant that millions of people lost their livelihoods overnight. The introduction of two new grants – the R350/month (USD $18) Covid-19 Social Relief of Distress (SRD) grant, and the Covid-19 Temporary Employer/Employee Relief Scheme (TERS) – assisted in alleviating some of the financial and food insecurity brought on by the lockdown. However, levels of hunger spiked massively from the imposition of the lockdown in March 2020, and one year later more than 1 in 3 adults were reporting that their households would run out of money to buy food by the end of the month – far above the hunger levels documented in pre-pandemic data (Visagie and Turok, 2021). When disaggregated according to type of neighbourhood, poor urban communities were the most vulnerable to high levels of both unemployment and food insecurity as a result of the lockdown (Ibid). Thus, the fact that the national coronavirus response had relatively minimal consideration of emergency food provision as compared to the readying of a small number of cost-intensive field hospitals or the deployment of military and police personnel to enforce lockdown regulations, was a significant gap in the state’s pandemic response.

Shortly before the lockdown was announced in March 2020, an initiative known as Cape Town Together (CTT) was catalysed by a group of community organisers, activists, artists, public health practitioners, and researchers (including the authors) with the purpose of supporting residents who organised themselves into neighbourhood-based response groups called Community Action Networks (CANs) (van Ryneveld et al., 2020). The premise of CTT was to build, support and connect multiple, localised, networks of care led by people living in the same neighbourhoods who could respond flexibly and in a self-organising manner to the many challenges brought on by the pandemic and ensuing lockdown. The CANs received no formal support or endorsement from the state. There was very little network-wide funding for the CANs – aside from funds for mobile data which was essential to enable connection – every CAN oversaw their own fundraising, as well as any accountability systems for managing funds. As a neighbourhood-based network, the CANs introduced a scale of organising that was hyper-local, encompassed wide contextual diversity and required the building of relationships as a core function of the work. This was described in a reflection written in the cookbook by members of the Langa and Kenilworth CANs:

“The CANs’ focus on hyper-local organising, either within one’s own neighbourhood or in partnership with a few others, meant that, on the one hand, the scale of movement was drastically reduced. In Kenilworth CAN we met in the local church, walking distance from all our homes. In Langa CAN we used bicycles to deliver food and medicine … on the other hand, the spread of the network across the city represented a massive and catalytic movement, not just of the concept of the CAN as an appropriate Covid-19 response, but of the underlying organising principles and vision for a city based on solidarity, radical generosity and togetherness” (Langa CAN et al., 2020, p. 25)

At its peak, towards the end of 2020, the network numbered over 170 CANs spread across neighbourhoods of varying class, race, and socio-geographical positions. While there is no numerical data on this, anecdotal evidence suggests that women made up most of the CAN membership. The CANs operated autonomously, responding to the challenges experienced in each neighbourhood. No two CANs encountered the same scenarios; however, a massive increase in hunger and food insecurity was ubiquitous across the network, mainly because of job losses due to the lockdown, as well as the closure of schools – a key source of daily meals for children through the Schools Nutrition Program. Over the course of 2020 and 2021, almost every CAN in the city was involved in providing some form of food support – whether through community kitchens cooking and serving meals directly to people, or through the distribution of food vouchers and grocery parcels. As food support became an important frontier of the community-based response to Covid-19, community kitchens and food gardens became some of the core spaces within the CANs where various collective strategies of “everyday” care could be put into practice.

## 2 Findings

Through the coding and analysis process, we made three overarching observations. The first is that, at grassroots and community levels, the pandemic response was often characterised by a push towards the collectivisation of care work. As a result, care work became a visible part of the response, partly through a blurring of the divide between the public and private spheres. The second is that this enabled emergent strategies and relational practices of care, centring notions of solidarity, inter-dependence and horizontal exchange of resources and knowledge. Finally, we observed that, notwithstanding the increased visibility of care work through initiatives such as the CANs, there remained limited recognition and material support extended to this type of work by powerful actors, such as the state. As the pandemic moment dissipated, a return to the “old normal” quickly set in. However, one should not disregard the value of the pandemic as a prefigurative moment, in which alternative logics and strategies of care can transform the notion of community participation in, and ownership of, our health and care systems, thus re-politicising care work as resistance work. In the following sections each of these observations will be discussed further using findings from the data.

### 2.1 A politics of doing – places and scales of care

“I wanted to do something with my hands. At least I can make a hundred loaves of bread. Just to feed some children, people who don’t have anything to eat. It’s a real honour because there is so many people, they are struggling that go sleep without food but at least they are getting a loaf of bread, even if I must get up at 5 o’clock in the morning or even three o’clock or even two o’clock. I do that for them.” (Thomas & Ocean View CAN, 2020, p. 64)

The idea of “*doing something with my hands”* – small-scale, localised forms of action such as checking in on elderly neighbours, sewing masks, distributing food by bicycle, cooking meals, or sharing fact-checked information about the pandemic to combat stigma and misinformation – sat at the heart of what the CANs’ referred to as a “politics of doing”. There was a widely shared and urgent need for people to feel they could respond to the pandemic in whatever capacity was available to them, at the scale that made sense. The neighbourhood CANs thus served as an immediate and accessible site of impact and meaningful action in the face of unprecedented crisis. While the scale of the action was local, the impact was often significant, representing mammoth efforts on the part of small groups of people:

“People would send as little as R40 to buy a few loaves of bread, uhm, we had pensioners reaching out to us “I would like to help but I can’t go to the shops, can I send you money for bread?” And that is essentially how it all snowballed, you know. We started out, the first time we bought bread we bought forty loaves of bread. And we handed that out … Again, it so happened that we went from 40 breads to 80, to 100 and eventually we stopped at 400 loaves of bread every single day. Now, you can do the maths. For close to six months, every day of the week, to buy 400 loaves of bread, how much money we collected, having no bank account, not being registered, but making sure that people could see what we were doing …” (interview with Nadia Mayman of the Joint Peace Forum-Bontehuewel CAN, 2022)

For many people, taking action was not a choice but a matter of survival – relying on other forms of aid or support (from the state, for example) was simply not an option – especially for food and other essential needs. As Pamela Silwana, a member of Gugulethu CAN stated in a newspaper article published about the CANs in a local online newspaper – *“people would be going home to empty cupboards and would literally die of hunger before they got sick from Covid-19 … Nobody can stay home to #stopthespread if they have no food”* (Whyle et al., 2020). However, what makes the call to *“do something with one’s hands”* not only an act of survival, but also a form of political action is rooted in its collectivisation and self-organisation that shifted the work into a blurred space between the private and public spheres.

The privatisation of care, and its association with familial or domestic spaces, the inside of people’s homes, or “women’s spaces” (such as the kitchen), is one of the key mechanisms through which care work is obscured in the everyday (Fakier and Cock, 2009; Jupp, 2022; Razavi et al., 2022). With food provision as a core activity of most CANs, there was an urgent need for spaces where CANs could pack food parcels or cook and serve meals (often for hundreds of people at a time), where anyone could stand in line for a meal, or ask for or share information. The “open access” nature of these activities demanded a restructuring of local spatial arrangements, notwithstanding the immense challenges in doing so during a time of restricted movement, as well as adherence to complex principles of infection control and protection from the virus. As CAN members reclaimed and repurposed various spaces – ranging from the inside of their own kitchens, garages and backyards, the streets and pavements outside their houses, their mosques, churches and community halls, and open public spaces like sports fields, abandoned lots, and public squares – they were actively bringing this care work out into the open, thus making it more visible and marking it as both a practice of collective caring *and* a form of political action. The choice (and limitations on choice) of spaces to provide care is an important way in which the CANs brought care work into the blurred space between public and private – making the work visible while also having to negotiate significant contestation in these spaces – for example, from law enforcement officers trying to shut down kitchens for contravening lockdown regulations, or angry neighbours acting from places of fear or prejudice towards community kitchens, or the dismissal of food provision and community kitchens as *“mere feeding schemes”*. Through the process of collectivising these widespread yet local, everyday practices of care, they became a visible part of the Covid-19 response, even if only at the community level - as one CAN member put it, *“we were cooking more than food”*.

The kitchen of Maria Nombulelo Dlokolo’s (also known as Gogo, meaning grandmother in isiXhosa) demonstrates many examples of the emergent strategies for repurposing and responding to the many new and existing care burdens during the pandemic. Before the pandemic, the space was not always thought of as a community kitchen, even if it sometimes provided a similar function – Pamela, Gogo’s daughter, explains:

“Before covid, it wasn’t that busy, my mom’s wasn’t a “kitchen” kitchen, because she had a catering business. So, what she would do is if she had leftovers or enough food, she had days where she would call people and give, also people would come and say Gogo, I’m hungry” so she would give, she did that for many years, she wasn’t like a regular kitchen, but it was a restaurant and people would come. (Interview with Pamela Silwana of the Gugulethu CAN, 2022).

Suhair Solomon, another Connecting CANs fellowship member, writes in the cookbook about Gogo’s kitchen:

“It strikes me as I walk in through the front gate of Gogo’s home how calm it is in the front yard. She’s sitting with one of her volunteer stalwarts Sis’Lindi, and they’re peeling and chopping potatoes and carrots with expert precision. They have just finished the morning breakfast serving from the kitchen and have moved smoothly into preparing the lunch meal, her famous meatballs recipe. They are not rushed, in fact that is something I notice about the entire visit; nothing is rushed, but everything is purposeful. Every moment appears seamless, from who steps up to check pots, sharing instructions on timing of dishes, negotiating the seemingly endless number of passers-by who check in to greet, express gratitude or enquire about various needs of support or aid. It’s a smooth flexible operation, bending to the varied unpredictable needs, with a clear goal in mind: feed people with dignity and always with a side portion of love.” (Gugulethu CAN et al., 2020, p. 38)

Gogo’s kitchen served meals to anyone passing by. It also became a meeting point for multiple people and CANs across the network. Volunteers in the kitchen were trained as Gender-Based Violence first responders to be alert to any signs of domestic violence in people coming to the kitchens. Across many of the kitchens, strategies for overcoming logistical and other challenges emerged. Bicycles were often used to transport meals from the kitchens to the households of people who were self-isolating, or too frail to stand in line themselves. Efforts were taken to ensure that the kitchens were well-managed in terms of Covid-19 safety and infection control principles, with information and ideas about how to achieve this shared amongst kitchens across the network through social media and online co-learning sessions. When kitchens had an oversupply of certain ingredients – which happened fairly often due to the haphazard and precarious nature of sourcing supplies – these were shared using the CAN WhatsApp groups.

These examples demonstrate how, in establishing alternative, collective sources of support through these localised, neighbourhood-level networks, the CANs became a “site of transformative politics” in the practice and provision of care work. (Gibson-Graham, 2003). This echoes the idea of the “everyday” as a site of alternative possibility, while at the same time highlighting the ways in which precarity demands responsiveness and adaptability from people, creating infrastructures and systems of care that are therefore inherently fluid, multi-purpose, informal and, at times, ambiguous (Gibson-Graham, 2003; Jupp, 2022; Razavi et al., 2022).

### 2.2 Reconfiguring flows of care

The CANs did not operate along one unitary logic of care. While acts of doing were at the centre of each CAN’s work, the ways in which this was achieved, and the underlying principles for organising, differed from CAN to CAN. In some neighbourhoods, default charity models between the “haves” and the “have-nots” prevailed, without attempting to unsettle the biases within these. It was also clear that the burden of labour fell disproportionately onto women within the CANs. This emphasises that there is nothing inherently emancipatory about care work – its mere existence does not necessarily engender transformation or a politicisation of the labour (Tronto, 1993).

However, the collectivisation of care work experienced in the CANs impacted the potential way that care could be understood and valued across the network, centring notions of solidarity, inter-dependence and horizontal exchange of resources and knowledge. These were viewed as contesting a capitalist logic of care as “charity”, or as an individualised responsibility, or as only valuable insofar as it responds to the needs of the most privileged in society, all of which tends towards the devaluation of care (Morrow and Parker, 2020; The Care Collective, 2021; Tronto, 1993). In emphasising horizontal flows of care across the network, where tangible and intangible resources were recognised, valued, and exchanged, the directionality of aid that normally flows from materially wealthy to materially poor was disrupted. Elizabeth Drevlow of the Hout Bay CAN reflected on resource sharing across the network:

“Engagement with CTT also enabled us to draw on the experience and expertise of other CANs across the city. We shared the lessons we had learnt and heard from other CANs what was working for them. Knowing other CANs struggled with similar issues to us, that we were in this learning process together, was comforting” (Hout Bay CAN & Drevlow, 2020, p. 109)

Pamela further explained how, in their partnership with the more economically well-off Seaboard CAN, there was an awareness of the various kinds of resources and labour needed to provide care – financial support from wealthier neighbourhoods was just one element:

“I was talking to someone, I don’t know where I was, but someone asked me about the relationship between Gugulethu and Seaboard. So they said, is it charity? And I was like, I wouldn’t take it as charity because if I count the hours that I work and put into helping my community I wouldn’t call it charity. The work that the kitchens are doing and the hours that they are putting even if you came to me and gave me R2000 to help in my kitchen, you know, I wouldn’t take it as charity because I’m putting the work in as well.” (Interview with Pamela Silwana, 2022)

Another example that speaks to the reconfiguration of care flows is how, during instances of other crises – such as shack fires, a common occurrence in townships across South Africa, and violent evictions of shack dwellers from new settlements – the networks of CANs were able to kick into action to respond to these:

“As we were writing this piece, 20 homes were burnt in Gugulethu. Within 12 hours, the affected families were provided with food and accommodation, while furniture and funds to help them start again are being collected.” (Gugulethu-Seaboard CAN et al., 2020, p. 66)

The pandemic presented a moment in which the care work could be understood and valued in a new light. Community-based responses such as the CANs reveal how labour, knowledge, relationships, and material resources intersect to create the possibility to care in any given context. These are the things that bridge the gap between being able to afford to donate 10 kg of ingredients and knowing how to cook a 100-kg pot of food with these ingredients, and serve it to 300 people in a dignified, organised and Covid-19 safe manner.

### 2.3 A re-politicisation of care work

If it is the invisibility of care work that helps to keep its subordination in place, then moments of disruption such as the Covid-19 pandemic certainly helped to bring out the contradictory nature of this into the open (Razavi et al., 2022), and responses such as the CANs made further inroads by highlighting the power and impact of collective action. However, it is clear from the ways in which the CANs struggled to access support, the ways funds started to dry up as soon as a sense of “normality” began to return, with donors ‘dropping like flies’ and kitchens being forced to close, that the devalued place of care, and the perceived expendability of community care workers who fill the gaps, converge as a broad set of limits to this work. Hunger stands out in particular as a “second pandemic” that continues to confront communities across the city and to which many CAN kitchens are still needing to respond – but now without the groundswell of action linked to the pandemic moment. Both Nadia Mayman (of the Bonteheuwel-JPF CAN) and Pamela Silwana (of the Gugulethu CAN) reflected on this during interviews with MVR in 2022:

“Up until today, the need is still so great, our people are not managing … just the other day I wrote an article of an old woman that was arrested for shoplifting, what did she steal? Formula! For her grandchild. Because that is the real lived realities on the ground, so a lot of sponsors during the first 12–14 months of the pandemic, and then we started seeing less and less coming because now restrictions were reduced, people were going back to work and donors probably felt the need isn’t there anymore but you know the need is still there, if not worse, because the effects of covid, it lingers on. People are without work, many people went back to work, but they were required to work one week off one week on, then breadwinners have passed away, pensions are no longer available.” (Interview with Nadia Mayman, 2022)

“If you looked at now and before I would say that the level of food quality has changed, you know, you’re not going to have a stable situation of food coming in. Sometimes someone donates, they will donate samp, they will donate uhm, butternuts, and they will donate split peas or something, but then there’s something that you don’t have you can’t make it nice, you can’t even put it together, flavouring, or garlic or anything, and all of those things you still can’t get protein to make it balanced” (Interview with Pamela Silwana, 2022)

Care-based responses such as the CANs were less likely to be seen as legitimate forms of pandemic response because they often took the form of self-organised, informal networks, following different logics of care, collective action, and sensemaking, in contrast to the formalised pandemic responses promulgated through regulations, based on the evolving medicalised scientific advice, and moving through hierarchical chains of command. Yet, CANs were able to demonstrate that a localised, neighbourhood-based response that could work flexibly and through interpersonal relationships was an appropriate modality for meeting fundamental non-medical care needs during the pandemic. Furthermore, responding to the shared sense of precarity and insecurity produced by the pandemic moment, helped to strengthen social reproductive solidarities and relationships across CANs – and therefore across socioeconomic divides. *“The food we make is for everyone, we’ll feed whoever is in need of food* – *which could be any one of us in these times.”* (Goodwood CAN et al., 2020, *p. 93*).

A result of this shared sense of precarity and the solidarity was the repoliticisation of the caring work performed in and by the network:

“Cape Town Together is a non- partisan, non-religious and vigorously independent network not beholden to any political party, donor, or organisation. While we continually resist the distraction of party politics, the work we do is, of course, deeply political. As one CAN member put it, in the context of extreme hunger and poverty, “every loaf of bread is political”. (Writers CAN et al., 2020, p. 117)

For most CANs, the work they did during the pandemic was not new. It built on existing relationships, structures and, in many instances, a living memory of community-based and collective organising that has persisted since apartheid, particularly in working-class neighbourhoods. The CANs’ demonstrated alternative practices of everyday care that recognise it as a valuable resource, a political act, and a collective responsibility. In doing so, they offer up new modes of resistance to a status quo in which care burdens are inequitably and unjustly distributed. While questions of sustainability or scalability of self-organising, community-led care models are important, focusing solely on these obscures the enormous prefigurative potential of initiatives such as the CANs. The care work found in the CANs does not simply represent an act of survival (although it may also be that) but demonstrates multiple strategies for the collectivisation and prioritisation of care work, and therefore demands that we engage with the contradictions and complexities of how this work is constrained under the status quo. As Emejulu & Bassel point out, such strategies create the possibility of new political subjectivities amongst care workers, enabling a “revalorisation of grassroots activism” that understands care as “a radical politics of everyday possibility” (Emejulu and Bassel, 2018, p. 116).

## 3 Conclusion

The pandemic, and its ensuing strategies of lockdowns, led to serious destabilisation and erosion of already-precarious systems and arrangements supporting and enabling care for the vast majority. For those CANs in urban poor areas, where the pandemic represented additional disruption on top of chronic states of emergency, the work continues unabated – in particular in the realm of household food insecurity. Feminist perspectives on care, in particular in its place-based, embodied, and everyday forms, enables a re-politicisation of this work, centring it as political action in its own right, while remaining cognisant of representations that seek to romanticise, neutralise or co-opt it to obscure underlying power structures (Jupp, 2012, 2022; Wichterich, 2020).

In this paper we observed the immense potential for demonstrating alternative structures and practices of care through networks based on solidarity and horizontality. The multiple, multi-functioning, and responsive local circuits and strategies of care that emerged from the CANs draw on memories, knowledge, and immense expertise in organising. Through engaging with the narratives and descriptions of care work provided during the pandemic, this paper paints a picture of the complexity of providing care through informal, local networks, where women, in particular working-class women, are the most active players, often working against immense odds. It is clear that, in times of crisis (and beyond), initiatives such as the CANs provide a backbone that fills in the gaps in care and social protection provided by the state and other more “formal” players. Yet, as the paper shows, this visibility of care work and the recognition of its political value is tenuously positioned and does not necessarily translate into more equitable treatment of care workers at the community level. There are important questions about the distribution of responsibility for this care work and the need for it to be resourced, legitimised, and valued differently. We should continue to take up these questions in further research informing conceptualisations of equitable models of care. But at the same time, we should not discount and further invisibilise the value of this moment, where care networks such as the CANS sprung up in many parts of the world, played a life-saving role and demonstrated the ‘politics of possibility’.

## Supplementary Material

**Appendix A. Supplementary data**

Supplementary data to this article can be found online at https://doi.org/10.1016/j.socscimed.2024.116881.

Data Kitchen Cookbook

## Data Availability

Confidential data such as interview transcripts will not be shared, however most of the data informing this paper is document-based data that is available in the public domain.
