# Supplementary material for: Every loaf of bread is political – Reflections on collective care responses to Covid-19 in Cape Town
Source: Soc Sci Med. Author manuscript; Available in PMC 2026 Oct 23. (PMC7619622; doi:10.1016/j.socscimed.2024.116881)
Supplement: Supplementary file 1 [file EMS216064-supplement-Data_Kitchen_Cookbook.pdf]

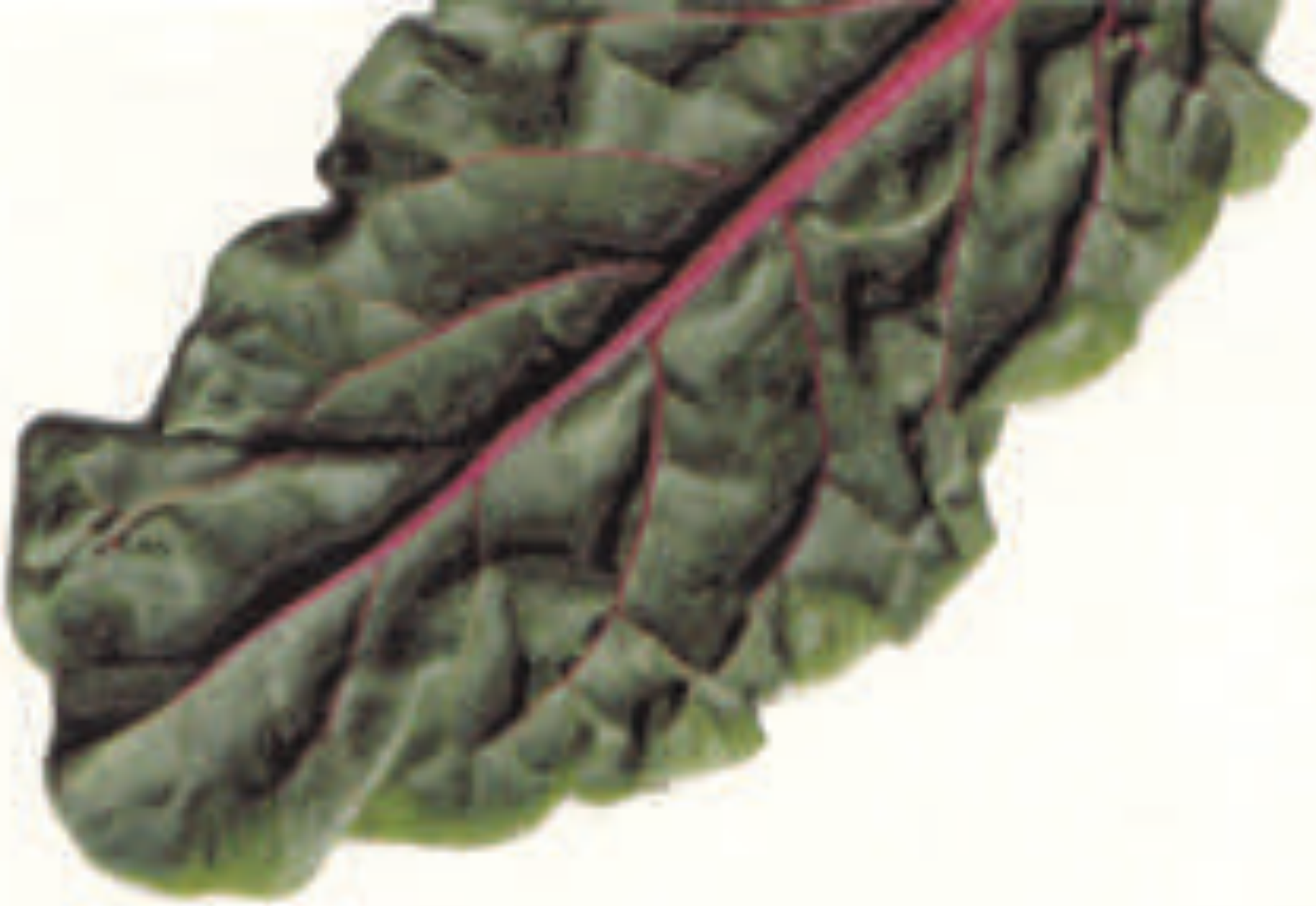

DALA Kitchen

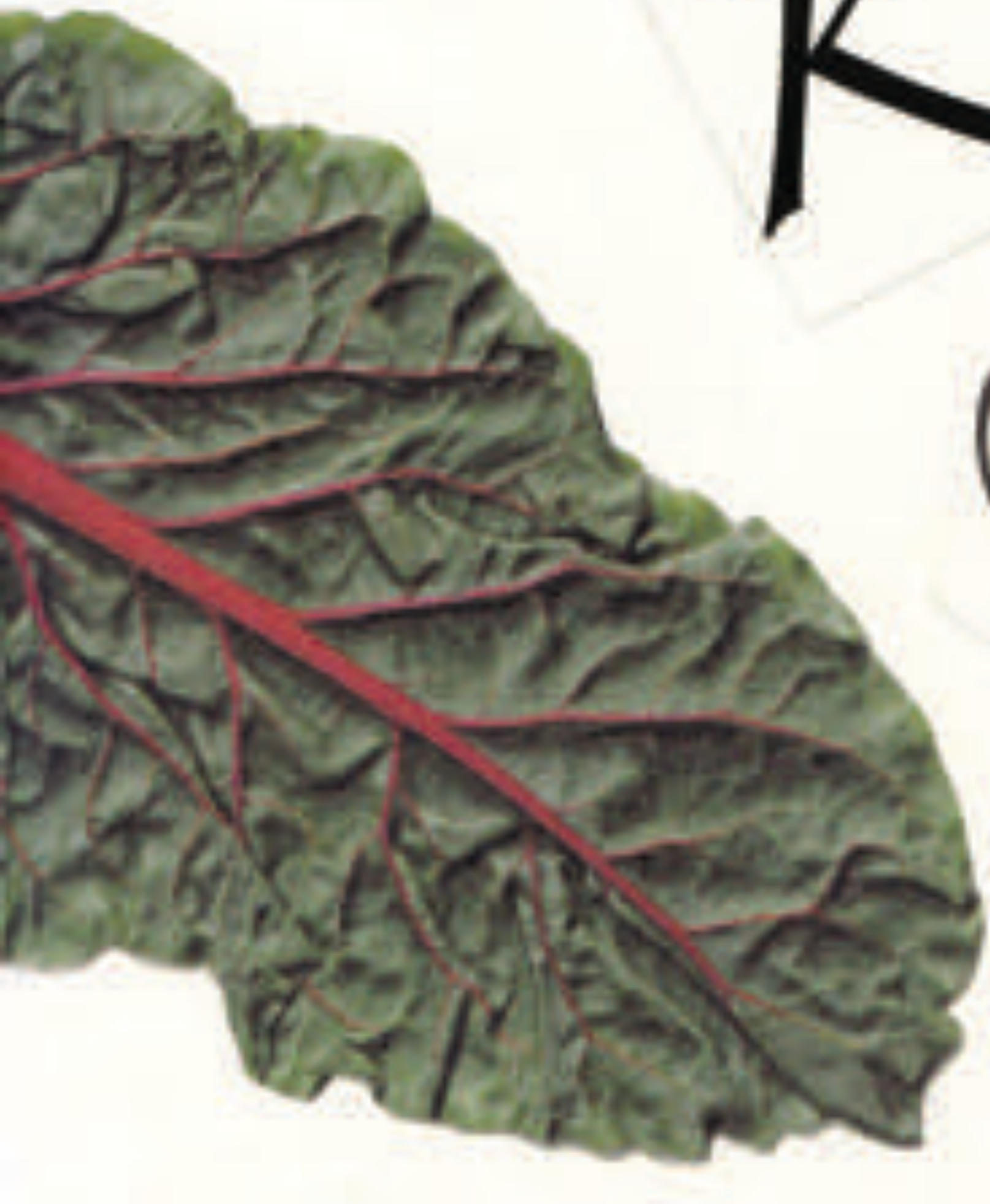

More than A cookbook

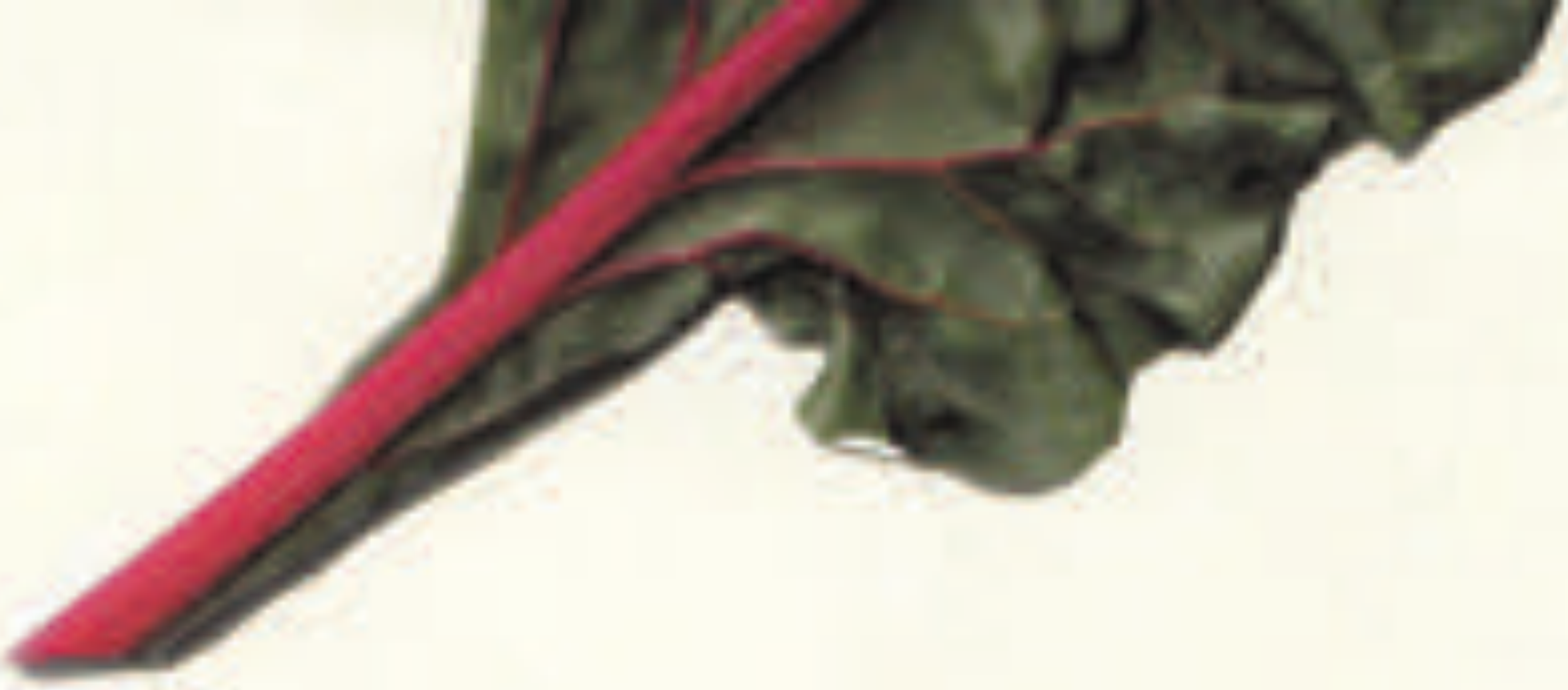

None of us are safe until we're all safe

Historically, pandemics have forced humans to break with the past and imagine their world anew. This one is no different. It is a portal, a gateway between one world and the next. We can choose to walk through it, dragging the carcasses of our prejudice and hatred, our avarice, our data banks and dead ideas, our dead rivers and smoky skies behind us. Or we can walk through lightly, with little luggage, ready to imagine another world and ready to fight for it.

– *Arundhati Roy*

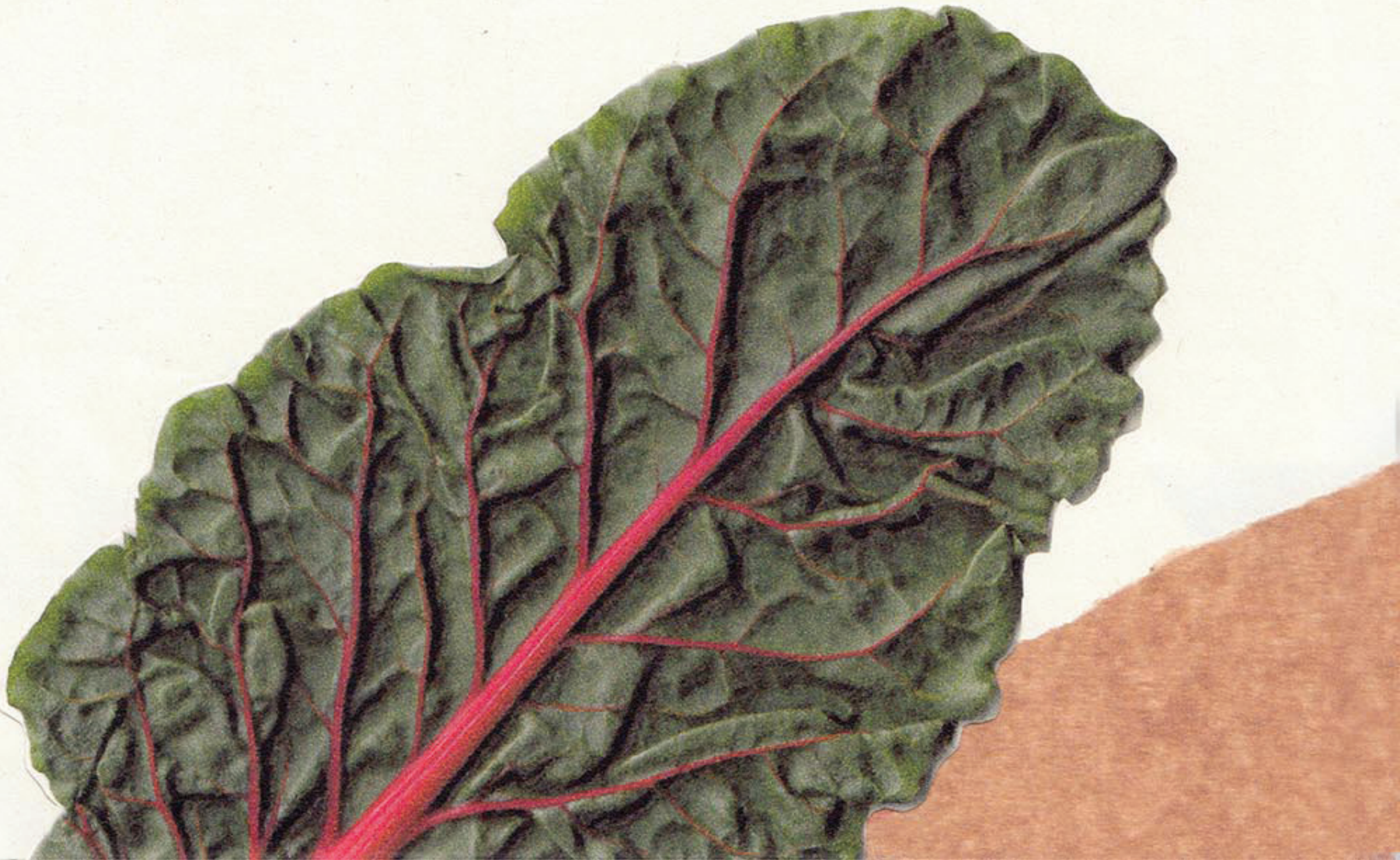

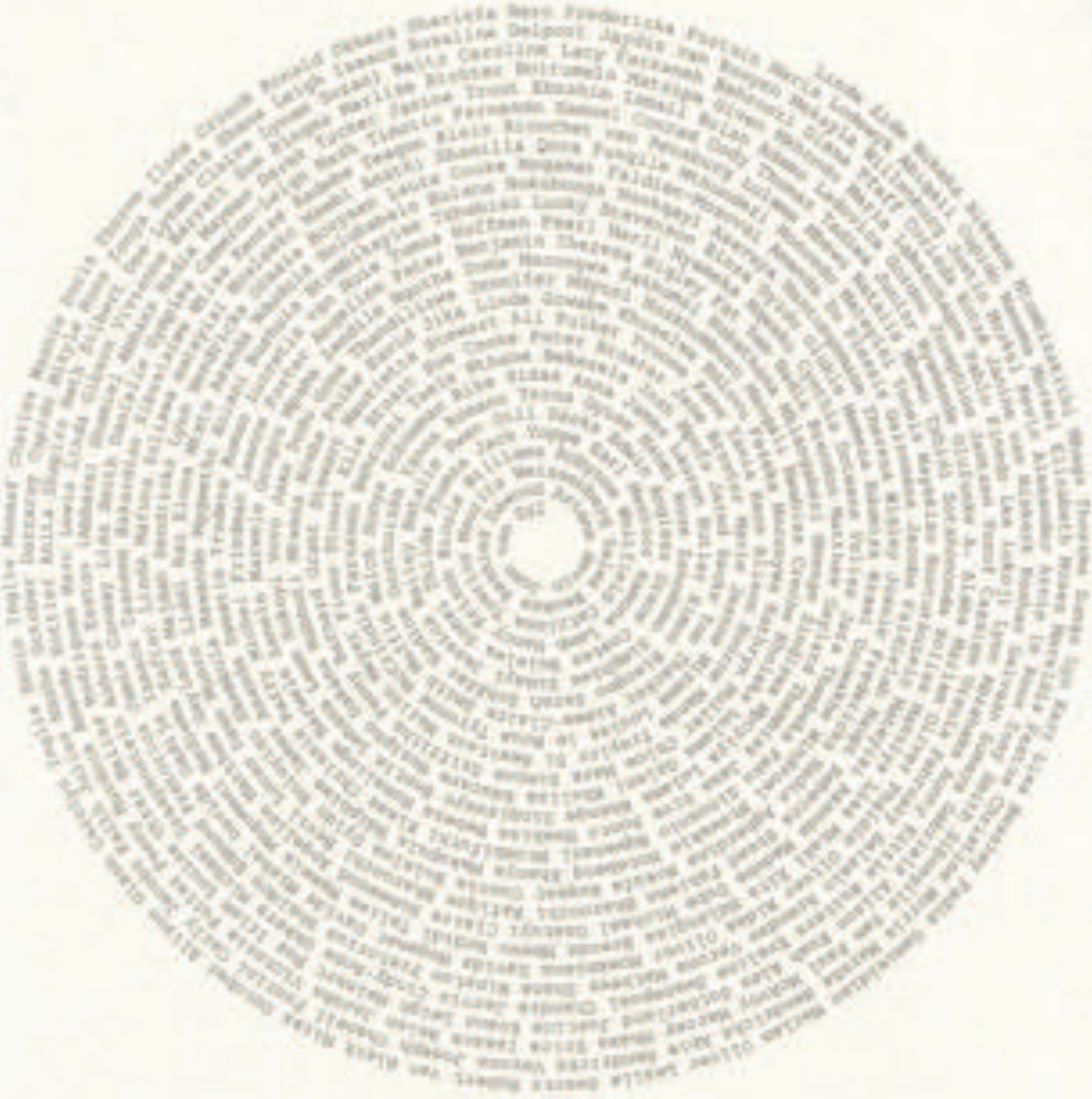

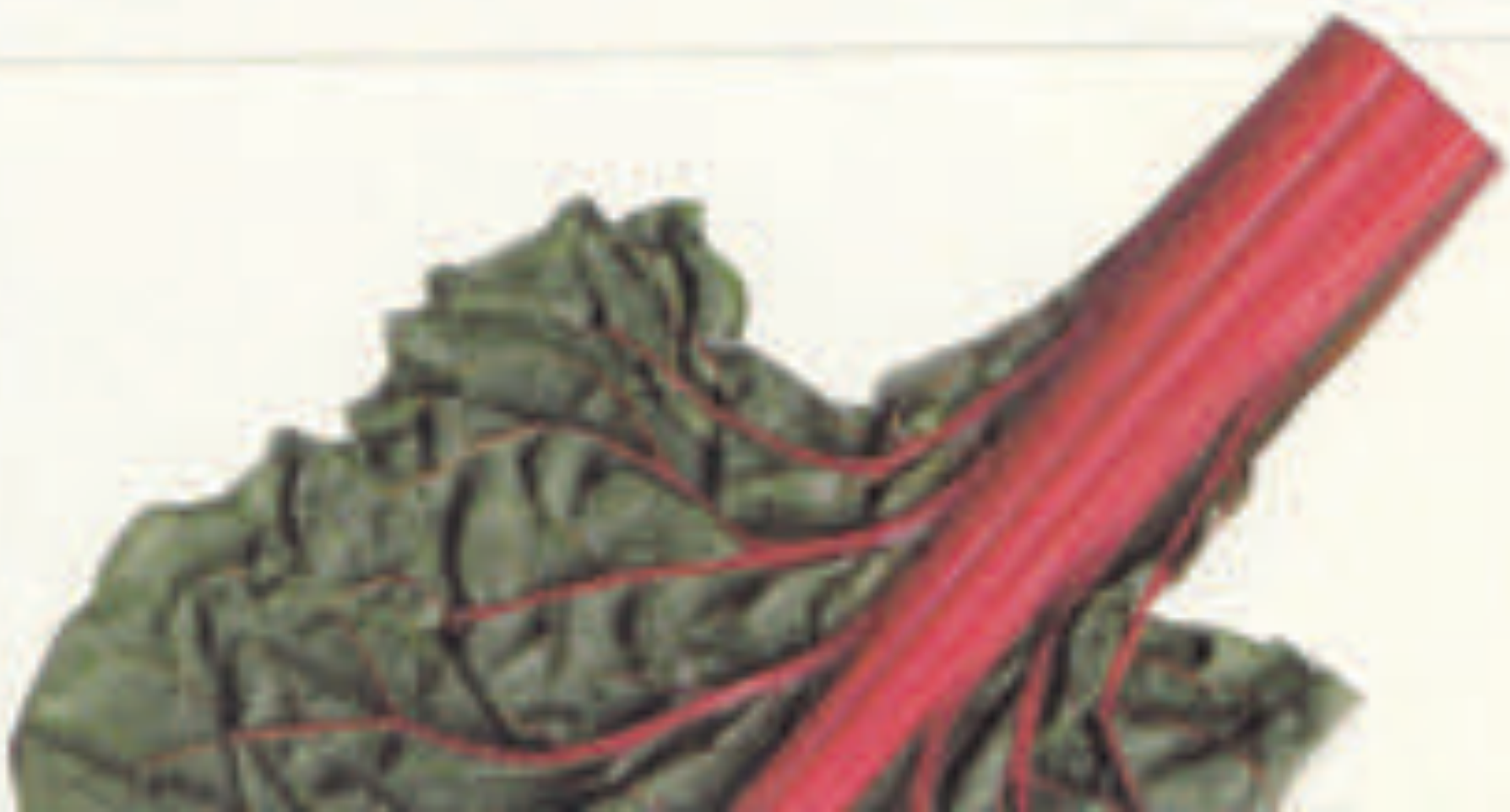

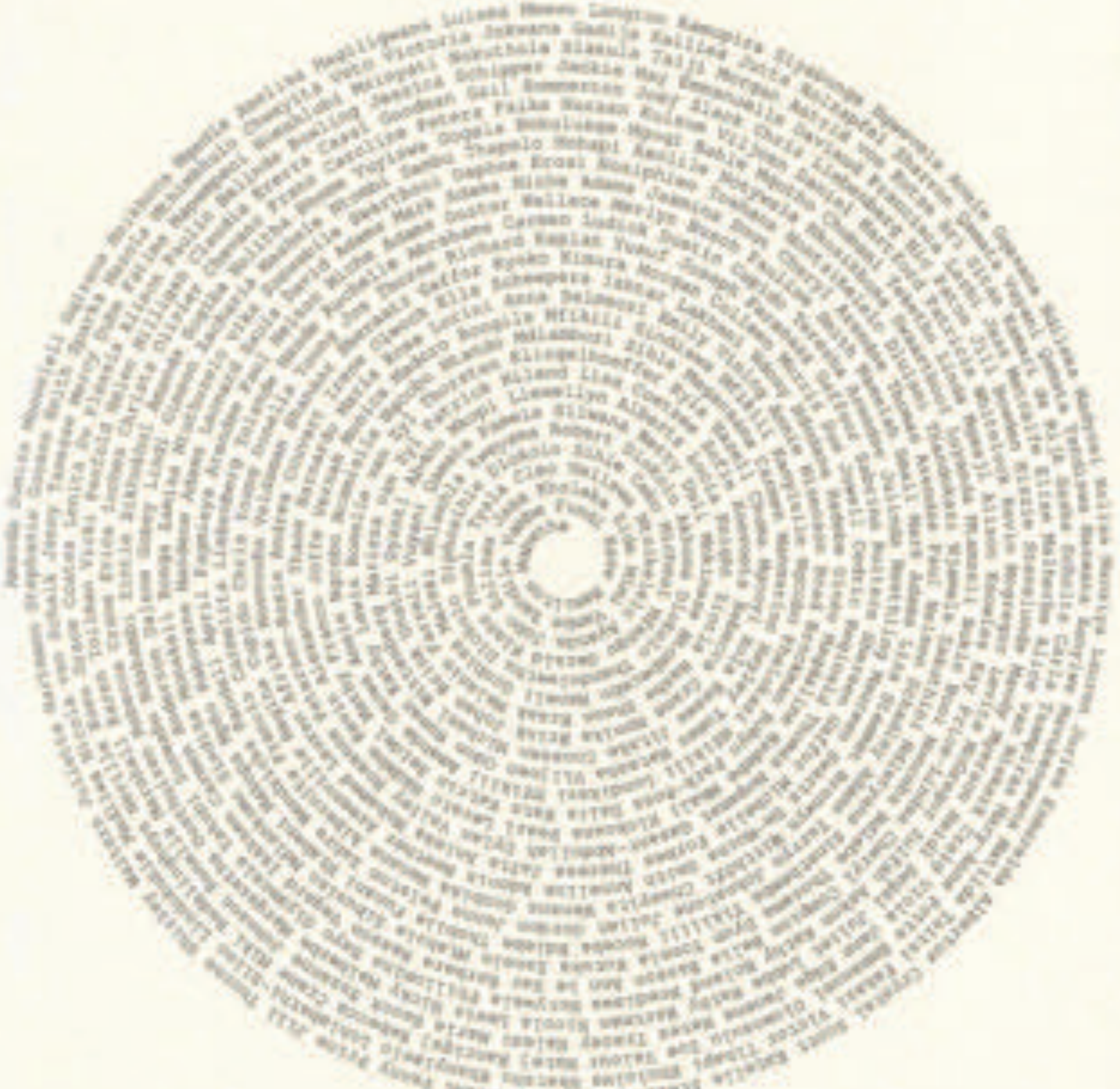

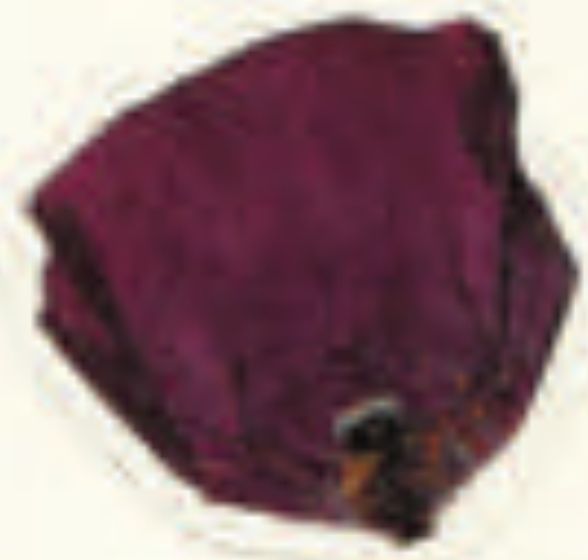

Contents

Acknowledgements 6
About this book 7
Map of the CANs 62
Making sense of magical chaos 125

CAN Kitchen Recipes

Nigerian Chicken Jollof Rice: Ekuphumleni CAN 14
Cheesy Fish Cakes: Ocean View CAN 21
Umngqusho: Langa CAN 24
Vegetable Stew and Meaty Bones: Brakenfell-Kraaifontein CAN 27
Gogo's meatballs: Gugulethu CAN 36
Lamb curry: Salt River CAN 40
Stir Fry: Heideveld CAN 44
Lasagne with Soya Mince and Butternut 46
Chicken Curry: Hanover Park CAN 51
Fish Breyani: Wesbank CAN 57
Turkish Chickpea Stew: Muizenberg CAN 58
Recipe for growing your own herbs: Salt River CAN 60
How to plant beetroot: Clovelly CAN 61
The bean soup that saved us: Kenilworth CAN 68
Borscht: Newlands CAN 74
Ubuhle Besizwe Soup: Site B CAN 78
Chicken akni: Bonteheuwel CAN 84
Spinach Quiche: Wesbank CAN 88
Nadine's Smoortjie: ObsCAN Feed You 90
Milho: Goodwood CAN 91
Soya Mince Frikkadels: Ocean View CAN 87
Tomato and Onion Salad: Salt River CAN 92
Veggie Steam Bread: Site C CAN 102
Homemade Bak Brood: Hanover Park CAN 106
Flapjacks: Ocean View CAN 107
Lemon and Ginger Cordial: Newlands and Philippi CAN 110
Toffee Apples: Hanover Park CAN 114
Whyle's choc chip biscuits: Connecting CAN 119
Heavenly Banana and Oat Muffins: Mini Meltdown 123
Potato Koesister: Ocean View CAN 124

Recipes for Organising

Cooking up a network: Perfect for any crisis 22
How to CAN 48
Recipe for a WhatsApp group 94
A recipe for learning together 98

Stories from the Network

The power of people caring: Health CAN 11
Isolated possibility 16
Home away from home: Ocean View CAN 19
Cycling across divides Langa CAN 25
Remembering Robyn: Mowbray/Rosebank CAN 28
We all bring something to the table: Tafelsig CAN 30
Trusted trouble-makers in government: Connecting CAN 33
On lifelines, legacy and love: Gugulethu CAN 38
Torched car becomes beacon of hope: Mini Meltdown 41
Tracing broken lines: CBD CAN 50
Love in the time of Covid: Clovelly CAN 52
Solidarity not charity: Gugulethu-Seaboard CAN 65
Streets of care: Mowbray/Rosebank CAN 70
A letter from Chev's Kitchen 75
Fighting for identity: Khikhi CAN 76
Planting seeds in the Kipling Street Food Garden: Salt River CAN ... 79
Bonteheuwel community group takes flack from DA councillor: Bonteheuwel CAN ... 81
Lighting candles: Pinelands CAN 86
Bringing Goodwood together: Goodwood CAN 92
Professors of the street: Health CAN 103
Building networks of trust: Hout Bay CAN 108
Girls on bikes: Harare CAN 112
Is Cape Town Together a political threat?: Writers CAN 115
Practising democracy in a pandemic: Newlands CAN 120

Acknowledgements

Dala Kitchen is a celebration of the work of Cape Town Together. During 2020, thousands of people came together to support one another in a range of creative and radically generous ways. This work is the inspiration for this book.

Special thanks to Maria Nombulelo Dlokolo, aka Gogo, from the Gugulethu CAN who hosted a series of Covid-safe book-making sessions at Gogo's Kitchen where we dreamed up Dala Kitchen while eating together. We gratefully acknowledge the contributions of all those who attended the workshops to share ideas and visual inspiration, submitted stories, sent recipes and photos, and picked leaves and flowers from their gardens. We also thank Jared Rossouw whose idea for a CAN cookbook planted the seed that blossomed into Dala Kitchen.

Dala Kitchen is one of the collective outputs from the Connecting CANs Fellowship – a group of CAN members from 12 different CANs and public servants seeking to creatively distil and document the lessons from nine months of Cape Town Together. The Connecting CAN Fellows include Leanne Brady, Shaheem De Vries, Yaseen Johaar, Nadia Mayman, Tyronne McCrindle, Mzikhona Mgedle, Irene Olwoch, Marlise Richter, David Pienaar, Khanya Qonqgo, Ricochet van Rensburg, Manya van Ryneveld, Pamela Silwana, Suhair Solomon and Eleanor Whyle.

The Connecting CANs Fellowship and Dala Kitchen have been enriched by the insight, encouragement and tireless support of Carnita Ernest, Asha George, Lucy Gilson, Uta Lehmann, Tamlin Petersen, and Helen Schneider.

We thank Ilze Wolff for guiding us through this collective creative process, We thank Graeme Arendse for his digital mastery. The beauty of this book is a testament to their thoughtfulness and generosity.

We also thank Dylan Valley for his huge contribution to the collective outputs of the Connecting CANs Fellowship, including the Cape Town Together music video and short film, and Conor Ralphs for the beautiful map of CANs in this book.

Lastly, and importantly, we acknowledge that Cape Town Together and the Connecting CANs node reflect the radical and progressive ideas of the folk who have been active members of this collective in the past. In particular, we thank Marcela Guerrero Casas, Dustin Kramer, Phumeza Mlungwana, Axolile Notywala, Injairu Kulundu-Bolus, Kentse Radebe, Jared Rossouw, Ella Scheepers, Gavin Silber, and Nkosikhona Swartbooi.

About this book

Dala Kitchen is a collection of recipes and stories from CANs and CAN community kitchens across the city of Cape Town. It gives a taste of some of the work that happened in the first 9 months of the pandemic. While these are just a small fraction of the delicious recipes and incredible stories that make up this rapid community-led response to Covid-19, we hope they give a good flavour of what the network is all about. Enjoy with a dash of fresh imagination and a serving of grassroots politics!

On March 5th 2020 Covid-19 arrived in South Africa. Shortly after that, a national state of disaster was declared, and schools were closed. It was a time of great uncertainty, and many people across the country were grappling with the shock of lockdown, lost income and food insecurity, alongside fears of becoming infected with the virus and spreading it to others.

During this time, a group of community organisers, activists, public health practitioners, artists and researchers came together to take action. It was clear that a community-led Covid-19 response that would spread faster than the virus itself was needed, and that the usual approach of working through top-down government structures and NGOs would not be fast enough. Instead, we needed something that built on existing networks, but reconnected them across the usual divides, while also inviting ordinary people to be part of the response in a safe and responsible way.

On March 16th, Cape Town Together was born – a rapid community-led response made up of self-organising neighbourhood-based groups, called CANs, that are connected through the broader Cape Town Together network.

Every neighbourhood is different, and this model recognises that there is no 'one size fits all' approach. The loose network structure means that CANs develop interventions that make sense in their local context, but also offers platforms to share ideas and lessons in real time between CANs across the city.

Of course, many community groups would have responded anyway – without any prompting from a network like Cape Town Together. The logic of grassroots self-organising exists in our DNA as South Africans, especially for those of us who lived (and continue to live) through the oppression of the apartheid system. But by working together as members of the Cape Town Together network, we are able to build relationships and connections that amplify our response and powerfully demonstrate what is possible through collective action. Covid-19 serves as a stark reminder of our inter-dependence and connectedness. And in a city as divided as ours, finding ways to build solidarity across race and class lines is essential.

By joining a CAN, each new member becomes a part of the broader collective, to share ideas and resources with people in different parts of the city, and to learn, collectively, through doing. With no organising centre, our mandate is to find ways to support each other. Many CAN members were involved in community work already – through existing structures like faith-based groups or NGOs. But those affiliations are left at the door when joining a CAN. This allows us to work beyond our organisational mandates, while bringing our skills and energy to the CAN. It also means that anyone can join a CAN, and many people who had never been involved in community organising before jumped in.

Soon, thousands of people were organising in their own neighbourhood CANs – sharing important health information, making masks, or spray-painting safe distancing lines outside their local clinics and spaza shops. When lockdown was announced, and it was clear that people would go hungry without support, hundreds of community kitchens opened up and started finding ways to safely provide food to their neighbours. As the lockdown led to the invisible second pandemic of hunger, and government support failed to materialise at scale, food became a central focus of the network. The CAN kitchens themselves became spaces to put Covid-19 safety measures into practice, share information about the virus and build local knowledge and responses to the hardship and hunger being experienced.

The work has been tough – and often the temptation is to slip into the usual command-and-control way of doing things. But in this network, we are fiercely decentralised and anti-hierarchical. There are no bosses, and every CAN decides for themselves how best to adapt and respond to changes and local conditions. This comes through in the stories included in this book, where the wide range of activities and focus areas of various CANs is evident. There are stories about cooking meals in community kitchens, handing out food parcels, and starting community food gardens. Some CANs focus on supporting people living on the streets, while others look out for the elderly. No matter the specific course of action taken by each CAN, the work is underpinned by the shared dream of establishing city-wide networks of care, rooted in a politics of collectivism and radical generosity.

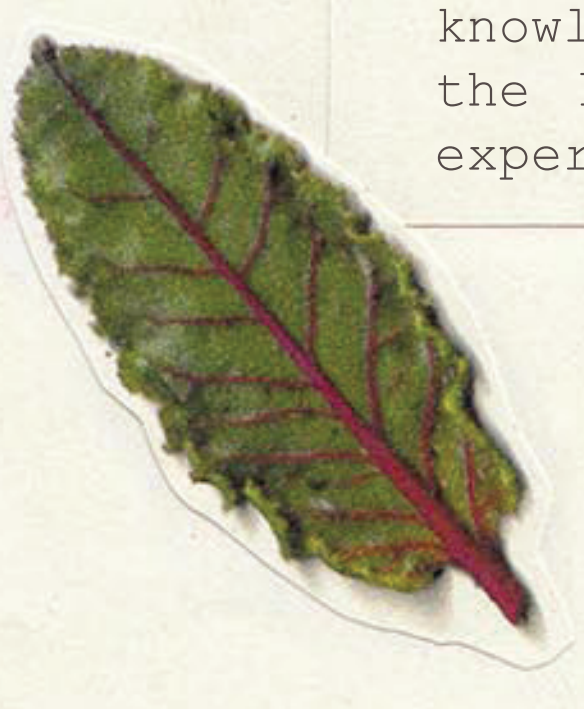

Relationships are key to how we maintain energy and commitment – by creating spaces of love, friendship, energy and laughter. Sowing seeds of belonging is the only way to invest in the longevity of our network. These relationships and friendships, which connect people across the city who might never have crossed paths otherwise, will sustain beyond the lifespan of the network and the pandemic. They represent a well-laid foundation for our collective response to the coming challenges we will face. And through these relationships new forms of leadership have emerged, often drawing on feminist principles that understand the individual as part of a complex whole and foreground a critique of power dynamics and oppression in our usual leadership models – even those that may emerge unconsciously.

This book is a testament to these relationships – and to the immense work done by ordinary, dedicated people caring for each other in a time of great uncertainty and with very little formal support or resources from government. The stories, images and recipes included here paint a picture of both the magic and joy as well as the hardship and heartache that is Cape Town Together.

The reality is that the challenges of inequality, oppression, violence and poverty that pervade our city exacerbate the health and economic consequences of Covid-19 and made the stringent lockdown period almost as deadly as the virus itself. While these are major issues, stemming from our country's colonial and apartheid history and require much bigger changes in how our society is structured, the CANs' 'politics of doing' provided a pathway to action in the present and at the hyper-local level that is deeply personal. It is about neighbours getting to know each other and working together, inspiring one another and generating collective energy to keep going, to start building a better world.

We warmly invite you to partake in this journey with us. The recipes have been left as they were written – intended as they are for use in community kitchens, providing meals to many hundreds of people. In these pages you can read how Gogo makes meatballs for over 300 people at her kitchen in Gugulethu. You will find Ricochet's mom, Rochelle's koesister recipe, which we have faithfully and gratefully enjoyed at almost every in-person meeting held within the network. There are recipes from across the city, as well as CAN stories that illustrate the political and social tensions and contradictions underlying this work. Together these recipes, stories and images weave a sense of the possibility and magic that arises when ordinary people come together to do ordinary things in an extraordinary way.

I realised that if I had kept quiet, something valuable would not have been said

— Pamela Silwana

Gugulethu CAN

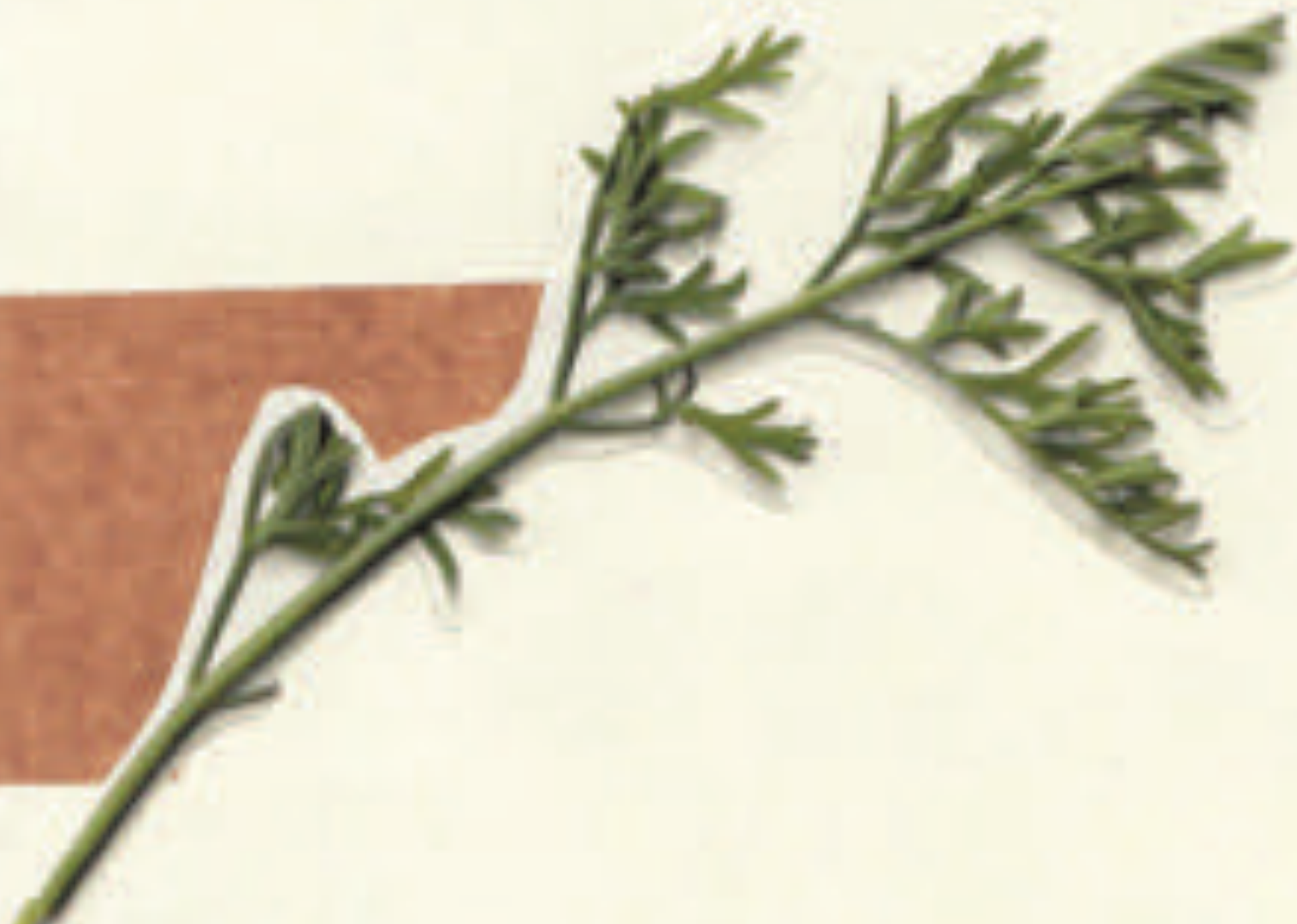

The power of people caring

Health CAN

First published by Daily Maverick as 'The power of people caring for those affected by Covid-19' - 13 April '20

In South Africa, while the national response has not been without mistakes and challenges, including reports of police brutality, unlawful evictions, and questionable relocation plans for the homeless, the national government's response has been largely commended for being appropriate, timeous and decisive.

In a country with complex economic and health challenges, implementing strict lockdown measures early in the epidemic has been viewed as a brave step, grounded in scientific evidence and intended to stop the spread and save lives at all costs.

In addition to the lockdown, a nationwide community-based screening and testing programme is being implemented. This programme is an integral arm of the response and should be rolled out quickly and carefully in order to justify the implementation of the severe lockdown. In the Western Cape the Department of Health has taken proactive steps towards this, along with a robust system for contact tracing.

However, there is a third arm in the response, which may not have received its fair share of attention, resources and, dare we say it, imagination: the key question of what to do after testing.

Public health folk, community organisers and medical doctors alike are all in agreement about one thing — as far as possible, we need to keep Covid-19 cases out of our already overstretched hospitals. Italy's experience tells us this — the stark contrast in the number of infections between the Lombardy and Veneto regions have been linked, at least in part, to the hospital-based and home-based care approaches that the two regions chose respectively. A community-based approach to managing the disease is the best chance we have, especially if it puts community inventiveness and responsiveness front and centre.

It's been estimated that 80% of the people who test positive for Covid-19 will not need high-level care. They will need three square meals a day, social support and protection, and, perhaps most importantly, a safe and appropriate place to self-isolate. A further 15% will need hospitalisation with slightly more advanced clinical care, and 5% will need access to ICU care.

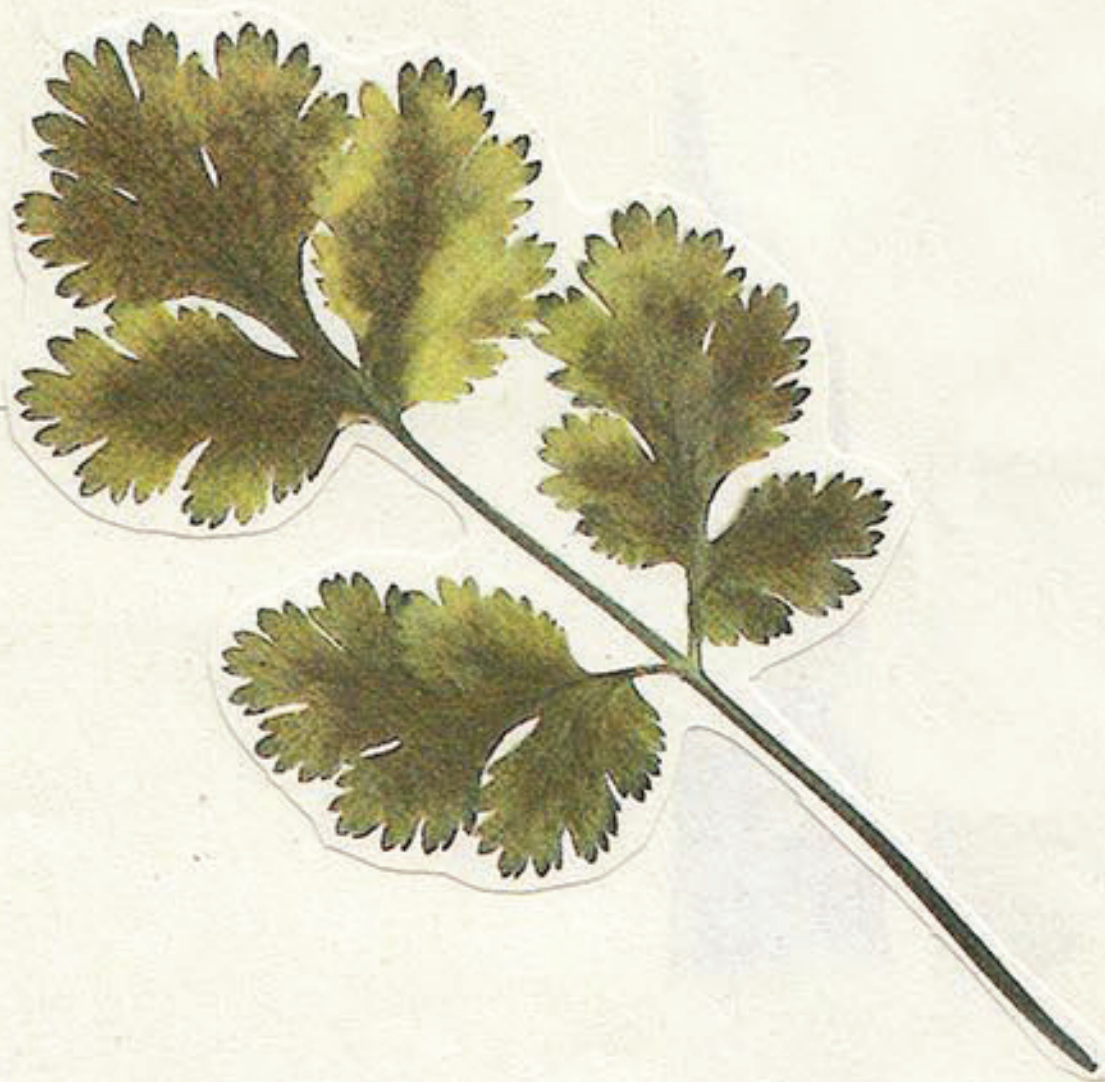

But in South Africa, many of the 80% who could self-isolate without hospital-isation, will find it impossible to do so at home. Highly overcrowded living conditions, a lack of water and poor sanitation and food insecurity are all common features of many South African neighbourhoods and informal settlements. These communities are also more likely to have existing comorbidities, and are therefore more vulnerable to Covid-19.

If people can’t self-isolate at home, there is an urgent need and, indeed, a responsibility, to imagine a feasible alternative for facilitated self-isolation outside of peoples’ homes. Moreover, it is of critical importance that this is done within the window of opportunity before Covid-19 case numbers start peaking and overwhelming communities.

What might a good self-isolation facility look like?

Rather than large, sterile and clinical self-isolation centres, let’s imagine for a moment, that everyone who couldn’t self-isolate at home could be accommodated within their neighbourhoods — in an environment that was designed not to transmit Covid-19, while also being familiar, humanised and conducive to social and community bonds. These community care centres would be clean, dry, warm, and soulful spaces - where following the “five golden rules” of infection control is completely feasible. A space of compassion, humanity and solidarity, where stigma and misinformation have no place.

A place with a community kitchen, where meals can be prepared by aunties and uncles from within the neighbourhood. A spaza shop could be attached, where food donations are processed and sterilised, stocking the community kitchen, and sharing the surplus with those in need nearby. Perhaps there are open spaces for the healthiest of the patients to interact, designed in a way to ensure the virus is not spread but solidarity and support is. A space well equipped with handwashing stations, and separate entrances and flows for volunteers and patients with different levels of illness to interact safely, without spreading the virus.

A space which considers the needs of people staying there, including medical needs such as co-infections of HIV and TB, needs for assistance with accessing government grants, and mental health needs. They should be conceived of as spaces of hope, of collective resource sharing and even small-scale income generation for the people dedicating their time to creating them. A space where community members, faith leaders, street champions, artists, musicians and local business owners can come together and do what they already do best - take care of one another.

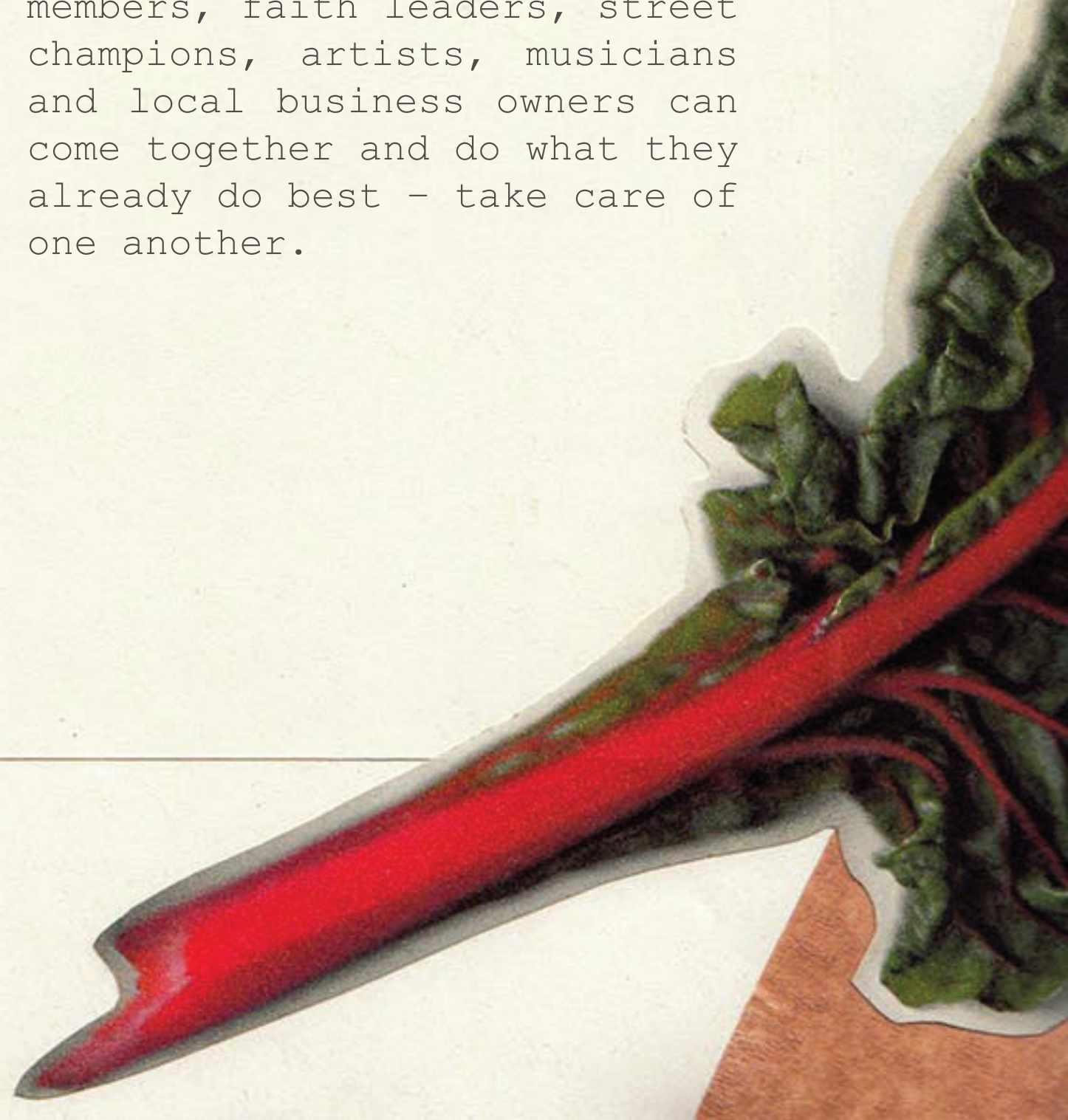

Importantly, these spaces must be developed from within neighbourhoods and communities, with guidance from other communities and spaces who have successfully done the same. In Cape Town, the Community Action Networks that have sprung up across the city in the wake of Covid-19 could be mobilised for this. Indeed, some CANs are already thinking along these lines — especially when it comes to tackling stigma, fear and misinformation, which many CANs are reporting is on the rise in communities.

These ideas are not new or untested. While Covid-19 is a very different disease with different considerations, community care centres played a critical role in providing care and reducing transmission of Ebola in countries confronted with this epidemic in 2014 and 2015. These experiences provide a wealth of knowledge that can be tapped into and adapted to the local setting for Covid-19 care and control.

Experiences in Sierra Leone show us what happens when care centres are set up as sterile, mysterious "facilities".

Misconceptions, fear and stigma abounded, as these facilities were perceived as places where people go in and never come out. Many were set up by mistrusted government bodies or NGOs that had parachuted in, with zero local footprint or trust. In many instances, buy-in from community members was sought far too late and the consequences were disastrous, with people fearing seeking care or diagnosis due to high levels of rumour, fear and misinformation that surrounded "official" quarantine centres.

On the other hand, several examples of a decentralised, community-owned model of care centres were documented, and the evidence suggests that these were both more feasible and more effective.

Such a model would need the government to change gear, to invest in the fundamental systems of life support - food, shelter and community. If funds are constrained, it may require a rethink of the balance between how much it spends on procuring a limited number of ventilators and other hospi-centric interventions versus more community-based approaches. It would require collaboration across the whole of society - bringing in other expertise to balance out a clinical approach to Covid-19 with a social one. Much of this expertise already exists at the community level. It might need a bit of training in basic virology and epidemiology, but it's a resource that we desperately need to see us safely through the next few months.

For a limited number of patients there will be a need for hospital-based solutions, but this virus will be defeated not so much by hospitals, but by communities acting creatively and responsibly to enable its isolation. Our strategies must reflect this.

By Leanne Brady, Axolile Notywala, Ayesha Ryklief, Nkosikhona Swartbooi, Manya van Ryneveld, Eleanor Whyle

NANA

Ekuphumleni CAN

Khayelitsha

NIGERIAN CHICKEN JOLLOF RICE

ingredients :

6 medium tomatoes sliced

1 red pepper

2 small red onions 1 quartered, 1 diced

½ cup vegetable oil

450g boneless chicken thigh cubed

2 tsp curry powder

2 cups rice

4 cups water

Preparations :

Cook the rice in 4 cups of water

In a separate pot add tomatoes, red pepper, quartered onion and pepper and blend until smooth

Over medium heat, add 2 tbsp oil, diced red onion, chicken and curry powder. Cook until onions are soft and chicken browned, about 8-10 minutes.

Raise to medium high heat and add blended tomato and pepper mixture.

Add back the chicken, cooked rice and remaining oil and fry 8-10 minutes

Cover with a lid and reduce the heat to low

Cook on low heat for 30 minutes

Serve while hot or cold

Enjoy!

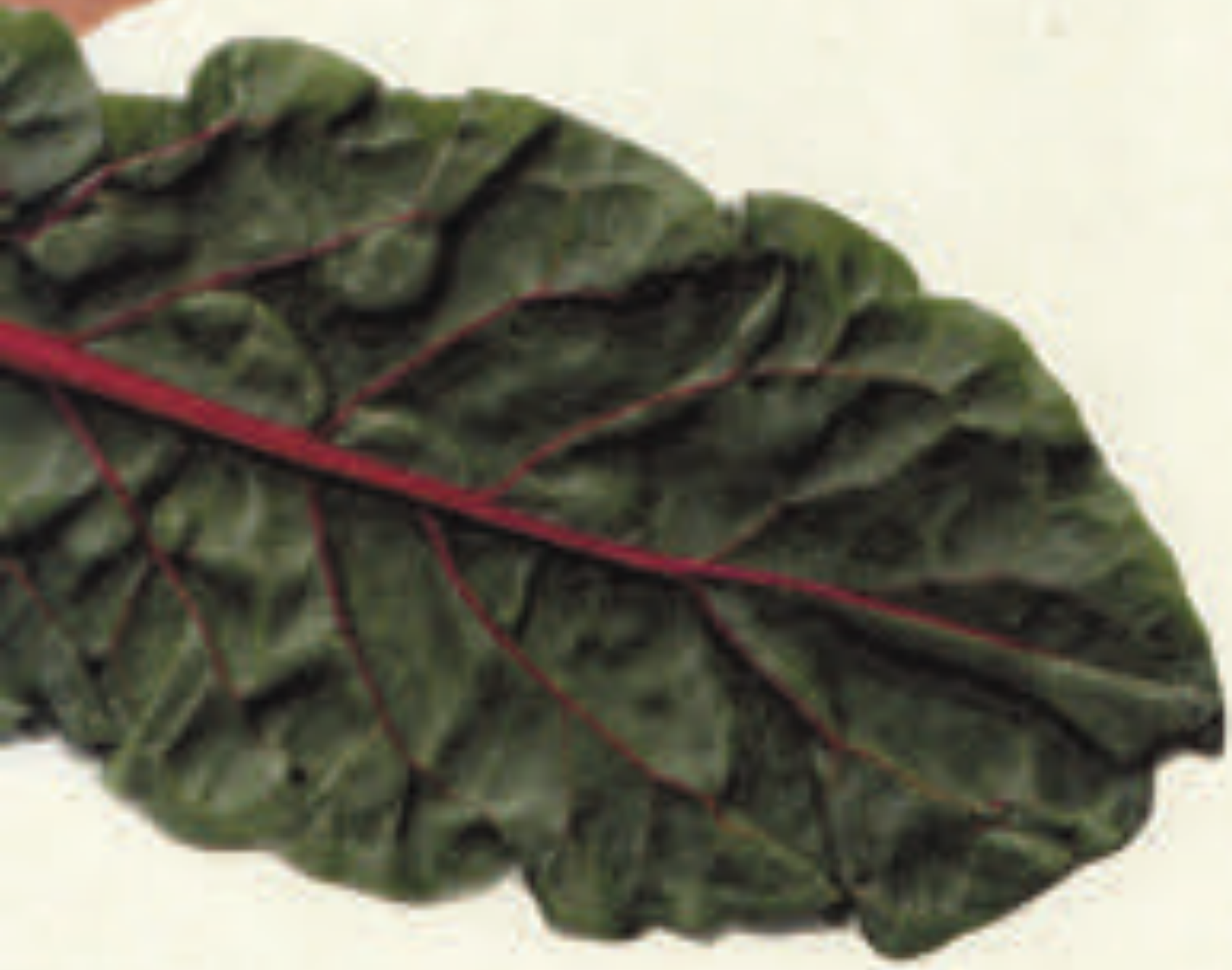

The cure for xenophobia
is jollof rice.
- Nana Odumuko
Ekuphumleni CAN

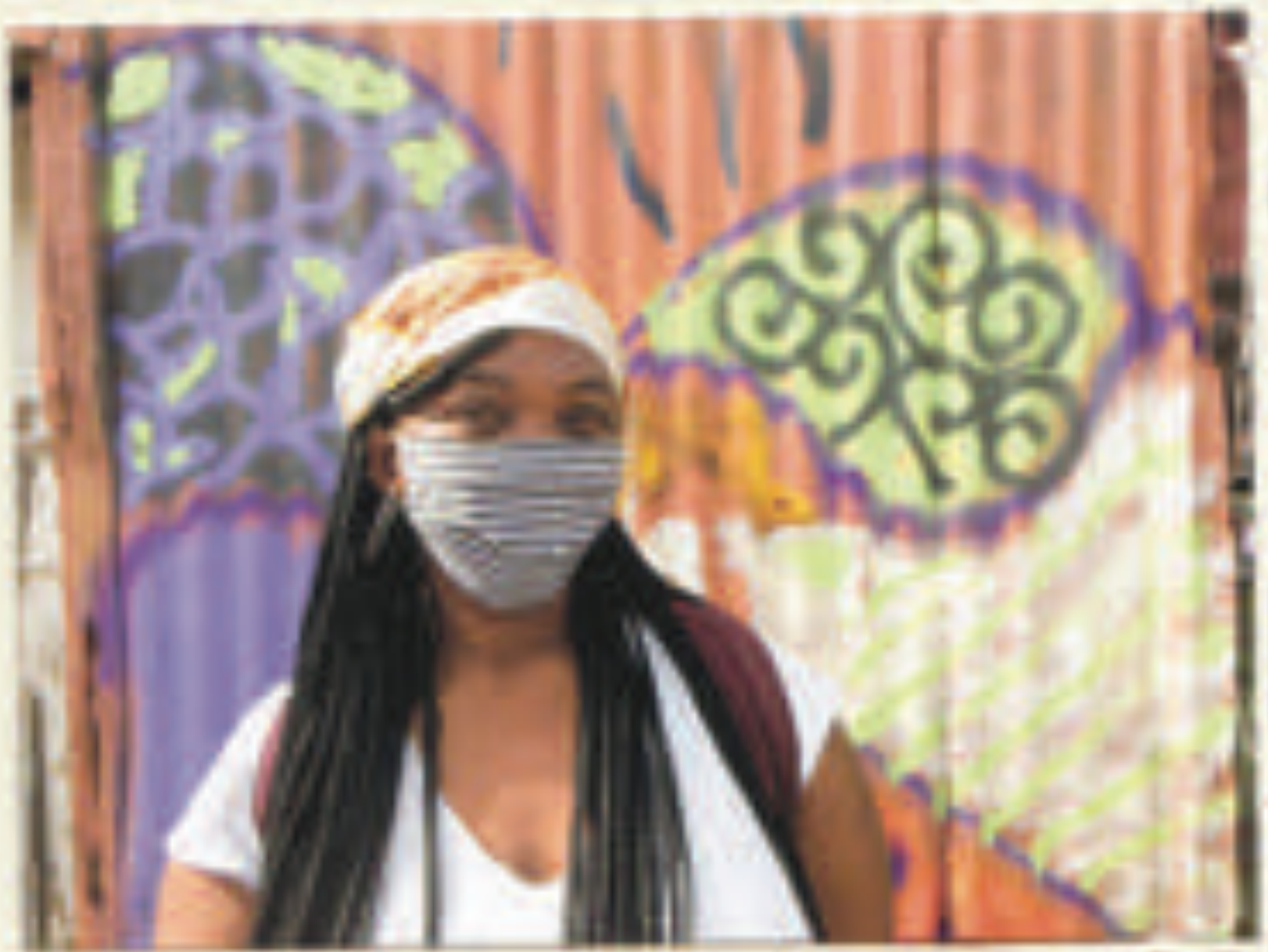

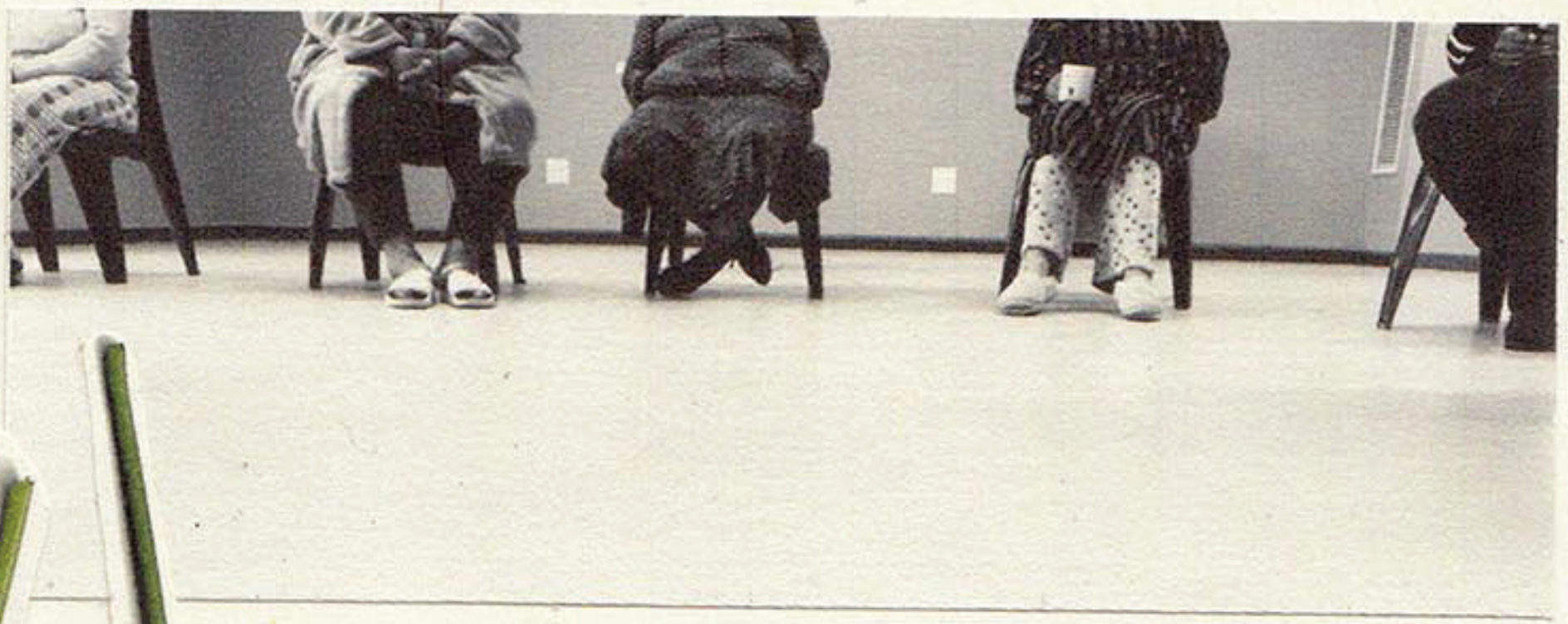

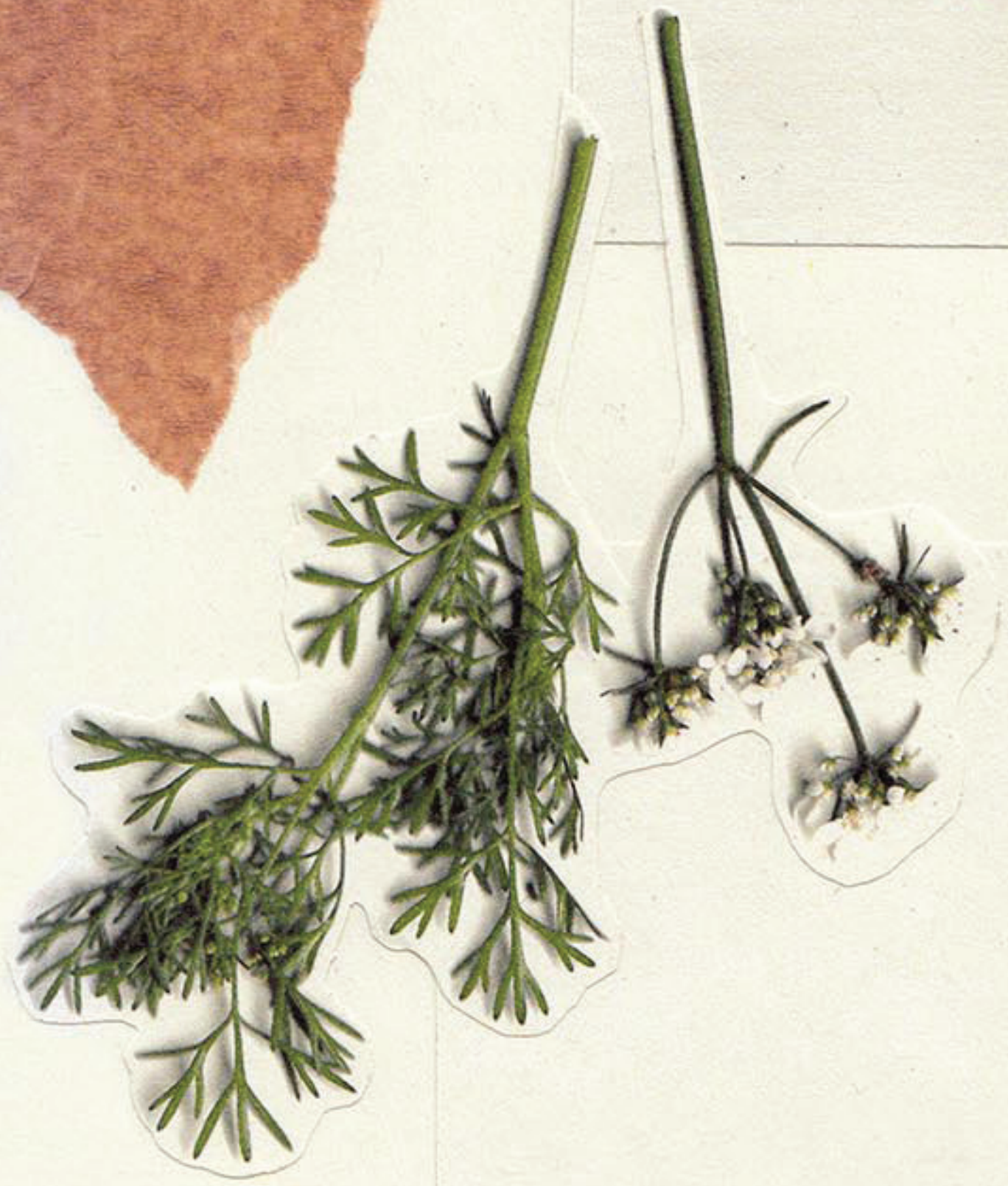

Isolated Possibility

There were things
we knew.
At least cerebrally,
we understood;
The shock and
isolation,
The unknown as both
friend and foe.

The impossibility of
geography,
Homes as refuge
and
simultaneously risk
possessed
proximity.

The unknowns
infinite.
Lived traumas,
loss
Struggle in survival,
matched grief.
Forgotten emergent
Pain
Amid despair.

Flirting with both
Possibility and
Overwhelm,
Fatigue and
Weariness
Instruction fraught
Orders and
Freedom in chaos

We built.

Conscious decisions
on
Safety with compassion,
Care into exchanges,
Within our power,
Dignity.
Building
recovery with little,
But listening,
together.
Heaving that
which brings,
proximity.
Woven links of
Knowledge to
Resource.
And finding
Kin-connection,
transcending
Communal, real
Possibility

for momentary relief.

By Suhair Solomon

At the heart of all systems is a network of people and relationships that keep the system moving. The relational power of the CANs intersecting with the health system has been hugely important as a bridge across networks in times of crisis.

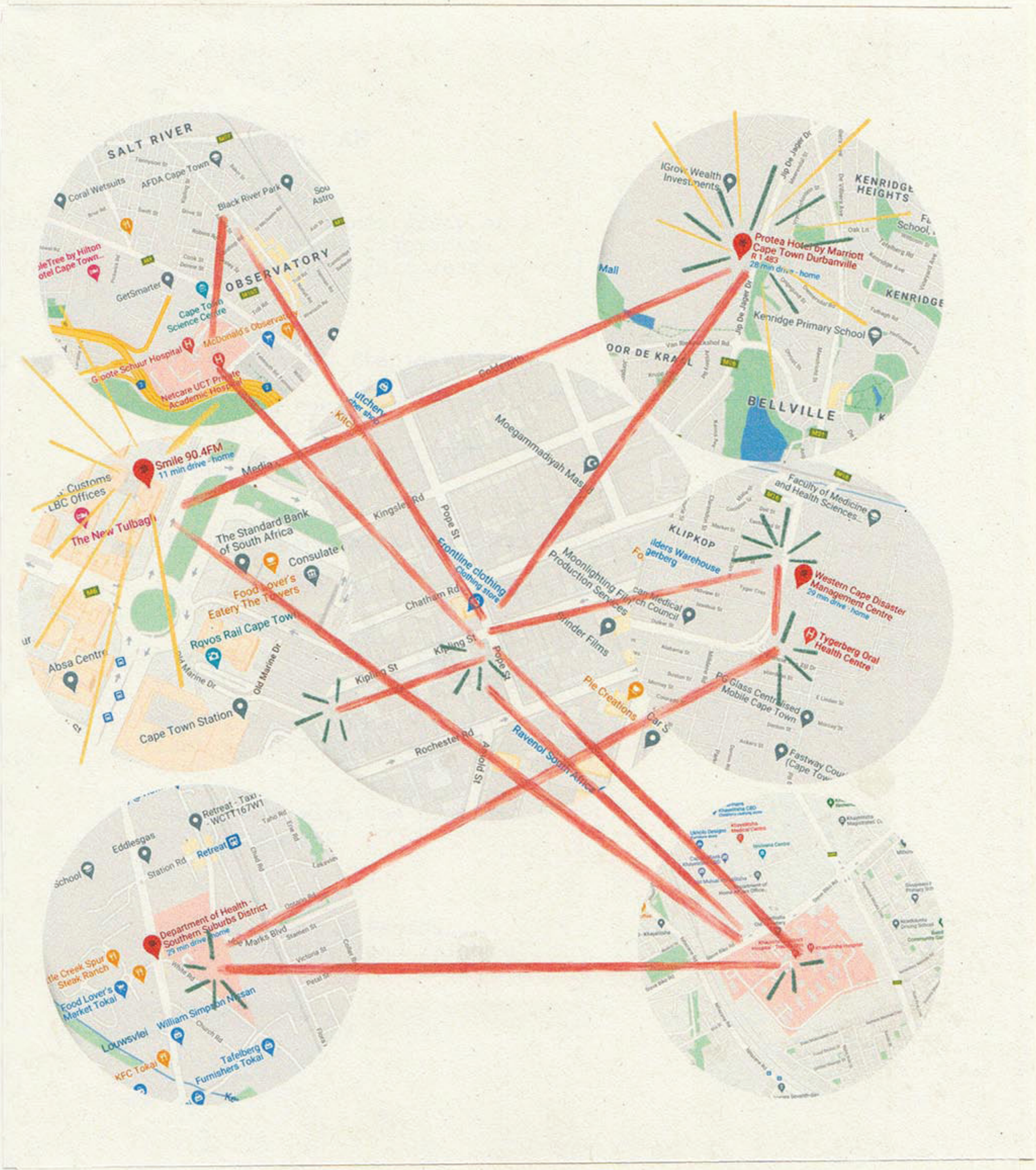

This image depicts the many connections between public servants and CANs that were necessary to enable testing and safe quarantine for a woman in Salt River who was experiencing Covid symptoms and needed a safe place to stay to limit the risk to her vulnerable parents and son.

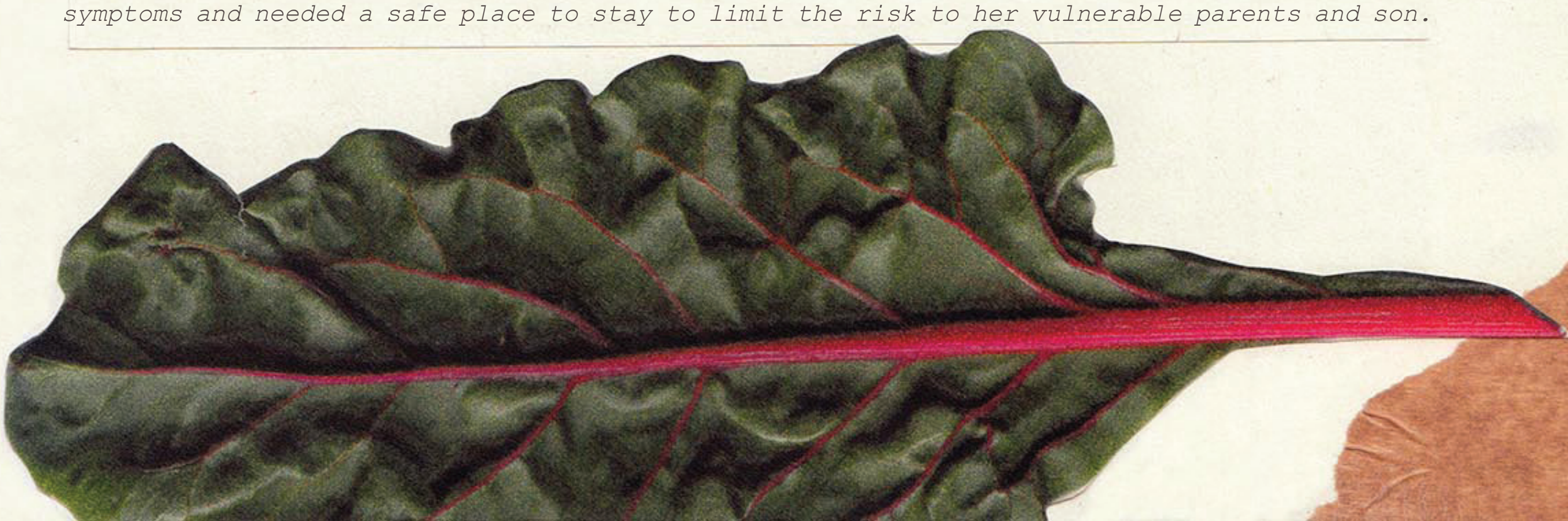

Home away from home

Ocean View CAN

South Africa, like many countries around the world, opted for a highly medicalised, hospi-centric approach to the Covid-19 pandemic. Given that only about 15% of people testing positive for Covid-19 need hospital care, and that preventing the spread of the virus is critical to controlling the pandemic, this approach makes little sense. Unlike many high-income settings, on which our response was based, the majority of people living in Cape Town are not able to safely self-isolate in their own homes. Many people live with extended families, with elderly parents or grandparents, or in houses shared with multiple families. There are also many families who do not have running water in their homes, and do not have the finances to stock up on food as daily wages are essential for survival.

The government's solution for the remaining 85% of people who simply needed a safe place to self-isolate and minimal clinical care was to rent big hotels for quarantine and isolation facilities, and convert a large conference centre into field hospitals. But it quickly emerged that, despite the vast expense of doing this, the occupancy rate of most of the hotels was only 30%. Most people did not want to leave their families, homes and neighbourhoods when they got sick.

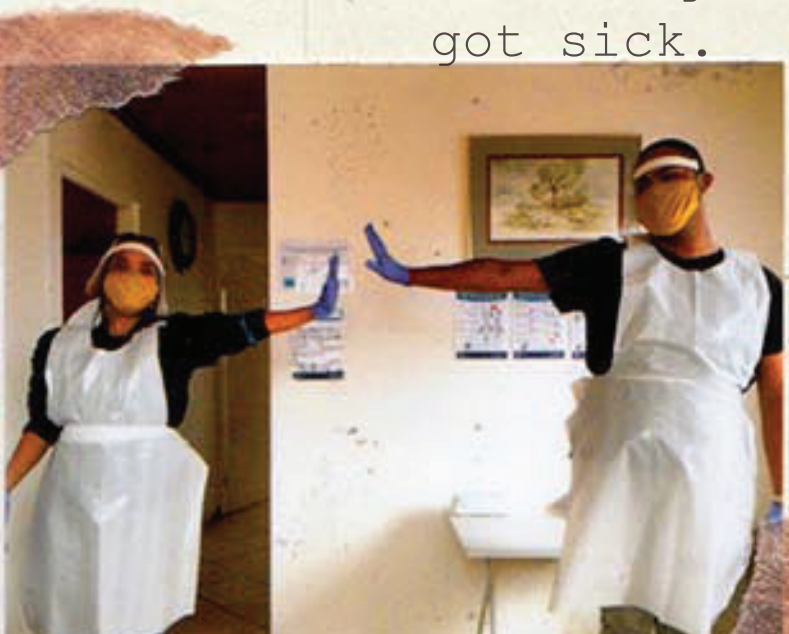

Building on experience from other global pandemics, CANs from across the city started to share ideas about what alternatives for self-isolation could be possible at the neighbourhood level. Meeting regularly as the Health CAN, a collective of CANs, healthcare workers from the public and private sector, scientists and researchers developed a model for neighbourhood-based care called Community Care Centres (CCCs).

In this story, Ricochet Van Rensburg, Ramona Lamb, Teagon Klein, Tonique Andrews, Eben Collins and Craig Butler share some reflections on setting up a CCC in Ocean View and caring for their Covid-positive neighbours.

Early in the pandemic, we knew that most people in our neighbourhood would not be able to self-isolate in their own homes, so we decided to set up a CCC to support them. Using the online training pack put together by the Health CAN as a guide, we started planning our first steps. We learnt about the virus, how to use PPE (personal protective equipment) safely, and how to do daily monitoring for early warning signs so we knew when to refer someone to the hospital. Because the CCC was for people who would otherwise be recovering at home, the main task was to make sure we created a welcoming space for people who got Covd-19, where families could safely come visit or drop off supplies. But, we wanted to make sure we were also on top of the clinical stuff and needed to get PPE training so we didn't get infected with Covid-19 ourselves.

Our biggest challenge in setting up the CCC was finding a venue. We struggled for weeks, and despite many attempts, could not get permission to use any of the churches or public spaces in Ocean View for the CCC. Then, because the creche that Ramona usually runs from her home was closed due to the pandemic, she suggested we convert it into a CCC. Now that we had a space, we could quickly jump into action.

The next important step was to set up a local network of care - this included the Ocean View CAN, and the local health system and luckily, we already had some connections there. Ricochet and Tonique had been volunteering with Emergency Voluntary Services (EVS) for years, and also had good relationships with Deon Terry, a paramedic who worked for the Provincial Health Department. We also needed to connect with the Ocean View clinic and False Bay Hospital so that we had a good referral system, and having Leanne Brady from the Health CAN, who was also part of the Provincial Health Department, part of our team was helpful for that. Then we had a series of WhatsApp groups with doctors living outside of Ocean View who could offer us advice. Although we ran and managed the CCC on our own, it was important to know that we had these support structures "in our back pocket", as Ricochet would say, in case we needed help.

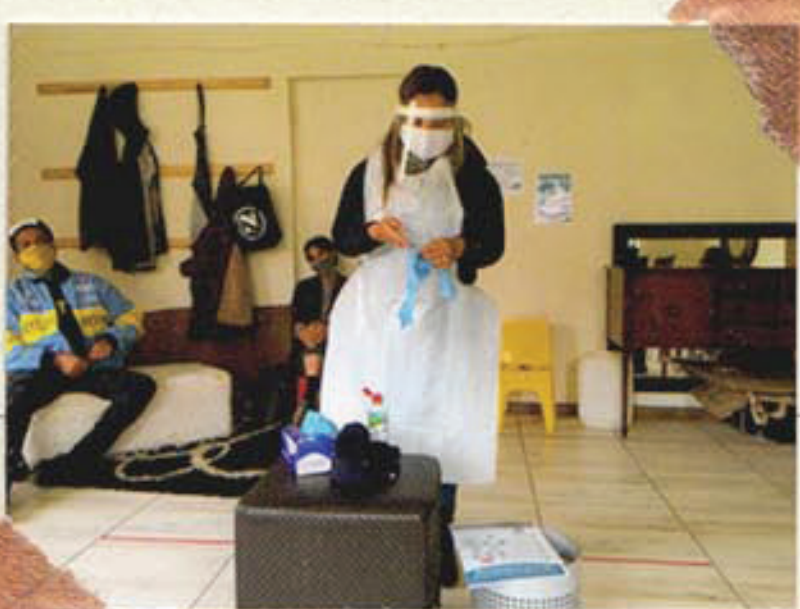

So, we got to work gathering supplies and started preparing the home. 20 beds were donated, and we received lots of support from other CANs who came to drop off bedding, curtains, basic equipment such as PPE and pulse oximeters and even a washing machine. Everything else somehow fell into place. Within a few days, Teagon had developed a delicious menu and Tonique, Eben and Ricochet started planning for the night shift, getting excited about the snacks we would eat, and the TikTok videos we could make.

Before we opened, we wanted to tell Ramona's neighbours about the CCC and invite them to the launch. Most of the neighbours were glad to know there was somewhere nearby in case they got sick, but some neighbours were fearful about having Covid-positive people nearby. They were feeling angry, and even threatened the CCC. Craig and Ricochet met with them, trying to explain why the CCC was important. One of the biggest challenges we had, was basically changing people's minds around here… people were afraid, and they thought we were getting money for doing this. But, after a series of 'open days' and neighbourhood conversations to bring everyone on board, the CCC was ready to open.

One of my favourite memories is when we actually got our first resident. To see how everyone just jumped on board and did their thing. There was fear - we all had that fear within us because we didn't know what to expect. But that, for me, was the best moment, everyone just jumping in and doing their thing.

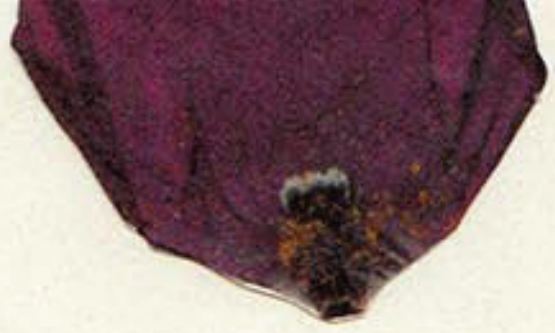

In the end, we stayed open for a few months. And, even though we only had a few residents, it was great to offer them a safe space to recover. Also, our small CCC had a much bigger impact than we expected, and maybe that's because it demonstrated that a local, neighbourhood level of care was actually possible – that big expensive empty hotels for isolation and quarantine were not the only solution, and that we could look after each other with just a little bit of support. Other CANs came to visit to learn more about how we operated, the CCC was in the news, and was even discussed by people in other provinces.

The small home away from home in Ocean View had powerful ripple effects on the Covid-19 response. It was a special time for us too and even after we finished a night shift and could go home to rest, we chose to stay and hang out with each other instead. While we set out to create a 'home away from home' for people with Covid-19 in Ocean View, we actually ended up creating a 'home away from home' for each other.

It should be said that this model of neighbourhood-based care, led by community members, with the enabling support of the formal health system, was not received well by some politicians in power. This is despite the evidence that shows the importance of local interventions that not only provide care, but also allow for awareness and knowledge mobilisation in a collective and horizontal manner, rather than trying to force behaviour change from the top down. While a pandemic opens up spaces for possibility to do things differently, and the CCC certainly received lots of local support and sparked lots of ideas of how to focus on supporting local interventions – we can't deny that health is political. When ordinary people offer better care than the state can, sadly, for some, this is seen as a threat.

By Ricochet Van Rensburg, Ramona Lamb, Teagon Klein Tonique Andrews, Eben Collins, Craig Butler and Leanne Brady

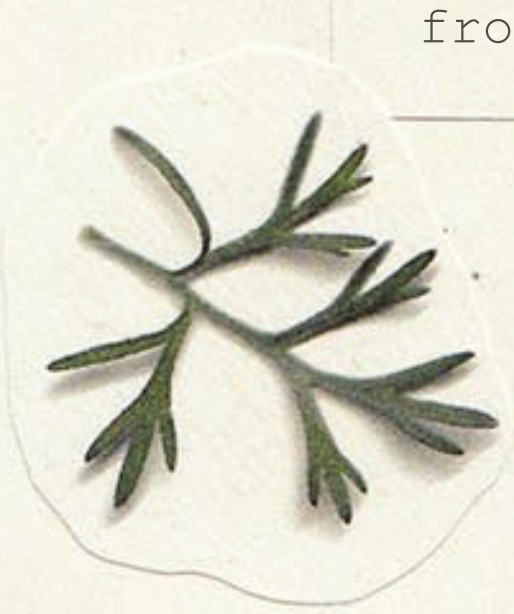

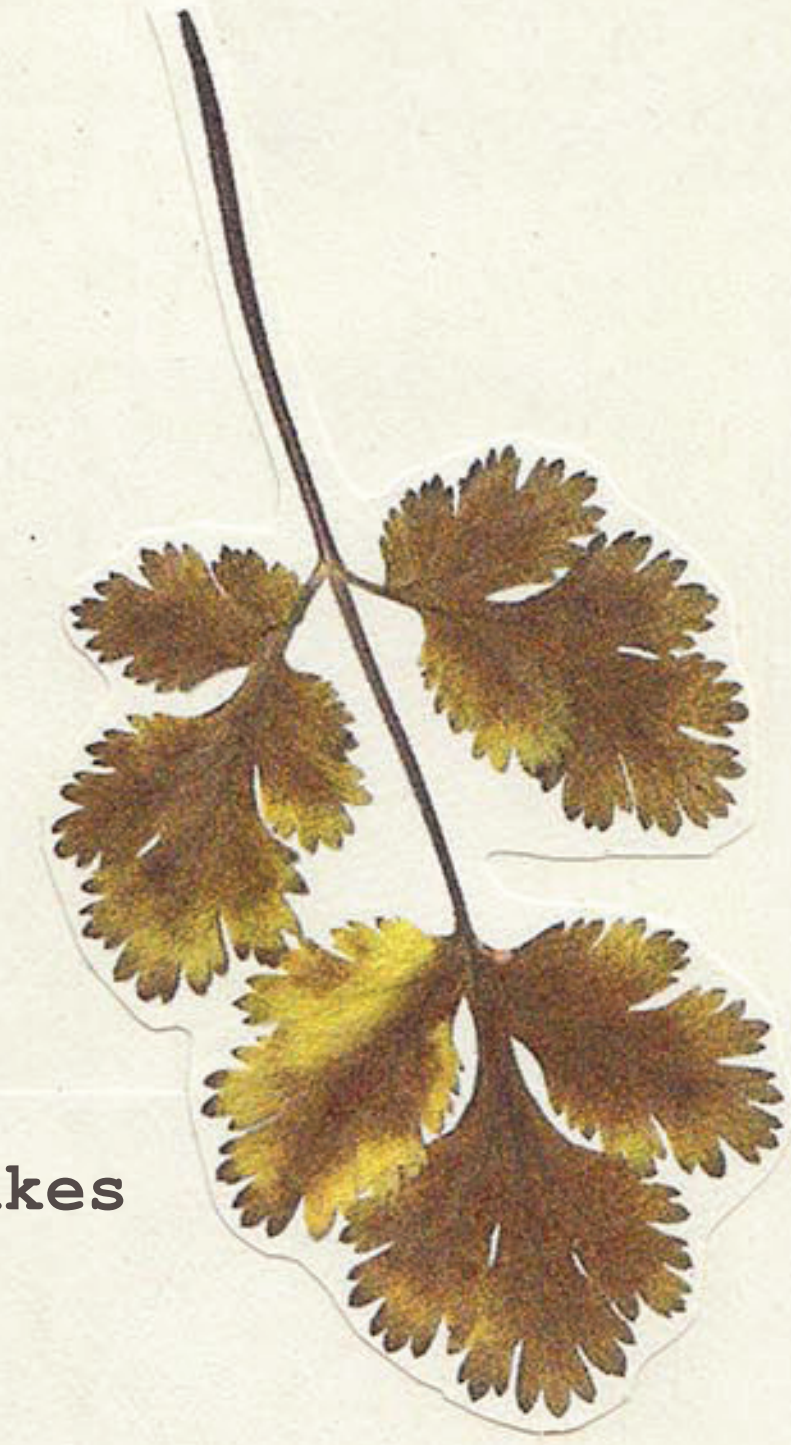

Cheesy Fish Cakes

Ocean View CAN

- 2 tins of pilchards
- 6 bricks Weetbix
- 1 large onion
- 1 small green pepper
- 1 small red pepper
- 1 small yellow pepper
- 50ml sweet chilli sauce
- 30ml chutney
- 3 eggs
- Parsley to taste
- Grated cheese to taste
- Pinch of salt
- Pepper
- Oil for deep frying

Finely chop the onion and peppers

Rinse the fish and remove the bones

Add the onions, pepper and fish into a bowl and mash together

Add salt, pepper, chutney, sweet chilli sauce and parsley

Crumble the weetbix into the mixture

Add the eggs, cheese and mix well

Shape into small or medium balls

Deep fry until golden brown

By Teagon Klein

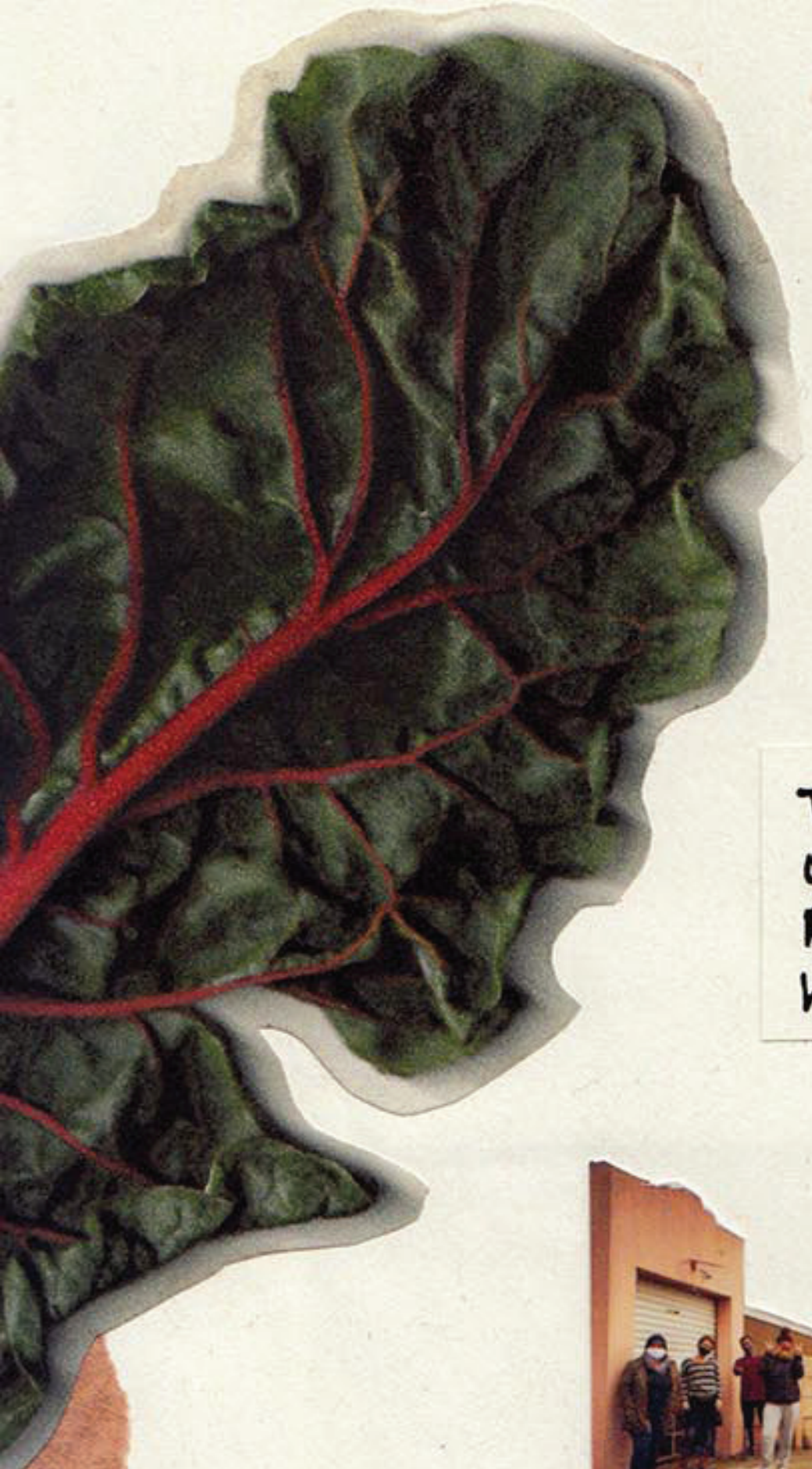

The CCC crew outside Ramona Lamb's house

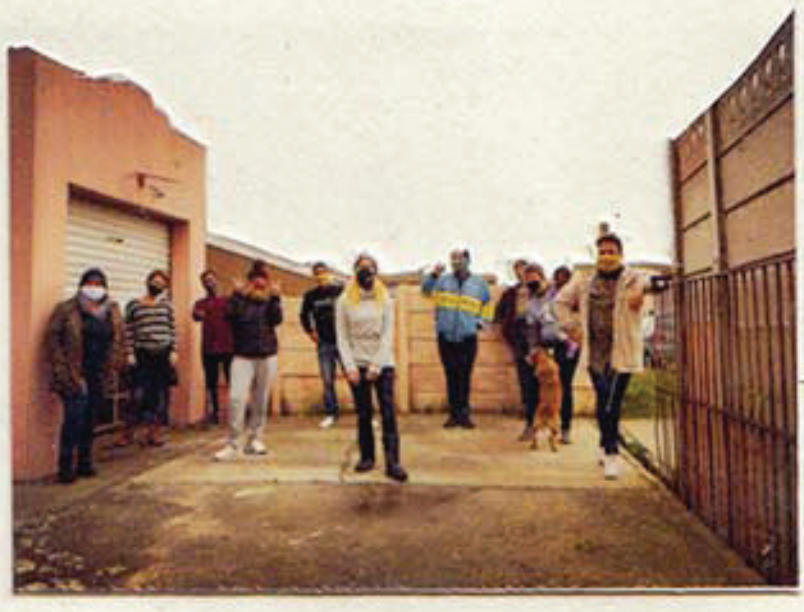

Cooking up a network: Perfect for any crisis

Connecting CANS

This modern take on a classic solidarity recipe is exactly what you need when catering for a year like 2020. Flavourful and loaded with tender stories, this network recipe stews in a rich and vibrant city, and produces some of the tastiest meals you'll ever eat!

INGREDIENTS

- A couple dozen social activists and community organisers (add a few trusted troublemakers for flavour)
- A healthy portion of ordinary people (start with hundreds and work up to thousands)
- Intersecting web of relationships
- 3 x digital tools - WhatsApp, Facebook, and Zoom
- A few bicycles
- 4 x large helpings of Action, Love, Care, and Courage
- A little revolutionary energy

INSTRUCTIONS

1. Preheat City with 400 years of oppression.

2. Bring to boil with crisis (a corona virus will do, but Cape Town is a pressure cooker and existing inequalities will work too).

3. While the City is heating up, make sure to identify existing networks of relationships of trust - use these in the next step.

4. Once at a boil, combine activists, organisers, and troublemakers in the pot. Mix with digital tools and start adding ordinary people from as many different parts of the City as possible (the recipe will flop without diversity).

5. At this stage, focus on getting tangible things done (add the Action!). Relationships are formed and trust is built as people work actively to assist each other through the crisis. Bicycles are an excellent tool for connecting across a divided City.

6. Stir the pot regularly. Let stories from the developing network emerge. Share these often - they are important sense-making moments.

7. Professors of the Street will start emerging. Allow them to develop by seasoning generously with Love, Care, and Courage.

8. Allow the network to cook for the better part of a year and you'll see some real magic emerge.

9. The network will keep while the crisis is hot, but to stay fresh beyond this will require a little revolutionary energy and the development of more long-term shared goals.

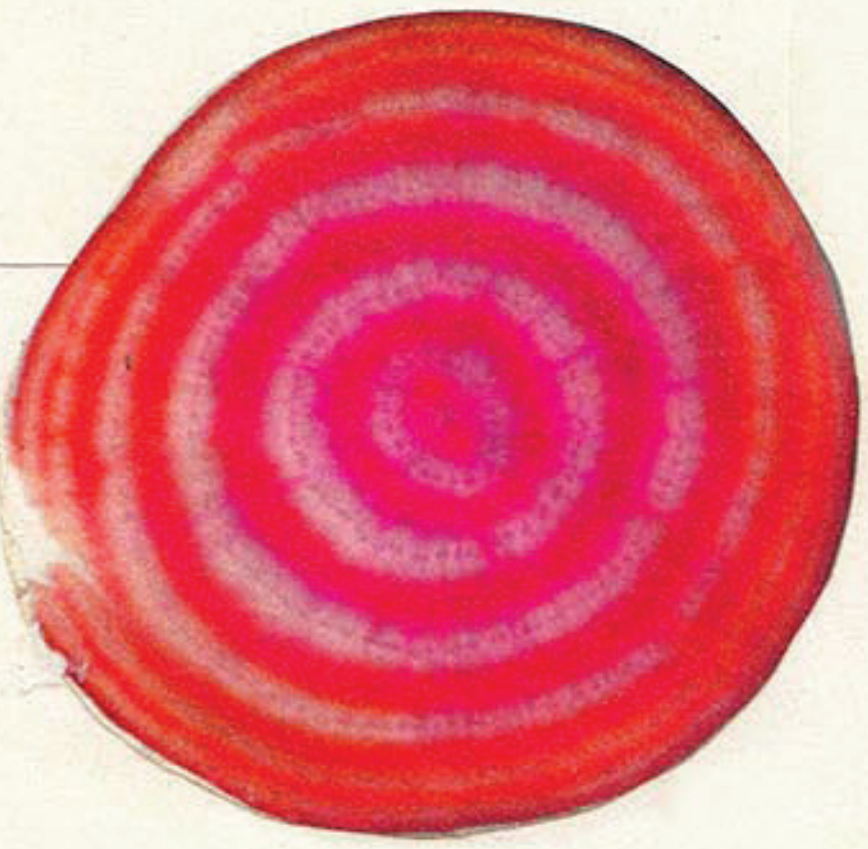

COOKING TIPS

When adding ingredients, be careful to prevent any one part overpowering the whole.

Some ingredients just don’t go together - be sure to pick out any hierarchy that falls into the pot (there’s often a lot of this floating around, and can sometimes be difficult to spot, so keep your eyes open!).

There are many nodes in a network, and these emerge as needed and disappear when they are no longer relevant. This is fine and healthy. Stay dynamic!

By Tyronne McCrindle

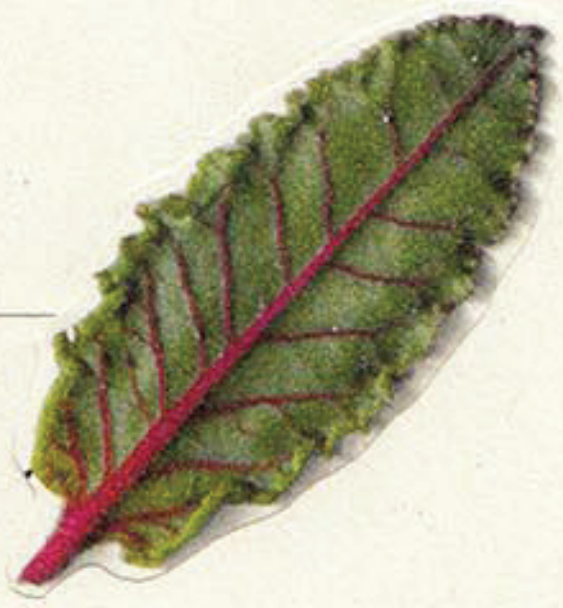

One of the problems with writing a cookbook is that recipes exist in the moment

-Thomas Keller

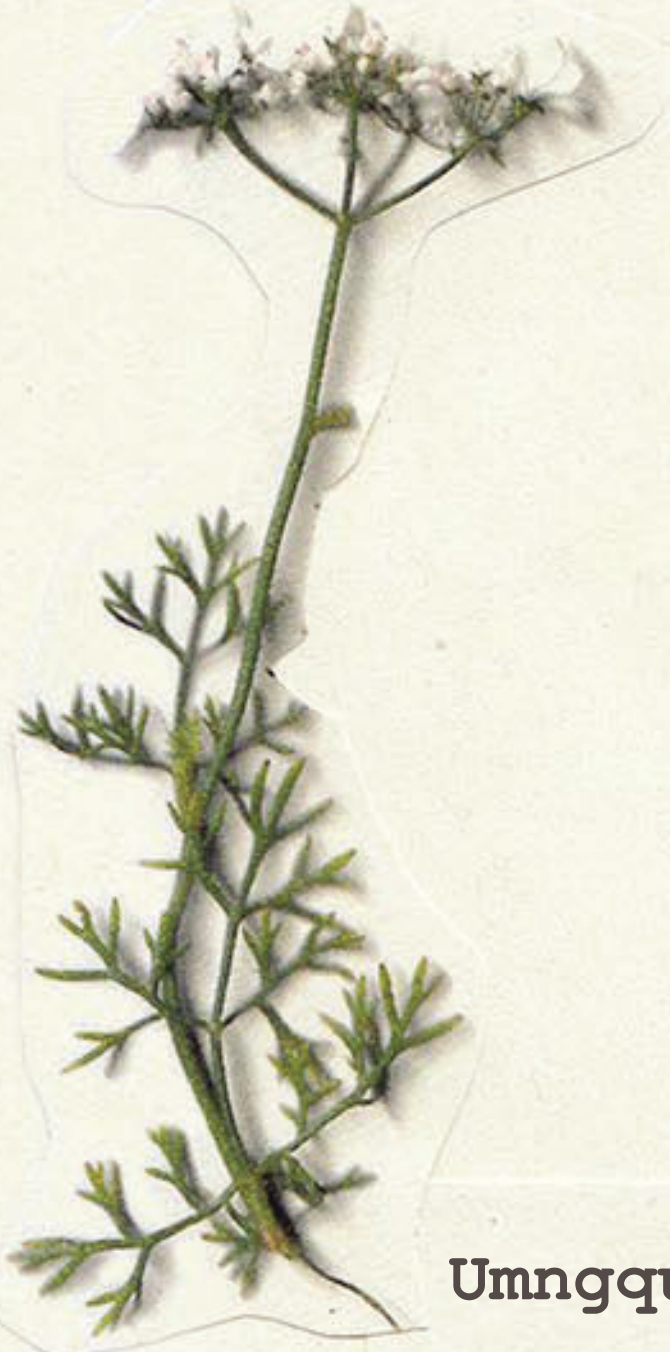

Umngqusho

Langa CAN

Samp with Spinach from Mama Lulu's Kitchen

- 10kgs white samp
- 3kgs onions
- 1kg potatoes, peeled, boiled and mashed
- 5kg carrot, grated
- 10 bunches spinach, chopped
- 3 packets of mushrooms
- 500grams Marvella (or butter)
- 2 packets Top Class (Aromat)
- 1 packet garlic (powdered)

Soak samp overnight

Rinse and cook white samp until soft

Grate carrot and sprinkle with cinnamon

Rinse the spinach with salt water

Fry mushrooms in Marvella and onion

Add spinach to onions

After 5 minutes, add carrot and fry together

Mix mashed potatoes and samp

Add spinach and carrot and mix in

Season with Top Class and serve!

By Lulama Mbewu aka Mama Lulu

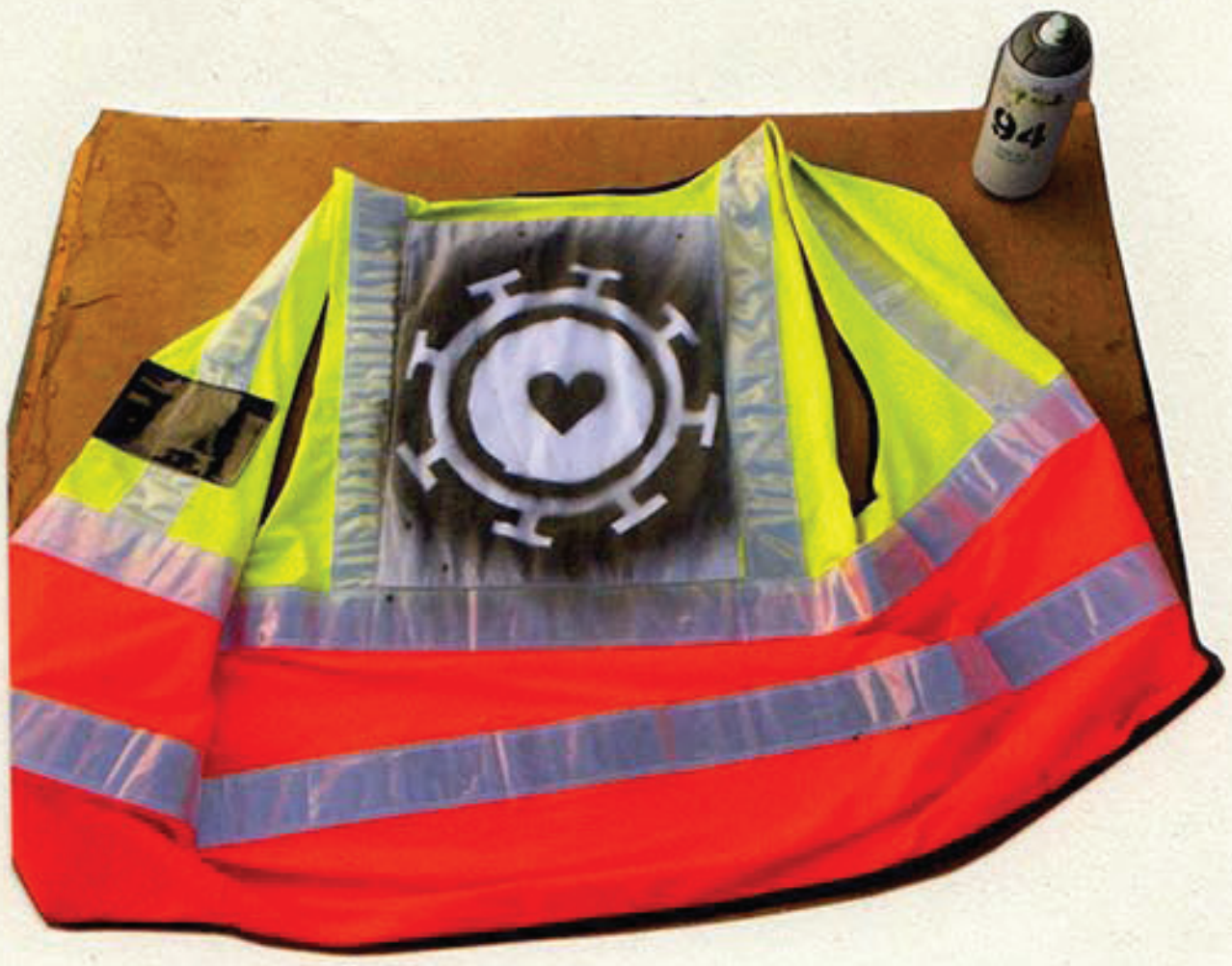

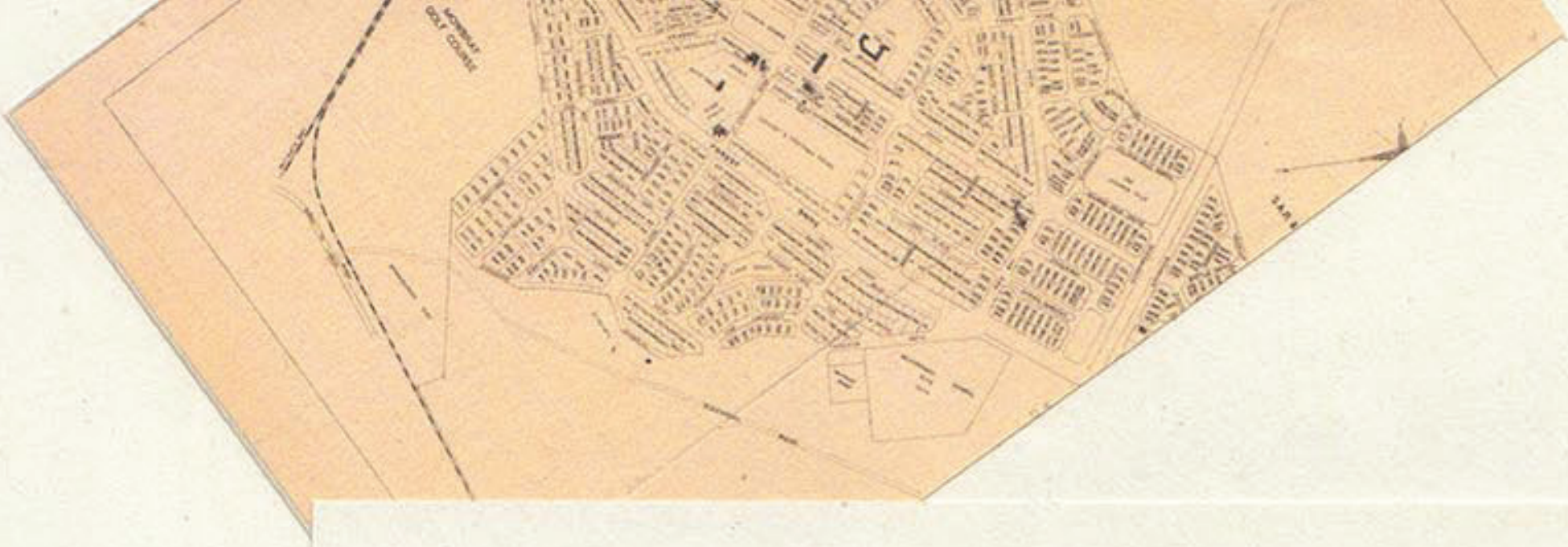

Cycling across divides

Langa CAN

One of the things about Cape Town is that things are never as far away as you might think. Years of entrenched apartheid spatial planning mean that you may never have set foot in certain neighbourhoods, depending on your race. It’s always easy to reason it away - “the city is so spread out”, or “I don’t know anyone who lives there”, or “everything I need is between here and the CBD”. But another insidious and invisible reason for why we don’t move through each other’s neighbourhoods is because there are literal physical obstacles in our way, put in place by an old and difficult to undo spatial planning mindset that keeps us in our racialised enclaves at all times.

The CANs pay no heed to this - they exist in neighbourhoods from Constantia to Crawford, Hout Bay to Hanover Park, Durbanville to Du Noon. With the network spread across the city, residents of Cape Town finally had a reason to visit each other’s neighbourhoods.

The CANs’ focus on hyper-local organising, either within one’s own neighbour-hood or in partnership with a few others, meant that, on one hand, the scale of movement was drastically reduced. In Kenilworth CAN we met in the local church, walking distance from all of our homes. In Langa CAN we used bicycles to deliver food and medicines.

On the other hand, the spread of the network across the city represented a massive and catalytic movement, not just of the concept of the CAN as an appropriate Covid-response, but of the underlying organising principles and vision for a city based on solidarity, radical generosity and togetherness.

As the first peak of the pandemic subsided around September/ October 2020, the Langa, Bonteheuwel, and Pinelands CANs organised a bike ride. It was a simple idea aimed at connecting up these three CANs by disrupting the glaring spatial arrangements that keep them apart. It’s about 7km as the crow flies from Pinelands to Bonteheuwel. Add in the highways, railway line detours and cul-de-sacs and those 7km become a lot more. The use of the bicycle was thus a strategic choice - a simple instrument to get from A to B in a city made for cars and segregation.

News of the event spread further than we could have imagined and the group that left from the Pinelands Sports Centre was well over 100 people - a jumble of bicycles, a fair amount of lycra, and a lot of overexcited kids. With the help of some spontaneous marshalling from the Hanover Park cyclists and the Langa Bicycle Patrol, we swarmed our way over highway bridges, across railway lines and through tunnels, stopping at each neighbourhood along the way to greet other CAN members and hear about their activities.

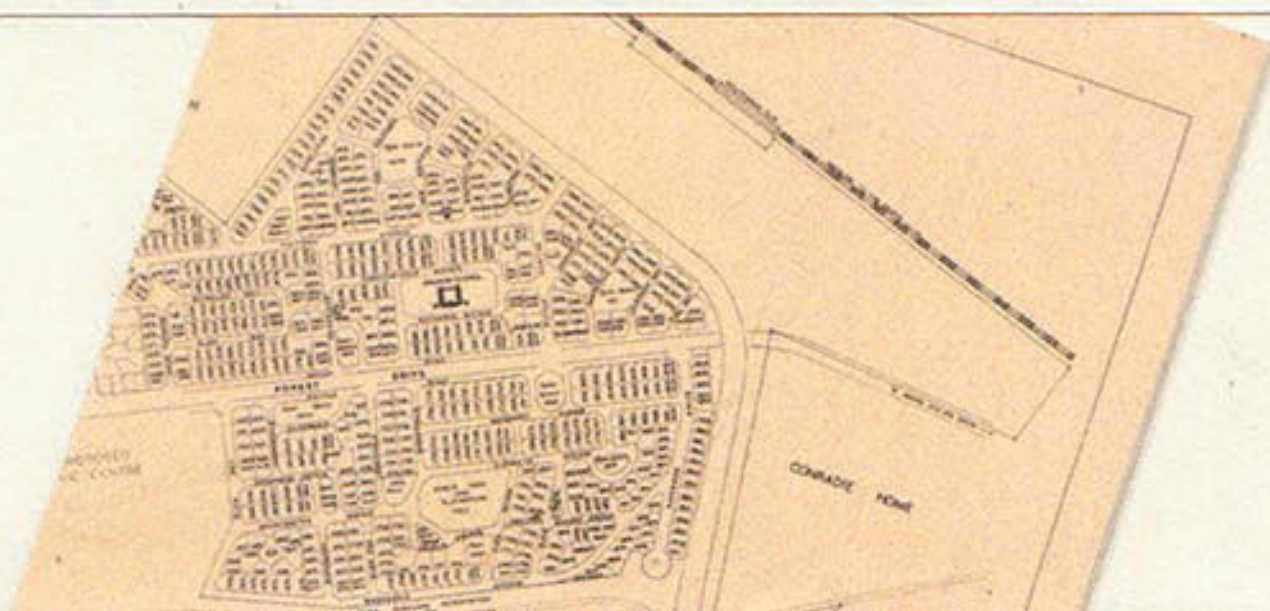

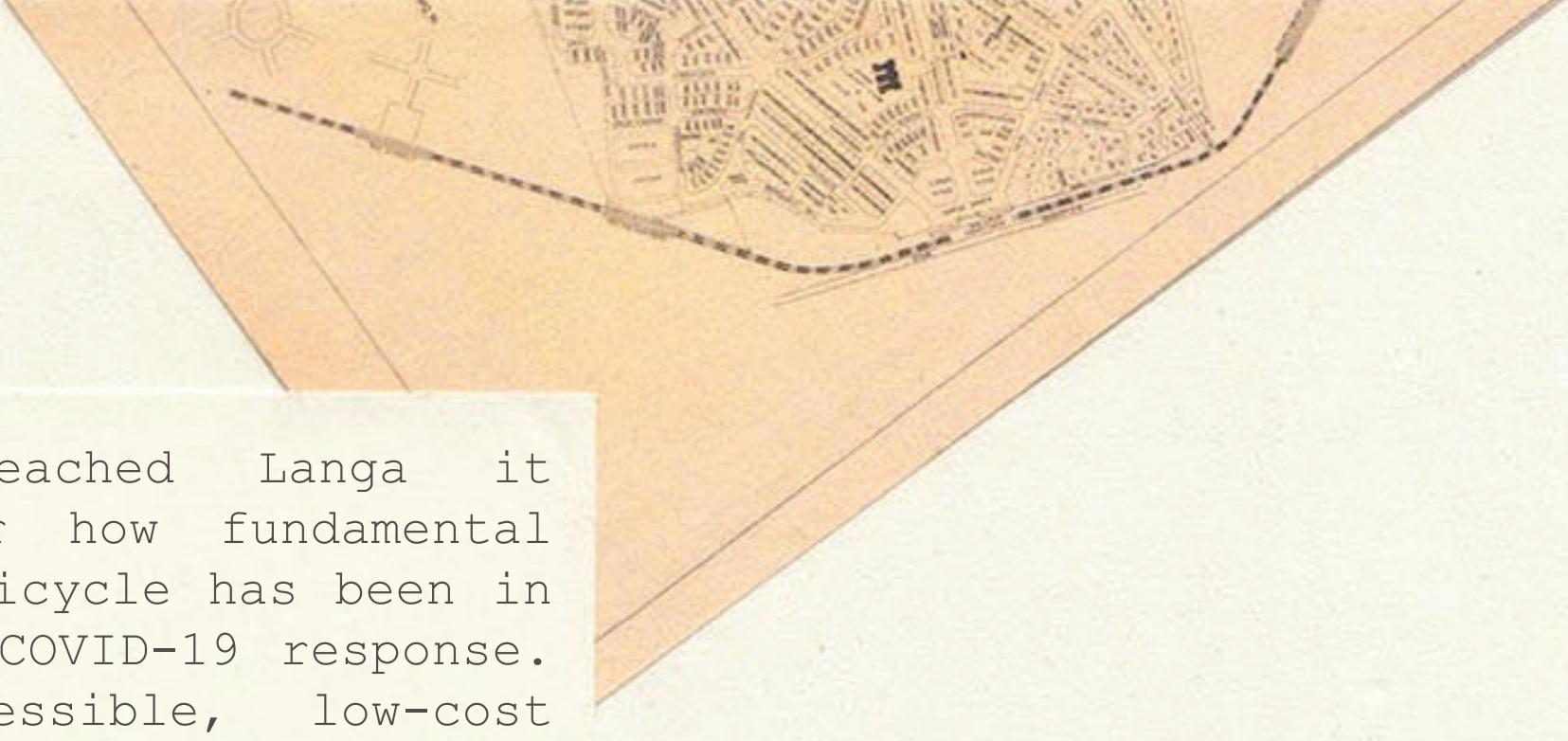

When we reached Langa it became clear how fundamental the humble bicycle has been in Langa CAN's COVID-19 response. As an accessible, low-cost mode of transport that is perfectly designed to connect people at the local level, the bicycle has made many of Langa CAN's activities possible. The bicycles were donated by the Western Cape Government, Khaltsha Cycles and Bike4all. They were given to the CAN's bicycle brigade, who designed food carriers that can be attached with cable ties to the bicycle frame. Food is cooked in the six kitchens dotted around the neighbourhood.

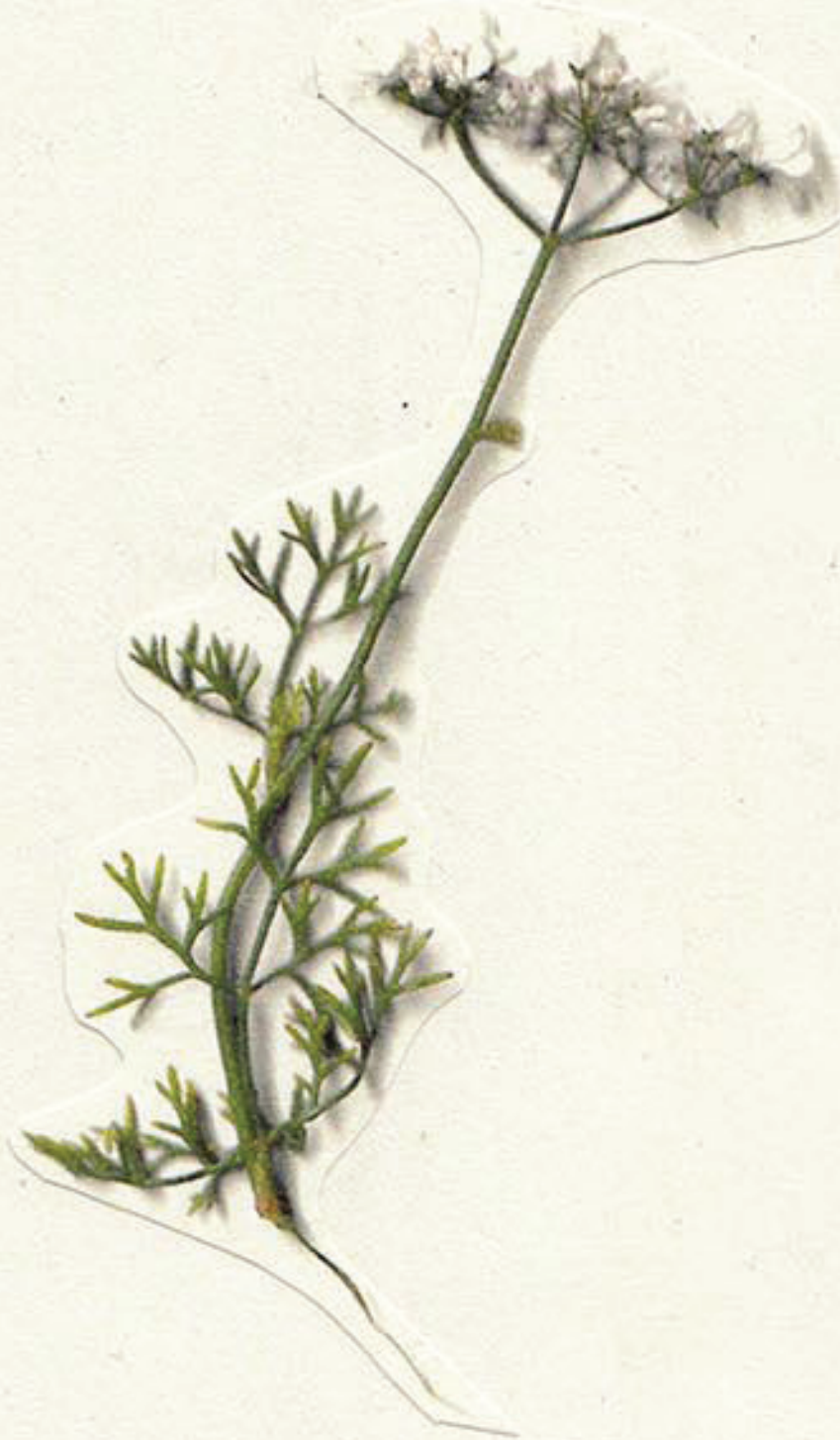

At mealtimes, cyclists from the bicycle brigade get on their bikes and make their way to each kitchen, picking up food parcels for people who cannot physically make it to the kitchens and delivering them to their homes. The CAN has also teamed up with a local non-profit clinic in the area to set up a similar delivery system for chronic medication. For Mzikhona Mgedle, one of the leaders of the Langa CAN and a bicycle champion himself, the bicycle represents the perfect vehicle for the CAN's activities, as well as its values and principles. Two wheels and a frame, fuelled by the energy of the rider. Connecting people street by street, subverting the anonymity and individualism that our city structures and infrastructures impose on us. And placing the power of local self-organising back into our hands and at our pedals.

By Mzikhona Mgedle and Manya van Ryneveld

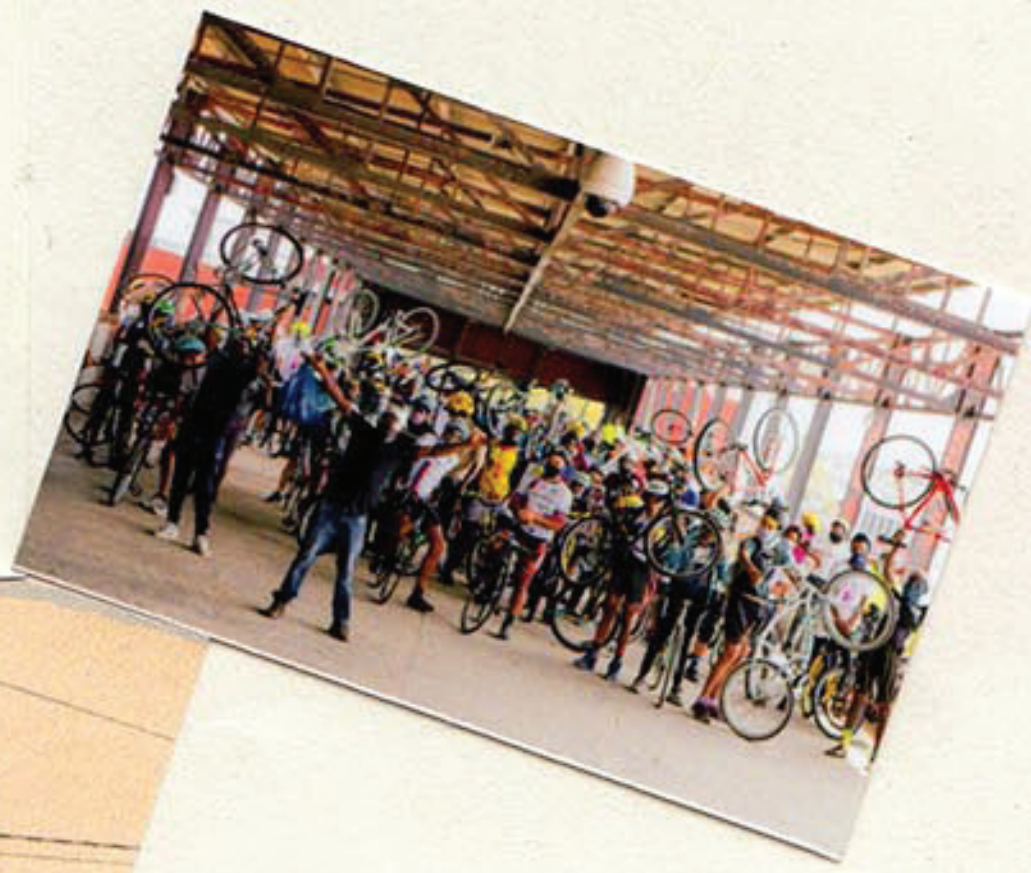

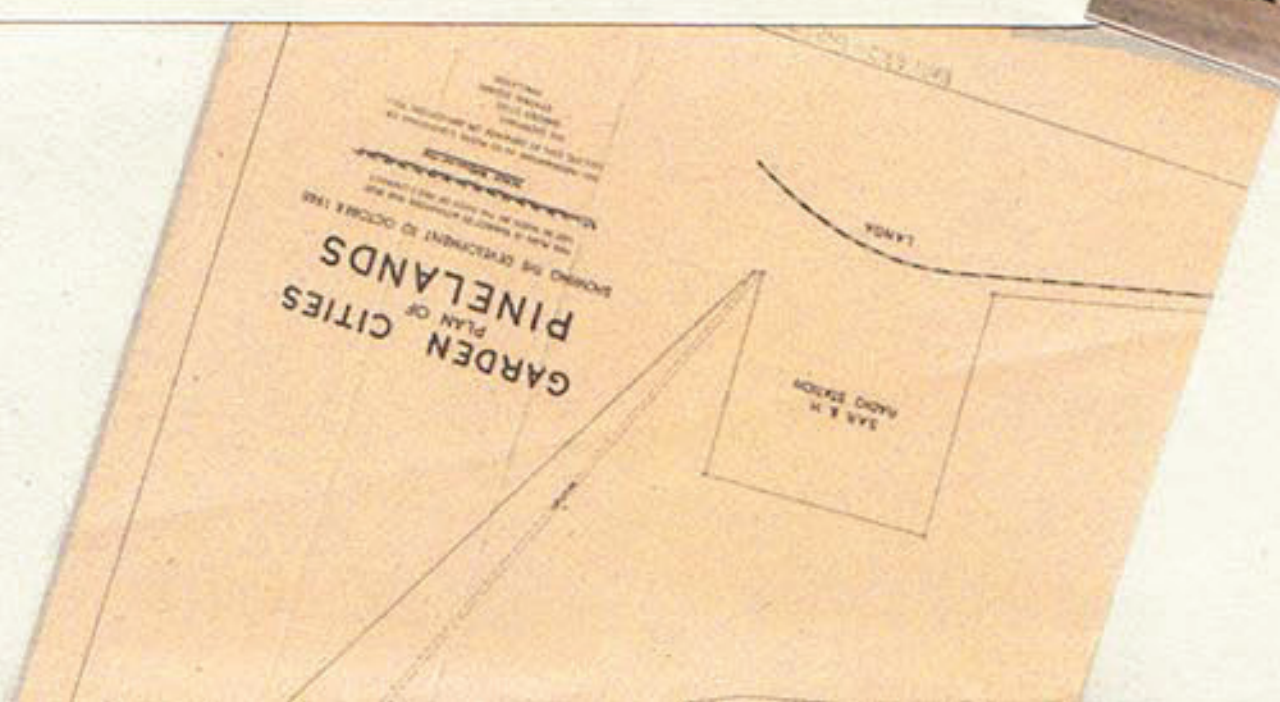

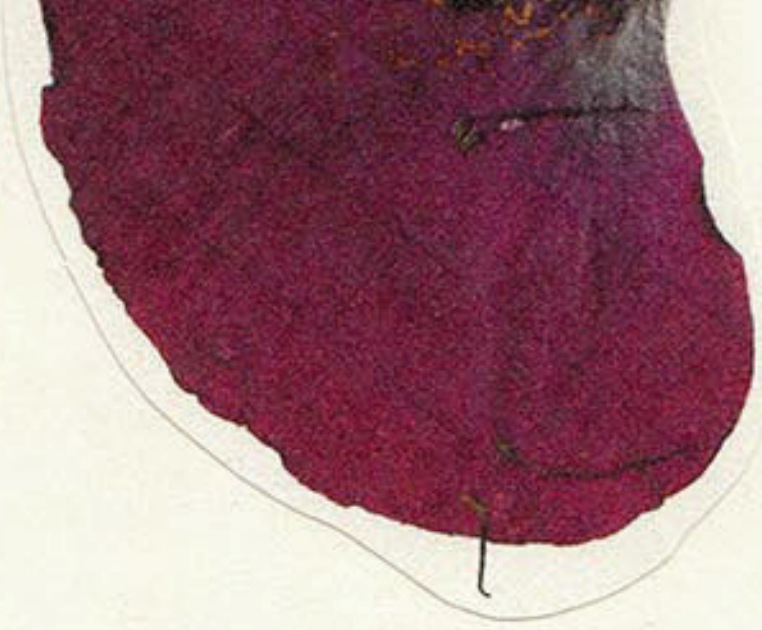

Vegetable Stew and Meaty Bones

Brackenfell-Kraaifontein CAN

- 3kg meaty bones
- 2kg potatoes, halved
- 2kg carrots, chopped
- 2kg onions, chopped
- 2kg spinach
- 10 litres vegetable or beef stock
- Salt and pepper
- Other spices you prefer
- ¼ cup oil

Make your stock

In a 25-litre pot, add the oil

Add the onions and braise until brown

Add the spices you choose

Add the stock

Add meaty bones and cook for 30 minutes

Add potatoes and carrots. Cook for 20 minutes

Add pepper and spinach

Taste it! If you want a more meaty flavour, add some bisto or more stock

Cook for 1 hour

For a soup consistency, add more stock. For a stew consistency, add split peas or beans to thicken, and serve with bread or rice

By Lynn Hendricks

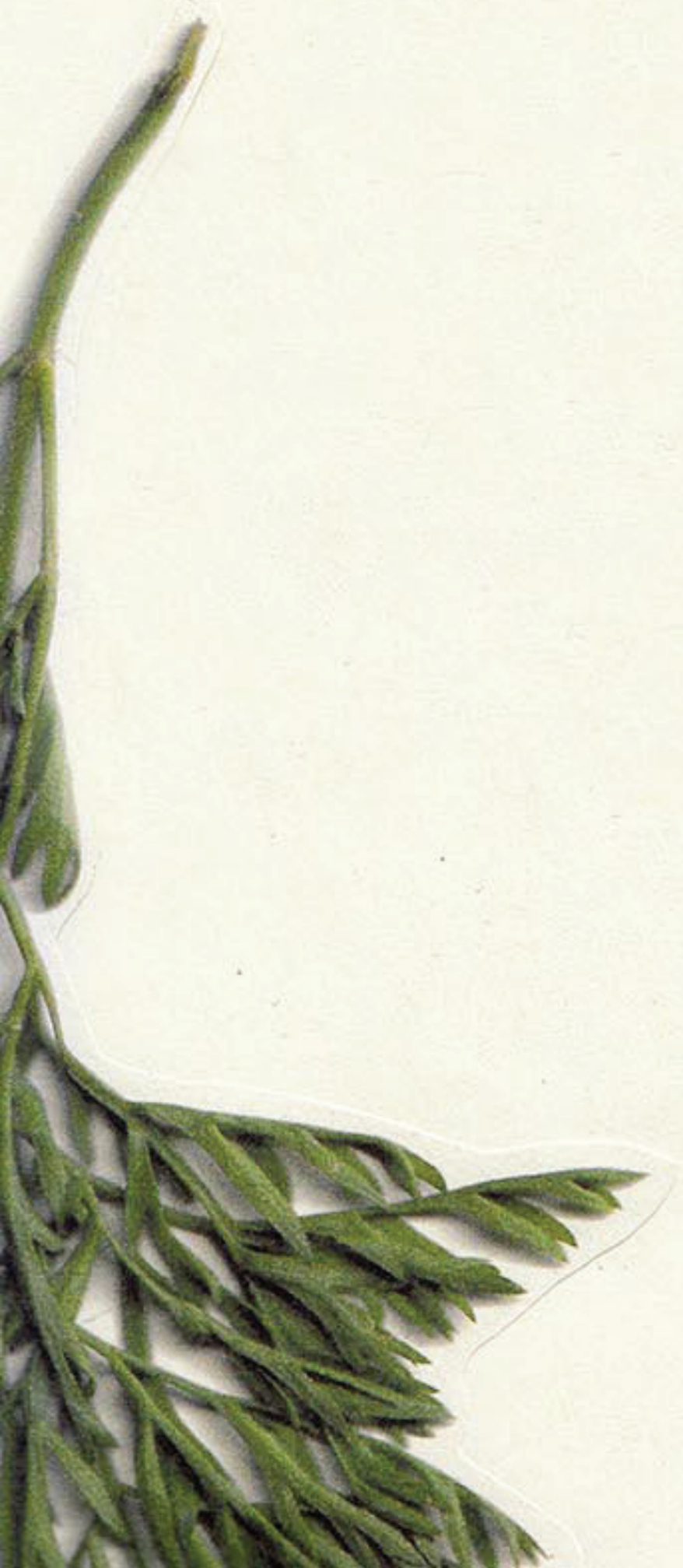

Remembering Robyn

Mowbray and Rosebank CAN

Robyn Montsumi was knitted into the fabric and hearts of various communities in Mowbray and surrounds. She was a service-user at the Sex Worker Education and Advocacy Taskforce (SWEAT) in Observatory and regularly attended the community suppers hosted by the Thursday Supper Club Collective at St Peter's Church in Mowbray. Robyn was a sex worker, a member of the LGBTIQ+ family, and part of the Mowbray homeless community. She was also an outspoken activist and challenged injustice and indignities of society's particularly marginalised.

During the most severe period of South Africa's lockdown, on 9 April, she was arrested by law enforcement officers of the Mowbray police station at a shop near the Mowbray bus terminus. Witnesses relayed that her arrest was violent and upsetting, and that drugs were found near her. She was taken to Mowbray police station and detained there. It is not clear what offence she was charged with.

Her partner checked up on her daily by shouting to her from outside the building. Robyn replied that she had not received food. The following day she told her partner that she was feeling ill. On Sunday, 12 April, there were no answers to her partner's calls to her. An ambulance later arrived at the police station, and after several enquiries Robyn's partner was informed that Robyn had died in the cells.

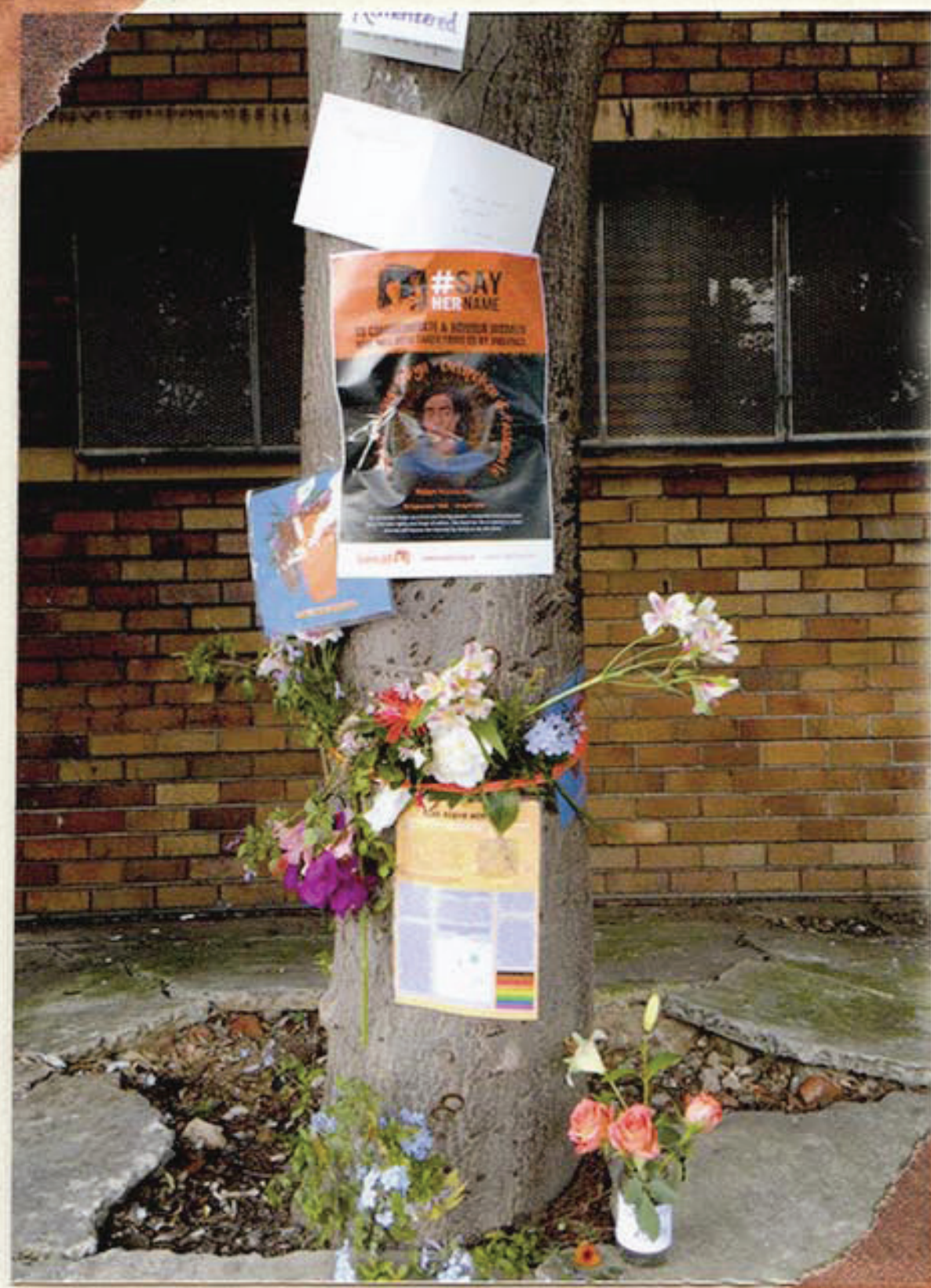

– Robyn Monstumi's memorial

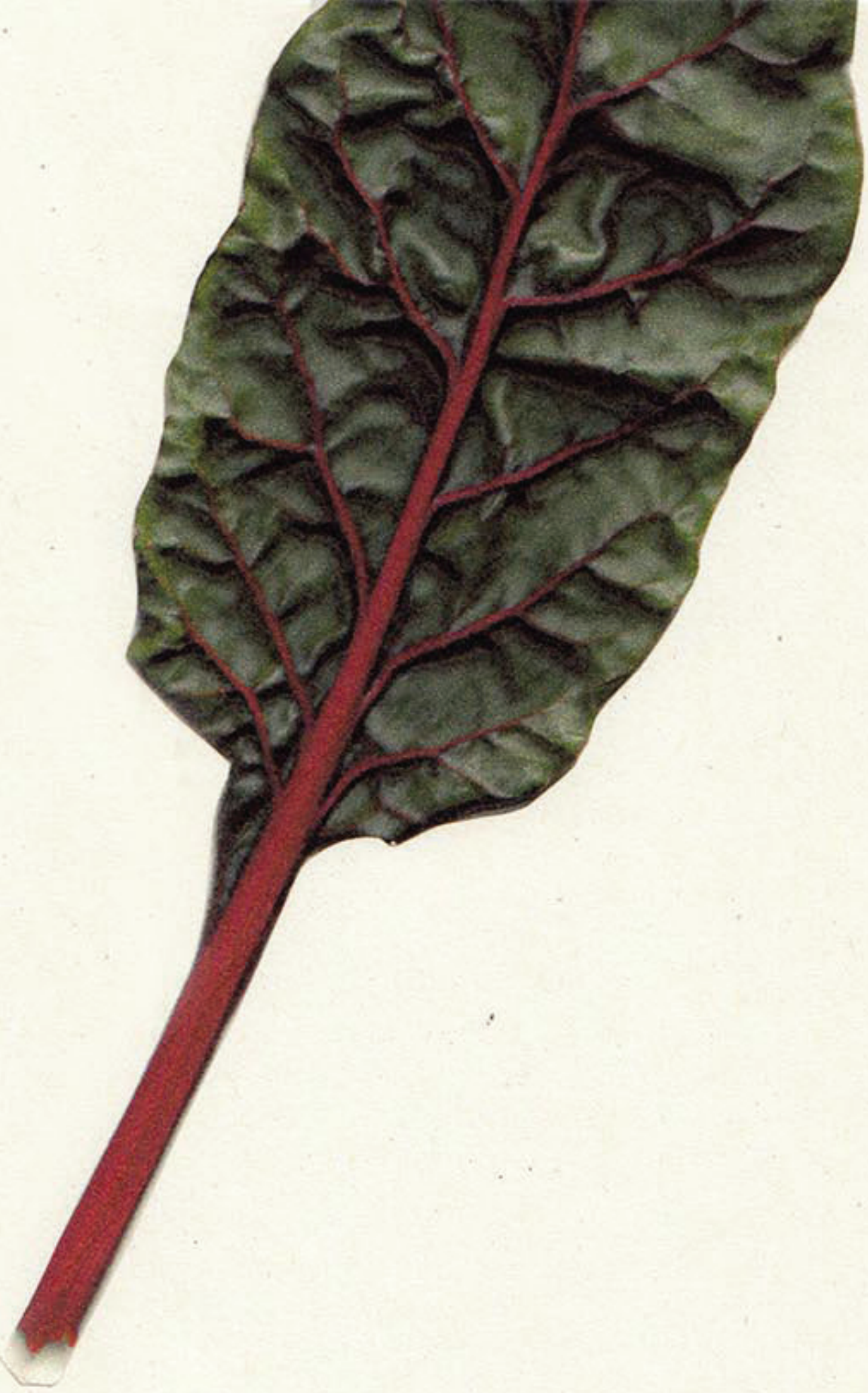

Police officers reported that it was a suicide by hanging, something her friends and family strongly dispute as they do not believe Robyn would take her own life.

Mowbray and Rosebank residents arranged a ceremony in her memory on the steps of St Peter's Church. A group of Robyn's friends, family and colleagues gathered with local organisations and community members. Speakers were invited to say a few words to celebrate Robyn's life, and everyone joined in a collective 9 minutes of silence — many kneeling — to show solidarity with the #BlackLivesMatter movement, and the many senseless and cruel deaths by law enforcement actions or neglect. The St Peter's church bell pealed loudly for the whole 9 minutes — the time period that an American police officer kneeled on the neck of George Floyd, that ultimately led to his death in May 2020. People walked to Mowbray police station thereafter and placed flowers and messages in tribute to Robyn to conclude the commemoration.

At the time of writing in November 2020, the Independent Police Investigative Directorate (IPID) investigation into Robyn's death had still not been concluded, and civil society complaints lodged with the Commission for Gender Equality and the South African Commission for Human Rights had not received adequate attention or action.

Civil society action continues to ensure justice for Robyn.

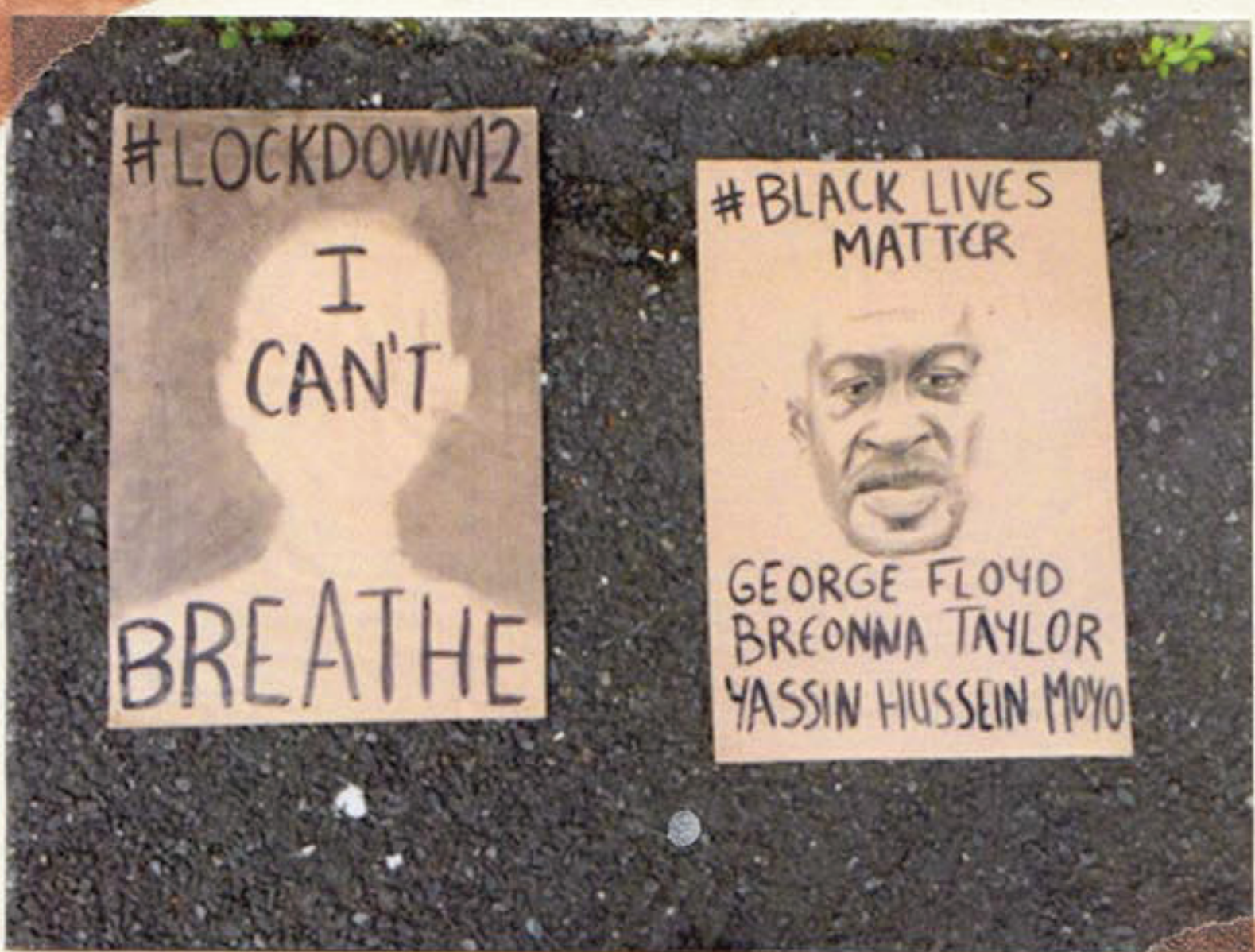

By Lloyd Rugara (Sisonke National Sex Worker Movement), Clinton Osbourn (Sex Worker Education and Advocacy Taskforce) and Marlise Richter

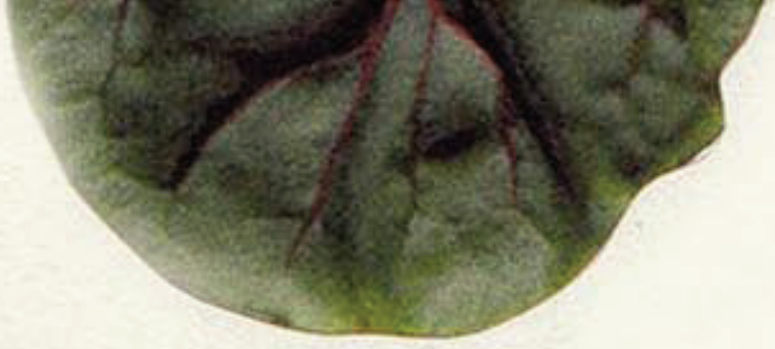

We all bring something to the table

Tafelsig CAN

The Tafelsig-Mitchells Plain CAN builds on strong histories, memories, and networks of community organising in the area. Joanie Fredericks, one of the women who started the CAN, is a well-known and widely respected community activist. Her vital insights into community self-organising come from her experiences of seeing people let down again and again by those who are mandated to provide support. When government and all the big non-profit organisations started to close their doors on the millions of people made hungry by lockdown, the Tafelsig CAN launched into gear. They started cooking food, growing food, and tackling the immense challenges of COVID-19 by looking inwards as a community. In this story Joanie reflects on the early days of the network, remembering how "right at the beginning, there was this frantic dash for the lesser-resourced CANs to team up with more resourced CANs". CAN pairings, as they came to be known, were a feature of the network that caught a lot of attention. But pairings were not necessarily as straightforward as they sound. Without real engagement with power dynamics and the politics of saviourism, pairings could easily have ended up in a model of charity that went against everything that Cape Town Together stands for – autonomous decision-making, self-organising, and respect for community intelligence.

Tafelsig CAN decided not to pair. In Joanie's words, "this dash to pair up was problematic because it means that people are falling into a trap of being in a vulnerable position and not doing things for themselves. There was this sense of powerlessness – unless a better-resourced CAN could help them, the work wouldn't be possible. This was problematic. For me, it is a time of crisis – as a community we have a responsibility during this time. We need to first look inwards to see what we can do with what we have, and how we can pull together before going out to ask for help."

This didn't mean that Tafelsig CAN was going all the way alone. "The connectedness to the broader network meant resources were readily available, and while I didn't want to partner with a wealthier CAN, I definitely made use of Cape Town Together to connect with individuals across different CANs. Doing that, the relationship building was absolutely invaluable, and continues to play that role. There were times we could reach out to individuals… and for me, I think the personal relationships play a much bigger role because there wasn't this heaviness of being looked after by a better resourced CAN, but there were people who wanted to assist."

Tafelsig CAN partnered with an organisation called Organic Earth Solutions to start the "One Household, One Food Garden" project. There are now food gardens popping up all over the neighbourhood, with people able to eat from their gardens, contribute to the kitchens and even sell some of their produce.

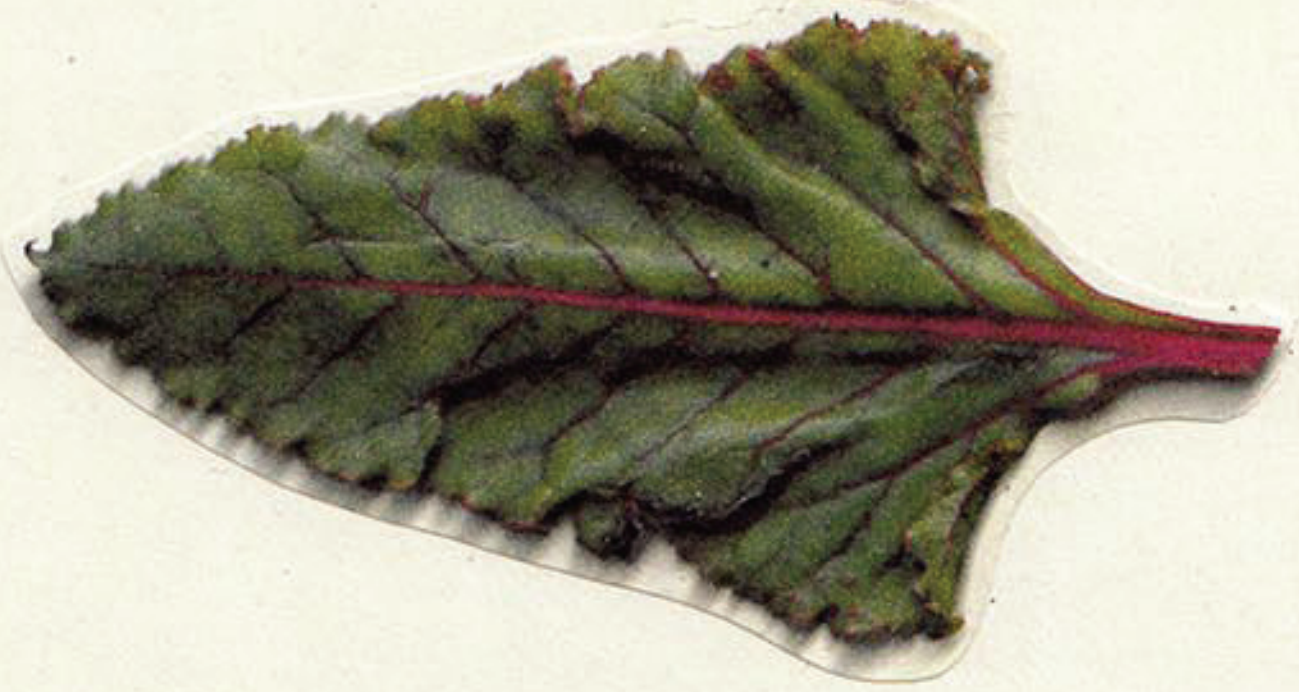

The CAN also received visits from Siya Kholisi and Mark Lottering – two well-known celebrities who heard about the Tafelsig CAN through Joanie's "Dear Mr President" videos, which aim to raise awareness about how the government has failed to fulfil even the basic promise that people would not go hungry during this crisis. Their visits to the neighbourhood played a huge role in how Tafelsig CAN viewed itself: "we have always been the step-daughter of Mitchells Plain – the place of murders, drug abuse and gangsterism. Having them here opened up the flood gates for people from other communities to come and visit the area and see, it isn't that bad. So, I think the fact that Mark and Siya and so many other people really paved the way also made us proud that in Tafelsig we can look after our visitors."

The network's strength lies in these relationships – warm, interpersonal, and built on mutual trust. The CANs have made remarkable contributions to their communities, bringing people together and generating excitement and willingness to participate in community projects. In the process, new forms of horizontal relating and being accountable to each other have emerged, and completely changed the ways in which things are done. But this kind of community power isn't easily recognised or accepted by those used to doing things according to the usual model. When it comes to accessing government support, both financial and otherwise, the CANs have struggled. The requirements to prove that community work deserves support lie not in the actual work being done but in having an NPO certificate, proof of registration as a Public Benefit Organisation from SARS, six months of recent bank statements, a copy of the founding documents and details of board members. This level of red tape and scrutiny is simply unable to recognise and support the good work of ordinary citizens who organise informally amongst themselves. It effectively means that those closest to the ground are automatically excluded from accessing state support. Meanwhile, politicians pat themselves on the back, taking credit for the work that community members have done for themselves with the little resources they have. This is why the Tafelsig CAN's strategy of self-reliance and organising one's own resources first has been fundamental:

"Tafelsig CAN has positioned itself as a group of people who want to be doing what they are doing. For us, it's clear, that for the unforeseeable future we will need to keep doing this work. Now is phase two, where we start re-claiming our dignity and our self-worth. The table is ours; we make it, and we are all responsible for bringing something – no matter how big or small – to share with those around it."

By Joanie Fredericks and Yaseen Johaar

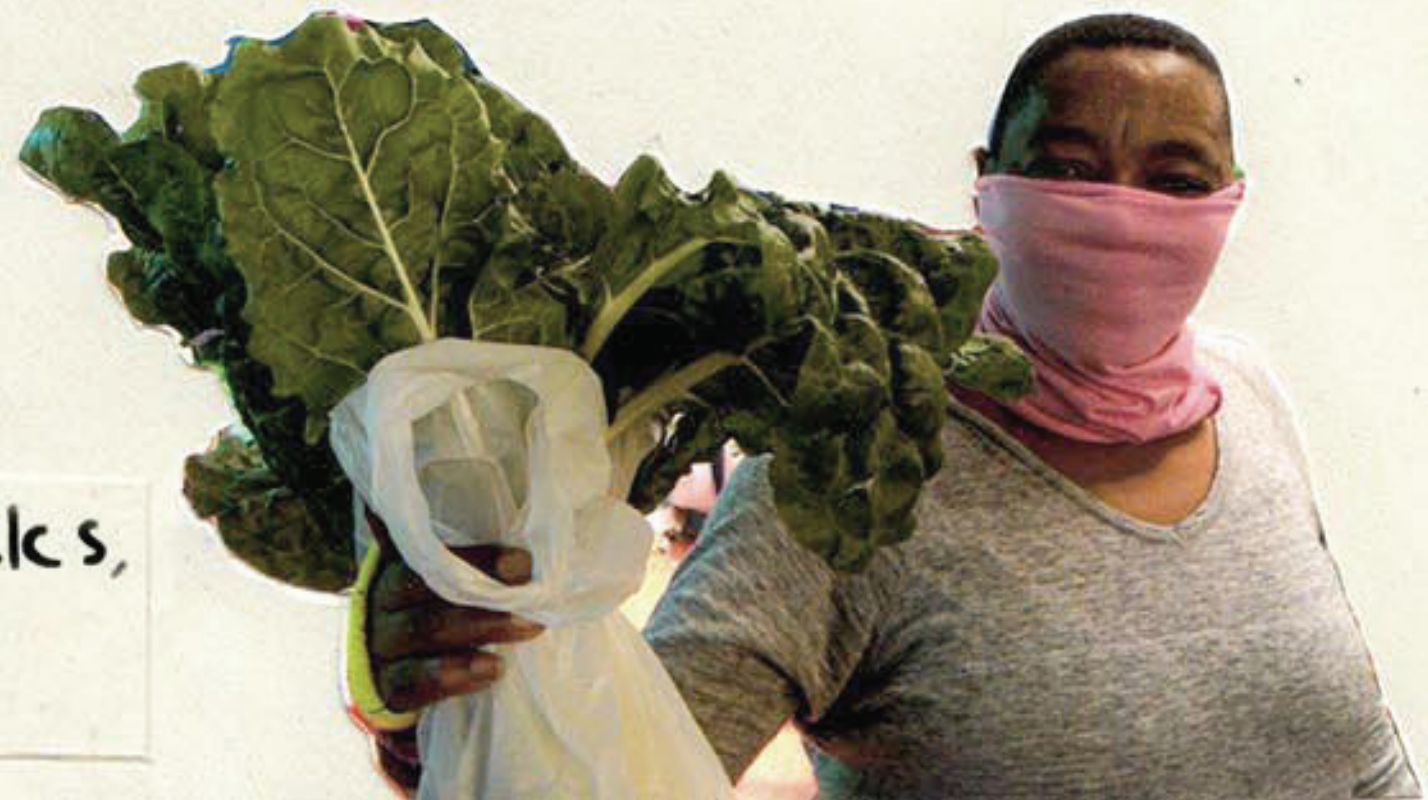

– Joanie Fredericks, Tafelsig CAN

Trusted trouble-makers in government

Connecting CANs

As the CAN network spread, many public servants chose to contribute to their neighbourhood CAN, or the broader network. Some also played an important role in bridging the divide between the CANs and formal state-led covid-19 response efforts – finding creative ways to respond to the crisis and its socio-economic consequences by sharing information (in both directions), linking to resources, and building trust.

However, many CAN-members experience the state as an uncaring, immovable, monolithic institution, promising so much and delivering so little. In attempting to reconcile this contradiction, the concept of 'Big G' and 'little g' quickly became an oft-used shorthand within the network. 'Big G' represents the GOVERNMENT – the institutions towards which we may well feel anger, frustration and disappointment. But within those institutions there are many 'little gs' – public servants working hard to share resources and create new possibilities for collaboration, in spite of the systemic constraints imposed on them from the institutions in which they work. This is a story based on a reflective conversation with three little gs, held over Zoom in early November 2020.

It's easy to see why people feel a sense of anger and frustration directed at Big G government. Even those of us who work in the system often fall into the trap of thinking of our service in the abstract, as a big, monolithic organisation. But for the people who need care, for a woman who gets hit by a car and picked up by one of our ambulances, the experience is actually of two people in a van. This means that the potential for human connection is always there, because government is just made up of people, many of whom are trying to help.

It is in those interactions between individuals that we find opportunities to build trust in the state. As employees of 'the system' who are interacting with CAN members, our role is to strive to build that trust. Often, it's just a matter of sharing information. The technical language we use as public servants is often very different to what is being used by the public. It was clear during the pandemic that people faced real barriers to finding their way into the system because of differences in language. For example, it's impossible for people to know where to turn to for help when our referral pathways depended on the sub-structure they live in, and people don't know what sub-structures they live in – they know what neighbourhood they live in. This discrepancy in language creates very significant barriers to accessing care.

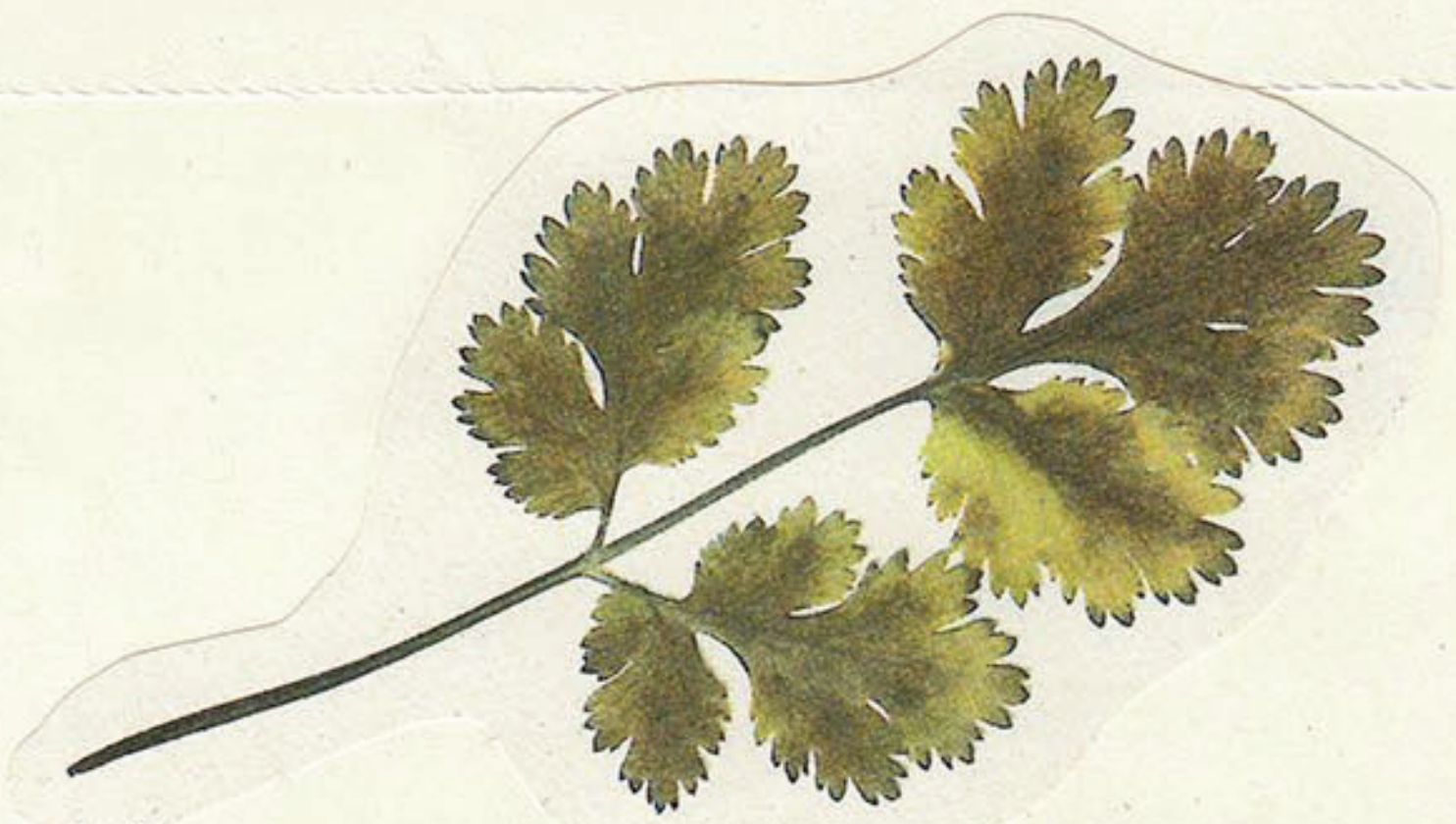

In this way, a big part of our role as supportive public servants is translation. Say you hear about someone who has a loved one in hospital and does not know how to get information about them. Instead of saying 'well that's not in my geographical area, I can't help', we are able to say, 'here is the phone number for the ward'. Often people would say they were glad to have a voice on the other side, knowing that we would follow-up and call back. These interactions are building trust all the time.

But while we are building this trust at the individual level, Big G is doing things that make it harder for people to trust the systems we work in. We can debate whether or not a hard lockdown was the right thing to do, and it's clearer in retrospect, but it seems like we made the mistake of pretending that we are England or America, where 95% of people live in a 4-walled, roofed house with ample food. We have to recognise that under lockdown, most people are just trying to survive, and neither Big G or little gs are experts in the survival strategies that work in informal settlements, or in rural areas. People are going to do what they need to do to protect themselves and their families from the most immediate threats, and we engineer a breakdown of trust if we don't acknowledge that, or if we try to criminalise acts of survival. We have to recognise that we don't have the resources to protect people from those threats. This requires us, as the little gs, to act with humility and to work in partnership with communities, with what we do have.

One thing we knew we were failing at was ensuring people had food when, for most, hunger was a much more pressing concern than Covid-19. Doing contact tracing, so many of the people we were working with were in home quarantine, and there were times when the DSD/ SASSA distribution structures were failing or delayed. It's frustrating when the people we should be serving are not receiving the food parcels that we promised them. It was a relief to find other contact tracers who were CAN-members or connected to CANs we knew were doing food deliveries. The CAN network was able to meet so many needs that we couldn't.

However, we have to recognise that there are tensions in this way of working, and that little gs working with the CANs faced threats and challenges from within the system. There is a Big G way of thinking that tells us that big problems, like a global pandemic need big solutions, and only Big G has the power and money to implement those kinds of solutions. In spite of this, there have been innumerable invisible and sometimes 'irregular' acts by little gs that you only see when you are looking for them, that have produced magic. But lots of people don't want to recognise them, because it hurts this Big G way of thinking. Public servants, or little gs, need to be granted the time, permission and resources to engage with community-led projects. This is a way of thinking about how Big G can do better, by creating institutional spaces for little gs to engage flexibly. The CANs have demonstrated what can be achieved when we work together, as CAN members and little gs, thinking small and acting local.

In some ways, then, the success of the CANs highlights the shortcomings of the Big G response. For highlighting the short-comings of the Big G response, the network gets represented by some as a political threat. Little gs and the community-led initiatives they participate in are constrained rather than supported by systems governed by politics and bureaucracy. Community engagement becomes a pathway to political point-scoring, rather than problem-solving. An effective response is deemed so by the auditors and the politicians, not the people. And our biggest resource – the little gs, CAN-doers and ordinary folk doing the work of caring for those around them are ignored or, worse still, suppressed, by an uncaring and immovable monolith.

By Leanne Brady, David Pienaar, Manya van Ryneveld, Suhair Solomon, Shaheem de Vries, and Eleanor Whyle

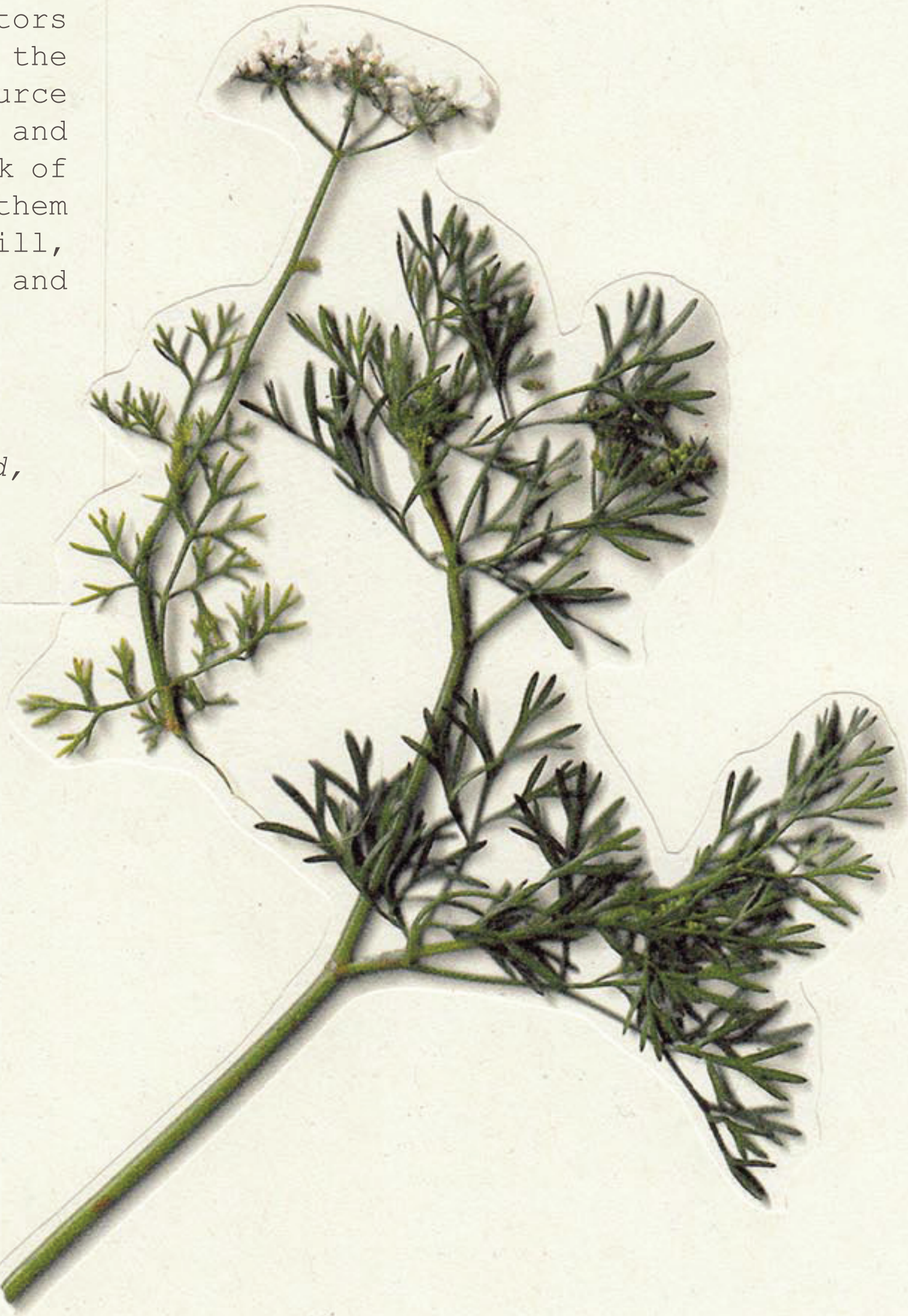

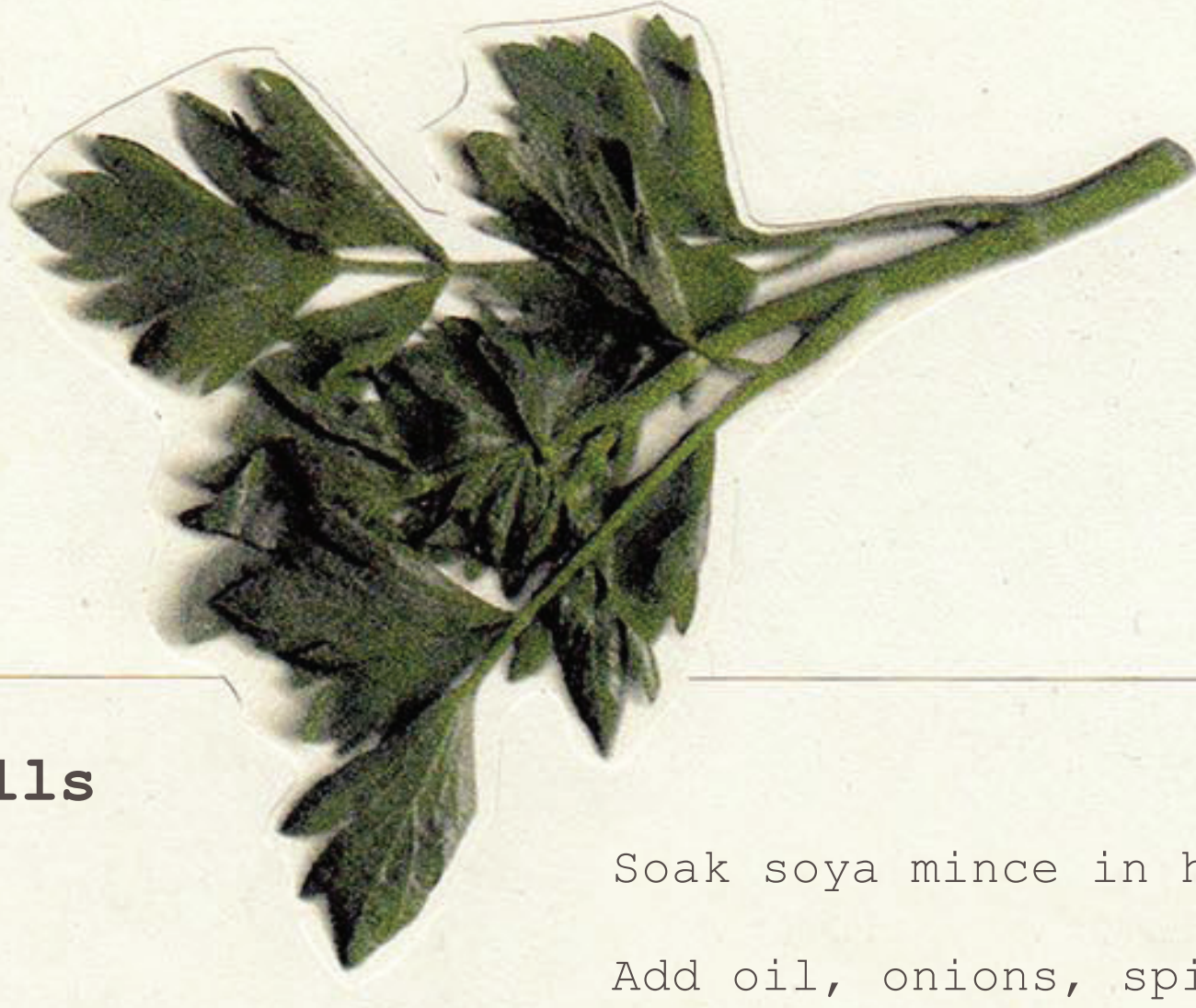

Gogo's meatballs

Gugulethu CAN

These meatballs are served in Gogo's community kitchen, supported by the Gugulethu CAN.

This recipe feeds 300 people.

For the Samp

- 40kgs Samp
- 80 litres water
- 20 tbsp Aromat
- 10 tbsp salt
- 5 tbsp pepper
- 500g butter

Wash and rinse the samp

Bring to the boil 80 litres of water in 2 pots (40 litres in each pot)

Add 20 kgs of samp to each pot

Cook, stirring intermittently, for 2.5 hours, keep the lid on the pot

Add half of the Aromat, salt and pepper to dry ingredients in each pot

Take off the heat, and allow to sit

For the meatballs

- 6kgs mincemeat
- 5 litres soya mince
- 1 litre hot water
- 6 onions, grated
- 125ml barbeque spice
- 50ml pepper
- 5 litres cooking oil

Soak soya mince in hot water

Add oil, onions, spice and pepper

Roll mixture into small-medium sized balls

Deep fry in cooking oil until brown

For the gravy sauce

- 5kg onions, finely chopped
- 500g soup powder
- 1kg tomato-flavour soya mix powder
- 100g curry powder
- 100g beef stock
- 2 bunches celery, including leaves, chopped
- 14kgs potato, chopped and cooked

Fry the onions

Add curry powder

In a separate bowl, combine soup powder, tomato-soya powder, and beef stock in 5 litres of water

Add powder mix to onions, stirring intermittently

Add celery and cook well

Add potato and allow to simmer

When the meatballs are done, add them to the sauce

By Maria Nombulelo Dlokolo, aka Gogo

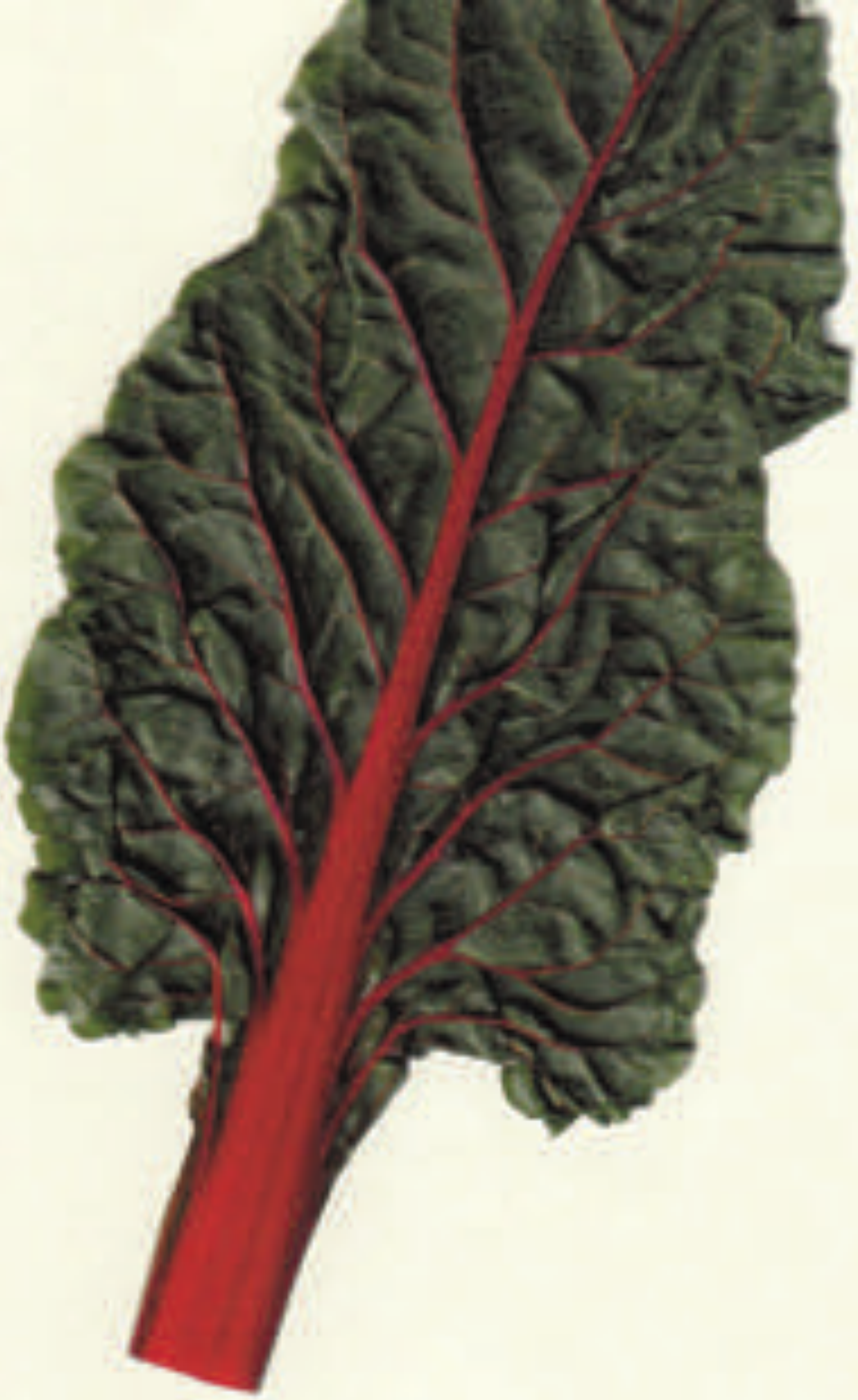

- Maria Nombulelo Dlokolo
Gugulethu CAN

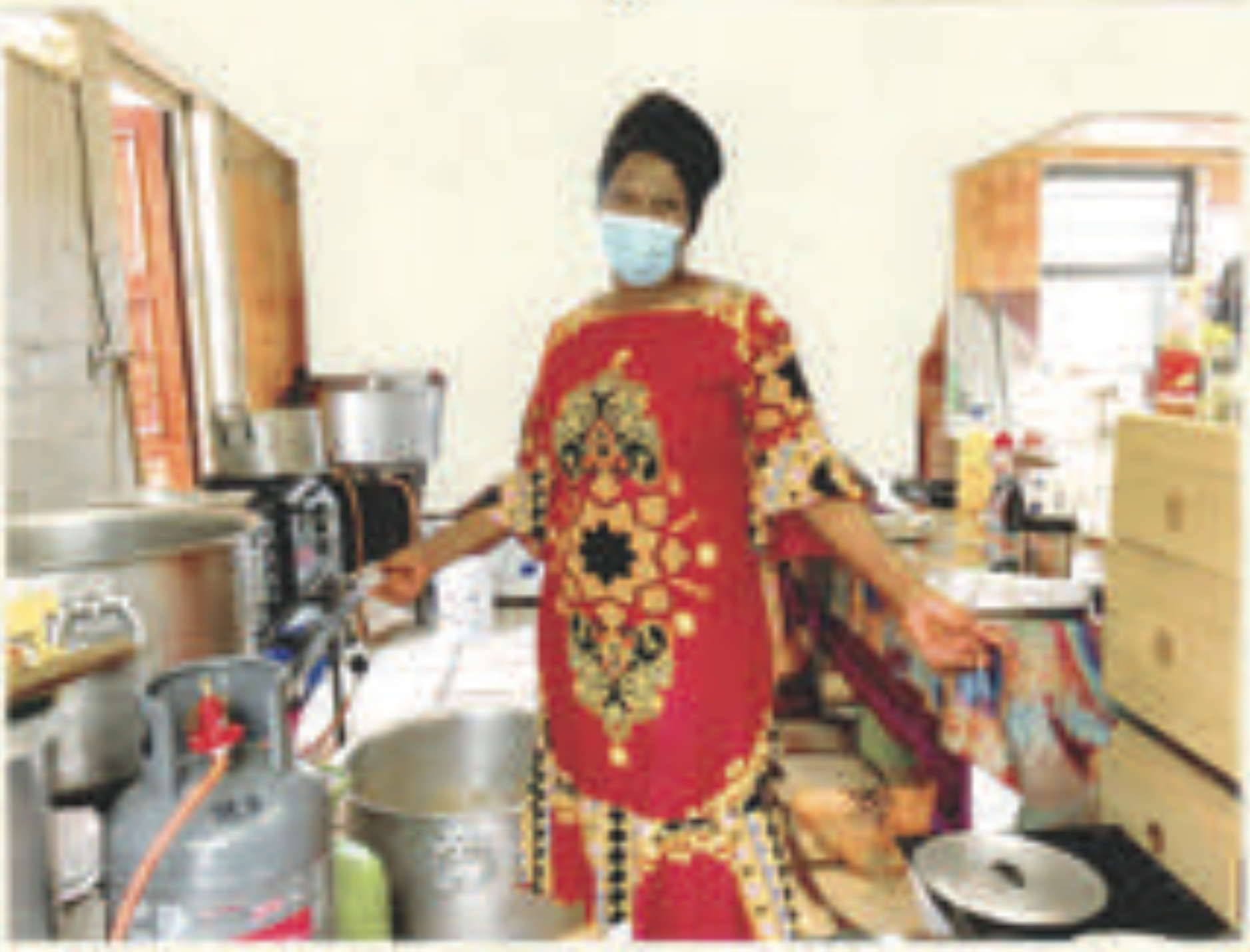

On lifelines, legacy and love

Gugulethu CAN

"I love, love, love what I am doing!" - Gogo

It strikes me as I walk in through the front gate of Gogo's home how calm it is in the front yard. She's sitting with one of her volunteer stalwarts Sis'Lindi, and they're peeling and chopping potatoes and carrots with expert precision. They have just finished the morning breakfast serving from the kitchen and have moved smoothly into preparing the lunch meal, her famous meatballs recipe.

They are not rushed, in fact that is something I notice about the entire visit; nothing is rushed, but everything is purposeful. Every moment appears seamless, from who steps up to check pots, sharing instructions on timing of dishes, negotiating the seemingly endless number of passers-by who check in to greet, express gratitude or enquire about various needs of support or aid. It's a smooth flexible operation, bending to the varied unpredictable needs, with a clear goal in mind: feed people with dignity and always with a side portion of love.

It is clear that this kitchen like countless others that have popped up during this time, are beyond just a nutritional life-line to the community it serves, but a site of reprieve and relief in a time where so many are forgotten or alone and in desperate need of resources and connection.

Gogo shares that her journey to cooking is multi-generational and rooted in community. Her earliest memories are centred around her mother, community, and food.

She recalls her mother's mixed heritage partnership, and how that was a source of inspiration and diversity in her cooking (ingredients, recipes and meals) and opportunity to build relationships across difference. Her earlier memories are of her mother cooking for her school events, her father helping people within their community and always, always having the best meals on the table at home!

Her mother's favourite dish always included fish. I can almost hear her salivating describing the fried, steamed or cooked varieties - though between us she confesses that meat is her number one favourite ingredient!

Gogo's life has been a series of expansions and contractions over these years. Her work went from domestic work and cooking, owning a restaurant to moving into small and large catering business opportunities, and closing it all down in crisis, only to begin again as a community kitchen.

She has worked with very little other than good intentions during these months, and those have clearly reaped extraordinary rewards.

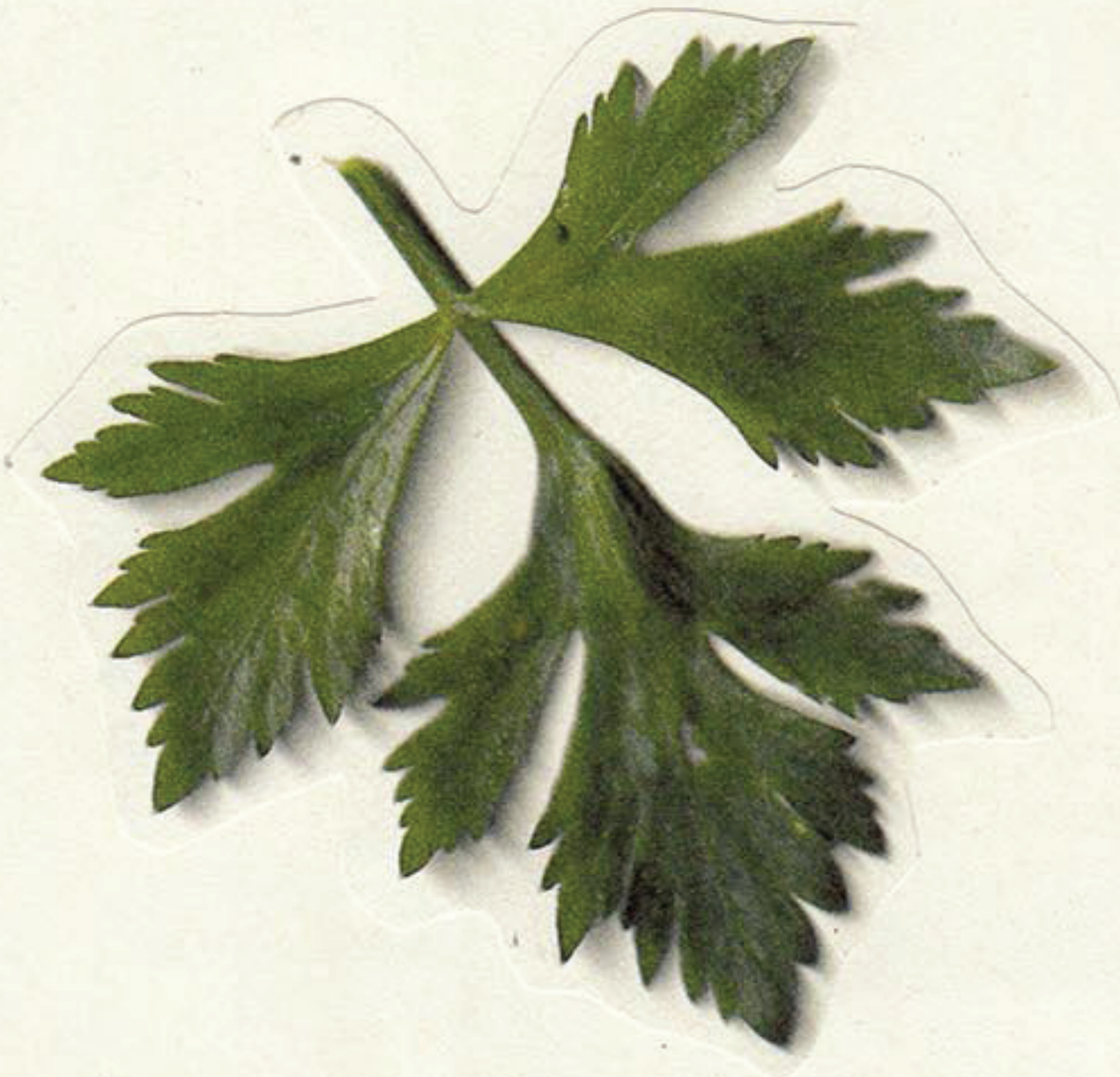

The proactive action to meet people's basic food needs including feeding her own family, collaborate and share information and resources freely inspired the opening of 36 community kitchens across Gugulethu, 15 of which are still operational today. She shares her donations and resources freely, building trust as she goes. It is unsurprising that Gogo's daughter Pamela is a community activist, championing the Gugulethu CAN, and her continued Organising for Work commitments throughout all levels of lockdown.

Gogo's Kitchen is a lifeline to young and old. It is a site of generosity and a safe place. One of the street champions who delivers meals to the elderly comes to report he's found temporary employment, and the kitchen erupts in joy at the news.

The kitchen is a by-product of a legacy of love for community. It is also a microcosm within a broader community and society - demonstrating what is possible. I cooked one meal and left simultaneously exhausted and filled with awe and gratitude. Community kitchens deserve resources and support. Gogo's Kitchen can teach so many of us a thing or three about life, radical generosity, and community solidarity. And those meatballs…delicious!

By Maria Nombulelo Dlokolo, aka Gogo, and Suhair Solomon

Lamb curry

Salt River CAN

- 1kg lamb pieces
- 10 potatoes, peeled and cubed
- 3 onions, peeled and finely chopped
- 3tsp oil
- 3 cardamom pods
- 3 cinnamon sticks
- 3tsp garlic, finely chopped
- 1tsp ginger, finely chopped
- 1 tsp turmeric
- 3 tsp roasted marsala
- 1 tsp chilli powder
- 3 tbsp biryani masala
- 9 curry leaves
- Salt to taste
- Fresh coriander

In a large pot, heat oil on a medium heat

Add onions, salt, curry leaves, and cinnamon pods

Fry until golden brown

Add meat, garlic and ginger

Braise for 5-10 minutes

Add 1 cup hot water, and cook for 30-40 minutes on medium heat, stirring occasionally

Add all spices. Stir and braise for 10 minutes

Add potatoes and 2 cups of water

Cook until potatoes are soft

Garnish with fresh coriander

Serve with roti or white rice

Delicious!

By Zainap Salie

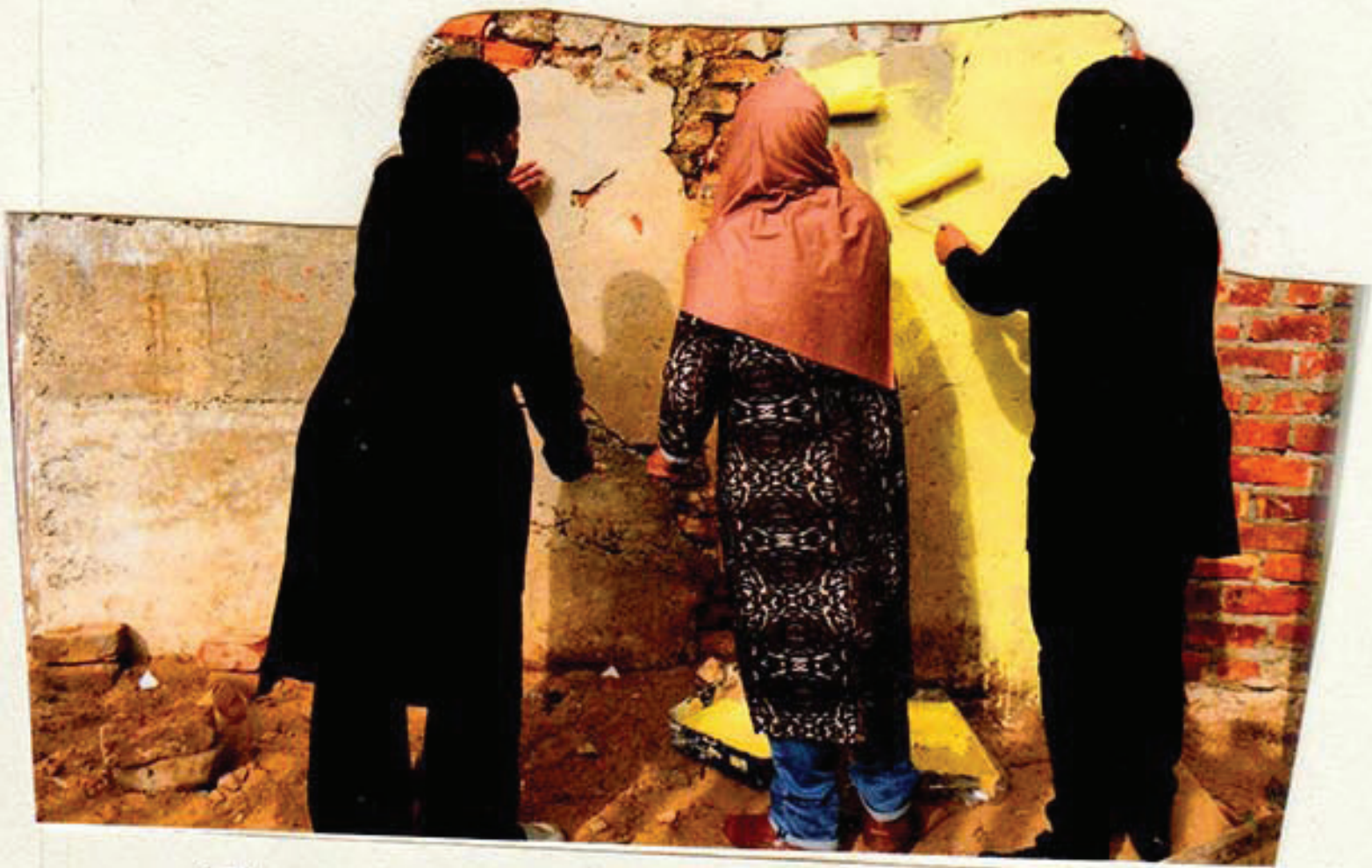

- Faeeza Alexander, Amina Ryland, Zainap Salie

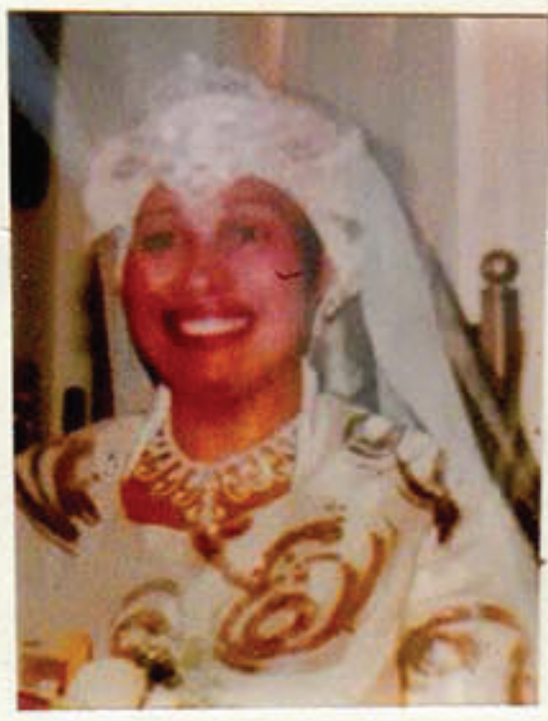

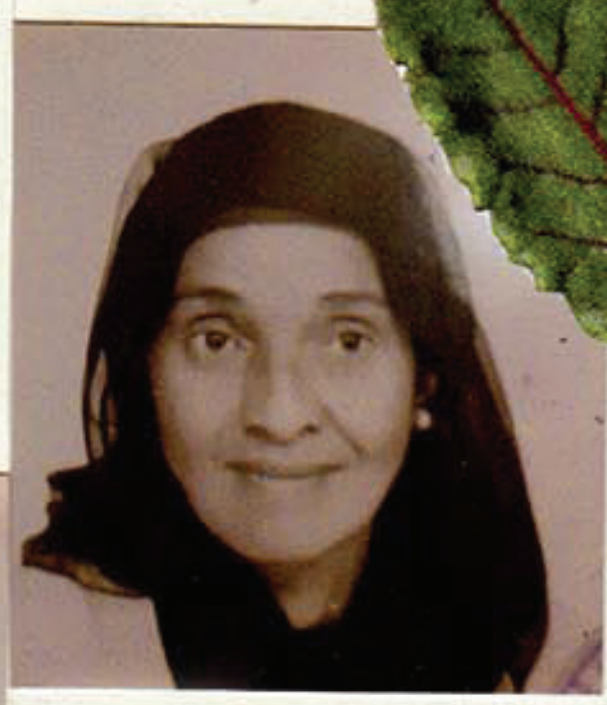

Rugaya Dramat

Torched car becomes beacon of hope

Mini Meltdown

On March 23rd 2020, Peter and Lesley (Wagenaar) began sharing food with the homeless folks in their Mouille Point neighbourhood. A few local residents strongly objected, and on May 6th, Lesley's Mini Cooper was burnt to the ground. This sparked the #minimeltdown movement, which quickly grew into a powerful network of people working with homeless folks in the area – including Tasneem Hoosain, a full time professional social auxiliary worker.

When lockdown hit, we knew that all the sources of food that homeless folks usually rely on would disappear. So, we started making food, and sharing it from our car.

The relationship with a few disgruntled neighbours was antagonistic from the beginning. They were unhappy with us handing out food in 'their neighbourhood.' Their complaints resulted in Law Enforcement and SAPS officers visiting us daily to deter us. We were reported by neighbours for supposedly breaking lockdown rules, even though we had a permit, and even got strip-searched in the Mini because someone complained to the police that we were dealing drugs.

Then in the early hours of the morning of May 6th, our car was set on fire. It was sad to think that someone would stoop to that level of violence as a final deterrent. But if you are homeless and starving then the fact that a car has burnt is irrelevant, because you are still hungry and in need of sustenance.

The burning of the car drew a lot of attention, and many neighbours came out in support of our project. People who had been on the fence were suddenly confronted by a very alarming visual representation of what was actually happening. It highlighted not only the inequality, but also how uncaring some people can be towards those who have less than them.

The overwhelming support and generosity since then, from the Atlantic Seaboard Community and others in the greater Cape Town area meant it was possible to employ Tasneem - a social auxiliary worker - full-time, to offer much needed services to homeless people living in the Seaboard area.

So often we talk about the 'wicked problem' of homelessness, as if there is nothing we can do. But when you stop for a moment and just think about the person standing in front of you and start to engage with them, then you begin to understand some of the layers of circumstance that have brought this person to this point. Instead of trying to fix the bigger problem, you approach it like an onion made up of many layers. You begin to unpack each layer until you reach the core. Dealing with the immediate needs of the person standing in front of you is where the healing can begin.

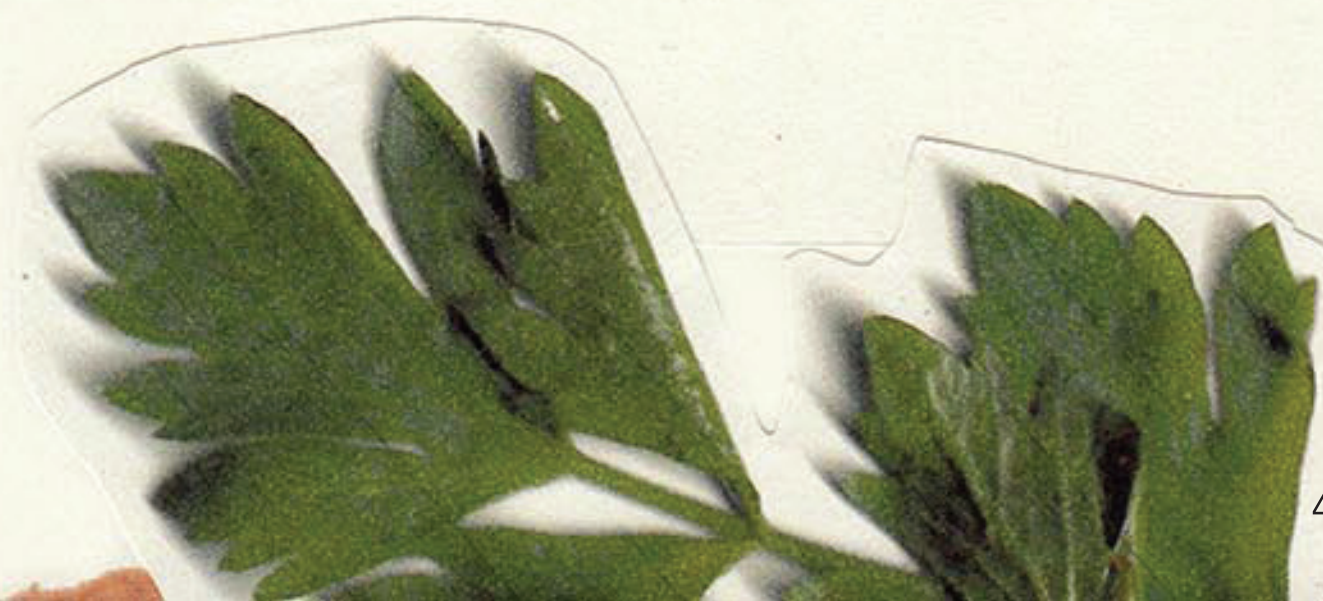

People might say that we are encouraging homelessness by sharing food with people living on the streets. But what they don't realise is that every hotdog is the start of something more – it's a communication tool. It's about building a relationship and developing trust over time. Meeting the most immediate need, whether it's hunger or something else, is the start of that relationship.

One woman we worked with went from living on the streets in an abusive relationship, to living at home with her family. We met her through a takkie drive. She wears a size 4, and received a brand new pair of pink and white takkies. Through that interaction we started to get to know each other, and it turned out that although she was homeless, she had a husband who was taking very good care of her kids back home in Mitchells Plain. She had left home to be with her boyfriend, who turned abusive, and they were living on the streets and going hungry.

We started talking through her options and ended up talking about love. We spoke about what love looks like, and how more often than romance and drama, it looks like acts of service: making porridge for your kids in the morning, or washing school clothes in the afternoons. After a number of conversations like this the woman asked Tasneem to help her get off drugs and to go back to her family. The good news is that she is no longer living on the streets and is back home being a wonderful mother to her family. This all started with a pair of pink and white takkies!

The story of the Mini Meltdown is a vivid demonstration of the power of ordinary people engaging in everyday acts of love and care. In the grander scheme of things, handing out meals is not a big thing, but by sharing food and meeting that immediate need, you open up a world of possibilities.

Over the last 8 months, we have become more than just a collaboration between Mini Meltdown, Souper Troopers and the Seaboard-Gugulethu CAN partnership. Initially, different groups were each 'just helping out' in their own little corners. Now it has grown into a massive network, spanning from Gugulethu to Sea Point. So, if a homeless client needs something that we can't provide, we can reach out, and nine times out of ten we get what we need for them. It's phenomenal!

It's a support structure for all of us doing the work, as well. If someone is feeling a bit overwhelmed or helpless, there is a whole network of people we can call, who will understand and assist.

We all need a little bit of support from time to time, and one of the biggest challenges we face as a society is that this support is so rarely available. Imagine a world in which every neighbourhood had a social worker and a feeding scheme to offer support to people struggling with homelessness, bereavement, mental illness, substance dependence or financial difficulties.

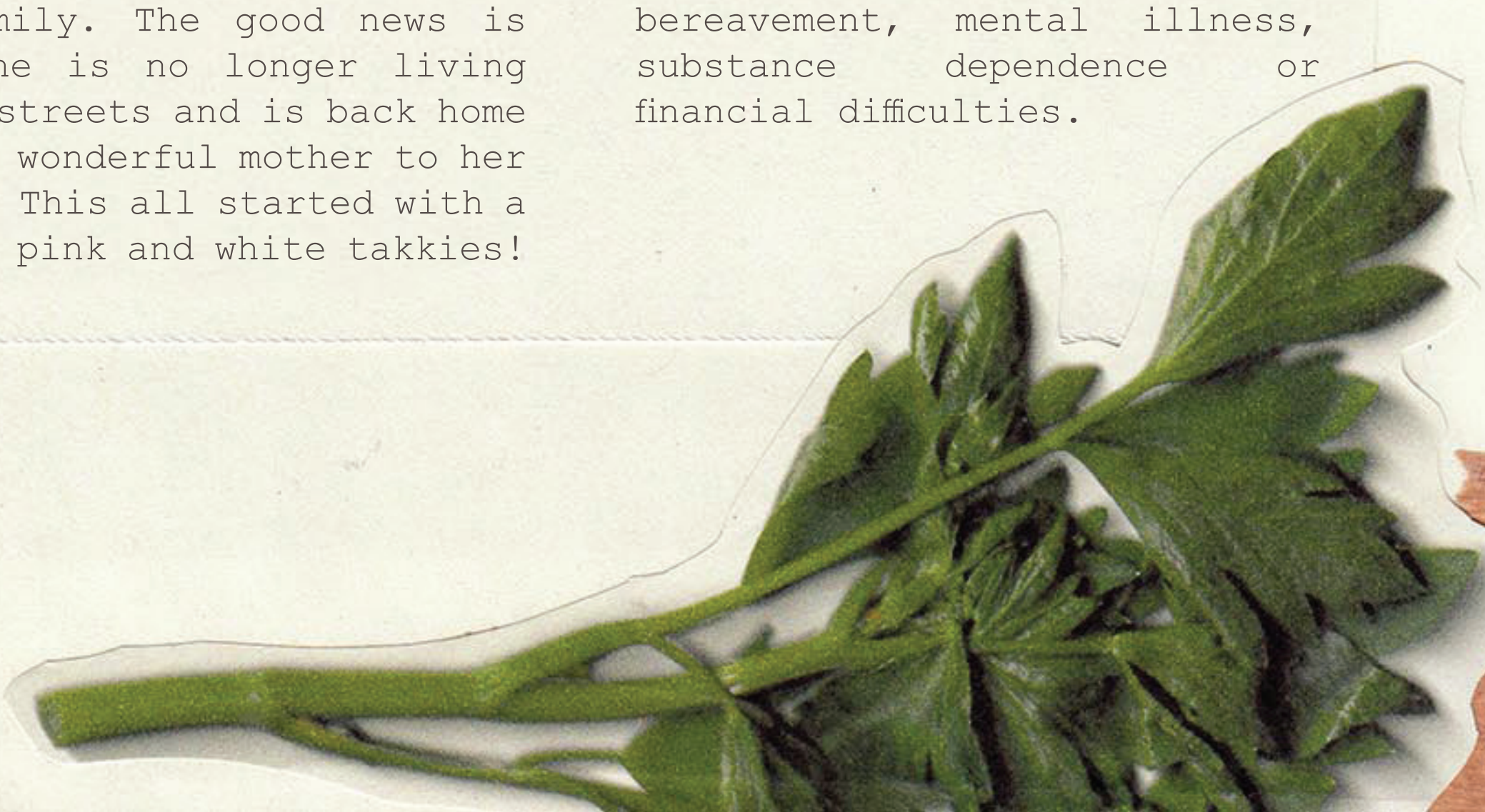

This is a new community, and a new society – one of love, dignity and respect – but it starts with a meal served from a Mini Cooper, a conversation about love and motherhood, or a pair of pink and white takkies.

By Tasneem Hoosain and Peter Wagenaar

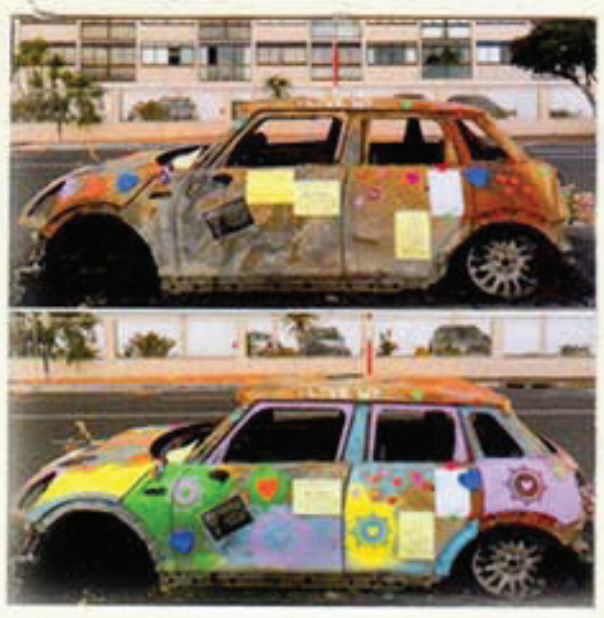

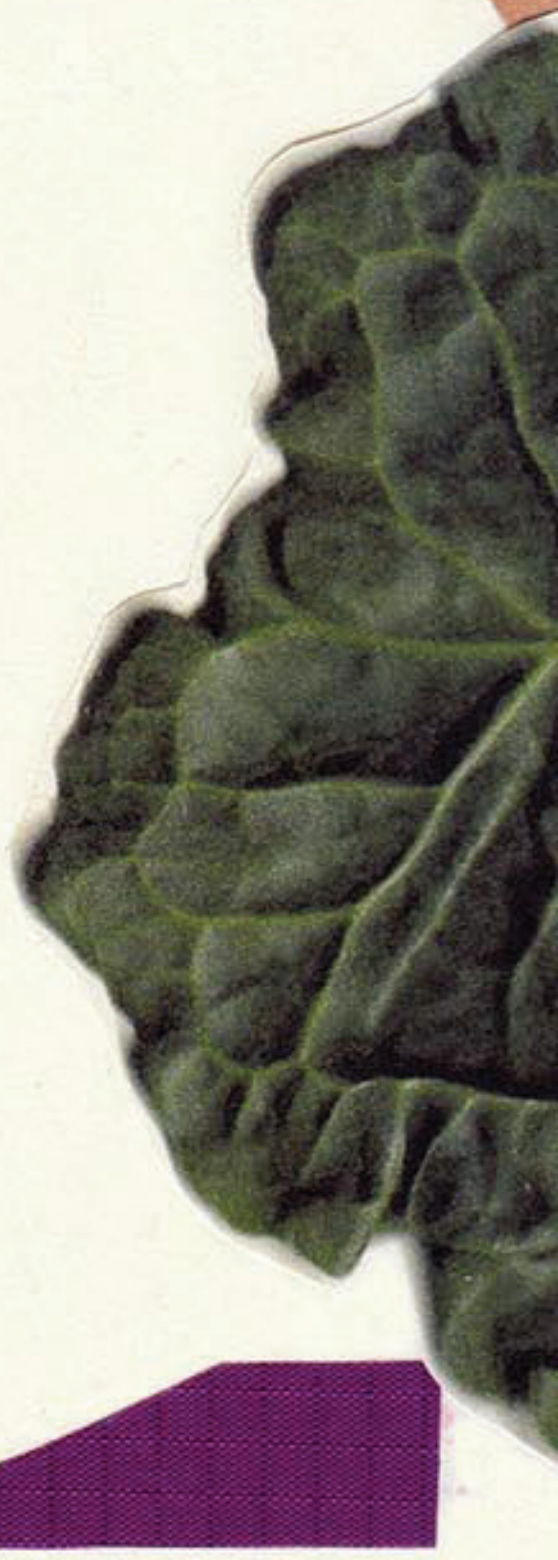

Stir Fry

Heideveld CAN

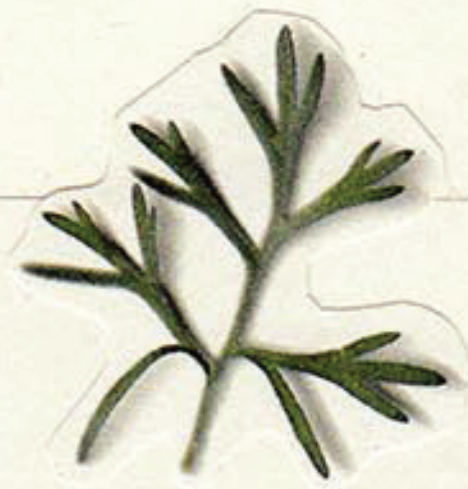

That is our stir fry recipe for all our extended families.

- 10kg chicken breast
- 15 cabbages
- 5 red peppers
- 5 green peppers
- 5 yellow green pepper
- 10 large onions
- 3kg carrots
- 10 packets spaghetti
- Fish oil
- Salt
- Pepper
- Beef stock cubes
- Garlic powder
- Potjie kos spice
- Cloves
- Stick cinnamon
- Stars
- All spice
- Cumin
- Chili powder
- Paprika
- Mixed herbs

Bring a pot of water to the boil with salt and fish oil. Add the spaghetti. Stir occasionally so the spaghetti does not stick together. Don't let it get too soft. When the spaghetti is done, drain the water. Keep some water aside to use later

Cut all veggies and chicken into thin strips

In hot oil lekker smoor (fry) your onions, until they are almost brown. Add the spices while the onions are browning

Break up 10 beef stock cubes, and add to the onions and spices

Add the chicken strips to the onions and spices, and smoor until nicely brown and soft (about 5 minutes)

Add all the cabbage strips cook until nicely soft

Add the carrots strips.

When the carrots are soft, add in all the peppers and cook until just a bit soft but still a bit crunchy

Then add the spaghetti in portions, mixing well after each addition. Once all the spaghetti is in, add more garlic powder, pepper, more beef stock and the left-over water from the spaghetti. Simmer for about 10 minutes on a very low temperature stirring occasionally

By Vanessa Nelson

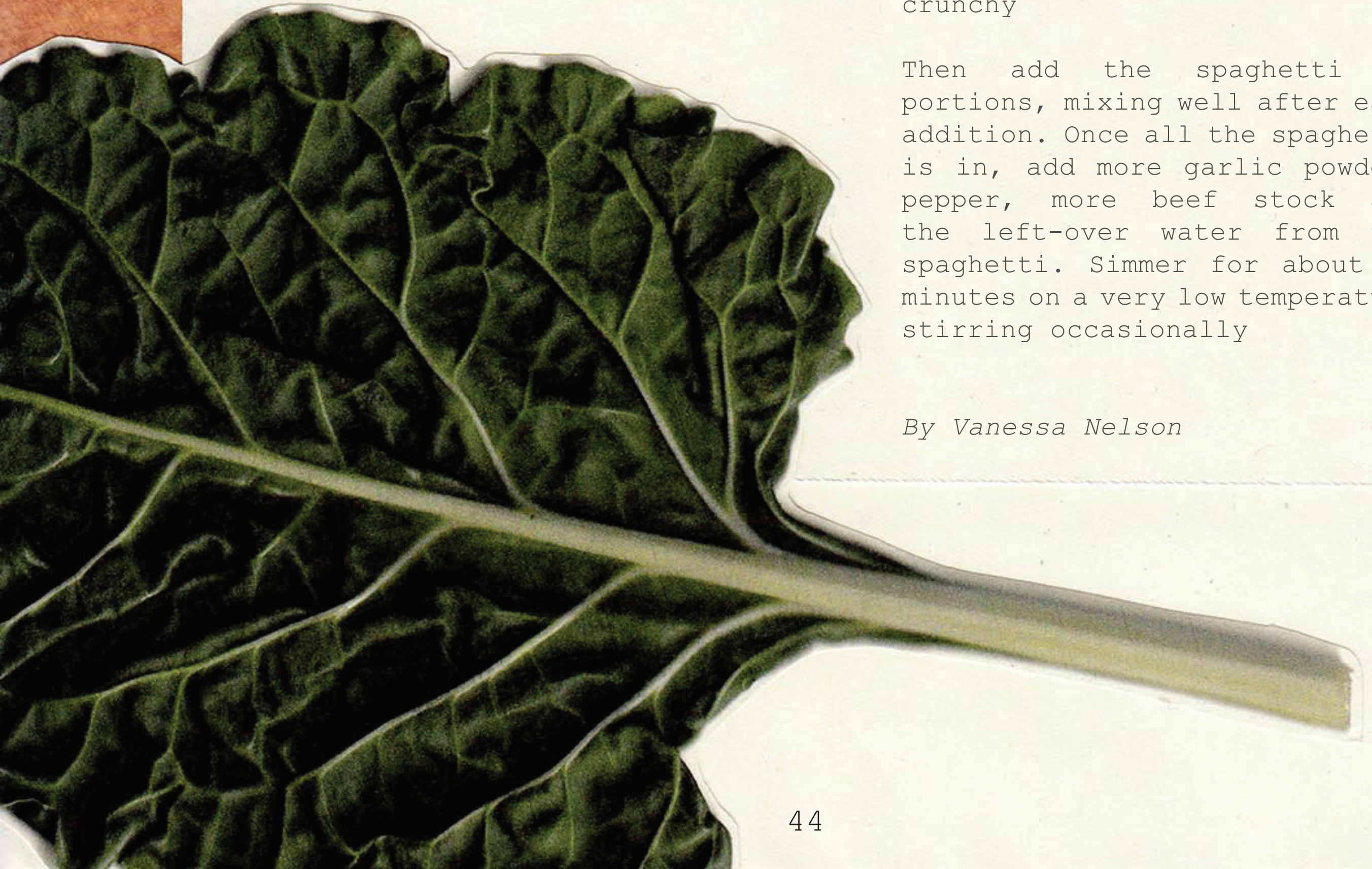

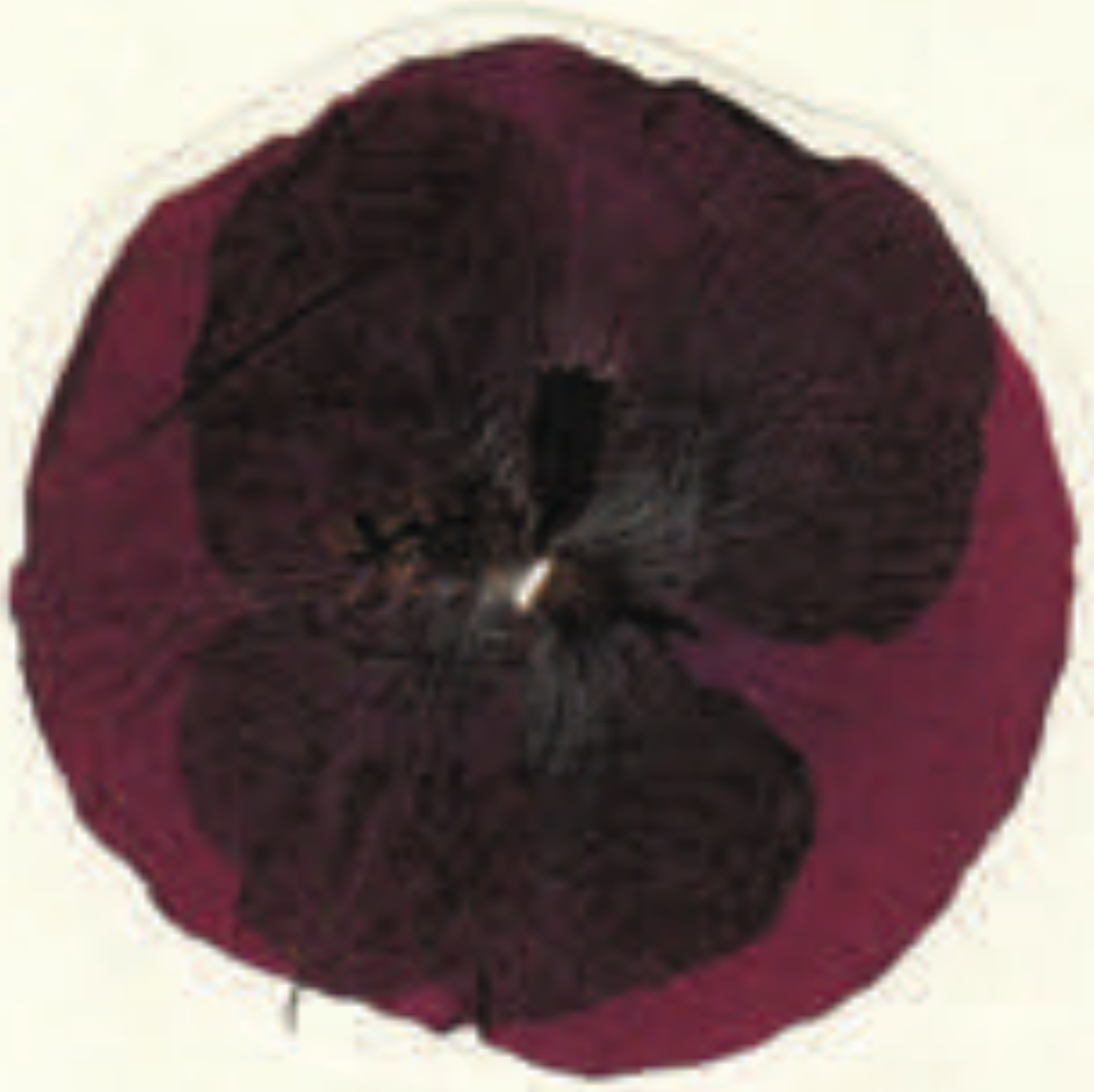

Lasagne with Soya Mince and Butternut

Brackenfell-Kraaifontein CAN

Imagine a delicious vegetarian lasagne that even meat-lovers would enjoy. In 1 hour you can make a lasagne big enough to feed 50 people.

- 3 large butternuts
- 3kg soya mince
- 6l milk
- 2 cups flour
- 500g cheese
- 4 cups stock
- 6 large onions
- 3kg pasta/lasagne sheets
- ½ cup oil
- 500g butter

Peel and chop butternut and either boil till soft or cook in the oven at 180°C for 2 hours until soft

Heat oil in a 25-litre pot

Make stock, and add to the pot with the oil

Add soya mince and cook for 40 minutes on medium heat

In a separate pot, fry the onions in oil (you can also just cook the onions in the oven with the butternut)

Add cooked butternut and onion to the soya mince

Add pasta to the soya mince and cook until the pasta is soft

In a separate 15-litre pot, melt a block of butter

Add ½ flour and mix together. Make sure to get rid of all the lumps

Keep adding flour until the mixture becomes a thick paste

On a low heat, add the milk bit-by-bit. Each time you add some milk, stir until the mixture is smooth and there are no lumps. Keep adding until all the milk is mixed in

Add salt and pepper to taste

Keep stirring until the mixture begins to thicken. Keep stirring continuously. Be careful not to let it burn. Keep the heat low, keep stirring and be patient

When the mixture has thickened, add grated cheese and mix until the cheese is melted

In a large servicing dish, add a layer of soya mince and pasta. Enough to cover the bottom of the serving dish

Pour over a layer of white sauce

Add another layer of soya mince on top, and another layer of white sauce

Keep layering, ending off with a layer of white sauce on top

Sprinkle cheese on the top

Serve and enjoy

P.S. An alternative is to bake butternut halves in the oven, then fill up the butternut halves with soya and top with white sauce.
Yum!

By Lynn Hendricks

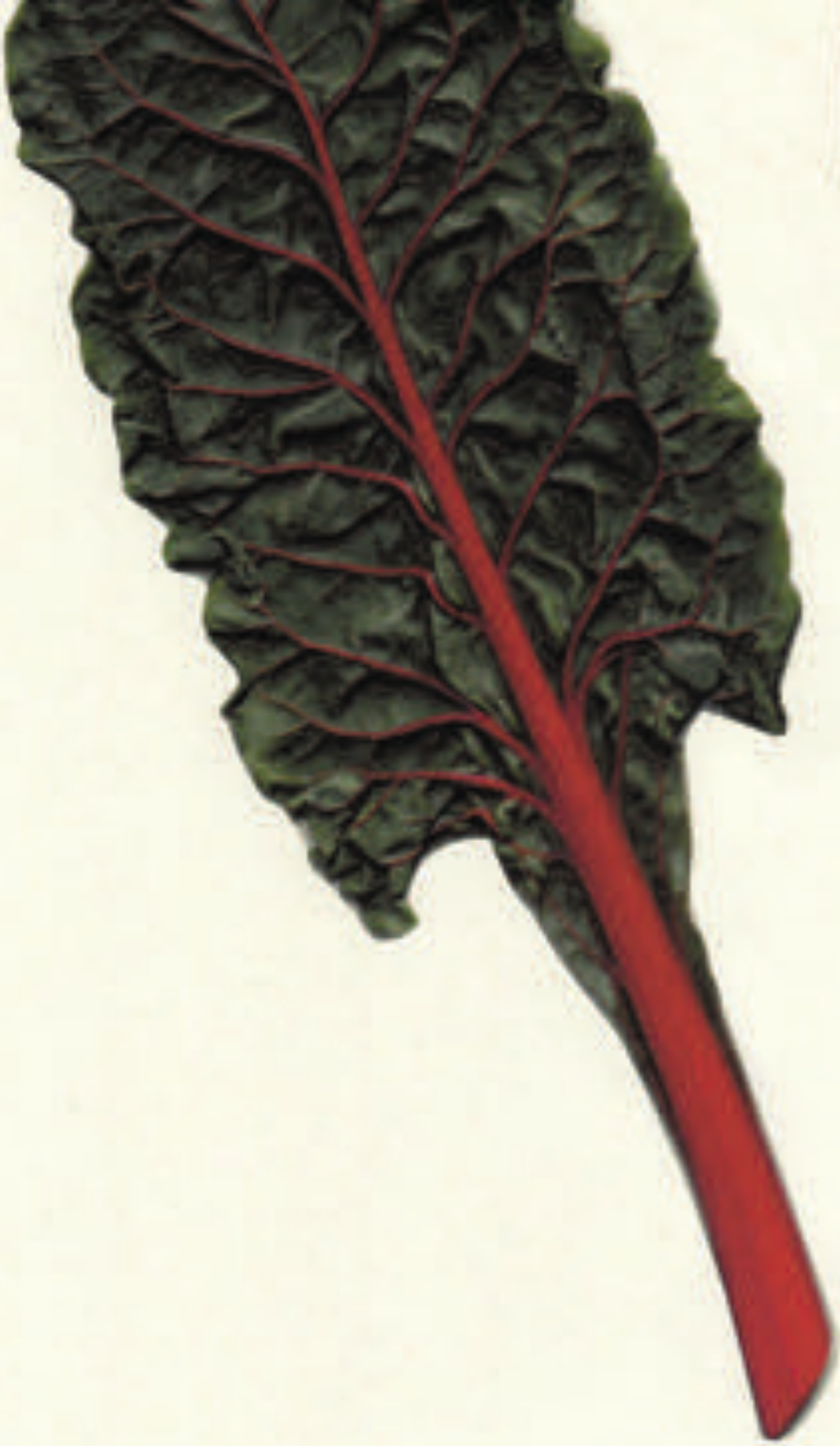

For some of us the normal
was not normal enough to
go back to. So we fight and
continue to fight for each
other

– Pamela Silwana
Gugulethu CAN

How to CAN

Connecting CANs

Together the CANs are building a response to the global pandemic that can help to transform the unjust economic, social and environmental challenges in our society.

Here is a straightforward guide to whipping up a CAN in your neighbourhood. They take many forms and come in multiple shapes and sizes - so make sure you do what works for you! No matter what form your CAN takes, make sure there is a healthy serving of energy, generosity, and care.

WHAT YOU'LL NEED

- Peeps who "get shit done" quickly - but with a big heaped spoon of care.
- A way to connect these peeps digitally. This ingredient is expensive, but you won't regret finding ways to get around the data issues. Being connected is delicious! WhatsApp groups are good, Facebook does the trick, and with Zoom for special occasions, you're all set.
- Plenty of collective energy and wisdom (everyone has something to contribute).
- Radical generosity. Share resources, yes - but also be kind.
- A web of personal connections and relationships of trust.
- Reliable and verified information. Don't act on rumours or conspiracy theories. Action needs to be informed by facts you can trust.

WHAT TO DO

First off, there is no one-size fits all approach here. Every neighbourhood is unique, filled with different kinds of people, operating in different ways. Like with any recipe, there are different ways that work. That being said there are some important ingredients to throw into the mix that will help make your recipe a hit.

1. Get your people together, a WhatsApp group might help but what you need is a small team to get going.

2. Identify the needs in your community. How will the pandemic affect everyone? Who needs to be supported? What are the most obvious ways that you can bring people together to take collective action? Are there communities outside your neighbourhood you could help?

3. Collective action. Take initiative, no one is going to tell you what to do. Prioritise local interventions that work, and recognise what you already know about your neighbourhood. Get creative and collaborate!

4. Don't be afraid to experiment with different ingredients, and make sure to share your recipe along the way

5. Rinse and repeat
Your CAN will evolve over time and find its groove, exciting new flavours will emerge, and you'll have a unique set of challenges to overcome. Here are some things to keep in mind as you stir the pot:

6. People are not going to agree on everything and will often have quite different ideas about how to do things. Find the points of agreement and focus on these, and let the rest percolate. Building a culture of "calling in" rather than "calling out", and inviting understanding and empathy rather than exclusion, is one way to do this. Use that radical generosity wherever you can.

7. Remember, Tina was right, we don't need another hero. You'll be surprised that when you trust others, it's not that difficult to make a 100litre pot of delicious food. Try to embrace a post-heroic leadership style. We need to support each other and value different kinds of contributions. It just doesn't taste good when one or two people dominate.

8. Don't spread anyone too thin - build slack into your system. Things will fall apart if they rely too much on individuals. Have overlapping responsibilities so that if someone needs to take a break, action can continue. Take time to breathe and check in with each other - burnout is all too easy and you need to actively guard against it.

9. Some of the best recipes are the ones we learnt from our grandmothers, but sometimes we need something fresh to spice it up. Stay open to both, and keep cooking till it tastes good.

10. Lastly, the most important ingredients in any the recipe are the relationships - this is what holds everything together, so it doesn't matter what you are cooking, make sure you handle these with care.

That's it. Remember that this only works if you go out and do it, and you CAN!

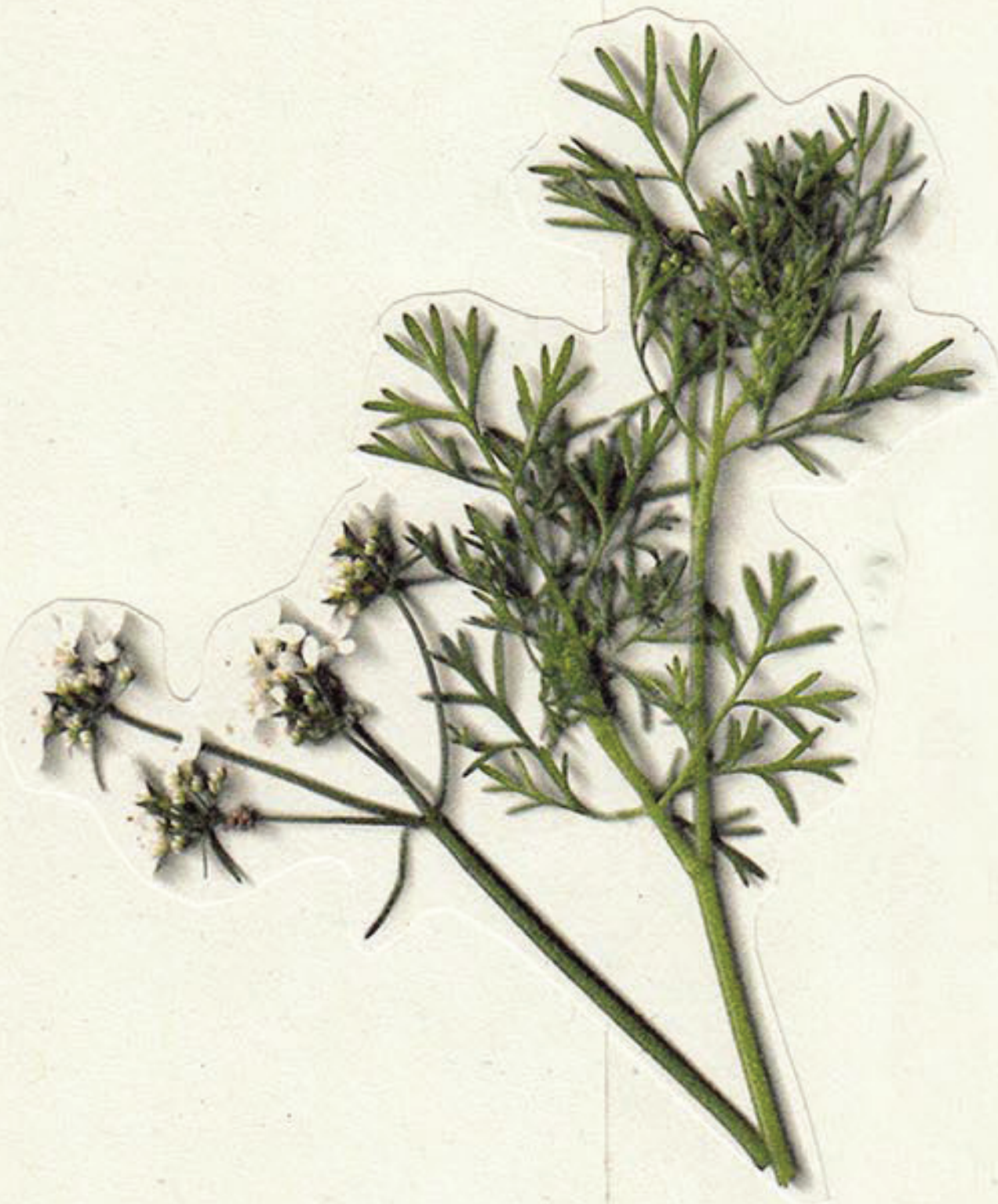

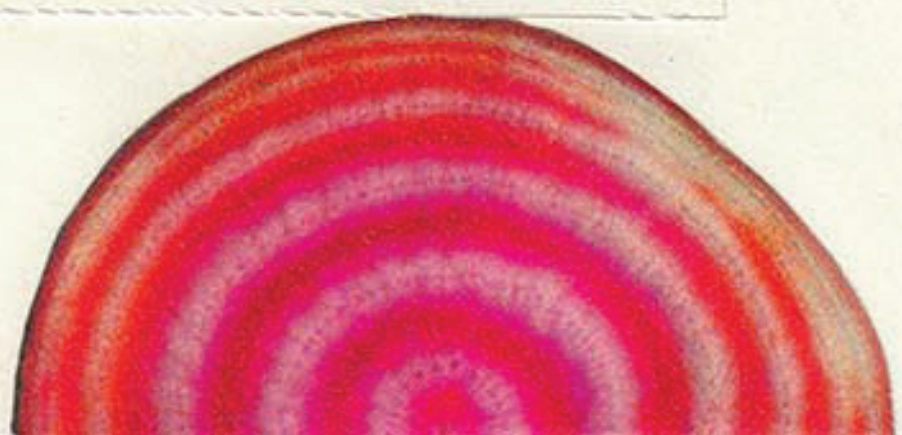

Tracing broken lines

CBD CAN

It confounds some people how this leaderless movement works. How we seem to work with and around each other. No single ideology. No centralised power structure.

People assume that it is accidental. Eagerly pointing out the gaps they see. The should haves. And could haves.

But we are more like a forest than a spreadsheet. It’s organic, self-seeding, adaptive, and nurtured as much by the seasoned expertise of organisers and activists as by the wide-eyed dreams of newcomers.

There is no single truth. The aim is multiple perspectives.

If something doesn’t work, we change, we grow. No guilt. Lots of pressure, sure. Pain, sorrow and sometimes despair. But only for a moment. Because there is always another angle, words of encouragement, something more to be done.

It is what it is because it needs to be. We’re tracing the broken lines. We’re filling in the gaps. And when we don’t know, we pause. We reflect. We wait. We talk. We learn. We watch. We build relationships.

In between our lives we find each other. In our voluntary hours. We understand. We come to trust.

By Zoe Valour

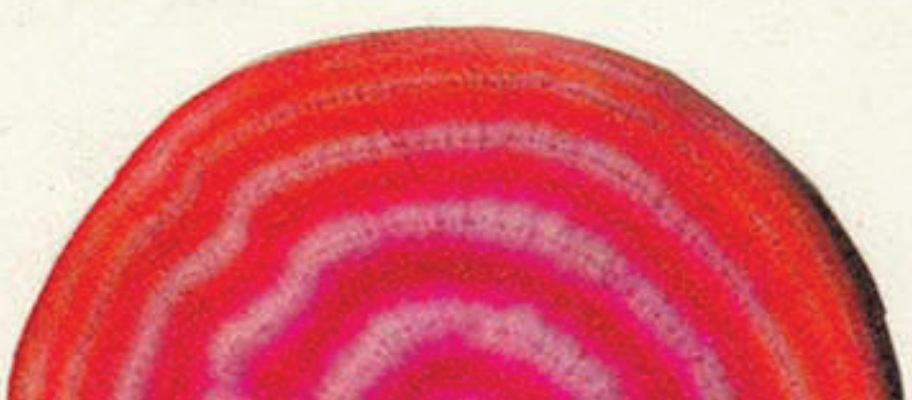

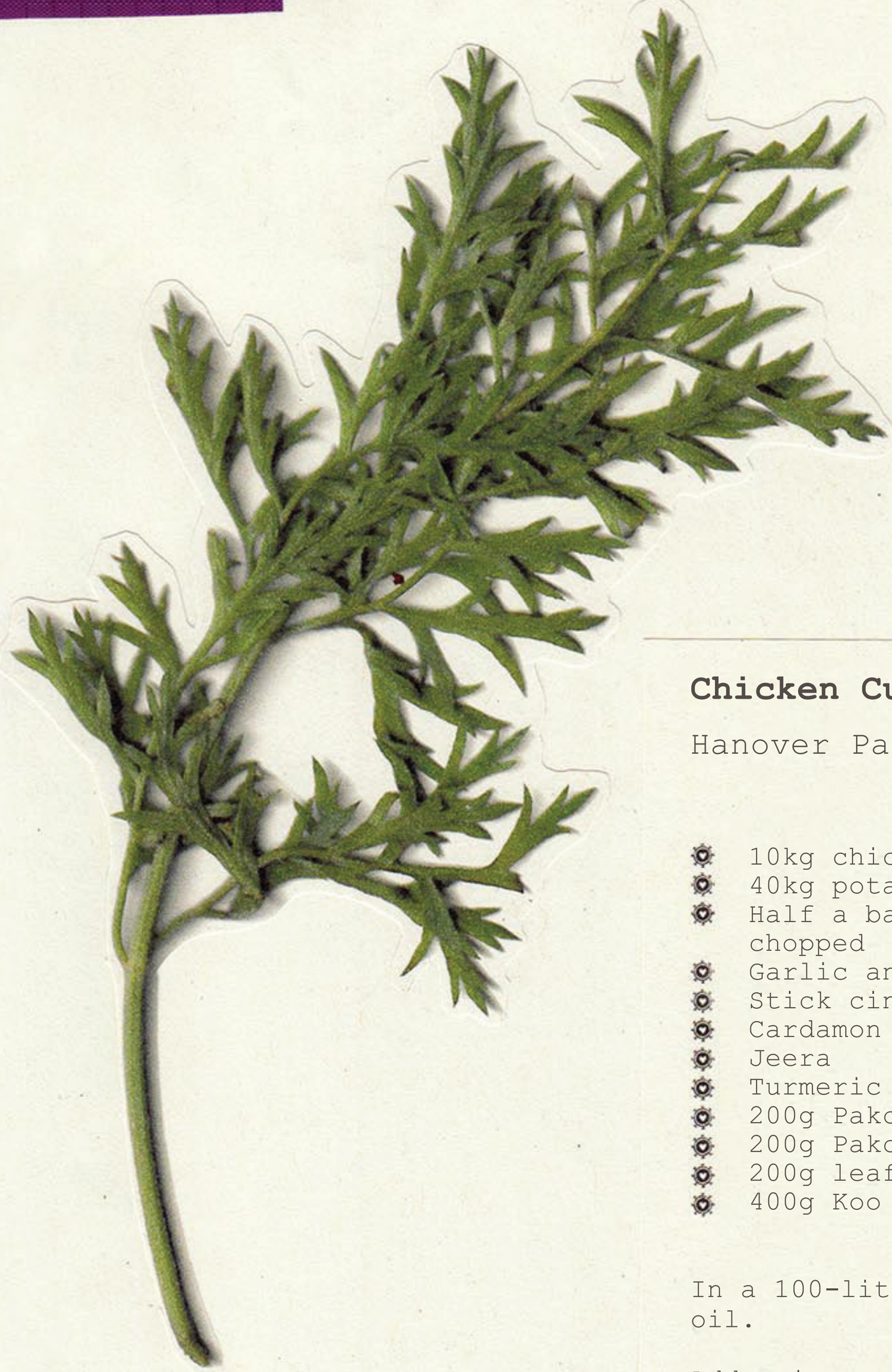

Chicken Curry

Hanover Park CAN

- 10kg chicken
- 40kg potatoes
- Half a bag of onions, chopped
- Garlic and ginger
- Stick cinnamon
- Cardamon
- Jeera
- Turmeric
- 200g Pakco curry powder
- 200g Pakco masala
- 200g leaf masala
- 400g Koo tomato paste

In a 100-litre pot, heat fish oil.

Add onions and fry until lightly brown

Stir in garlic and ginger

Add a stick of cinnamon, and cardomon

Add 3 litres of water and other spices

Add chicken and potatoes together

Boil until potatoes are bit soft

Add tomato paste

Add salt and curry leaves

Simmer on a low heat

By Yaseen Johaar

Love in the time of Covid

Clovelly CAN

Clovelly is a small obscure suburb tucked between Fish Hoek and Kalk Bay. We are proud that we have 'love' in C'love'lly.

Our CAN started right at the beginning of the lockdown. We already had quite close-knit, ongoing friendships among us and even at the times of other crises like fire and water shortage, we were able to come together.

So, it was quite natural for us to pull together and discuss whether we were going to be okay under the lockdown, and how we wanted to help other people who may be struggling.

Our CAN is very organic and very organized at the same time. We have 75 residents in the CAN WhatsApp, with an 'allocation' team of 7 people who have been meeting at least once a week, mostly on Zoom but also 'mask-to-mask'.

Many Clovelly residents took the initiative to help others in different ways. Many people dropped-off in-kind donations. One person organized weekly food parcels via Desmond Tutu Foundation in Masiphumelele. A young man who belongs to the 2nd Fish Hoek Scouts organized the 'Jar of Hope' campaign for the Mandela Day and donated 165 jars of home-made soup mix for people in need. Every now and then, someone makes a request for assistance on the WhatsApp Group and people respond warmly and quickly.

As the allocation team, we decided to take quite an organised approach by financially supporting two partners so that we could make a real difference without being spread too thin, and form long-term relationships with our partners. We set up an account with Common Change so that we are accountable and transparent although they take 5% of our funds!

One of the partners we chose is Wesbank which is 40km away from Clovelly, next to Delft and Kuils River. Why choose such a far place and why Wesbank? For two reasons. Firstly, we already had a relationship with community members there. One of our CAN-members has been going to Wesbank for 15 years for her Buddhist group activities and also teaching origami in Wesbank No1 Primary School.

Secondly, it's precisely because they are far! Because of the architecture of Apartheid, many townships are far from the well-off suburbs and they often do not receive resources. We felt choosing to support one far-away place was important.

Because of the high unemployment rate in Wesbank, churches and individuals have been running soup kitchens for many years. We did not set up something new, but rather plugged into this operational network.

At first, we started supporting a few soup kitchens through a community leader called Edith who successfully piloted the 'Walking Bus' initiative. Through Edith, we got to know the newly elected councillor who is very hard-working and organised. He asked us to support 24 soup kitchens in his ward covering 5 townships with whatever we could offer! His ward had been divided into 12 blocks and 2 soup kitchens in each block were identified. So, we did some research and decided to supply soup mix, which can keep for a long time and also has a high nutritional value.

We formed a relationship with the supplier, Yellow Submarine in Ottery, who gave us a very good discount. In May we started delivering to 24 kitchens, and in October we reduced to 10 kitchens. Each kitchen feeds 80-300 meals once or twice a week.

In August, we started distributing Wonderbags to 3 food garden initiatives. We also had a Kite 'flash fly' with the kite-making children linked to Cape Mental Health Kite Festival, and a community fundraising market (Hope Market) at one of the new food gardens, which raised funds to buy a water tank for that garden.

Our other partner is Ocean View Organic Farm. They are quite famous now, but when we started supporting them, they were only starting out. One young man from Clovelly, who volunteered at Neighbourhood Farm in Fish Hoek in his gap year, knew about the Ocean View farm initiative, and so the partnership started from that tiny seed.

Now we sponsor them to bake 150 fresh loaves of bread weekly for the soup kitchens in Ocean View and Masiphumelele run by an organisation called Ukama Foundation. By supporting and working with the existing local initiatives we have been able to support hundreds of people! Many Clovelly residents now order a weekly fresh veggie box from the farm. So, many seeds of friendships are sprouting and a new urban small-scale farming cooperative has been formed in our area.

How do we keep going with our fund? Initially we had many people donating to the account and gradually the number started to drop. So, we initiated a number of fundraisers, such as a raffle for a weekend stay at a Clovelly guest house, an art auction on Facebook, a garage sale, a cake sale, and we are even organising a music concert by Derek Gripper.

We keep having fun as well and getting to know more people within Clovelly. Honestly, we don't know what the future holds but we are willing to continue as long as possible. Let's keep planting seeds of love now.

By Clovelly CAN Allocation Team

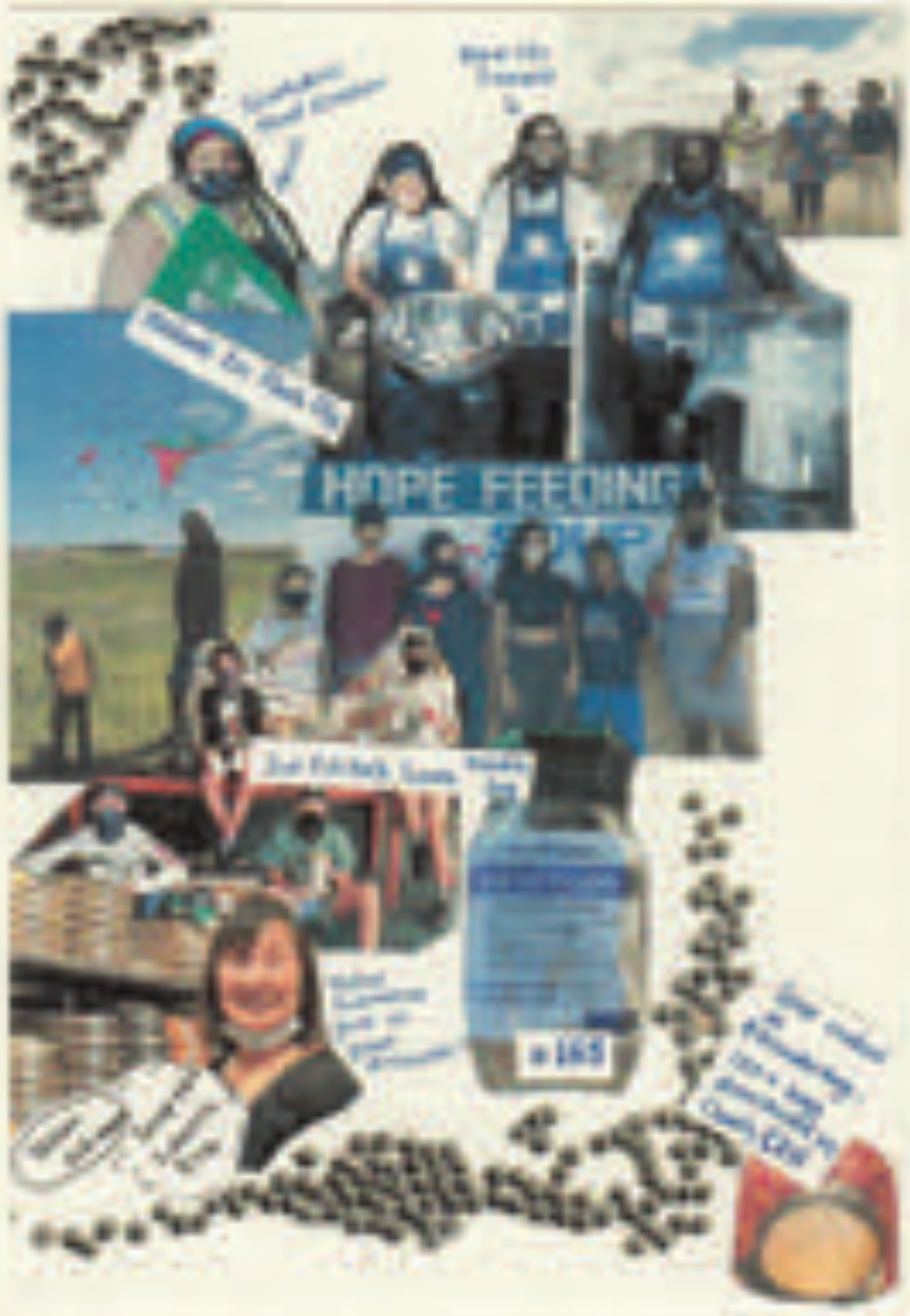
HOPE FEEDING

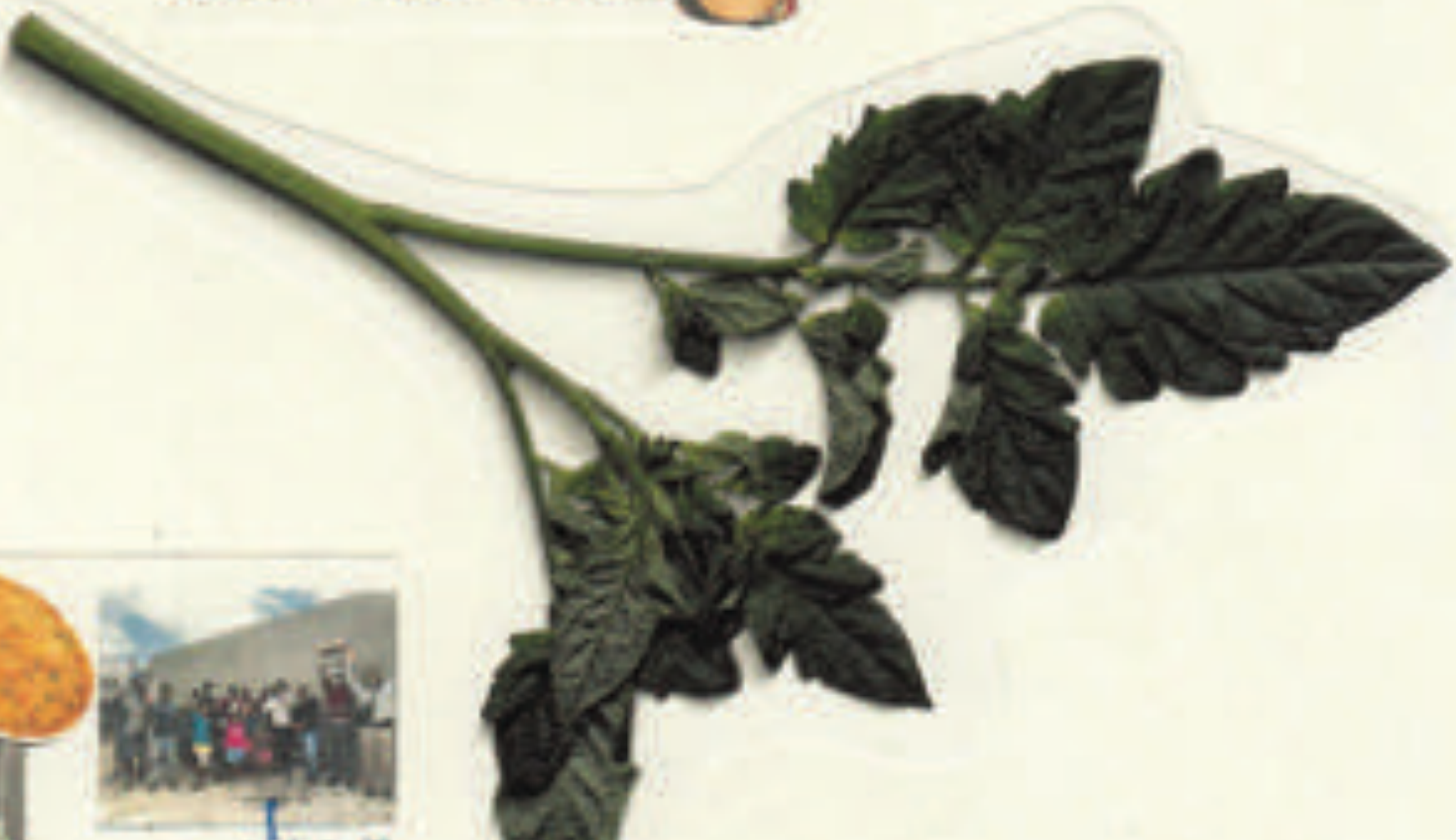

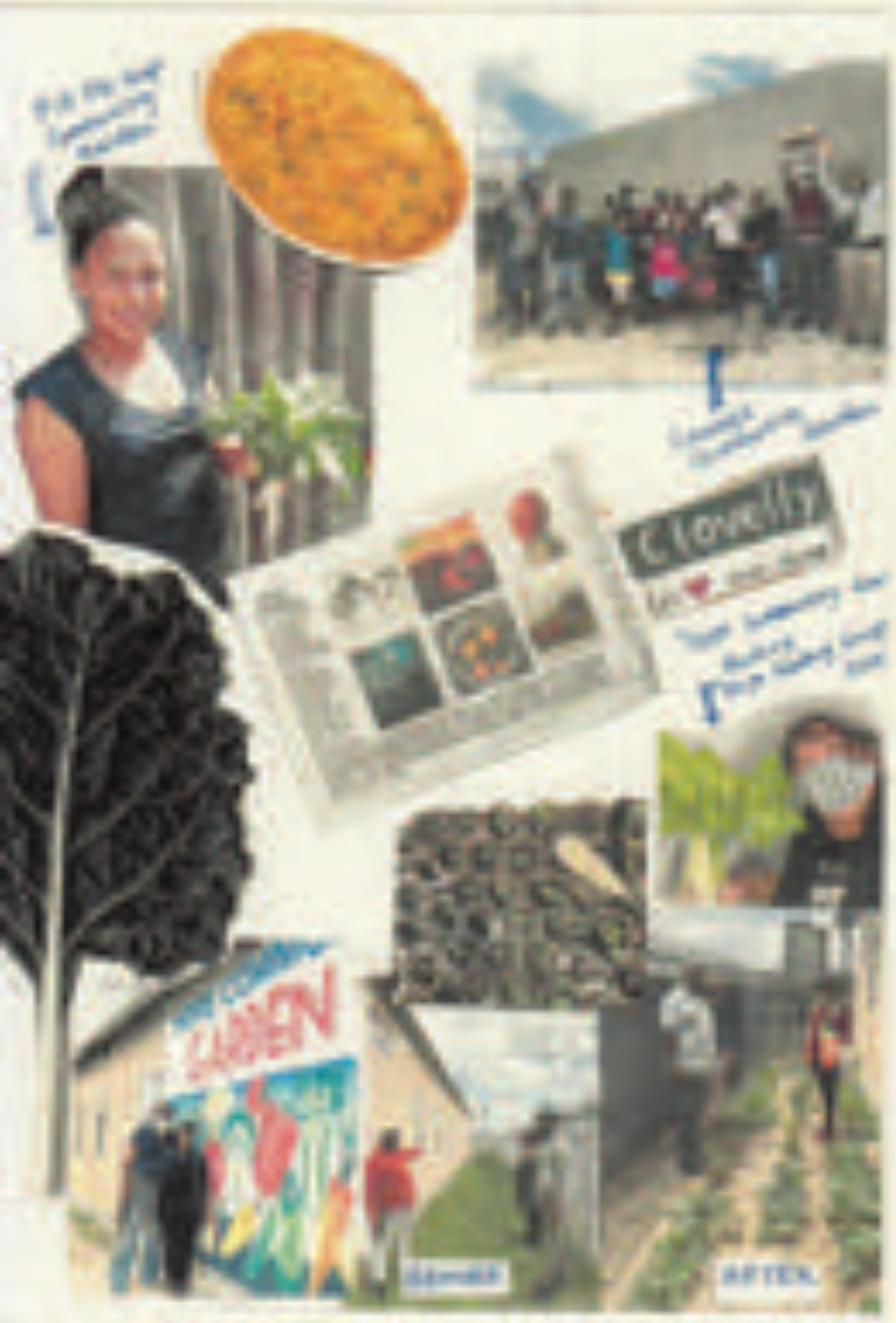
Clovelly
GARDEN

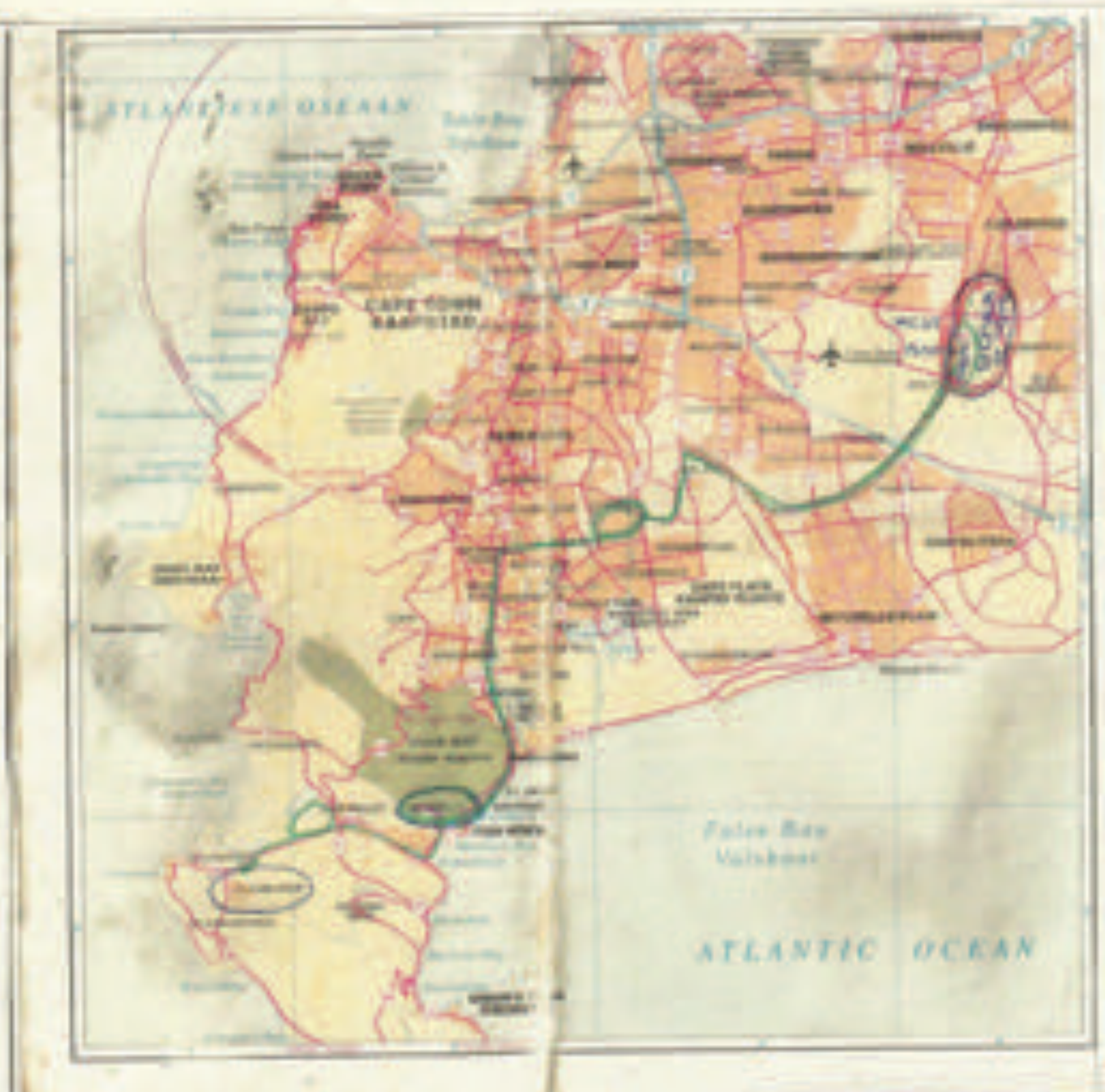
CAPE TOWN
False Bay
Valsbaai
ATLANTIC OCEAN

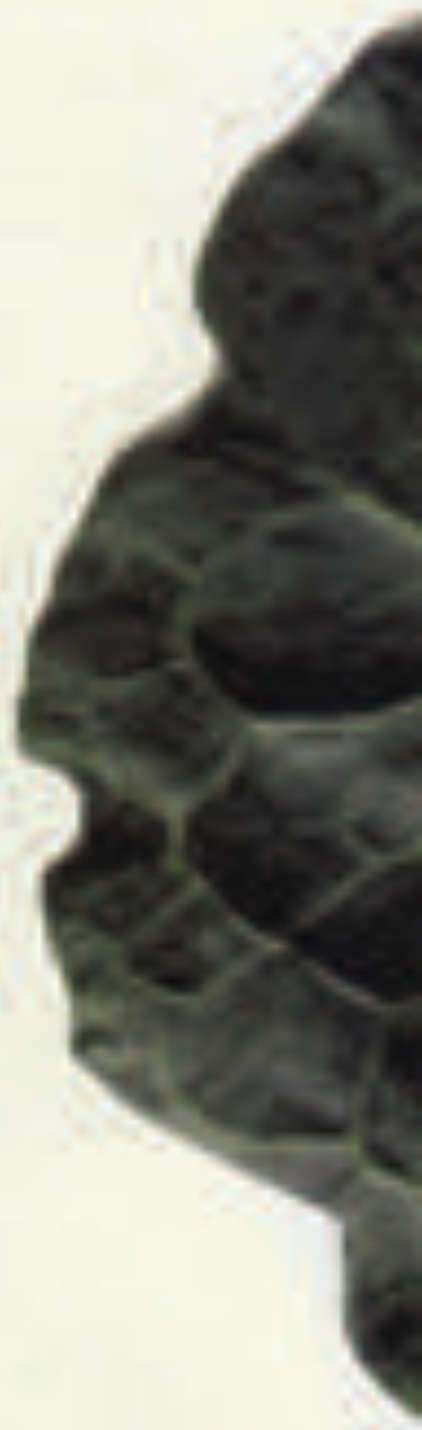

"The resistance is about being better in relationship not in opposition. We must, as a matter of course, pull down the oppressive and extractive system in order to be in relationship"

- Tunde Wey

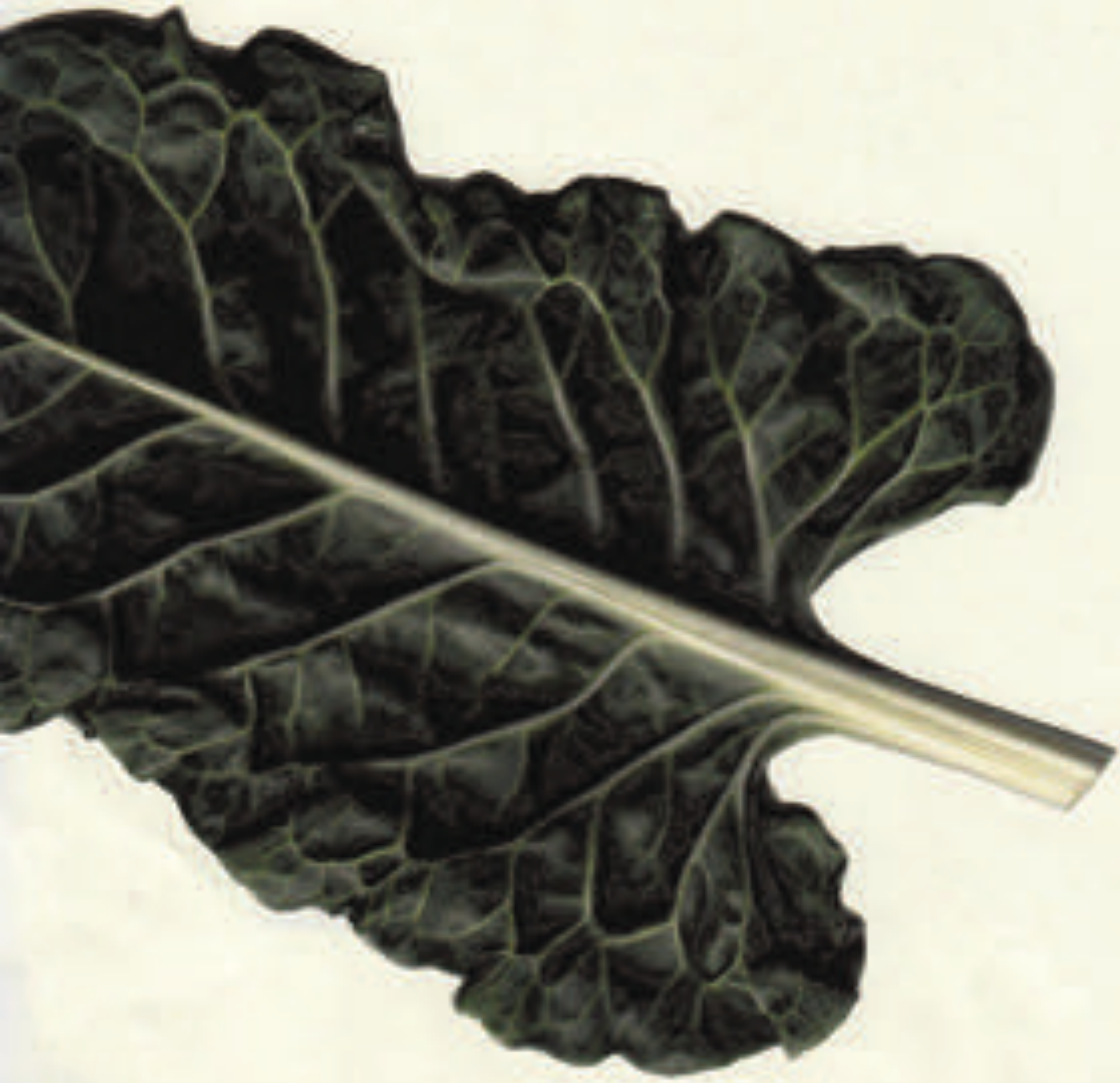

Fish Breyani by Theresa Steenkamp (Block A, Wesbank)

Cook the breyani and the rice & lentils separately.

1. Mix spice (Robertson's spice, herbs, brown onion soup powder)

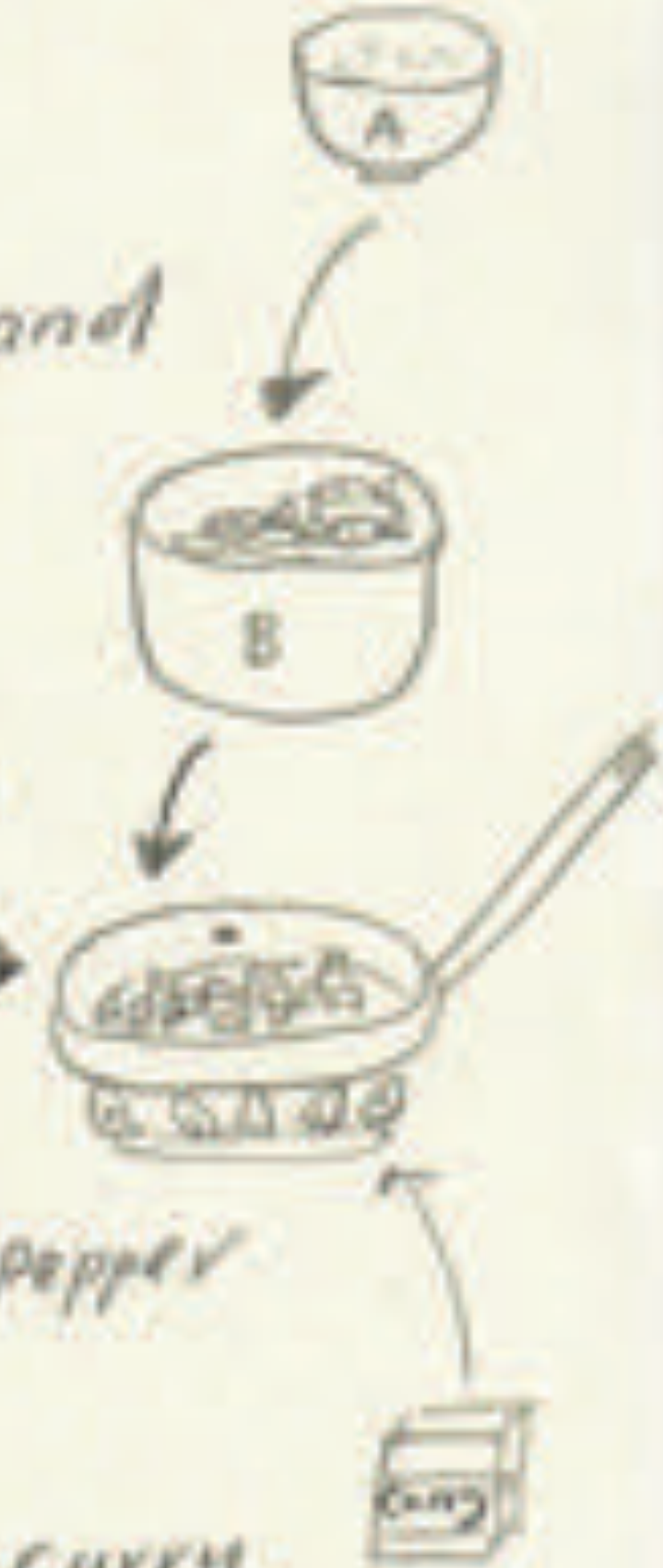

2. Put tin fish in a separate bowl and Mix together with spices.
3. Fry onion and peppers together.
4. Add Fish mixture
5. Add little bit of salt

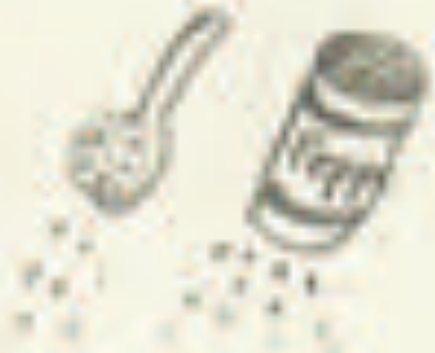

6. Mix curry together (I don't use hot curry because of the kids)
7. Cook this Fish Mixture till it's nice + smooth.

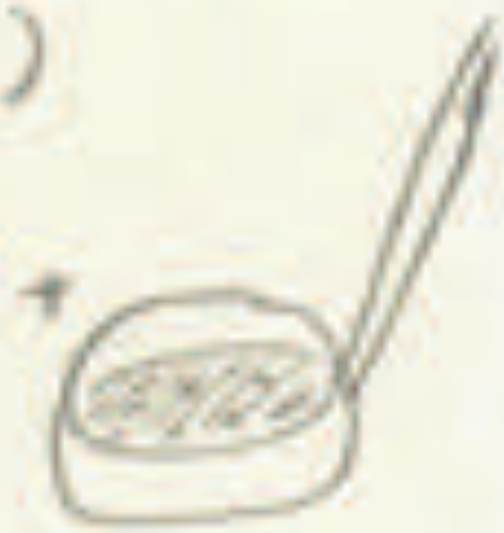

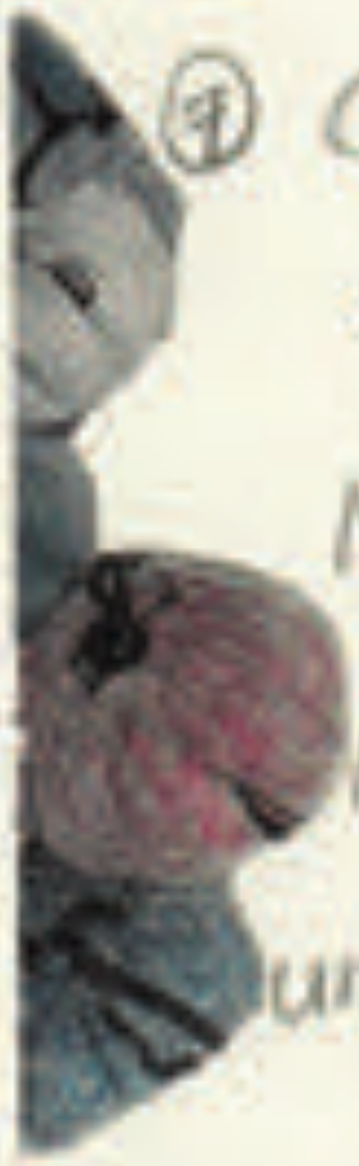

Mix lentils + rice together in a pot with water.

Add the Fish mixture into the pot bit by bit, until the lentils and rice are finished cooking.

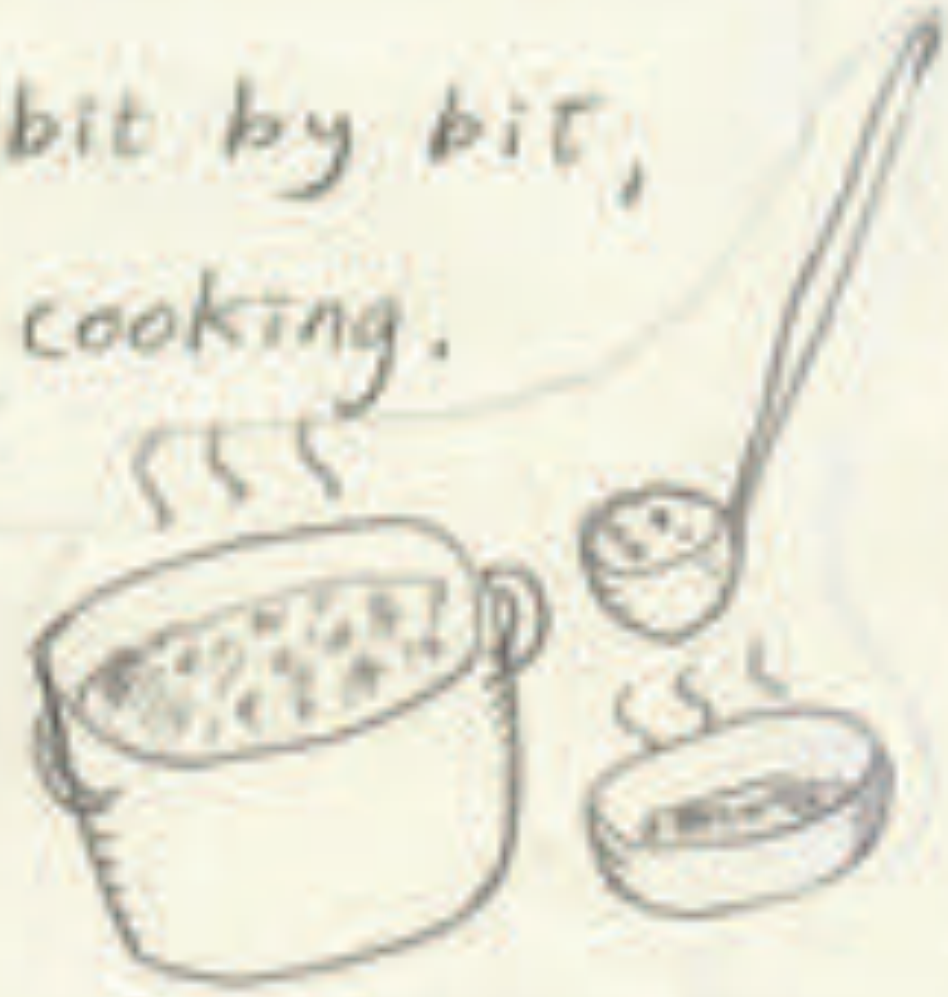

Turkish Chickpea Stew

Muizenberg CAN

Travel has opened my eyes to global flavours, weird and wonderful.

Sadly, Covid-19 has created travel restrictions globally. But our taste buds are still free to travel.

With this recipe, I wanted our Muizenberg CAN community's taste buds to travel to Turkey.

So, I made a simple vegetarian Turkish stew, with a raw salad and plain white rice.

This recipe is scaled down for 4, however we fed approximately 200 from our large volume pots.

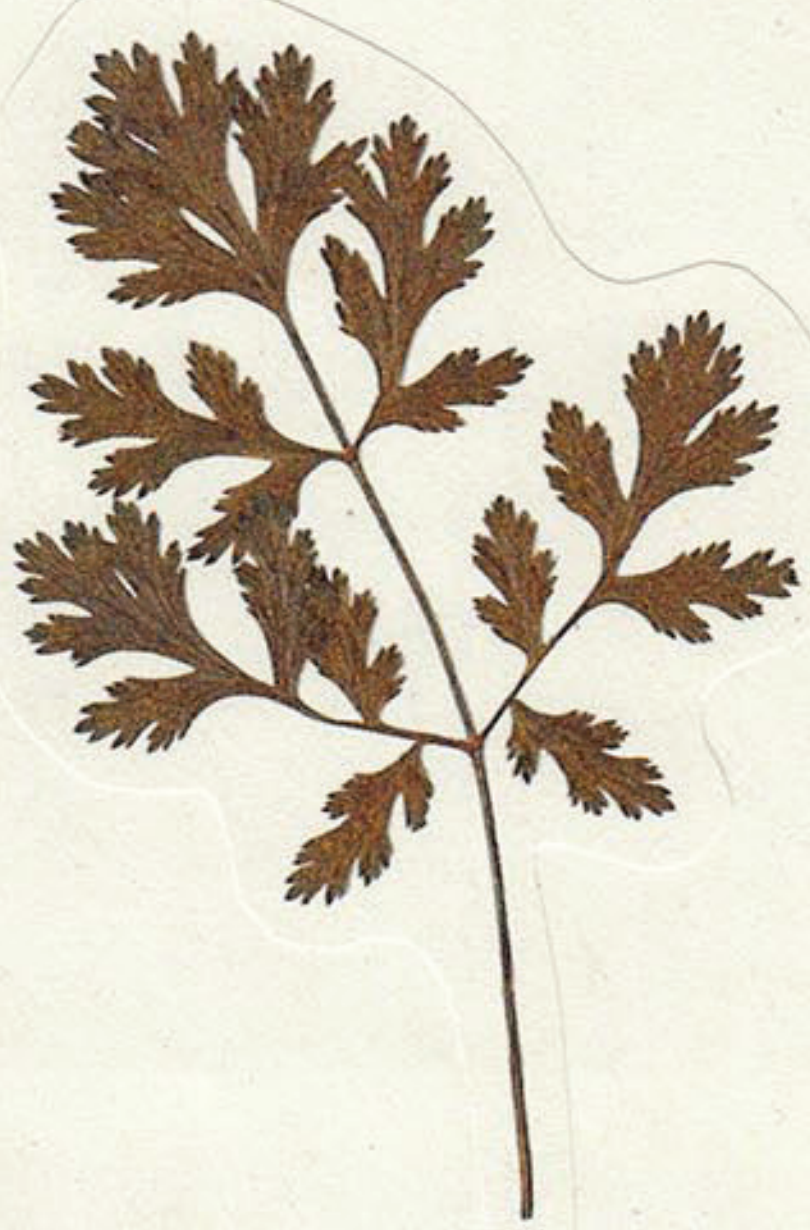

- Olive oil
- 4 white onions
- 2 stems fresh celery
- 500ml fresh tomatoes mix (blended with smoked paprika and 2 carrots)
- Spices * tablespoon each of dried mixed herbs, cumin, garam masala, smoked paprika
- 2 cups pink (red) lentils
- 2 cans chickpeas
- 500ml chopped/diced carrot

Chop onion finely and fry in olive oil in pot until caramelized

Add garlic and celery and fry

Add spices and fry further (watch spice does not burn)

Add 500ml fresh tomatoes

Add 2 cups pink(red) lentils

Boil till thick consistency and sauce becomes thick

Add chickpeas and carrots

Boil further till carrots cooked

On the side make simple red cabbage salad (shredded cabbage in lemon, oil and salt dressing)

Serve with cous cous or flat bread or white short grain rice. Add fresh mint on top and fresh lemon rind

We hope you enjoyed your taste-buds travel to Turkey, made in Muizenberg by Max.

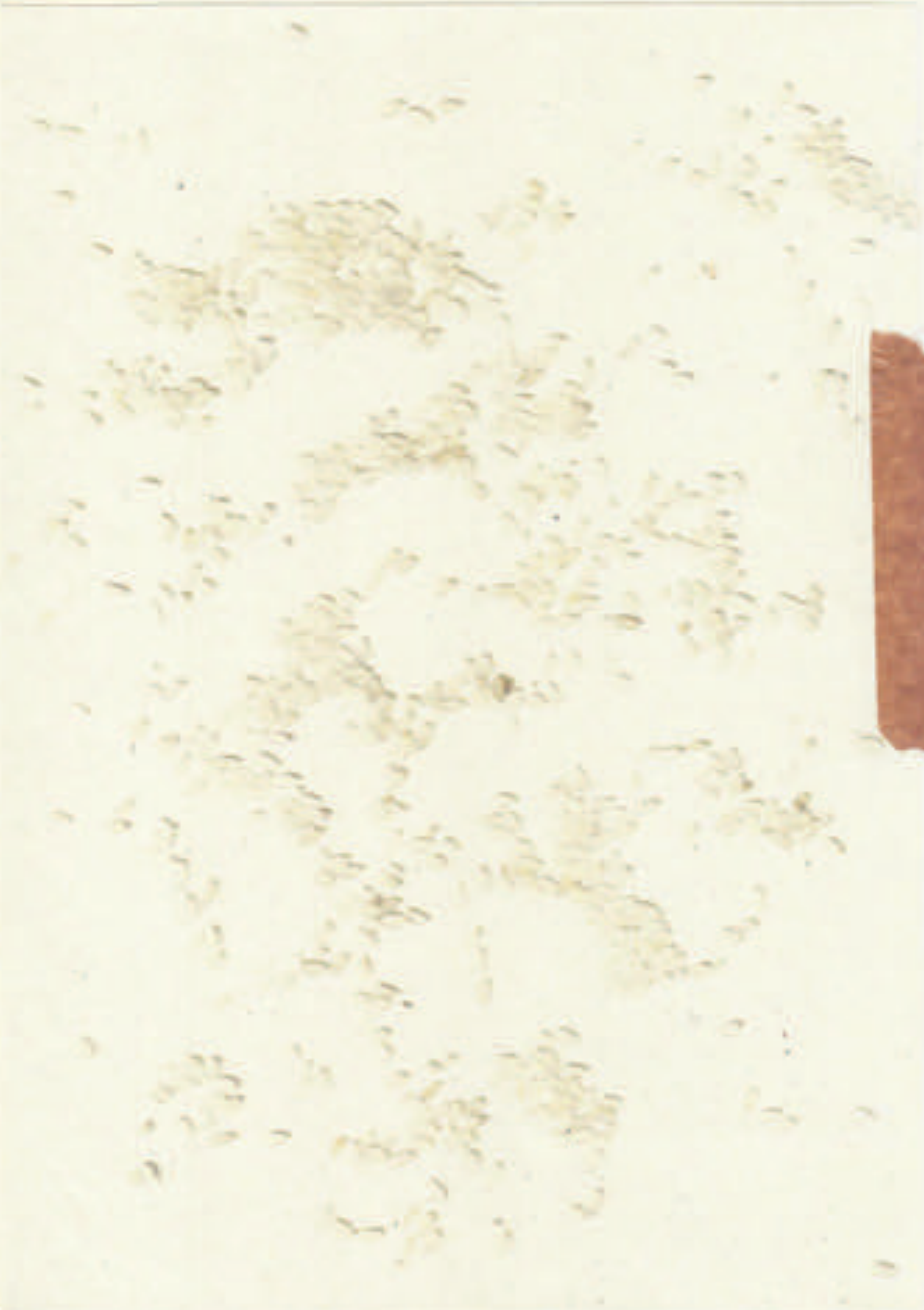

Recipe for growing your own herbs

Salt River CAN

Even if you don't have a garden, you can grow your most loved herbs at home. Herbs are all the more delicious if you have been tending and helping them grow yourself.

- *Sunny window, balcony or outside spot.*
- *Yogurt Tub*
- *Scissors*
- *Soil or a bit of compost*
- *A seed, seedling or cutting*
- *Water*

Use the scissors to poke some holes in the bottom of the yogurt tub

Put your soil or compost mix into the tub. Push the soil down very lightly. Remember, plant roots need lots of oxygen, so make sure you are not compressing the soil too much

Push your seed in the soil about 0.5cm deep

Water the seed gently but thoroughly

Put the tub in half sunny, half shady spot

Water it regularly

In two months, you can harvest your herbs

By Marius Zenker

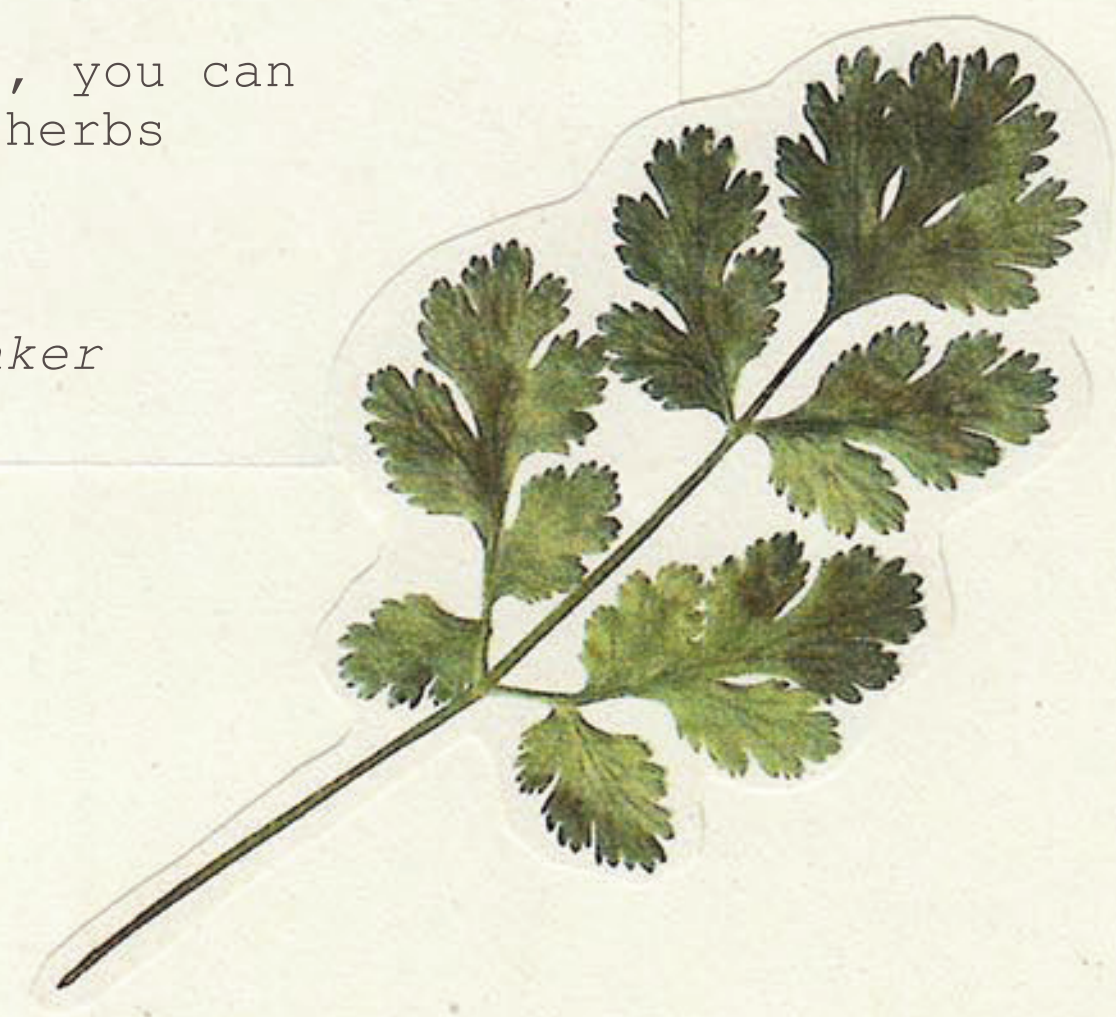

How to plant beetroot

Clovelly CAN

Beetroot can be used in salads, roasted with other vegetables, or added to vegetable juices.

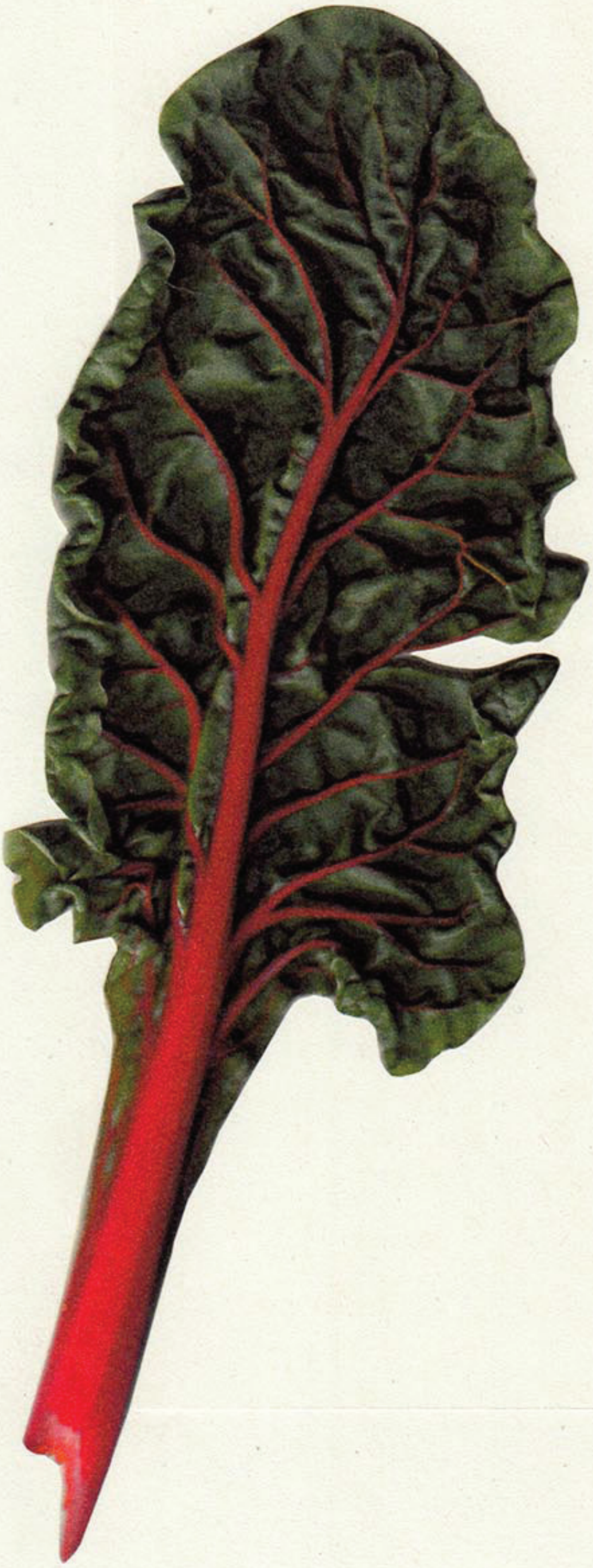

1. Prepare seedling potting soil by mixing sand and compost together.

2. Pour seedling soil over seedling tray and ensure that all compartments (holes) are filled up, and soil is level.

3. Make a small dent in the soil with your finger (not too deep!).

4. Place one seed in each hole and gently cover seed with soil.

5. Water the soil gently. Make sure each hole gets some water. If you have kelp solution, add some to the water before you water, to encourage root growth.

6. Place seedling tray in an area that gets both sun and shade. Not direct sun all day – that's too much sun.

7. Seedlings will start to grow after a few days.

8. Prepare your garden soil by adding compost to the sand. Make small holes in the garden bed about 20 cm apart.

9. When each plant has germinated and is about a pinkie finger tall, gently pull the seedling out of the tray by its stem, and plant into your garden beds, and water.

10. Water the seedlings in the garden every one or two days.

11. Harvest your beetroot after about 6-9 weeks.

12. Enjoy!

By Stephanie Goosen

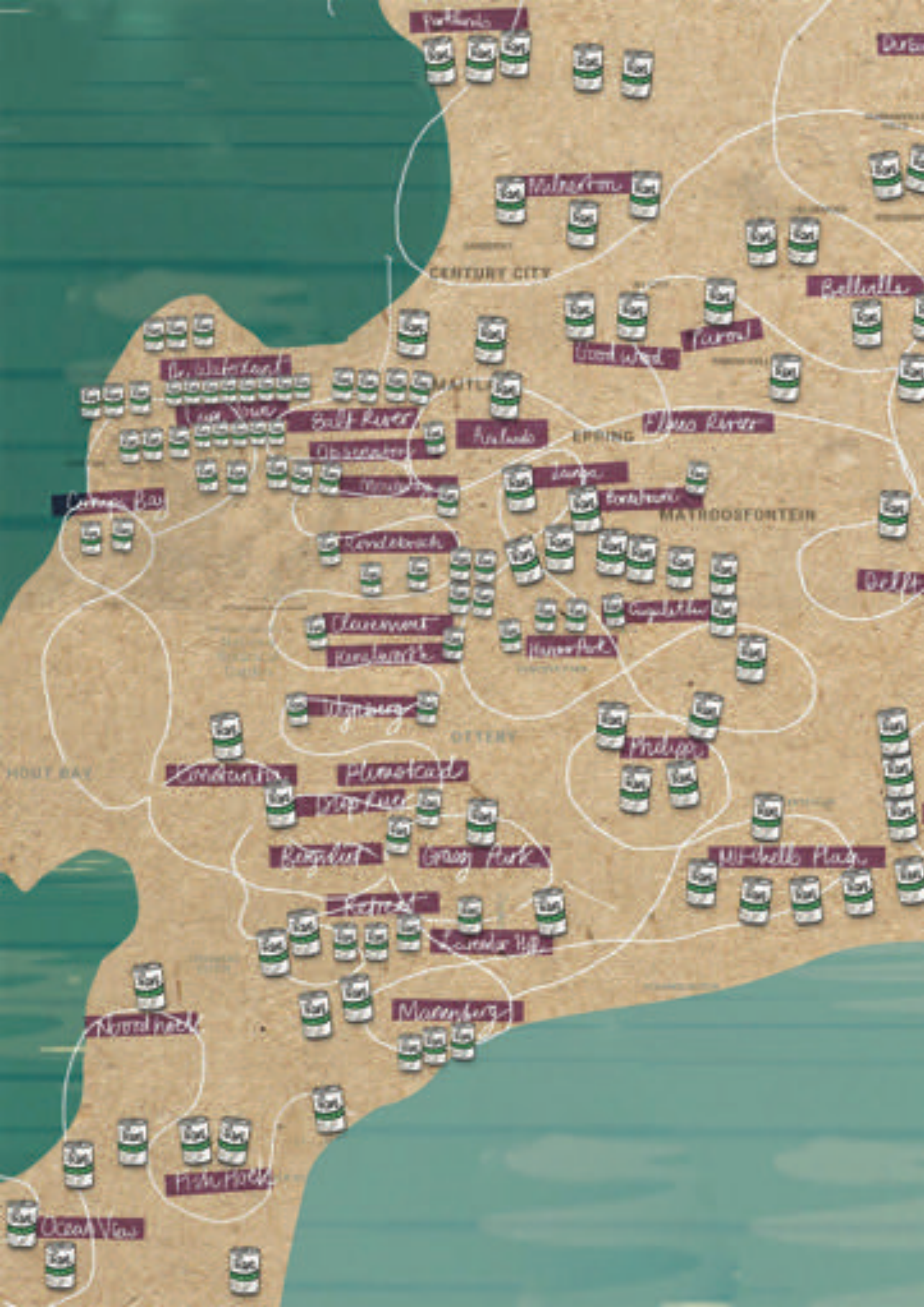
CENTURY CITY
EPPING
MATROOSFONTEIN
OTTERY
HOUT BAY
Milnerton
Bellville
Goodwood
Parow
Elsies River
Cape Town
Salt River
Observatory
Camps Bay
Langa
Rondebosch
Claremont
Kenilworth
Wynberg
Hanover Park
Philippi
Constantia
Plumstead
Diep River
Bergvliet
Grassy Park
Retreat
Lavender Hill
Mitchells Plain
Noordhoek
Fish Hoek
Ocean View

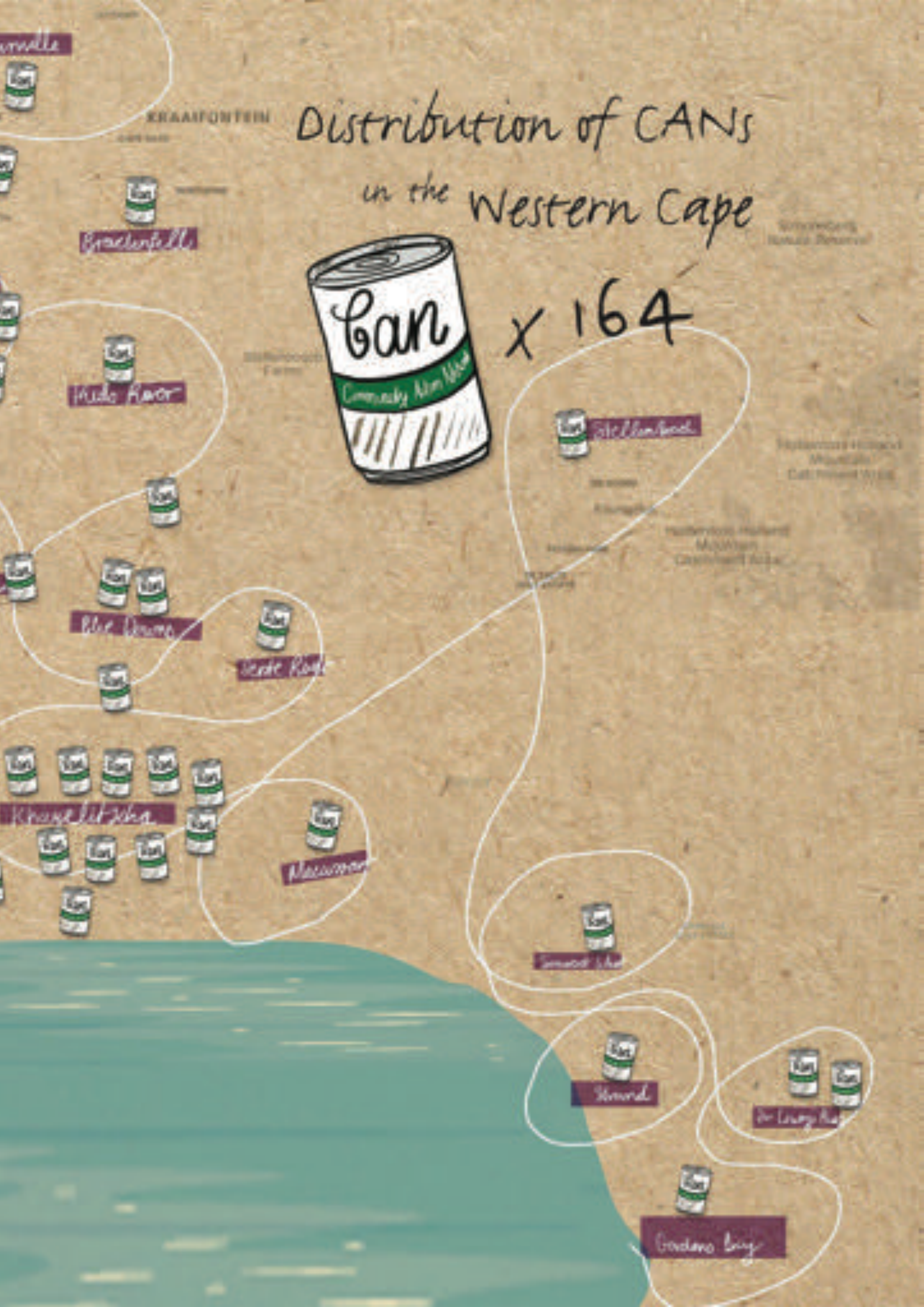
Distribution of CANs
in the Western Cape
Can
x 164
KRAAIFONTEIN
Brackenfell
Blue Downs
Stellenbosch
Strand
Gordons Bay

"I wanted to do something with my hands. At least I can make a hundred loaves of bread. Just to feed some children, people who don't have anything to eat. It's a real honour because there is so many people, they are struggling that go sleep without food but at least they are getting a loaf of bread, even if I must get up at five o'clock in the morning or even three o'clock or even two o'clock. I do that for them."

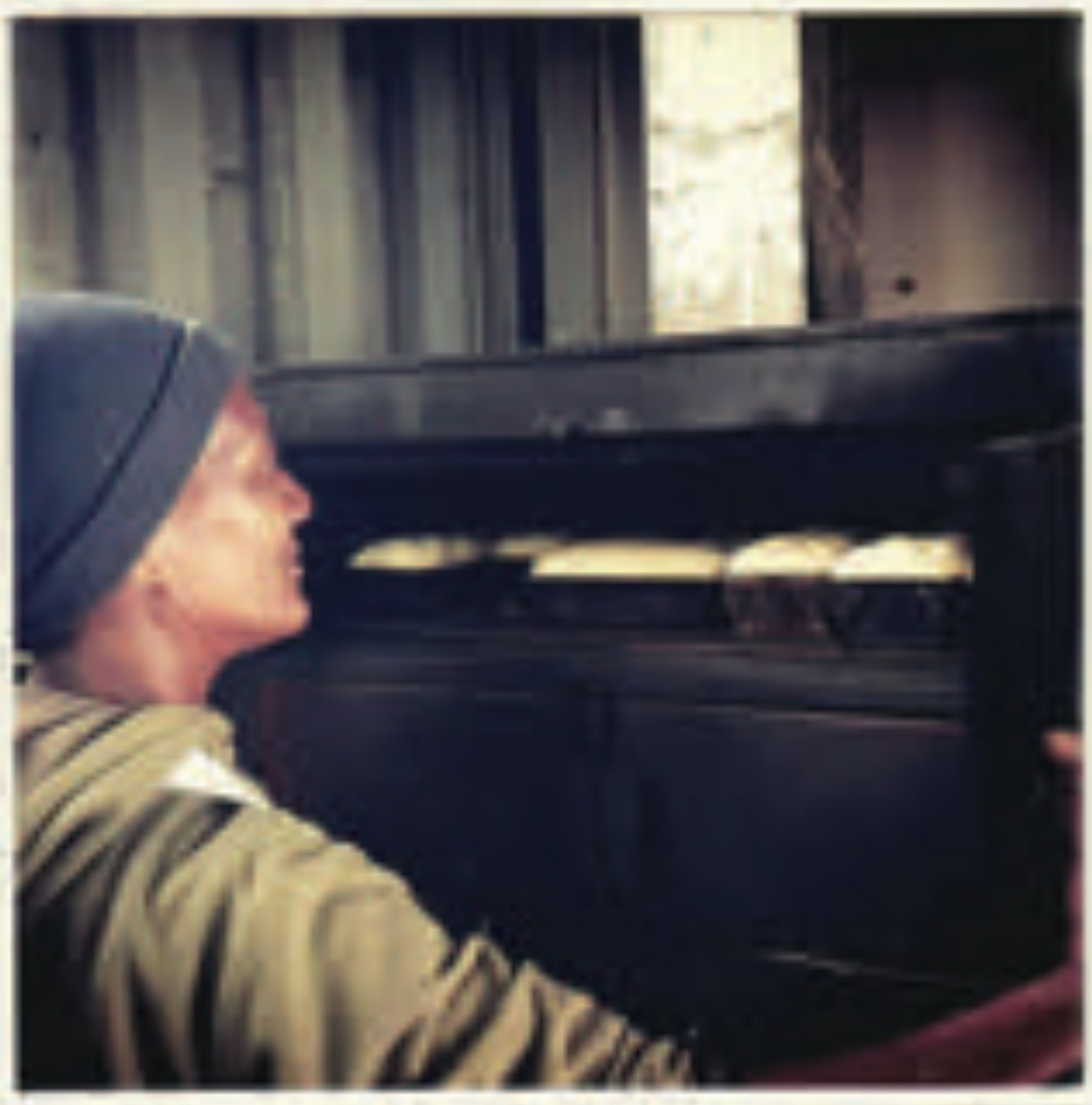

– Jasmine Thomas
Ocean View Organic Farm

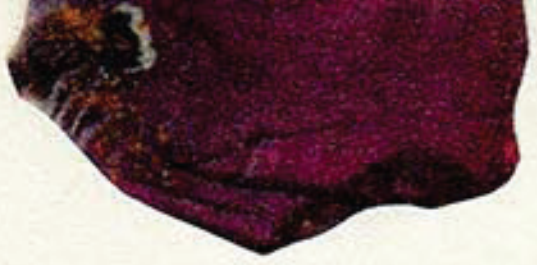

Solidarity not charity

Gugulethu-Seaboard CAN

When President Cyril Ramaphosa announced the lockdown, the 21 days sounded like a death sentence for the many unemployed and precariously employed people. Yes, it was essential to save the lives of the thousands of elderly and high-risk South Africans, but there was no clear emergency plan for food security – not in the townships or the rural areas, where 10 million unemployed were expected to stay at home with empty cupboards and children who were suddenly denied their school meals.

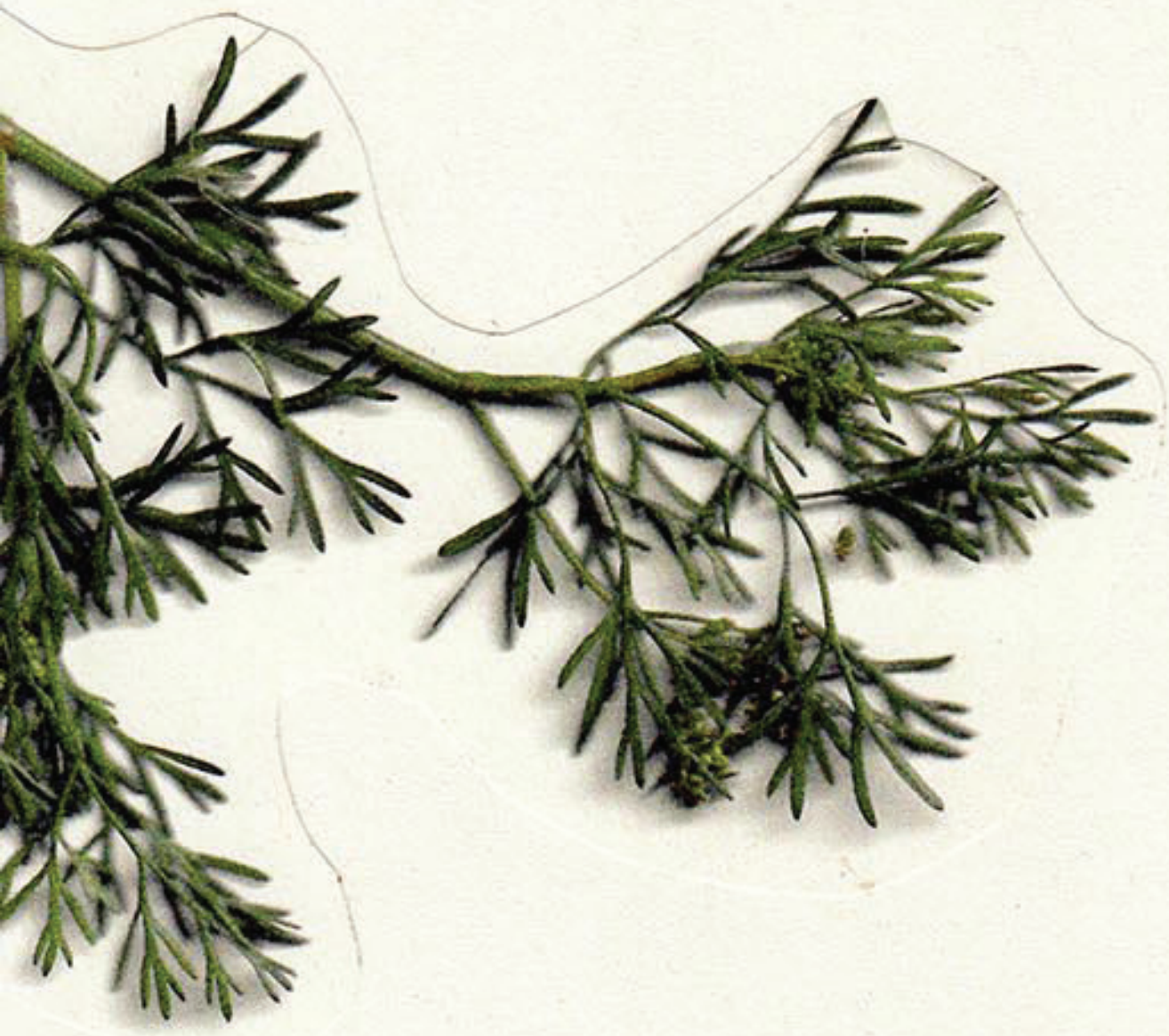

Into this, came the Community Action Networks (CANs). As the virus began to spread across Cape Town, so did these informal networks of purpose. We began to connect and organise through the Cape Town Together platform. Across the Seaboard, a collection of (mostly) strangers linked through WhatsApp. In Gugulethu, we hosted a house meeting with a collection of neighbours, contacts, and Organising for Work (OfW) members. Together, we prioritised needs and identified street champions who would take responsibility for supporting the areas they lived in. At the top of the list was food for everyone and running water in the informal settlements. Then it was soap and toilet paper. At the Seaboard, the CAN took various actions to assist unhoused and vulnerable neighbours, as well as residents of the Ahmed Kathrada House (AKH) – a former nurses' accommodation occupied by Seaboard residents who could no longer afford rising rents in rapidly gentrifying neighbourhoods. However, it was always clear that since concentrations of need and resources would overlap with Cape Town's apartheid geographies, bridges had to extend across those divides.

On the 26th of March, less than 36 hours before lockdown began, we forged our partnership as the Gugulethu-Seaboard CAN. Some of our members had (vaguely) known one another through OfW. Our intention was to do as much as we could before lockdown, but also build bridges and support networks that would last into the future.

By the morning of the 27th, supplies began arriving in Gugulethu. Hundreds of litres of water for Gugulethu's informal settlements, soapy water bottles, water containers and information pamphlets.

What was to be three weeks of lockdown, turned into eight months. As our collaboration grew, so did our 'circle of friends': Ladles of Love; Souper Troopers; Woodstock Breweries; Laughton's Hardware; Bentley's bakery; In2Food, Move for Two and the Western Cape Angel Network, among others, all facilitated a transfer of material resources from areas that have too much to areas that have too little. Food parcels and shopping vouchers were distributed by street champions. One kitchen grew into two, then three. At the height of the lockdown, 38 kitchens operated in Gugulethu and two in Sea Point.

Even while we were sprinting to meet urgent needs, we were concerned with the bigger picture, and what a holistic approach would look like. Our Sinani vouchers campaign ring-fenced 20% for purchases from Gugulethu-based fresh produce traders. Tired of spending money on bread from large retailers, we set out to find a Gugulethu-based baker with whom we could work instead. In Gugulethu, Vuyani Qamata, one of the street champions, brought together both experienced and 'newcomer' Gugulethu farmers, and the Gugulethu Urban Farming Initiative (GUFI) was born. During the lockdown, they revitalised or started 15 food gardens. Food gardens were also started in the Sea Point library and at AKH.

We have hit bumps on the road. We've spent time on things that didn't go as planned. Donor fatigue has set in. Volunteers are exhausted. Efforts to cooperate with government have been disappointing. Many government officials agree that in the first wave, our help staved off even greater hunger. Yet little is being done to remove the barriers between us, find ways to harness the goodwill, innovation and speed with which we were able to move, and ensure that public resources amplify our capabilities.

So far, for all our efforts, we have yet to fundamentally change the structural conditions in our society that make us all vulnerable. The causes underpinning the grossly skewed distribution of resources are still in place. But we do believe that the cross-suburb networks of support we have built, and the relationships we are deepening, can be transformative. For too long, networks of care have been severed along apartheid boundaries, distributing the greatest burdens to those with the least, and the smallest to those with the most. We hope that our CAN partnership is a tiny crack in that mould.

As we were writing this piece, 20 homes were burnt in Gugulethu. Within 12 hours, the affected families were provided with food and accommodation, while furniture and funds to help them start again are being collected. To be sure, our response is a charitable one. But as we move forward, we will continue reflecting on the overlaps and divergences between charity and solidarity, and ensure our work is situated in the latter.

As we look back on this year, and reflect on the inequities covid-19 exposed, and the wreckage it has left behind, we also find ourselves to be a group of people who refuse to go back to what was once considered 'normal'. As the second wave approaches, we may be tired and exhausted, but giving up is not an option. We have shown to ourselves that even where government has failed, we can take steps to correct the wrongs of our past, and present. We hope that in time, they find ways to harness our offerings and work with us. Failing that, we will continue making our own way.

By Pamela Silwana and Keren Ben Zeev

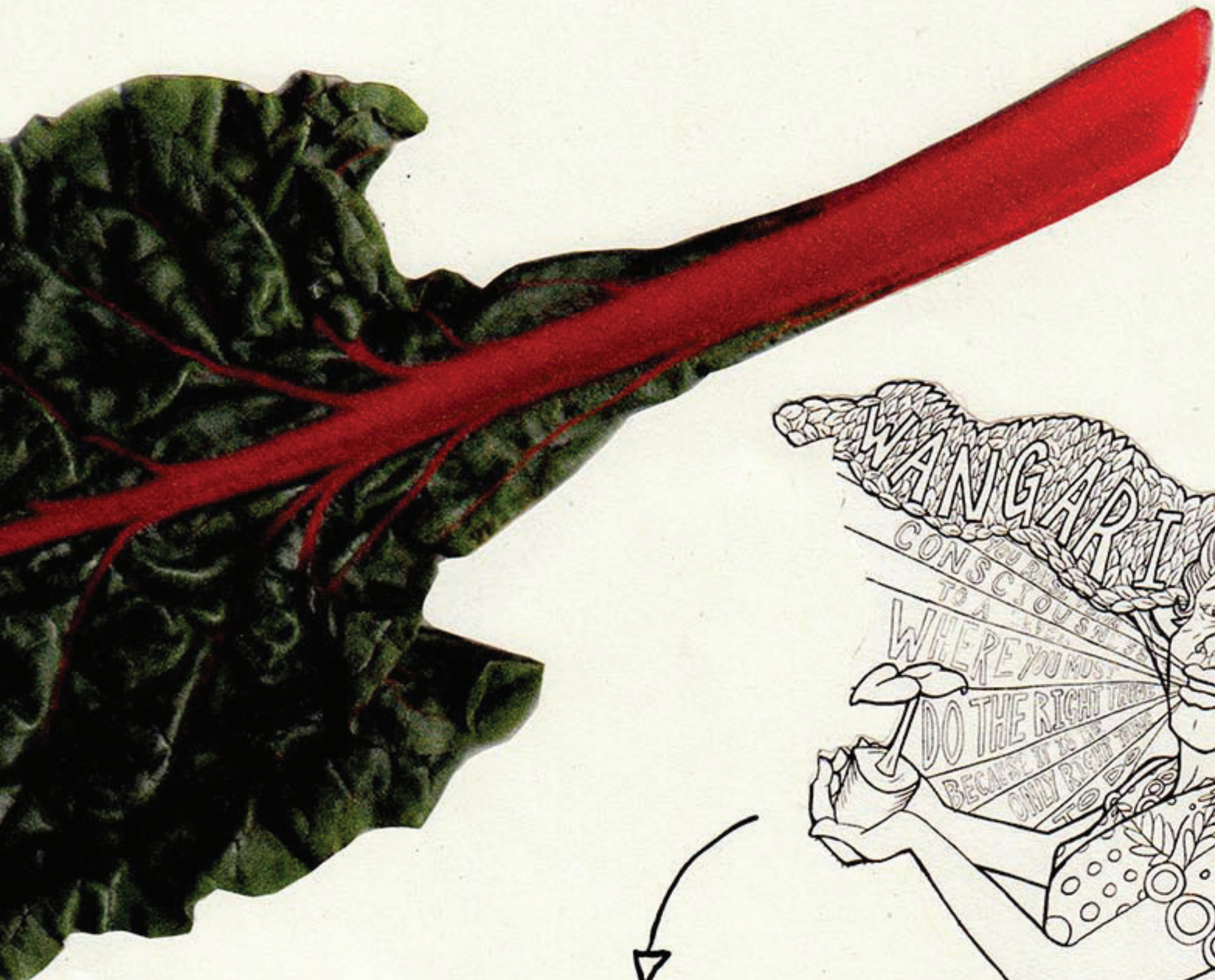

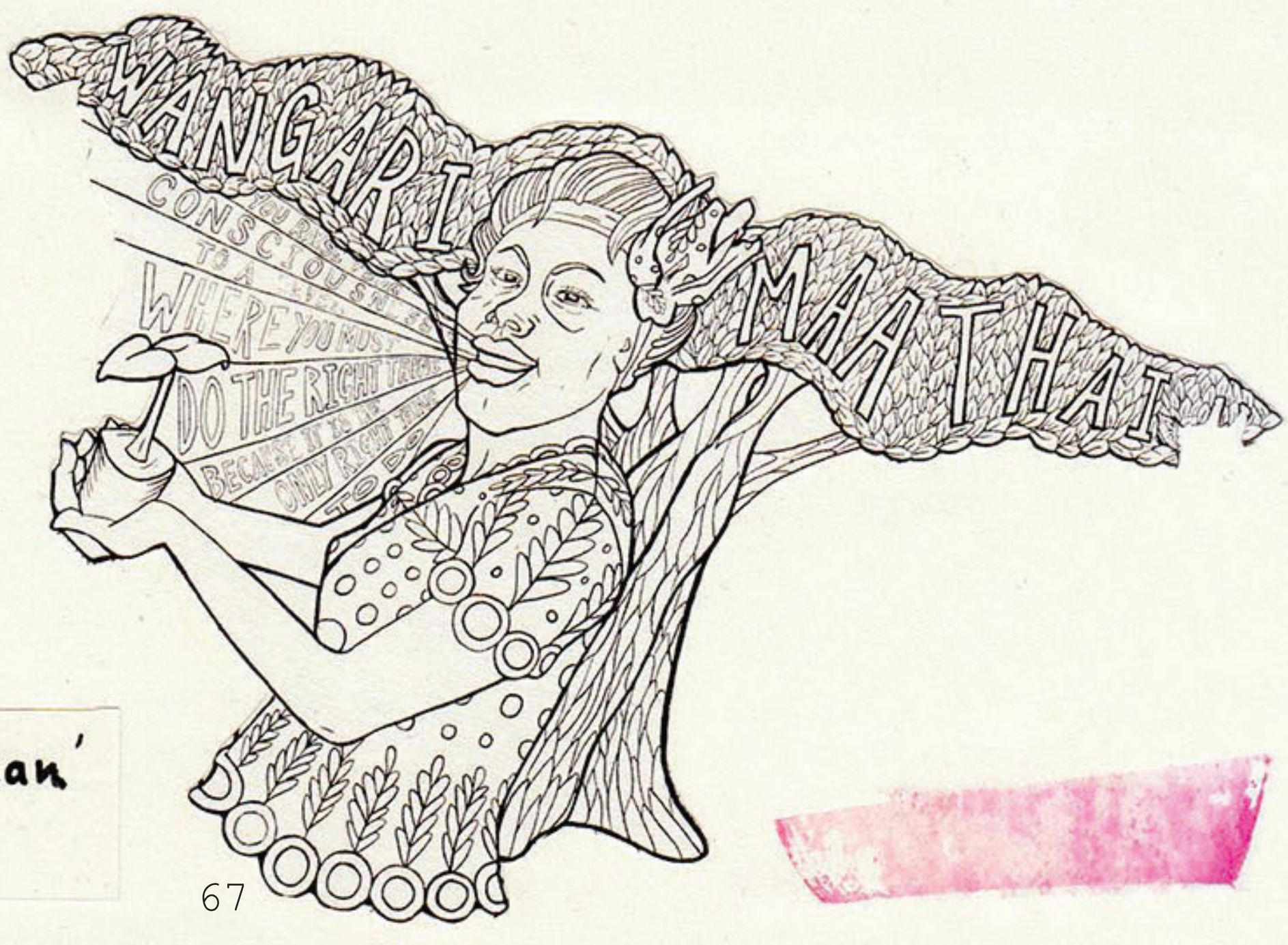

Image from 'Color of Woman' by Sarah van Borek

The bean soup that saved us

Kenilworth CAN

- 500g red/kidney/black-eyed beans
- 1 large onion, chopped
- 3cm piece of ginger, finely chopped
- Fresh garlic
- 2 sticks celery, finely chopped
- 2 carrots, finely chopped
- 1 tin of tomatoes
- 500ml stock
- 4 large potatoes
- 1 red chili
- Lemon
- Fresh coriander
- Salt
- Pepper
- Cooking oil

For the stock (this can be made in bulk and frozen)

Save all your vegetable scraps: potato peels, onion skins, carrot tops and peels, celery leaves, pumpkin and butternut skins, beetroot leaves, etc.

Place in a heavy pot with plenty of water

Add 2 cloves of garlic, a bay leaf, pepper corns and salt

Bring to a boil

Turn down the heat and simmer for 1 hour

For the soup

Soak beans in plenty of water over night, then strain

On medium heat, gently fry the onions, carrots, celery and ginger until very soft

Add garlic, chili and beans and fry for 5 minutes

Add tinned tomatoes and stock, salt and pepper (to taste), and simmer for 15 minutes

Add the potatoes and continue cooking until potatoes and beans are soft.

Garnish with coriander and lemon juice.

By Manya van Ryneveld and Tommaso Cosentino

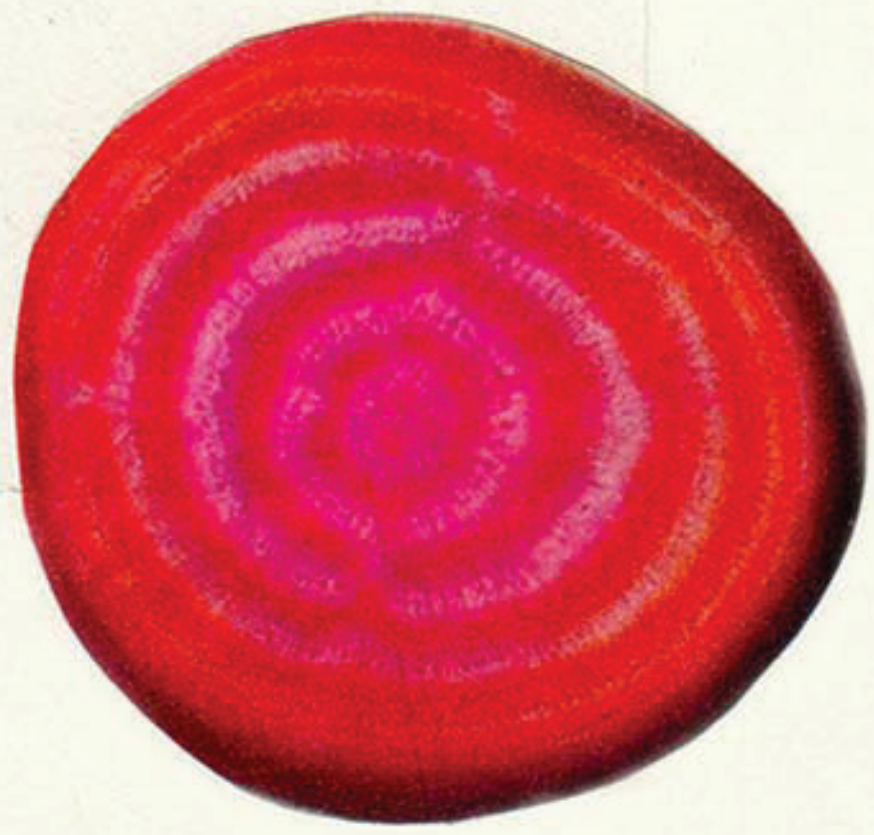

During the hard lockdown, Jasmina Hendriks stopped making Koesisters and started making masks.

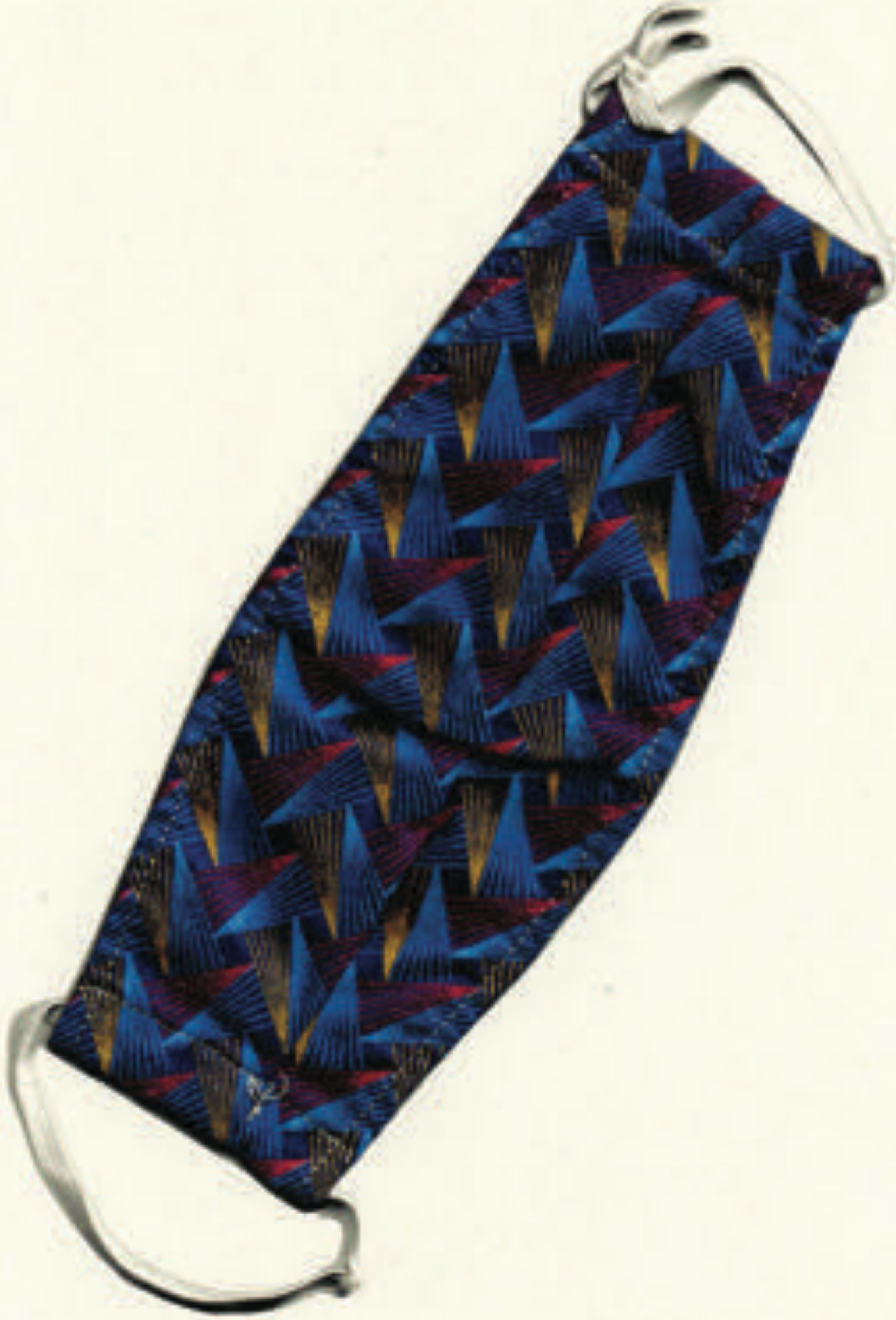

Within the first few days of masks being made mandatory she had made (at least) 500 and these were distributed for free through the CANs

Streets of care

Mowbray and Rosebank CAN

Lea Lukoji was born in 2005 in Cape Town (her family is originally from Congo). She lives with her mom (Emily), and younger brother (Exauce) in a small 2-bedroom apartment that has no garden. She is in Grade 9.

Marlise Richter was born in 1976 in Pretoria, South Africa. She lives with her partner (Marc), her two-and-half year-old daughter (Linda), three dogs (Knibbel, Basil and Snoetie) and one cat (Freanie) in a house with a small garden. She is a contract researcher

Everyone has dealt with the Covid-19 pandemic in different ways. It has affected every tiny aspect of people's lives, shaken up 2020, and thrown it about in a crazy and deeply disquieting manner.

The Mowbray Rosebank CAN mapped their neighbourhood, putting together 'Streets of Care' and inviting all residents to join a street WhatsApp group. The aim was to get know one another online, during this time of physical distance, and support the broader community through the work of the CAN.

This conversation captures the reality of two people living in the same neighbourhood, with different but connected experiences of South Africa's lockdown period. Through participation in their local CAN, the power of local, neighbourhood-level connection and solidarity emerged.

What was the hardest part for you during Level 5 and Level 4 of Lockdown (the most severe parts of the Lockdown)?

Lea: The hardest part was the fear and anxiety in my life which was leading to depression. The separation between us and my father was really hard – he was stuck in the Congo because the borders were closed.

Marlise: Yoh, it was a difficult time! For me the most challenging part was the isolation and being confined at home, and not knowing whether South Africa was going to survive the pandemic.

What was hardest for your family?

Lea: Our home's income comes from my dad's trading business between South Africa and the Congo. With the borders closed, there was no business. This meant that there was sometimes no food in our house, and my mom developed high blood pressure and was sick. There was too much stress and anxiety in our house.

There wasn't South African government support for my family, my mom applied twice to the Covid-19 relief grant but she did not receive any money. She thinks it is perhaps because she is a foreigner. Our main support came through the neighbourhood CAN.

Marlise: That sounds immensely difficult, Lea.

Our family was extremely fortunate that no-one lost their jobs and that we had a regular income. The contract work I do, continued throughout the Lockdown.

My broader family lives in different places - Pretoria, Johannesburg and London - and everyone was extremely worried about our older family members who had various risk factors that would make things very bad for them if they were to become infected. I really struggled with not being able to see my family.

What did your family battle with the most during this time? What helped?

Lea: My uncle in Lubumbashi passed away in June. It was very tough. We had to arrange the funeral from afar and because Congo was also in lockdown and all my relatives were in Kinshasa, it was immensely problematic.

At our house, we sometimes didn’t have food to eat and what really helped was the CAN monthly vouchers. It bought goods to our table. Our lovely neighbours have also been helping us with food and groceries.

Marlise: I am so sorry about your uncle. I remember that time well and how hard it was for your family.

I found the end of March to July the most difficult time. It was when anxiety and uncertainty levels in the country were the highest and the lockdown regulations the most severe (and some made no sense at all). Our family had to balance full-time childcare, as our daughter’s day care centre closed while “normal work” obligations and deadlines, household chores, together with advocacy and the work of Cape Town Together and our CAN, all carried on.

It certainly helped to be part of the CAN and the Cape Town Together network and to feel as if we were doing something constructively about the pandemic.

How did your broader family members experience Covid-19 - those in South Africa, and those elsewhere?

Lea: It impacted badly on my broader family members. There was fear and many lost their jobs here in South Africa, Congo and other countries where my family members live.

Marlise: My mom lives alone and I worried a lot about her. My brother and his family in London were caught up in the middle of the crisis in the United Kingdom and my sister-in-law works as a doctor for the National Health Service there and would likely be exposed to the virus during her work. My mother got Covid-19 during a card game with friends in October and she was very fortunate to only have very mild symptoms and recover completely. We were all immensely relieved.

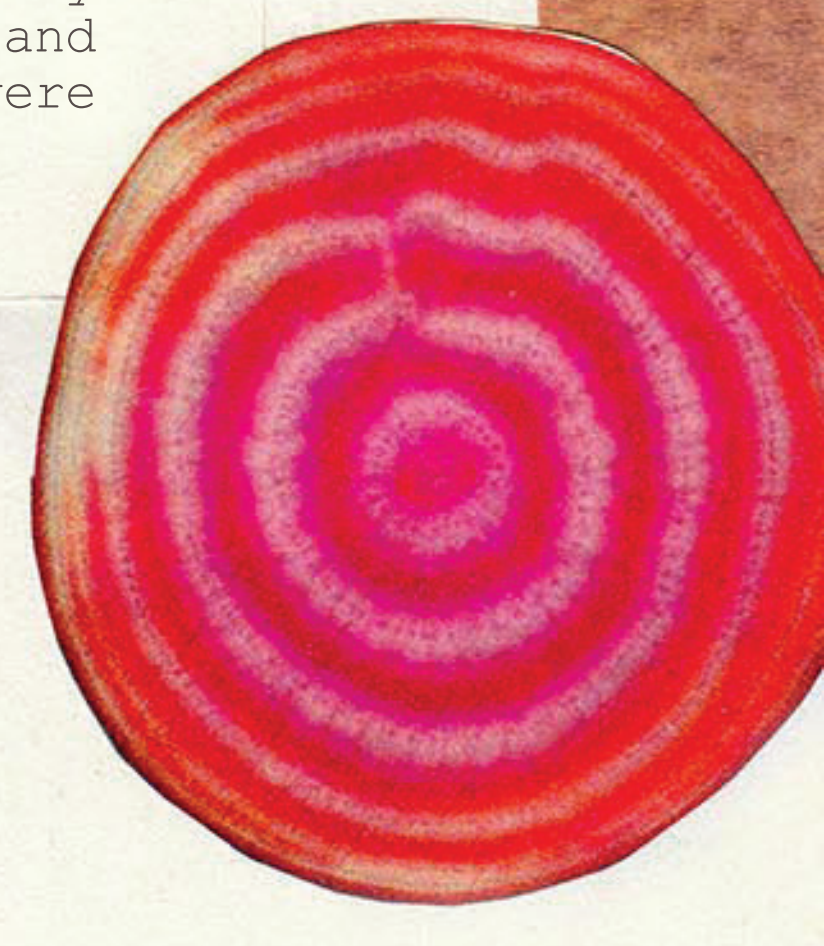

How did your neighbours experience lockdown?

Lea: There was a lot of fear and stress and people's way of living had to change a lot. If we had food, we would share it with neighbours as they were hungry too and we would just have to see what tomorrow would bring.

Marlise: Our street had a WhatsApp group and it was set up to help neighbours check-in on each other, and to help those in particular need. Many people in our area lost their jobs and had a very hard time paying normal expenses like rent and electricity, and looking after their families. Our CAN set up a Relief Fund that fundraised money from those community members who could provide support, and the CAN then linked that support to people who were battling financially. The CAN set up a group of volunteer callers who could check in often with families who applied to the Relief Fund to find out how they were coping, and to provide support. The Relief Fund also supported a community kitchen with our paired CAN in Montrose Park, Mitchells Plain. It was a difficult time, but it also pushed us to get to know and share with our neighbours.

What did you enjoy about the lockdown, and is there anything specific that you learnt?

Lea: What I enjoyed from the lockdown was that I've learned a new skill, came out a new person and met new people that made a big impact on my life. Through the CAN WhatsApp Group, I joined the neighbourhood's Mask Brigade. The Mask Brigade was made up of 25 volunteer who sewed masks for communities in need of them. My mom and I both sewed masks and one of the Mask Brigade organisers started involving me in making Finger Puppets for Red Cross hospital patients. I sewed so many and included inspirational messages to the children to help them keep their spirits up. People really liked my finger puppets and RX Radio Station at the hospital even interviewed me about it. I also started selling finger puppets at a market stall at our local neighbourhood market at Alma Café.

I learnt at school that when you volunteer, it reduces stress and anxiety, gives you joy, and you vibrate on another level.

Marlise: I love those Finger Puppets - you also gave my daughter one!

It is hard thinking about lockdown in terms of enjoyment, but I did appreciate that the streets were without cars and that people asked about others' well-being. I learnt how connected our neighbourhood is, and what generous, warm and empathetic people live in our community.

By Lea Lukoji and Marlise Richter

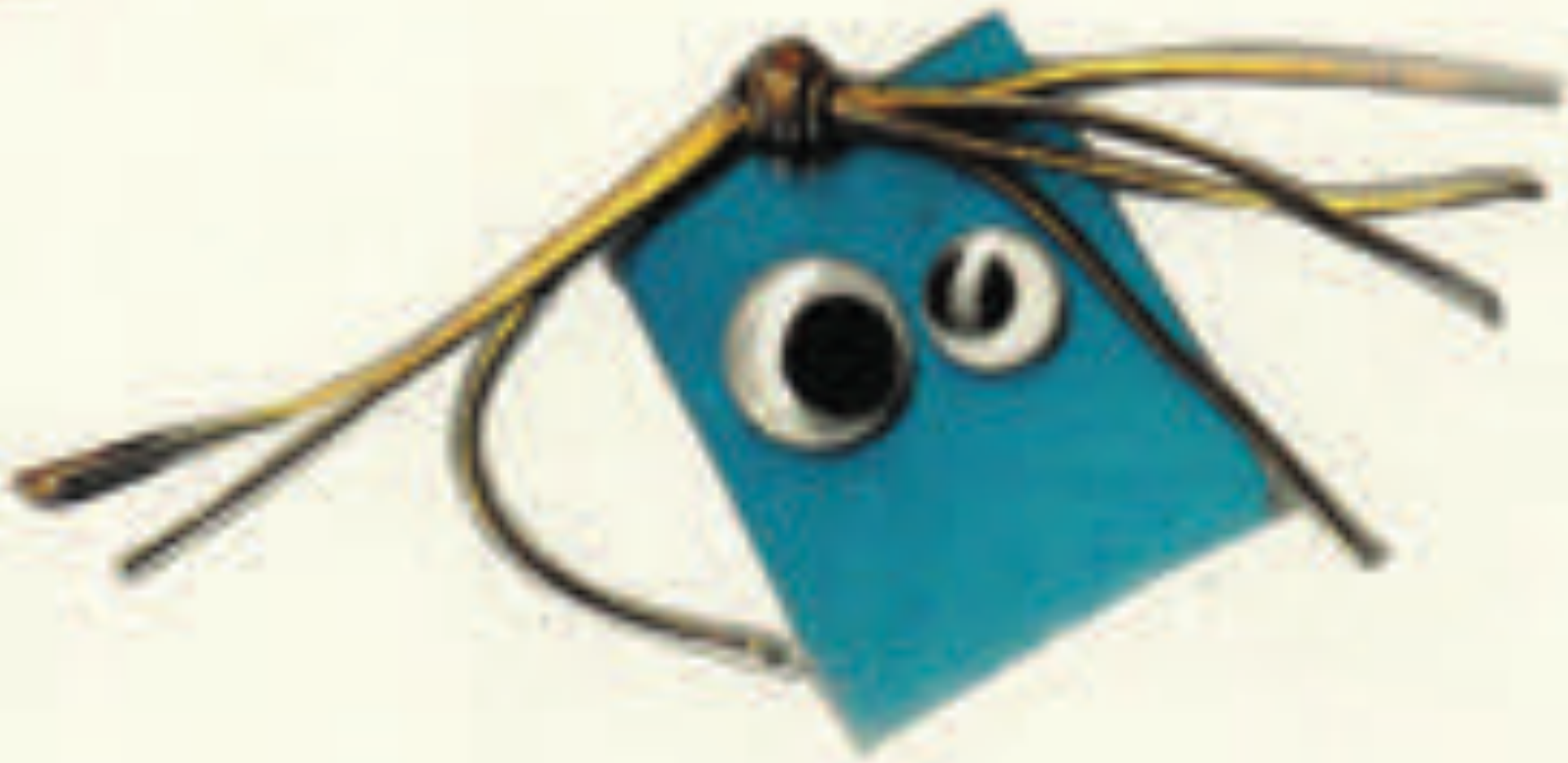

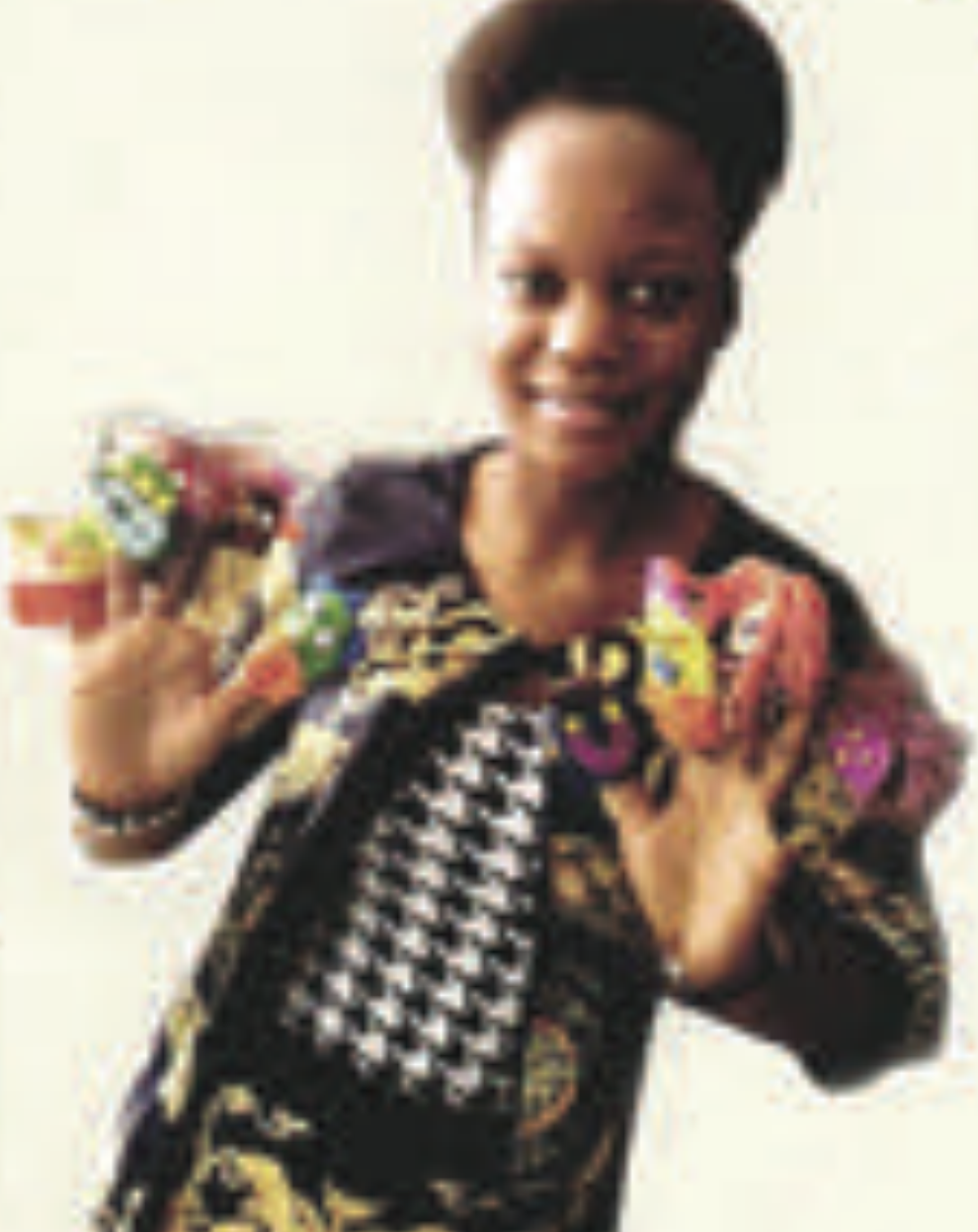

- Lea Lukoji
Mowbray-Rosebank CAN

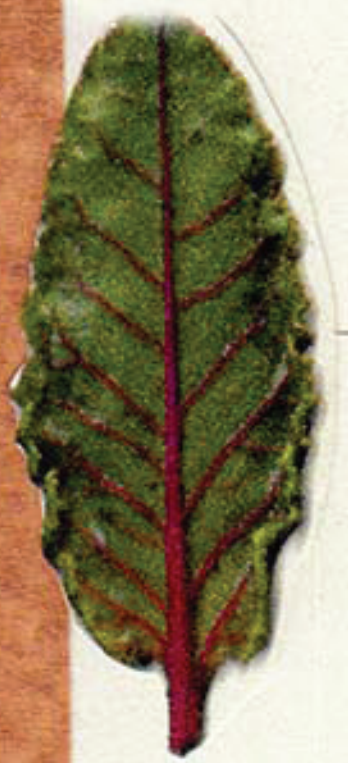

Borscht

Newlands CAN

Borscht is a Ukranian soup made from beetroots, potatoes, beans, carrots and onions, and flavoured with dill, garlic and vinegar.

Borscht has numerous variations. You can experiment with vegetables and choose to grate or to chop them. The best vegan recipe includes porcini mushrooms from Newlands or Cecilia forest.

The classic borscht recipe usually refers to red borscht and includes beetroots, beef and cabbage. It is quite dense, and the ingredients are usually largely-chopped. The classic recipe calls for serving borscht with sour cream - and pampushky (white bread buns with garlic) but it's also amazing with rye or black bread.

Borscht is always tastier on the second day.

- 3 large beetroots
- 300-400g beef (you can also use any other meat, or use porcini mushrooms for a vegan soup)
- 1 kg cabbage
- 3-4 potatoes
- 1-2 carrots
- 1 onion
- 2 bay leaves
- 2l water
- 3 cloves of garlic, diced
- 1tsp olive oil
- 4 tbsp tomato paste (or diced canned tomatoes)
- 1 can red kidney beans
- Salt and pepper to taste

First, prepare the broth. Wash the meat, put it in a pot and pour in cold water. When the beef boils, remove the foam and cook the broth for another 20 minutes

Peel beetroot, grate on a large grater and fry in oil. Add a little meat broth and stew on a low heat

Finely chop onions and fry in oil until golden colour. Add the carrots, pour some more broth and stew for another 3-5 minutes. (If you are short on time, you can just put all vegetables directly in the pot when the broth is ready.)

Peel the potatoes, cut into cubes, add to the broth and wait until it boils

Put the shredded cabbage in the broth and cook for 15 minutes more. Add salt, pepper, tomato paste and kidney beans, stewed beets, herbs and bay leaf

Cover the pan with a lid and cook for another 3-5 minutes. Remove from the heat and let it steep

To serve sprinkle with some dill, add a dollop of sour cream and serve with black/ rye bread

By Dzvinka Kachur

19th November 2020

Dear Cape Town Together

I am Chevano Frans, founder of Chevs Kitchen and have started Chev's kitchen as a direct result of lockdown.

I am 21 years old and am residing in Kewtown Athlone where I feed up to 150 people per day.

My most special memory feeding is a story of how one of my neighbours, an elderly lady of 84 years old, told me that she is proud of me and what I am doing and that she is praying for me.

This was very special to me as I grew up in front of this woman and now I have the ability to feed her and to be a blessing to her.

I would like to share this story with you and with the greater Cape Town community.

Please find attached a picture that I would like to share as part of my story.

Regards
Chevano Frans (Bridgetown, Silvertown, Kewtown, Parktown) CAN)

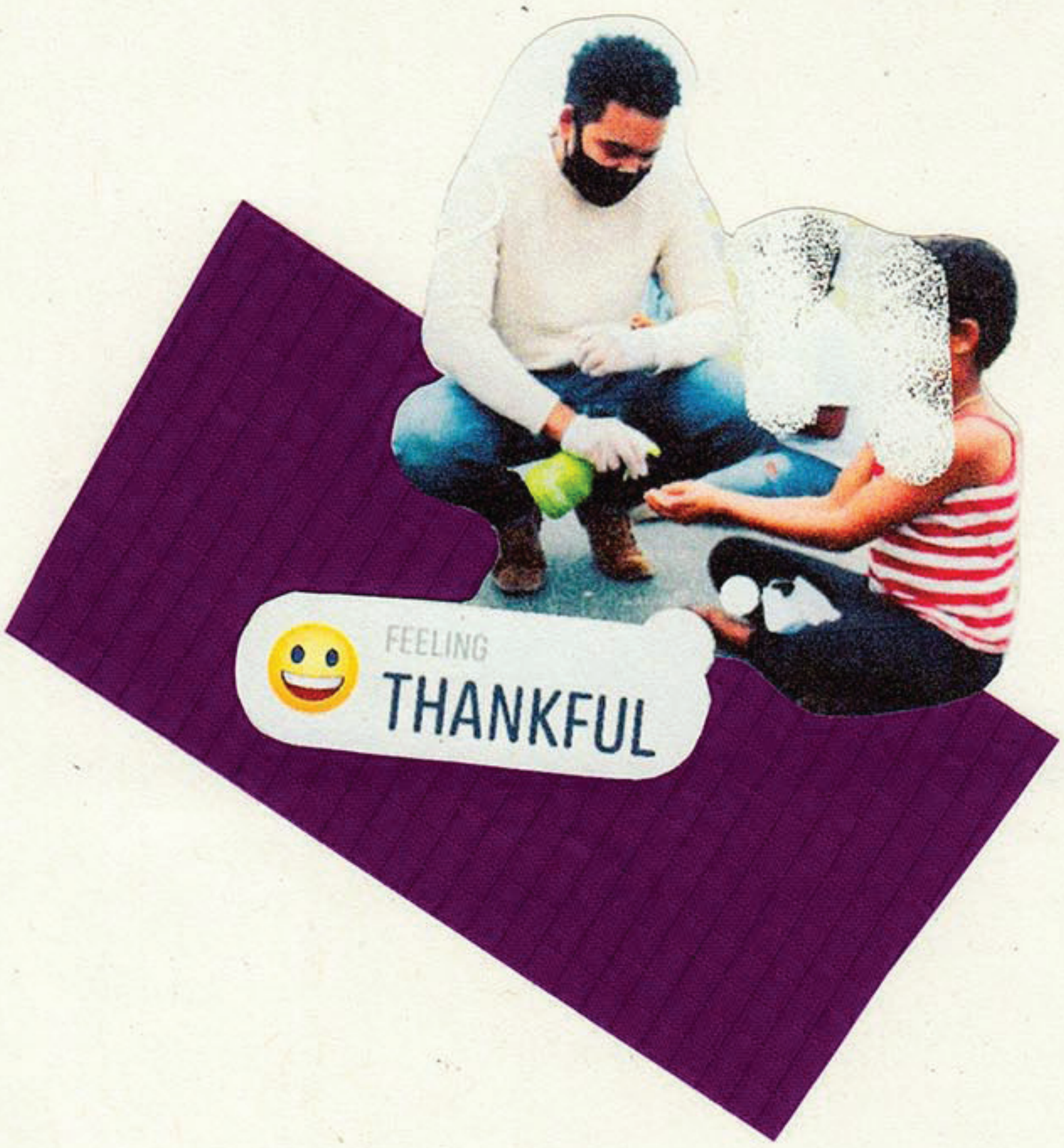

Fighting for identity

Khikhi CAN

Khikhi is an informal settlement at the far end of Gugulethu. Khikhi Community Action Network was established in early April 2020 by six Khikhi residents whose vision was to transform people's lives by creating better living situations for the poor in informal settlements during the COVID-19 pandemic. Khikhi consists mostly of hostels and shacks and is, in that way, quite different from the rest of Gugulethu. For this reason, we thought it was important to have our own CAN, distinct from the Gugulethu CAN, as we have very different stories to share and challenges to face.

In the '70s, under apartheid, the hostels in Khikhi were for working men. Three men would share each room. Each hostel had 6 rooms, 1 kitchen, 1 shower, and 1 toilet. The men were not allowed to bring their wives or families to the hostel. When the men brought their families from rural areas to the city, to be close to their wives and allow their children to go to school nearby, they had to find somewhere else to stay - this is how the informal part of Khikhi was born. It includes hostel backyarders, Polar Park, Water Front, Ladies Park and Lotus park.

Practicing social distancing, regular handwashing, and sanitising surfaces is nearly impossible in Khikhi. Khikhi residents share everything, including water taps and toilets. There is also a problem with excess drainage water that runs through the informal settlement, particularly in Polar Park. During the hard lockdown, Khikhi CAN members were working on the ground to prevent the spread of Covid-19. We hung soap and water next to each toilet for people to wash their hands before and after use and sanitised the toilet seats with bleach.

During our work, we found that many people in Khikhi don't have ID documents, and that limits the chances of them being employed and the children getting social grants. We took it into our own hands as a network to dig deeper and find out how we could help as Khikhi CAN. In September, when case numbers started slowing down, we started an initiative to get identity documents for Khikhi residents. We approached the manager at Home Affairs, told him we were from Khikhi CAN and he was happy to assist. It was powerful to use the network to build a relationship with Home Affairs. He is still helping us with this project to date.

We are now in the process of getting IDs for the people that lived mostly their entire lives without being recognised as South Africans. These people had lost hope because they did not know what to do to get ID books. We were given documents by home affairs on what to do in such a situation and worked with residents to start the process of 'late regis-tration'. Now they are recognised as South Africans, and no longer have to live life without an identity.

The next step, after getting registered with Home Affairs, is to approach SASSA to make a plan for children who do not get the grants they are entitled to because they don't have a birth certificate. SASSA policy clearly states that a child should get a grant while the mother is awaiting ID from Home Affairs. However, while engaging with Home Affairs to get registered was easy, we have not been able to get an appointment with SASSA. This is the next challenge we are taking on. We are determined to continue to help the residents in our community get the social support they are entitled to.

By Ncediswa Notywala

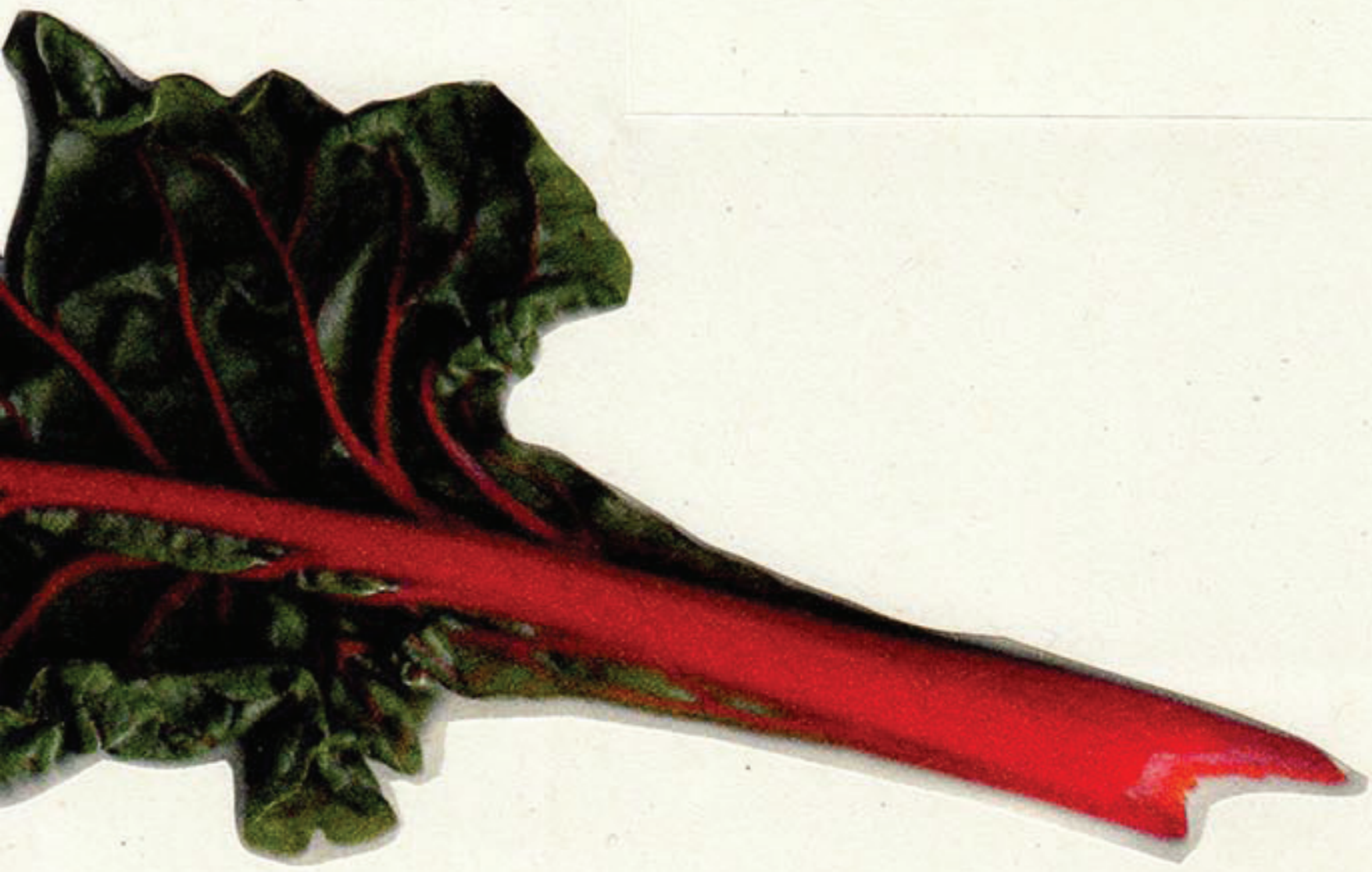

Ubuhle Besizwe Soup

Site B CAN

Ubuhle Besizwe means 'The Beauty of the Nation', and is an NPO operating from Site B, Khayelitsha, with a focus on providing education, skills, welfare and a sense of belonging to disadvantaged primary school children in the informal settlement.

The NPO's founder, Linda Mafuna started the NPO to address the educational needs of children in her community, who she observed were falling behind in their education and, for various reasons, not receiving the help they needed from caregivers or their school educators to catch up.

During the COVID-19 national lockdown, Linda and a team of volunteers turned their attention to feeding children and families in their community. They have provided cooked meals to over 150 families a day – roughly 200 adults and children – since the end of April 2020, using financial and food donations from concerned Capetonians, as well as from their own pockets.

The following is a family recipe Linda and her team have been using to make soup for her community.

- Samp, 10kg
- Beans(brown), 5kg
- 2kg onions, or green onion leaves
- 5kg potatoes
- 3kg peppers
- 5kg carrots
- 24 cubes beef stock
- 5 cups (1litre) cooking oil
- 10tbsp salt
- 200g Robertson spices
- 200g Yellow Rajah

Wash samp with cold/lukewarm water

Chop the onions, potatoes, peppers and carrots into small pieces.

Fill a pot with water and put on the stove to boil. When boiling, add the samp with brown beans.

Leave the samp and beans to boil and simmer, with the pot lid tilted to let the steam escape, for 45-60 minutes or until the samp and beans are half cooked.

Rinse the samp and brown beans until the water runs clear.

Add your chopped potatoes and carrots to the samp and beans. Add enough hot water to cover samp, beans and other veggies.

Add the salt, stock and spices and allow to boil.

Remove from heat when the soup ingredients are soft and the gravy has thickened.

Serve with bread or magwinya (vetkoek).

By Linda Mafuna, Ubuhle Besizwe Soup Kitchen, Khayelitsha

Planting seeds in the Kipling Street Food Garden

Salt River CAN

Happiness is found in homemade

Preserve in the garden
Turn to love
On the table

Capture the joy
Of sharing
Food
With your nearest and dearest
Family and friends

Soul food in need
a garden indeed

Let's stir up a bowl of love

During the Covid-19 lockdown many people in Salt River found themselves unemployed, and because of this there was an immediate need to start growing food. But, rooted in relationships both old and new, our garden was definitely about much more than food.

Pandemic times are tough, and 2020 has been a difficult year, but in between planting dhania and harvesting spinach we shared stories about the past while figuring out how to support each other right now. What sprouted was the Kipling Street Food Garden – a beautiful collaboration between a growing number of neighbours in the area, the Salt River CAN, and Neighbourhood Gardens.

In June, as food gardens started popping up across the network of CANs in Cape Town, we got excited about the possibility of setting up something similar. Soon we were collecting wood from the dump, clearing the alleyway alongside the sports field, and transforming it into a thriving veggie garden.

Spending time in the food garden is good for our health. Being outside, having our hands in the soil and being physically active is a great way to improve physical and mental wellbeing in these trying times of the pandemic. We learnt from one another about how to grow vegetables, what herbs to plant, and the health benefits of all the different veggies we were growing. The food we grow has not been treated with harmful pesticides and preservatives like produce found in a grocery store.

Growing food in community gardens is also good for the planet. Firstly, the food we grow does not need to be harvested by big tractors that create harmful emissions. Also, our locally grown veggies do not need to be shipped or flown across the world to be eaten somewhere far away, and so it is much better for the environment.

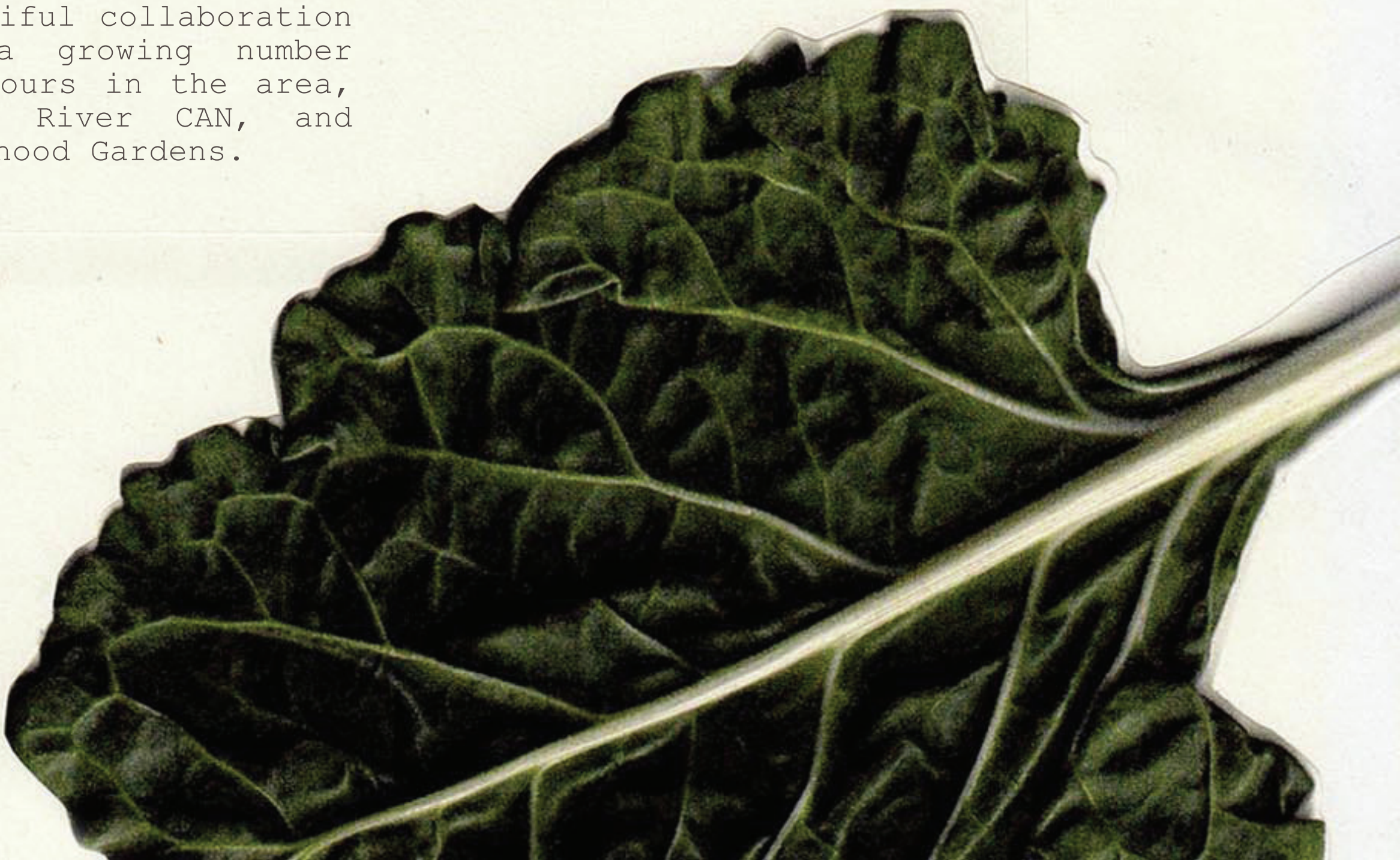

Food gardens + insect highways = growing healthy communities

Because we don't use pesticides, we are also helping to create healthy environments for the insects that are an important part of our eco-system. Without bugs, bees, birds and all the rest we cannot sustain life on our planet. After all, insects are the main pollinators of our own food crops. Bees and insects need plants to survive. In this way our food garden (or even planting a few pot plants on your windowsill) is contributing to a healthy ecosystem in the city.

Once you learn how to grow some of your own vegetables, you need to spend less money on food in the stores. Ron Finley (the Gangster Gardner from Los Angeles, USA) put it well when he said, "Growing your own food is like growing you own money".

Most importantly, the garden has created a space for us to come together as a Salt River community. One positive thing about lockdown was that it slowed our pace of life, so that we had time to spend together, planting seeds and gardening. The garden has even allowed us to reconnect with the childhood friends we grew up with. It has been an outdoor Covid-safe place for the coming together of neighbours, building community and being able to spend time with our beloved plants.

Community gardens transform unused land into positive spaces, where people feel safe, where new relationships are built, and old friendships solidified. They are places for generating a sense of community, as well as growing food. Already we have a very beautiful and thriving garden providing food for a number of families, and creating a positive space for connection in our neighbourhood. Our vision is for the Kipling Street Food Garden to be the start of a whole network of gardens to grow healthy food that is free for everybody, develop insect highways for insects and birds, and create spaces where neighbours connect.

By Marius Zenker, Zainap Salie and Kulsum Viljoen

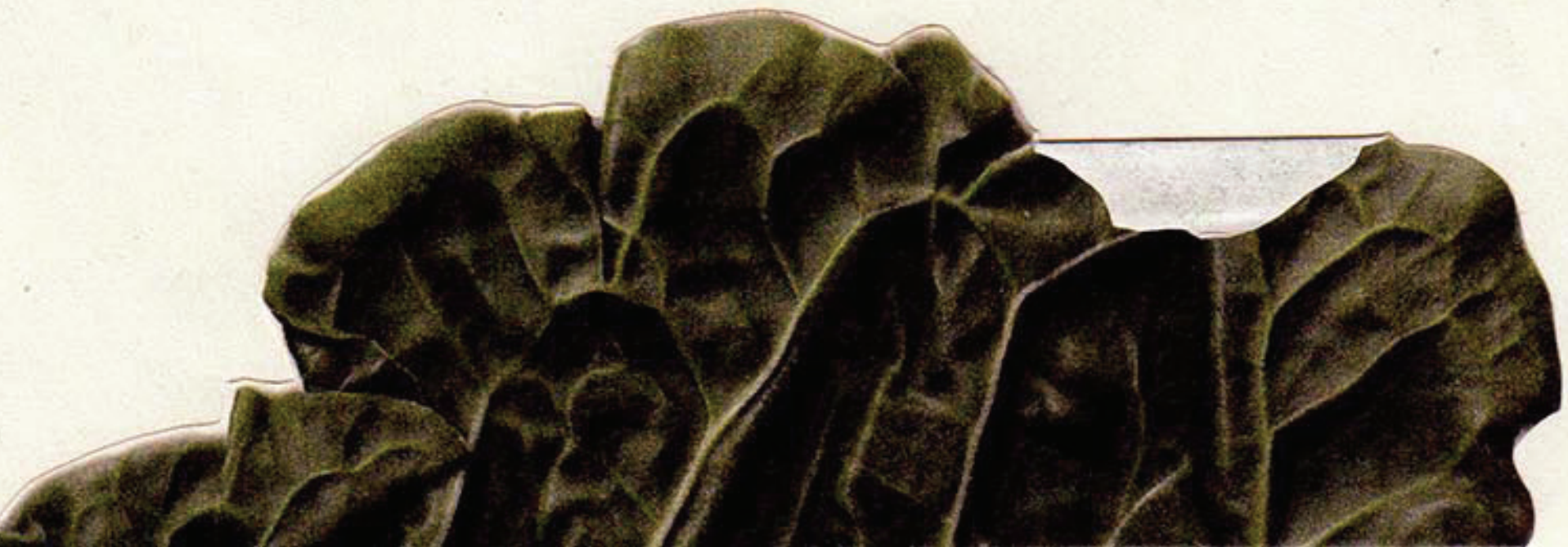

Bonteheuwel community group takes flack from DA councillor

Bonteheuwel CAN

First published by Daily Maverick – 11 August '20

The Bonteheuwel community centre was always a vibrant home to community groups – until it fell into disrepair during lockdown. Now, combined efforts to revive the centre as a Covid-19 self-isolation site are finally bearing fruit. But not everyone is happy.

The multipurpose community centre (MPC) in Bonteheuwel is a government-owned facility in the heart of the working-class suburb. It was always a vibrant community hub hosting community meetings, weddings, karate and dance classes, church services and weekly Alcoholics Anonymous meetings.

However, when lockdown forced the centre to close, vandals moved in. Wiring was pulled out, doors were ripped from their hinges and windows were smashed. The outdoor space became overgrown with weeds and littered with household trash. No attempt was made to clean up or repair the damage. In this condition, the centre could no longer be used.

It was at this point that the Bonteheuwel-Joint Peace Forum Community Action Network (BH-JPF CAN) approached the centre's management for permission to restore the MPC and its grounds. The intention was to repurpose the facility as a community-run care centre for people suffering with Covid-19 who lacked the space to safely isolate at home.

The BH-JPF CAN is a community initiative that for the past five months has been providing vital Covid-19 relief in a suburb that is badly affected by poverty and crime.

The CAN is not a formal organisation, but rather an inclusive collective of residents tackling local issues together. As a part of the Cape Town Together Network, the BH-JPF CAN is partnered with the Rondebosch CAN which supports their initiatives wherever possible. The BH-JPF CAN has been a symbol of hope to those struggling with the social and economic consequences of the pandemic. Its efforts include maintaining a network of 46 community kitchens throughout the lockdown period.

The centre's manager told BH-JPF CAN that it would not be used for public or community events during lockdown, and was therefore available for use as a community care centre (CCC).

In May, an agreement was signed between the manager and BH-JPF CAN, in which a rental amount was stipulated. Shortly afterwards, on 24 June, the manager notified community groups to collect any property they'd left behind at the facility before lockdown, so that it could be used as an isolation space during the pandemic.

Over the past two months, the BH-JPF CAN, with the support of the Rondebosch CAN and members of other CANs across Cape Town, have begun restoring the MPC. They've been hard at work raising money for building supplies, imploring local construction and repair companies to donate their time and expertise, and calling on nearby CANs to join in the clean-up effort.

The hard work has paid off. Today, thanks to the BH-JPF CAN and others, the centre has been returned to the welcoming space it always was.

Vandals can no longer gain access to the premises. The electrics and plumbing are being repaired, and the community vegetable garden is thriving once again. Broken windows have been replaced, ceiling boards repaired, and the hall, kitchen, and security area repainted.

Now, just days away from welcoming the first 'resident' to the Bonteheuwel community care centre, the BH-JPF CAN has hit a stumbling block.

The group has angered DA ward councillor, Angus McKenzie. He says the CAN's use of the centre is illegal and that it's "an ANC project".

A series of posts on Facebook and circulated on WhatsApp groups state that:

"The Rondebosch CAN/JPF attempted to hijack a government facility that does not belong to them, effectively leaving various other organisations, churches and sporting groups on the street."

This is a gross misrepresentation for a number of reasons, not least of which is the fact that none of the community groups was using the facility due to lockdown and the centre's state of disrepair. In addition, the facility has not been "hijacked" since the BH-JPF CAN has an agreement with the centre's manager and is paying a monthly rental.

The social media post also claims that the BH-JPF CAN is affiliated with the ANC. This is quite simply not true. Neither the BH-JPF CAN, nor the Rondebosch CAN, is politically affiliated. Cape Town Together is a network of self-organising CANs engaged in rapid, community-led responses to Covid-19: it is not a formal organisation and is not aligned to any political party.

The truth is that the Bonteheuwel community centre was always a vibrant home to many community groups and was only interrupted by Covid-19 and the lockdown. It became impossible to use the indoor facility for community activities, religious meetings, and recreational purposes.

In a time of crisis, such facilities must be used as part of a comprehensive, multi-sectoral Covid-19 response that meets the most pressing needs of local communities.

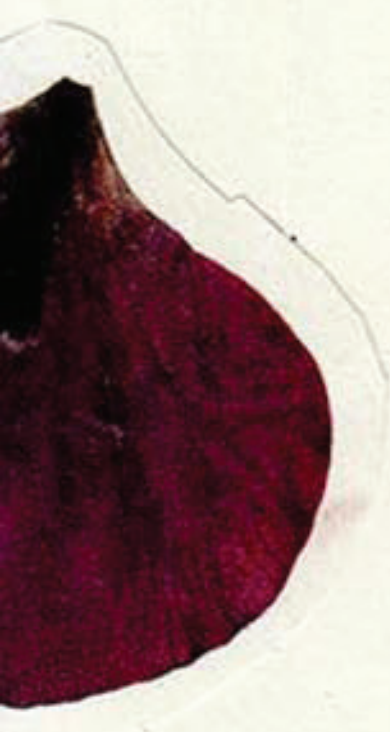

One of these urgent needs is a safe space near to people's homes, where loved ones can drop off food and clothes, and where Covid-19-positive folks can self-isolate while being cared for by those from their own community.

BH-JPF CAN is also part of the Cape Town Together Health CAN in which a group of medical doctors, public health specialists and CAN members are supporting CANs to set up community care centres for those needing a local space to self-isolate.

The model has been successfully implemented in Ocean View near Kommetjie, where the Ocean View CAN has established a community care centre with the support of local residents and health workers.

There, the centre provides "home-away-from-home" care to residents needing somewhere to safely self-isolate while recovering from Covid-19. One of their most recent residents was a woman who shared her home with 10 other family members, some of whom are high risk. She did not want to leave her neighbourhood for a far-off government quarantine facility, but was unable to self-isolate at home. At the Ocean View centre, she received care and support from local volunteers until she was no longer infectious.

The BH-JPF CAN is a well-respected community organisation within Bonteheuwel, and the work of the JPF is trusted by many. Since the negative social media messages emerged, statements of support for the BH-JPF CAN have flooded in from organisations including Bonteheuwel Churches in Action, Bonteheuwel Legacy Arts Cooperative, Apostolic Church of Christ, Bridgehouse, Bonteheuwel Crime Alerts, La-Bon Foundation, 3rd Bonteheuwel Scouts and Communitree SA.

This tension between ward councillor McKenzie and the CAN comes at a time when CANs across the city are exhausted from months of doing the work that should be done by elected officials.

What the CANs do is hard work; it is a daily toil of love, empathy, and radical generosity, largely from the side of women. Their efforts receive very little support and are often met by opposition from those who see community organising as a threat to their power.

By Nadia Mayman, Eleanor Whyle, Aimee Hare and Ghadija Hayat

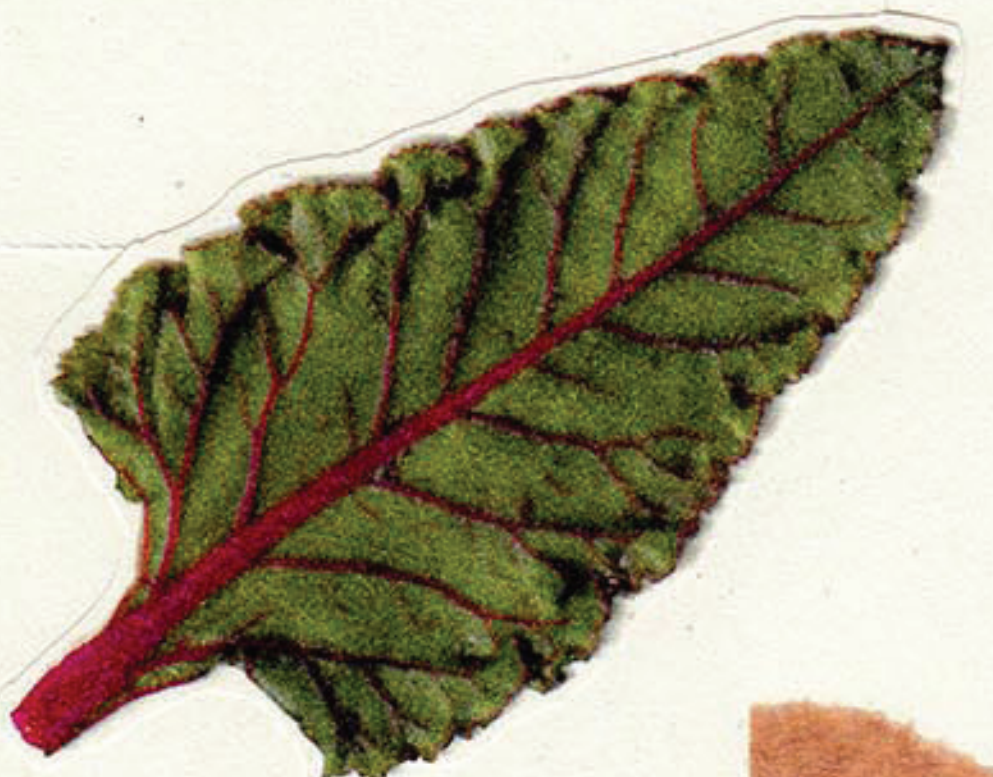

Chicken akni

Bonteheuwel CAN

- 15kg Chicken pieces (chicken breast is best, cut in half)
- 15kg medium potatoes, peeled (halved if big, whole if small)
- 15kg Tastic/Akila/Basmati Rice (soaked for 3 hours or overnight in advance)
- ½ bag large onions, finely chopped
- 500g margarine (marvelo)
- 500g garlic and ginger paste
- 2 cups curry leaves
- 6 bunches chopped fresh dhanya
- 500g course salt, to taste
- 500g Osmans Akni Masala
- 250g ground jeera
- 250g garam masala
- 50g chili powder (optional)
- 50g turmeric
- 2l cooking oil
- Handful bay leaves
- ½ cup cardamom pods, slit open
- 1 cup stick cinnamon
- ½ cup star aniseed
- 20 litres water

Wash the chicken pieces, and drain.

Marinade chicken with ½ the garlic and ginger paste, akni masala, jeera, garam masala, turmeric and ½ the salt. Mix thoroughly, ensuring that all the chicken pieces are covered in the marinade. Set aside for 1 hour.

In a 100-litre pot, pan-fry the chopped onions, remaining garlic and ginger paste, curry leaves, bay leaves, cardamom, cinnamon and star aniseed in 1litre of cooking oil. Braise onions until golden and translucent.

Add 2 bunches of chopped dhania.

Add potatoes and cook until the potatoes are half done. You may add 1 litre of boiling water to prevent burning the potatoes.

When the potatoes are half done, add the chicken. Simmer until chicken is cooked.

Drain the rice and add to the pot. Add 20 litres of water, the remaining oil, and the remaining salt. Allow to simmer until water is nearly evaporated.

Slice the margarine and place on top of rice with remainder of dhania. Close the pot and switch off the stove. Allow to stand for 1 hour. All water will be soaked up and food will be ready to dish.

All aknis and breyanis should be dished from the bottom up.

Variation: The chicken may be substituted with mutton pieces, soya mince, soya cubes (brown or white) and prepared in exactly the same manner.

You may also add, cooked lentils and/or mixed veg when using mince (soya or beef).

By Nadia Mayman

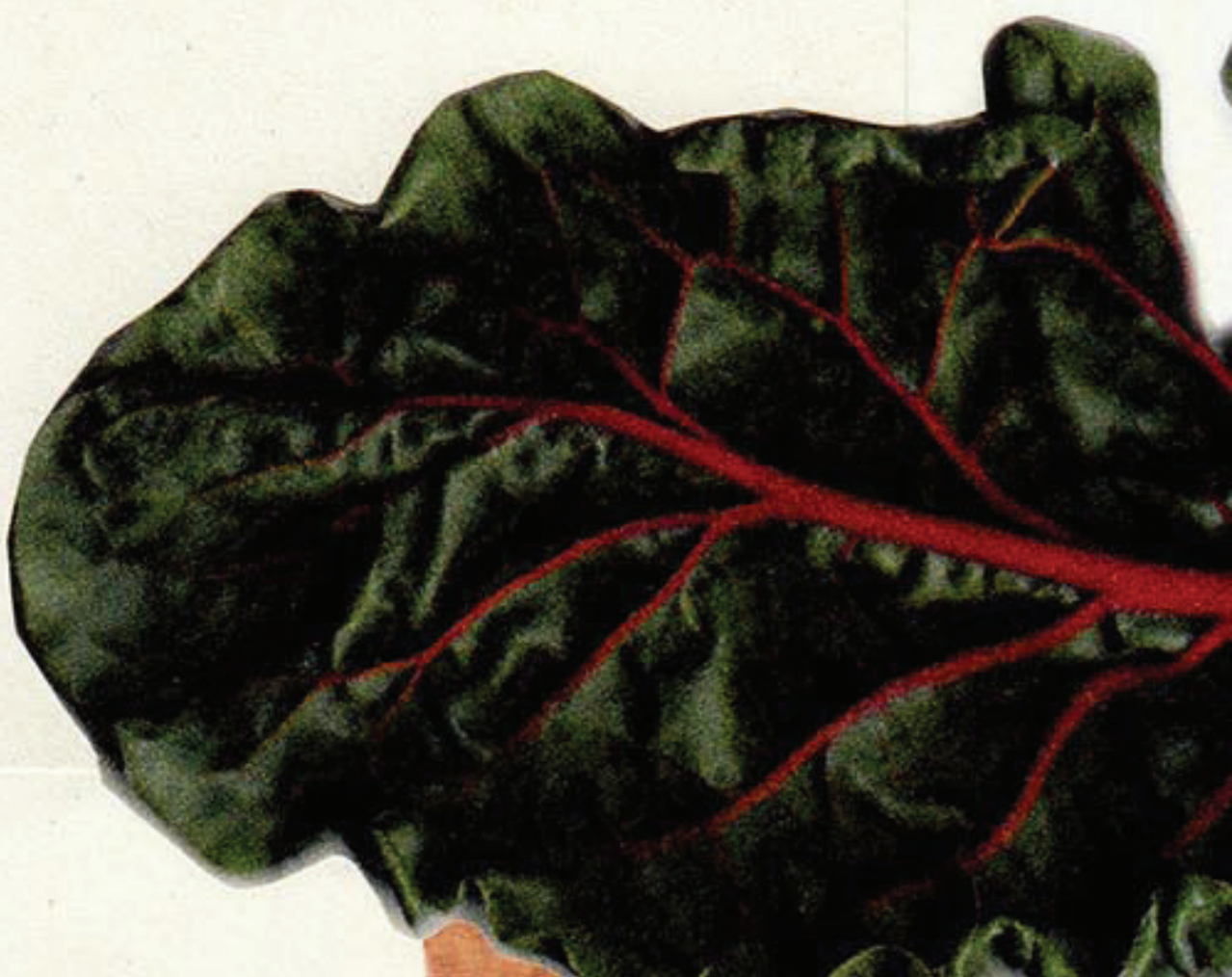

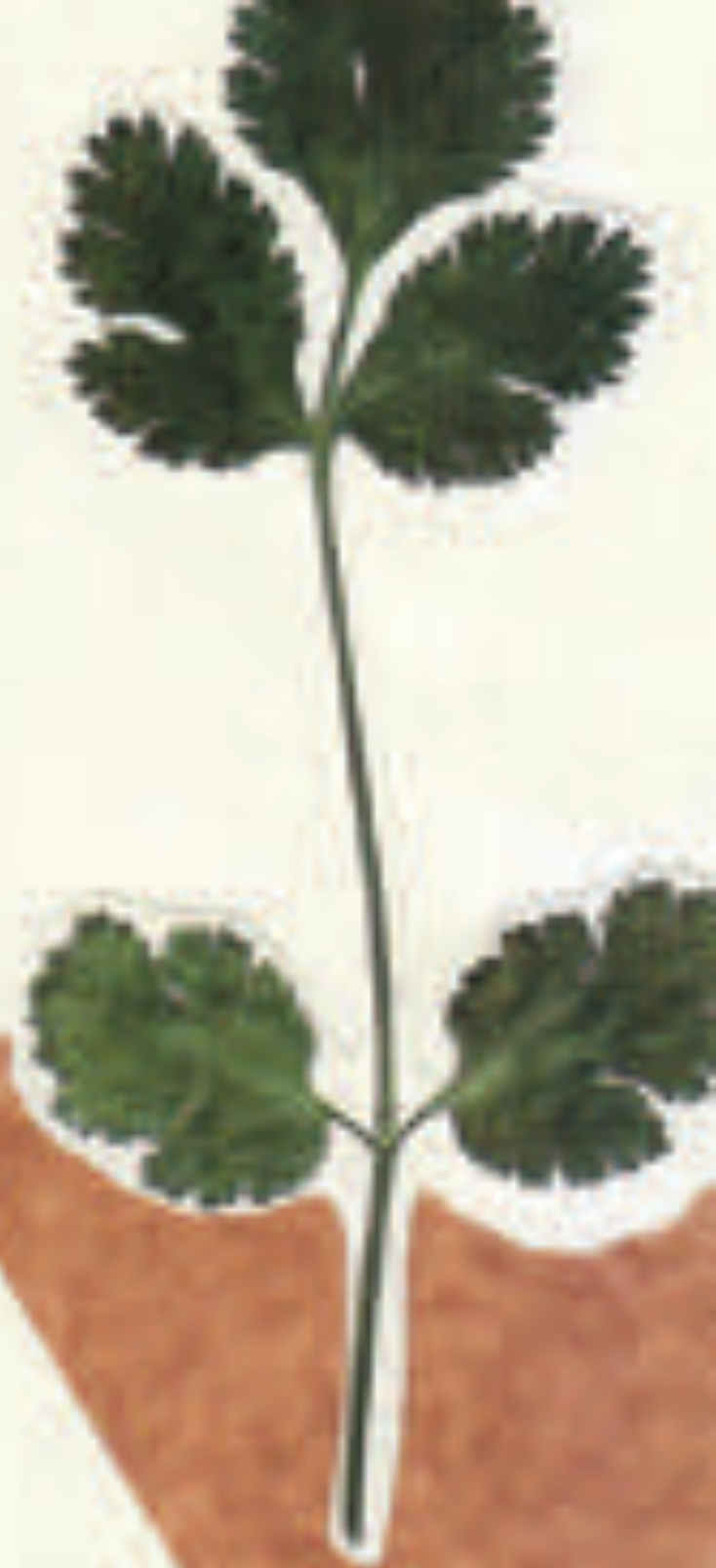

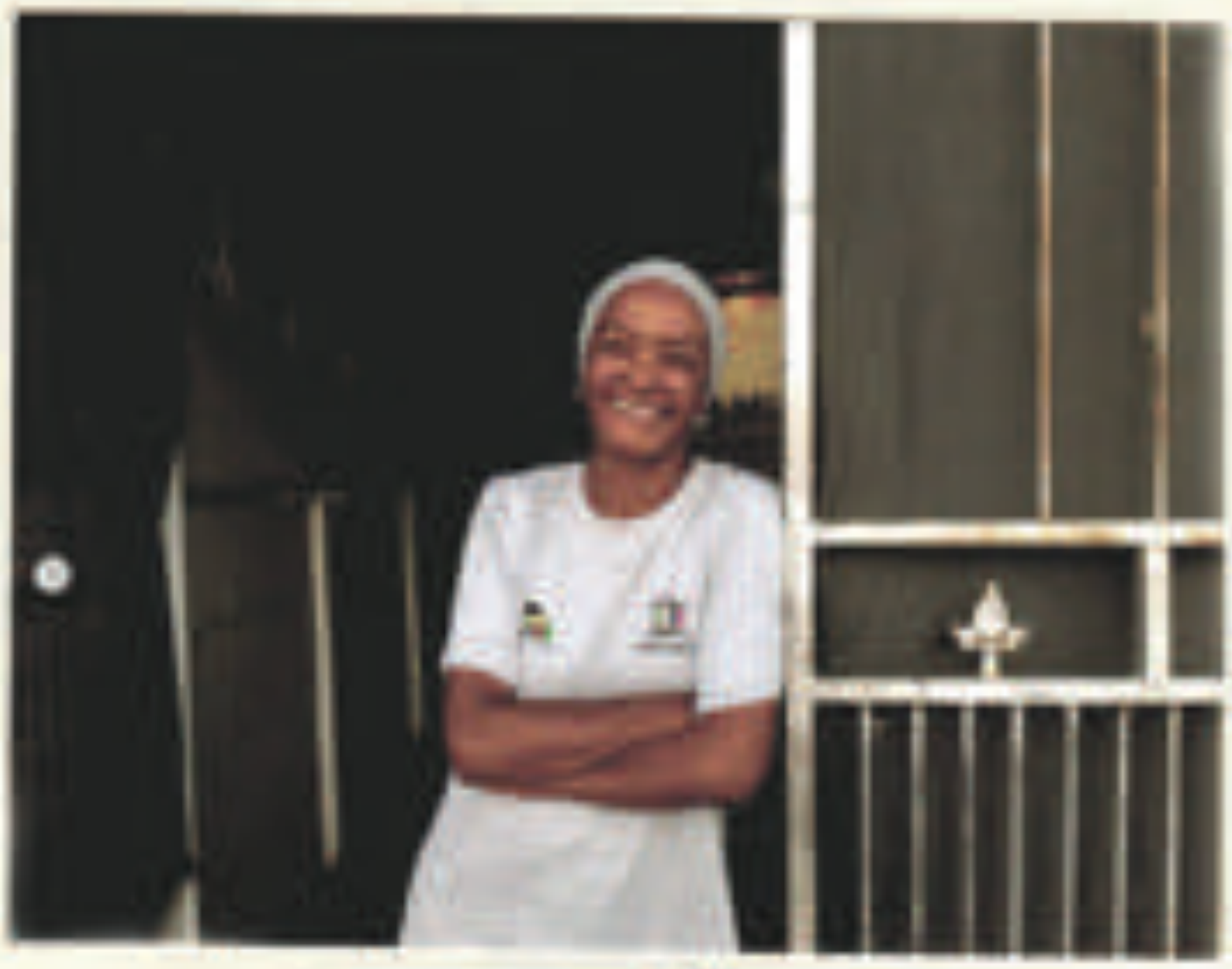

- Nadia Mayman
Bonteheuwel - JPF CAN

Lighting candles: You don’t have to put one out to light another

Pinelands CAN

On how everyone has something to offer

In our CAN, we all have different strengths. Annie is not a great admin person, she prefers caring for people and working with people. “And I am not afraid to ask for things, which is definitely a strength” she adds. But we also have great admin people in the CAN – a team of doers and drivers who are willing to put in the hours. Everyone has something to offer.

Alison’s background is in tourism, and she has experience running big teams in sales and marketing. “That’s where I learnt to be resourceful and to connect people to one another,” she says. But you don’t need a degree or ‘book knowledge’ to help people, you just need to have a heart for people.

On starting small and the CAN’s work with local homeless people

There are a couple of homeless people in our area who had been living here for years. When lockdown hit, Alison and her family started cooking for them. They cooked and handed out food every single day up until level one.

Eventually, when it was not possible to continue daily meals, we had a conversation with the homeless folks about what else we could do to support them. Kate, another CAN member, runs a project called the Avatar project, where people can earn some money by cleaning the canal that runs through Pinelands. So, we invited the homeless folk to be a part of that project. Sharing a meal and inviting them to contribute and be a part of a community project by helping to clean the canal, really opened up a dialogue. After two months, people started trusting us.

You have to start with getting to know the person and their circumstances. Some homeless folk don’t have an ID book and need support getting one. One person might have a family they can go back to, where for others their family is the reason they are on the street in the first place. Then, you have to wait for them to be ready.

There are also logistical hurdles that are hard to overcome if you are homeless. Once they say they are ready to go to the shelter, we take them to get a Covid screening and medical check-up, and then either we take them, or the city sends a vehicle, to take them to the shelter. So far, we have managed to help 6 people get off the streets.

On the power of networks

Pinelands CAN formed very early on, before lockdown even started. We were lucky we already had existing networks and relationships through Church, school and other social groups. But being able to call ourselves the Pinelands CAN really helped, because everyone knows what the CAN is. If you ask for donations or volunteers, but people aren't familiar with the organisation, they are a bit hesitant. But because the CAN was a recognised thing in Pinelands, people knew that they could trust it. When we need to call on people for support, we have this network ready, people are very happy to give.

The CANs really do capture the magic of collaboration. One person can have a heart for helping people, and can do their little thing, but when we collaborate, there are these synergies that are created that are incredibly powerful. It's network of trust, and the whole is more powerful than the sum of its parts

On self-organising and radical generosity

What is different about the CAN network is that we are civil society, so we are not bound by the rules and the red tape that would prevent us from helping. It's much easier to fundraise when you can say, 'I am part of a human network and all we want to do is help people'. There is no bureaucracy.

The CANs are united by a spirit of radical generosity. Within more organised structures, there is often some competitiveness and a hoarding mentality. But in the CANs it's like lighting candles, there is always more light, you don't have to put one out to light another.

By Alison Dixon and Annie Smith

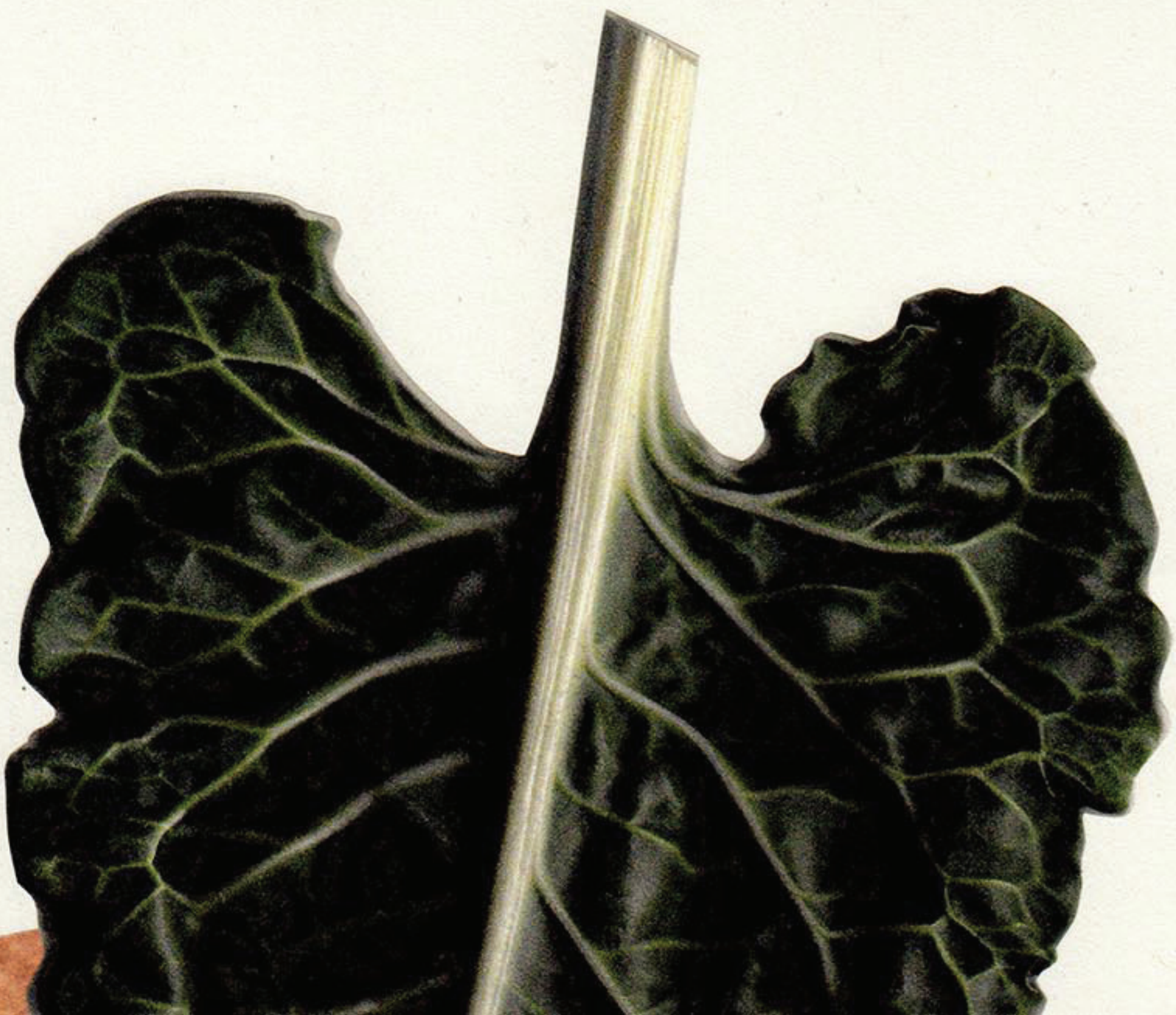

Spinach Quiche

Wesbank CAN

- 1½ cups flour
- 6 tbsp butter
- Ice water
- Chicken breast, cooked and chopped (optional)
- 1 tbsp butter
- 1 small onion, chopped
- 1 cup spinach, chopped
- ½ cup cheddar cheese, grated
- ½ cup cheese of your choice
- Salt and pepper
- 4 eggs
- 1 cup milk
- Red pepper powder

For the base

In a mixing bowl, add flour, butter and a pinch of salt. Rub between your fingers until it looks like breadcrumbs.

Add your ice water bit by bit, and mix until it forms a soft dough.

Put the dough in the fridge for 30 minutes.

Roll out the dough very thin, and cover the bottom and sides of your baking dish with the rolled out dough.

Put it in the oven and blind bake for 5-10 minutes. Then Put it aside and let it rest.

For the filling

Fry onions in butter until soft.

Add chicken(optional)and spinach and simmer for 5min.

Put the cooked chicken and spinach in the baking dish (on top of the baked base) and add the cheese, salt and pepper.

In a mixing bowl, mix eggs milk and red pepper.

Pour the egg mixture into the baking dish over the filling, and bake for 40-45 minutes in a pre-heated oven 180'C.

By Edith Naomi Van Wyk-Nelson

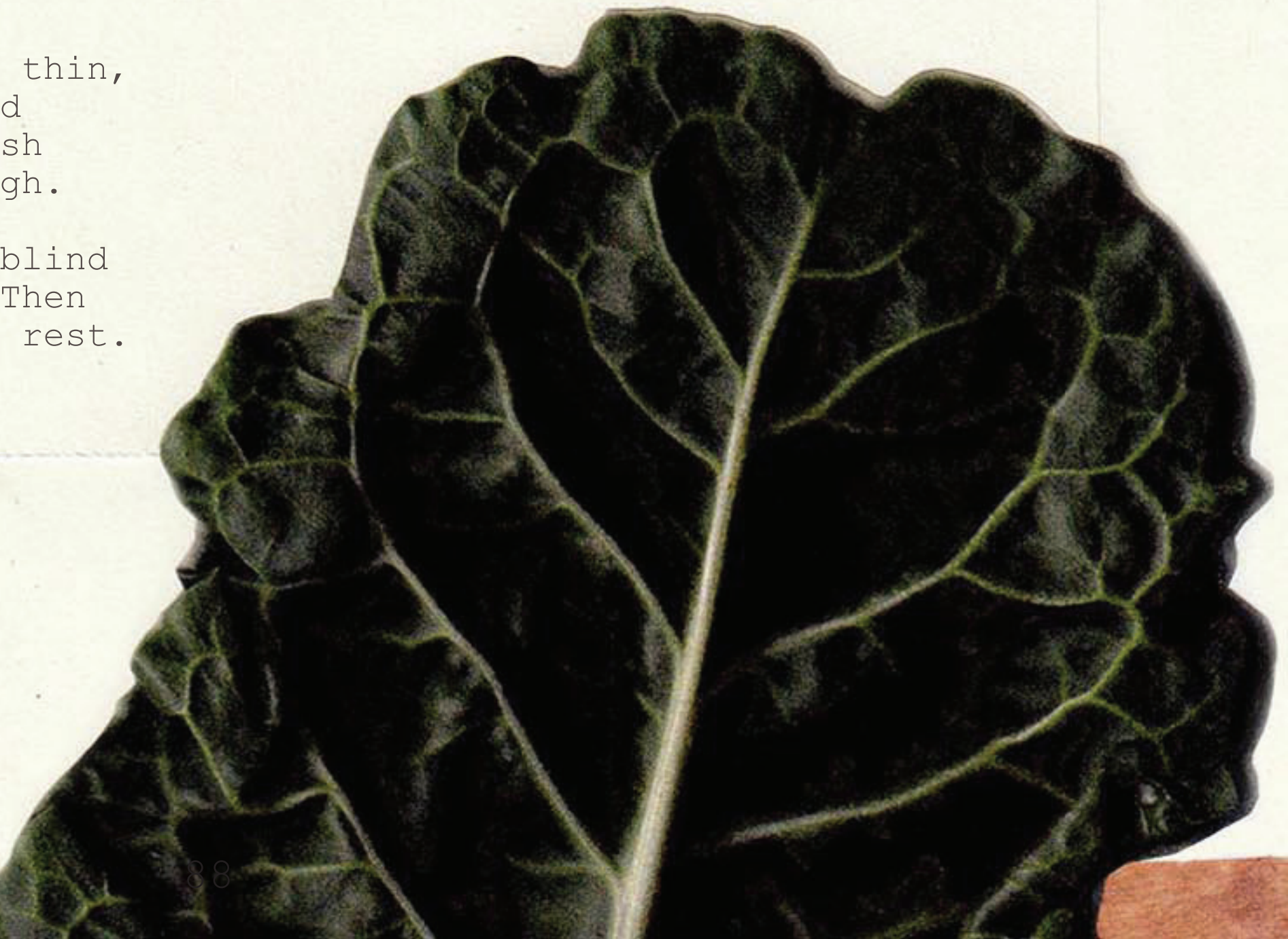

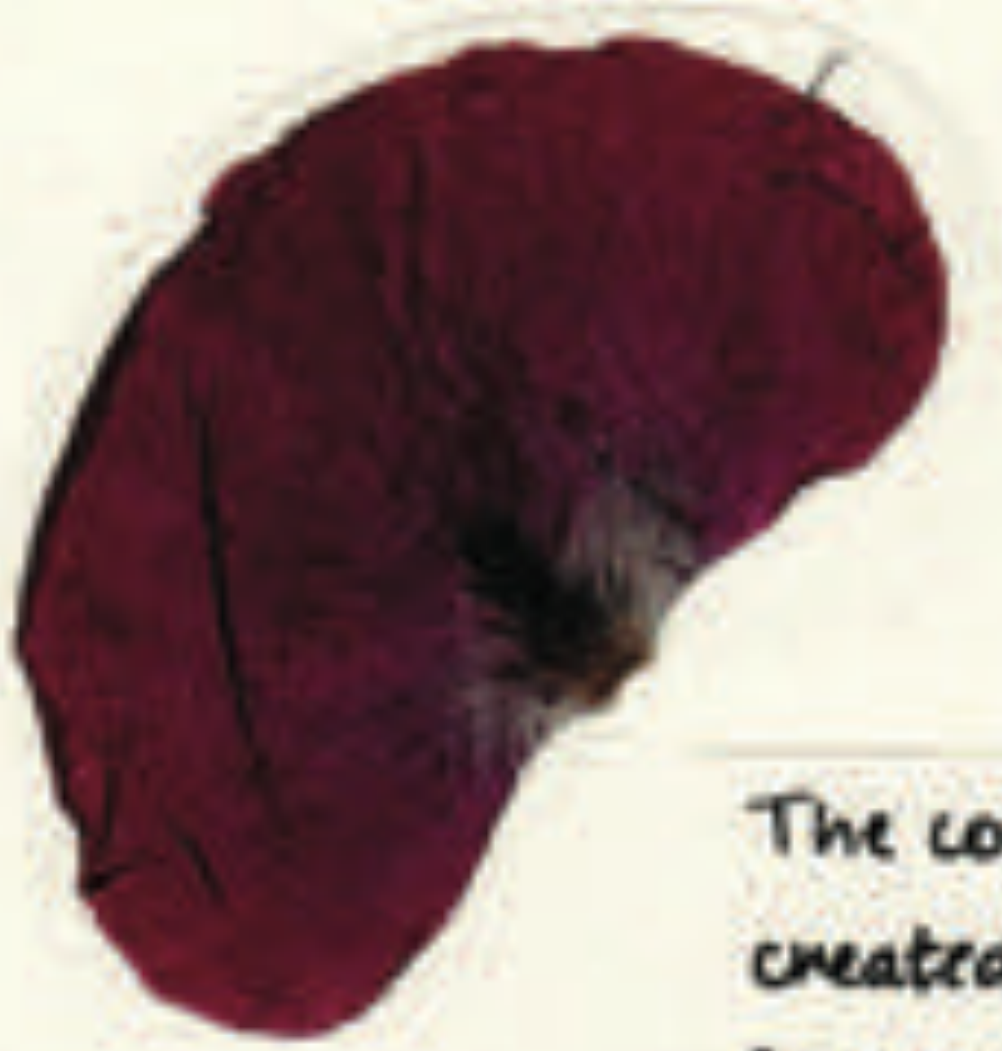

The coronavirus has really
created a large potential
for pulling apart, so you
see people divided on politics,
on what they think about
lockdown regulations,
whether they follow them...
Being part of a CAN is an
opportunity to build, to be
part of building and
creating something together
- Brendon Bosworth
Muizenberg CAN

Nadine's Smoortjie

ObsCAN Feed You

ObsCAN Feed You is an evolving community kitchen that emerged in the COVID-19 lockdown, level 5. The aim of the initiative is to move beyond the soup kitchen model and – through mobilising resources of time, money and food within the neighbourhood – to build relationships between house-based and street-based residents

- 2 kg onion, finely chopped
- 4kg tomatoes, finely chopped
- 200 g (1 tub) tomato paste
- 4 tins baked beans
- 3 tins diced tomato and onion mixture
- 3 tbsp garlic paste
- 2 tbsp vegetable spice
- Salt and cayenne pepper to taste

Gently fry the onions and garlic for a couple of minutes until soft, transparent and fragrant

Add a few ladles of chopped tomato and the tomato paste. Mix and scrape to get all the stuff off the bottom of the pot, and to make a thick paste

Season with vegetable spice and salt and pepper to taste

Add the rest of the tomatoes and canned tomato onion mix

Bring to boil and simmer 10 minutes

Just before serving add baked beans, taste for seasoning and heat through

By Nadine Oosthuizen

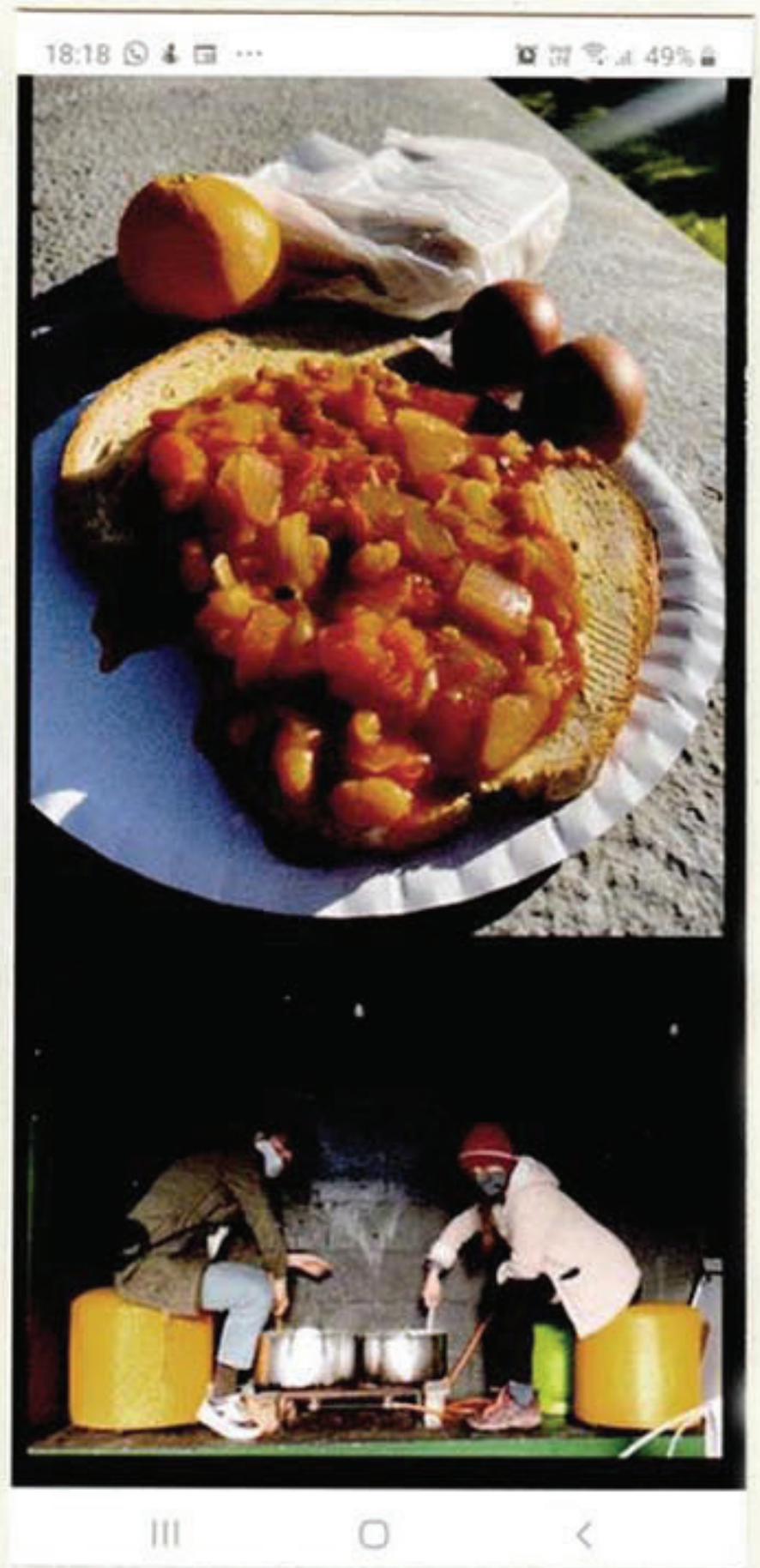

Milho

Goodwood CAN

- 15kg maize meal
- 3 handfuls salt
- 3 cups olive oil
- 1½g fresh parsley
- ½ cup sugar
- Oil for frying

Add water, salt, olive oil, sugar and parsley in a pot and bring to the boil

Slowly add the maize meal, stirring consistently until you get a thick paste

Pour the mix into baking trays and allow it to set over night

Cut the mixture into squares and deep fry

Serve with smoortjie

By Maria Helena Fernandes

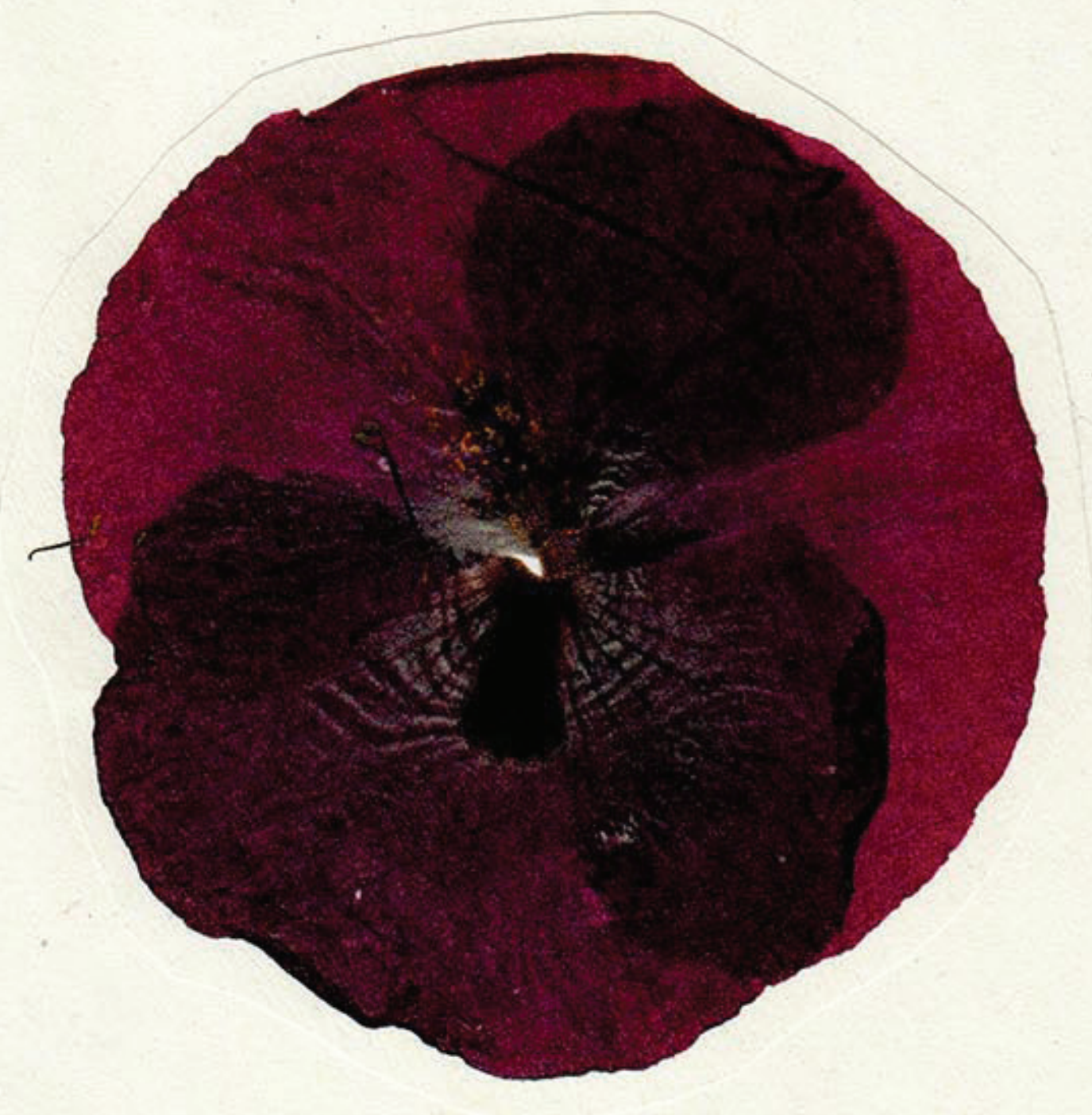

Bringing Goodwood together

Goodwood CAN

Goodwood is situated between two railway lines. The mainline is popular, consists of people from most (if not all) townships and is usually overflowing with traffic. In the mornings especially, one is unlikely to find a seat on the train. The line is close to Voortrekker Road, where there is public transport-ation. This part of Goodwood is congested and can be classified as the less affluent part of Goodwood.

The Monte Vista line is not too popular, is generally used by professionals, is never full and one can even use their laptop on the train. This line passes through the affluent part of Goodwood which is along the N1 highway where there is no public transport.

The idea behind the Goodwood CAN logo is to acknowledge these disparities and startling differences. Our mission is to unite as diverse people from both sides of the railway line, serving everyone equally since Covid-19 knows no class, race or social standing. The CAN tries to be the link that brings Goodwood together, to overcome the "us and them" mindset.

Funke got things rolling when she joined the bigger Cape Town Together group…

"I asked, 'is there a Goodwood CAN?'. The answer I got was – 'no, but why don't you start one!' I'm not used to being in such a position but, anyway, I put my hand up, along with Adelaide – another CAN-member. Lo and behold, we started getting requests for food. I remember it was a Saturday morning and we received requests from four people. Adelaide and I managed to source some funds and help the families. Within twenty-four hours we had more than twenty requests. We knew we would need to come up with a bigger plan. Verona joined, Selaelo joined, and we became a squad of four. Suddenly, there were lots of donations and food parcels to distribute."

"The beauty was that none of us knew each other (we didn't meet in person for months) but it was just flowing, that was the beauty of it. We collected food parcels, we distributed, we kept track of things on excel, everybody stepped up to get things done – and they got done! There were so many connections, and it all just evolved fluidly over time. We started with the packed parcels and then somehow, somehow – I don't know how - we started serving meals. And it just took off like that!"

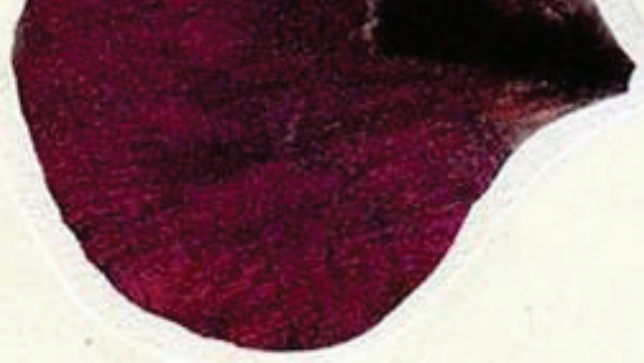

One of the biggest things the Goodwood CAN took on were the unhoused people living in the area. Homelessness has become very dehumanised by the neighbourhood watches in our area. The neighbourhood watch members abuse homeless people, they do not treat them with respect or dignity, they just don't see them — or maybe they see them but don't recognise them — and that has gone on for years. For us it was important to be a part of a network or a movement or even a group of people that humanised humans, regardless of their class or their race.

Selaelo spends sleepless nights because of the dehumanisation of humans who find themselves without a house. "I don't think of them just as homeless people, they are people who happen to find themselves homeless. Or, as Adelaide says, who find themselves unhoused. When someone is thirstier than they are hungry - that, for me, is dehumanising to the core. Water is a natural resource, no one should own water privately, but we have made it to be a resource that somebody owns, that the government owns, that local people own."

The CAN helps a lot of different people, and not all of them are homeless. The food we make is for everyone, we'll feed whoever is in need of food – which could be any one of us in these times. And we've learned important lessons from our unhoused neighbours in Goodwood. Their way of protecting one another, looking out for one another, when you give food to one person they will ask also for their partner, for their neighbour. And the fact that, despite the difficulties they face, when they see us there is warmth in their welcome to us, over time we have built trust, we've become like family.

By Funke Alaba, Selaelo Arendse, Verona Mathews and Irene Olwoch

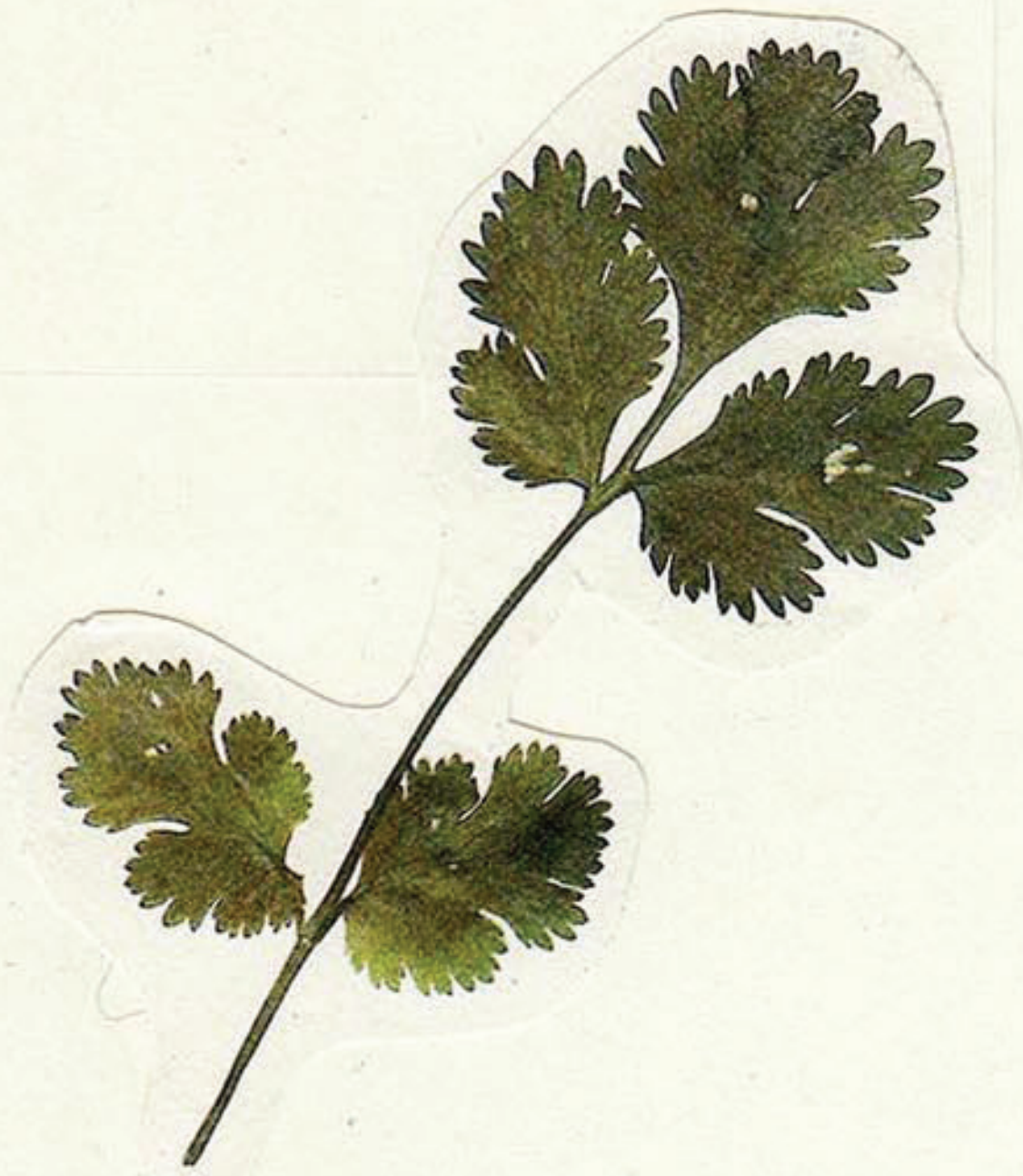

Recipe for a WhatsApp Group

Connecting CANs

The CAN Admins WhatsApp group was a central organising node of the network, including at least one member from all the CANs across the city. At the height of the pandemic there were over 200 people in the group, communicating about a vast range of issues, responding to calls for help, sharing accurate information about Covid-19, fact-checking viral messages and generally helping to create a sense of togetherness within the network.

Given the circumstances of lockdown and social distancing, this digital space was one of the only shared spaces where all the CANs could be together. It was also one of the most generative and safe nodes of the network, where an intentional culture of respectful communication, engagement and calling-in was cultivated - not something to be taken for granted in the digital space!

This recipe is a guide to the key ingredients for digital organising in the time of Covid-19.

What you'll need

- 1 WhatsApp group
- 6 group admins
- Over 200 group members who probably haven't met in person
- 1 daily summary
- Collective care, calling in and communication skills
- A fresh bunch of emojis 👌 😋

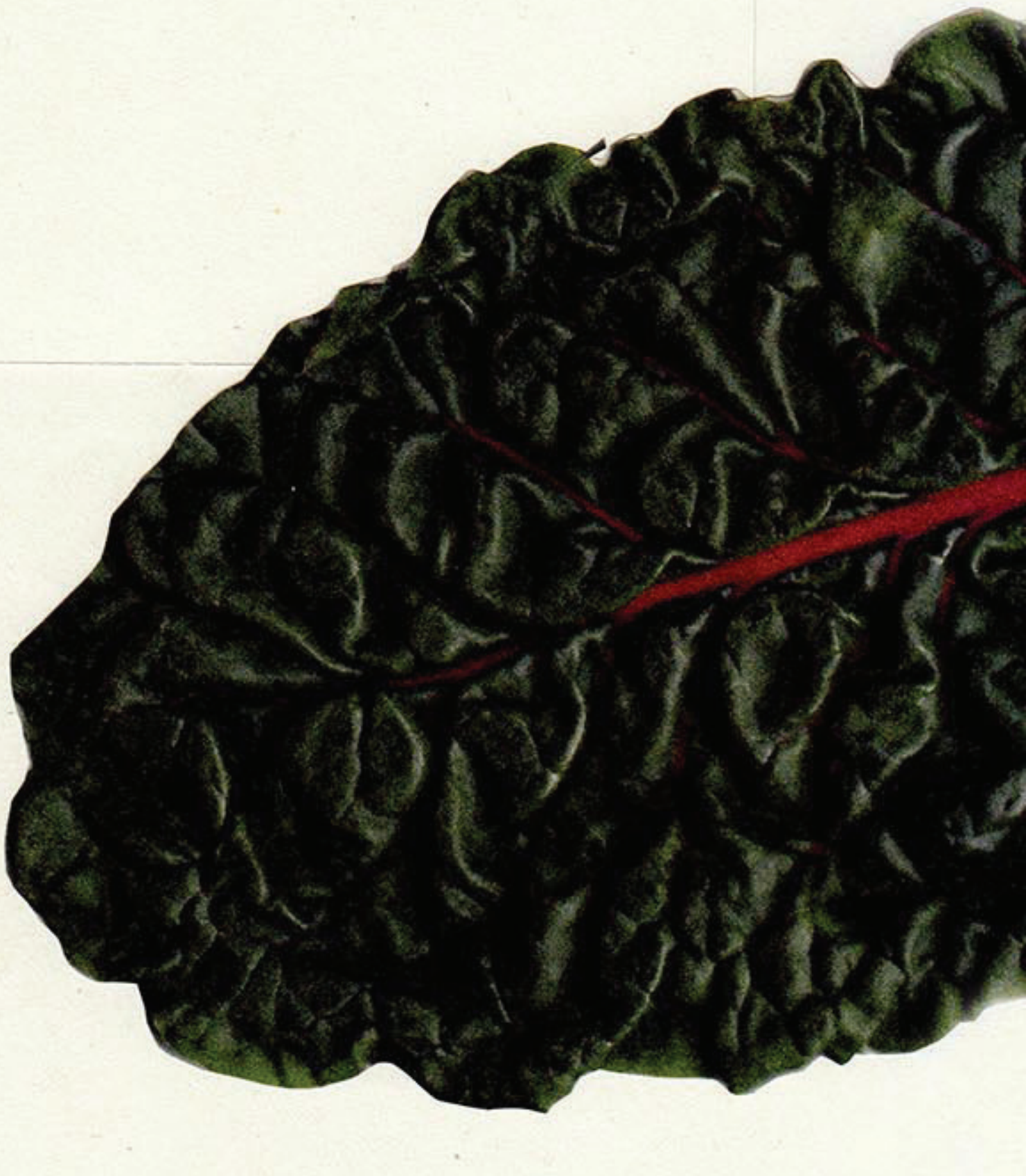

What to do

1. Set up a WhatsApp group, giving it a simple name – nothing glamorous. Remember, there are no hierarchies, this space is just another organising node.

2. Watch as the group grows, mirroring the growth of the network and the spread of the virus. Realise that the group might need a bit of curation in order to be a useful organising tool.

3. Gather your volunteer group admins and set some concrete boundaries – close the group between 8pm and 8am so that CAN members can get some sleep.

4. Establish simple practices that help people to connect with each other on the group – such as signing off every message with one's name and CAN, and using reply functions and message titles so that people can follow the conversation even if they joined in late.

5. Have the group admins post a daily summary of all that's been said just before closing the group – so that people who have tapped out that day can catch up without reading the whole thread.

6. Generate and sustain a culture of calling in, rather than calling out. Engage the contentious or offensive things that will inevitably come up in a respectful, transparent and caring way, without shaming or excluding people off the bat.

7. Remember that the network is a guilt-free zone, that fatigue is real and that central nodes like this work best because they are collectively cared for and engaged with.

8. The group might ebb and flow, people will leave and more will join. But once a respectful culture of engagement has been established it will stick around without much work or prodding.

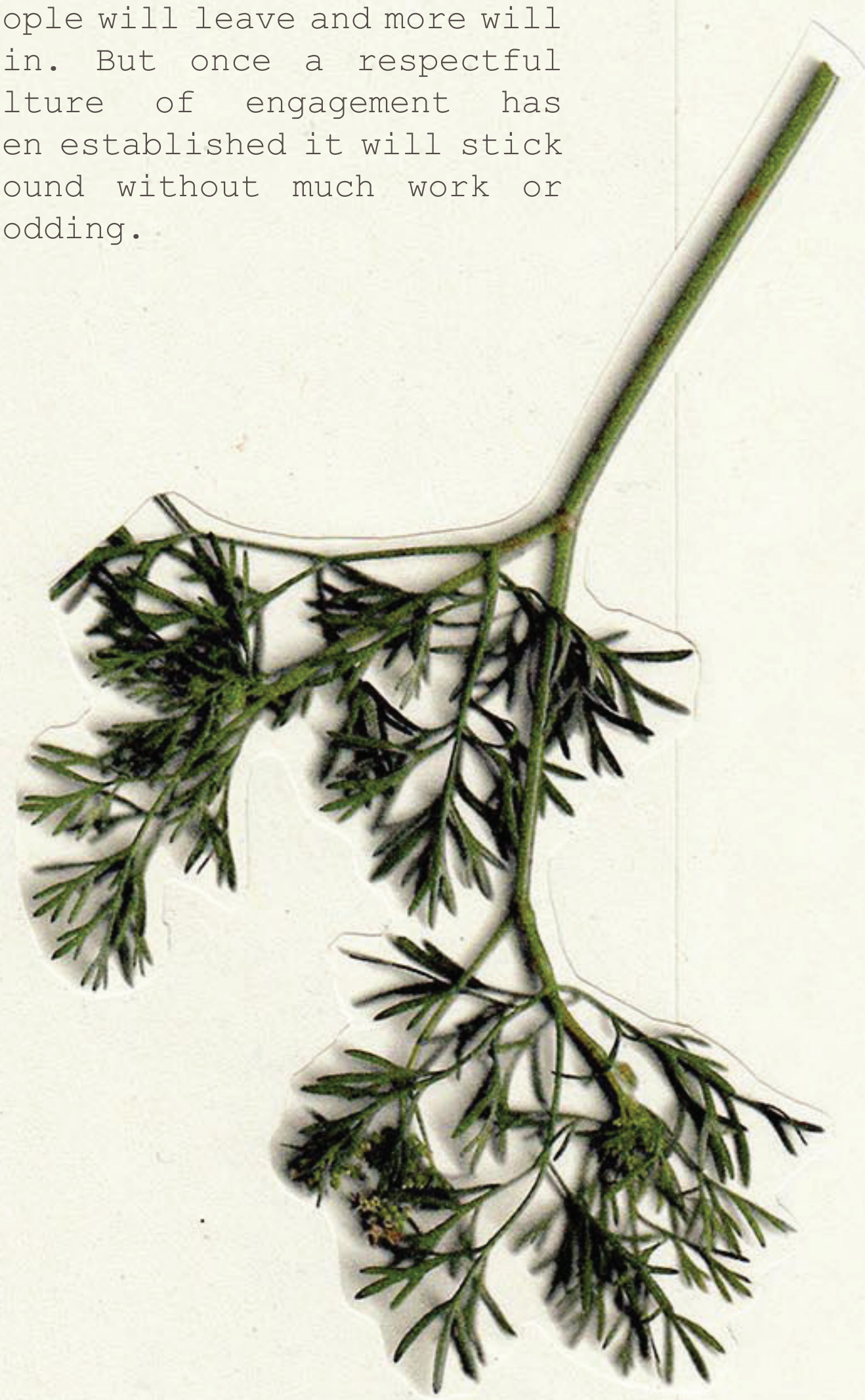

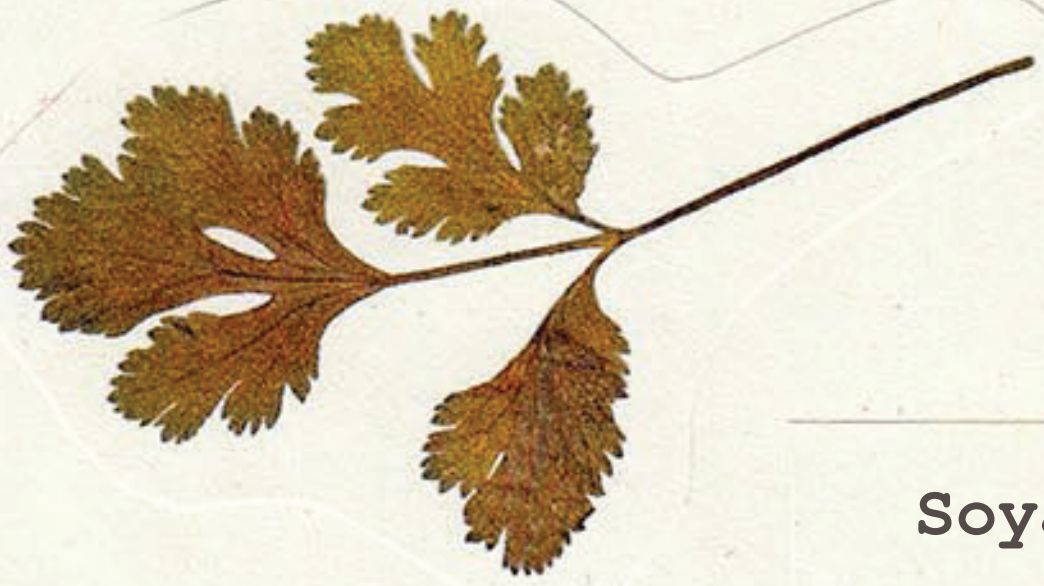

Soya Mince Frikkadels

Ocean View CAN

- 5 cups dry soya mince
- 3 medium onions, chopped
- 2tbsp crushed garlic
- 1 green pepper
- 3tbsp leaf masala
- 3tbsp BBQ Spice
- 3tbsp chutney or sweet chilli
- 10 slices of bread, soaked in water
- 3tbsp sugar
- 6 large eggs
- Salt and pepper
- Oil for frying

Rinse and strain the soya mince (to remove the pre-packaged spices)

Put the soya mince in a bowl and cover with boiling water. Allow it to sit for at least 5 minutes while the soya mince expands

Strain it again and make sure it's dry and soft

Add the chutney or sweet chili sauce, onions, green pepper, salt, pepper and spices and mix well.

And the bread, eggs and sugar and mix well

Roll the mixture into small balls

Deep fry the balls in oil until firm and golden brown

By Teagon Klein

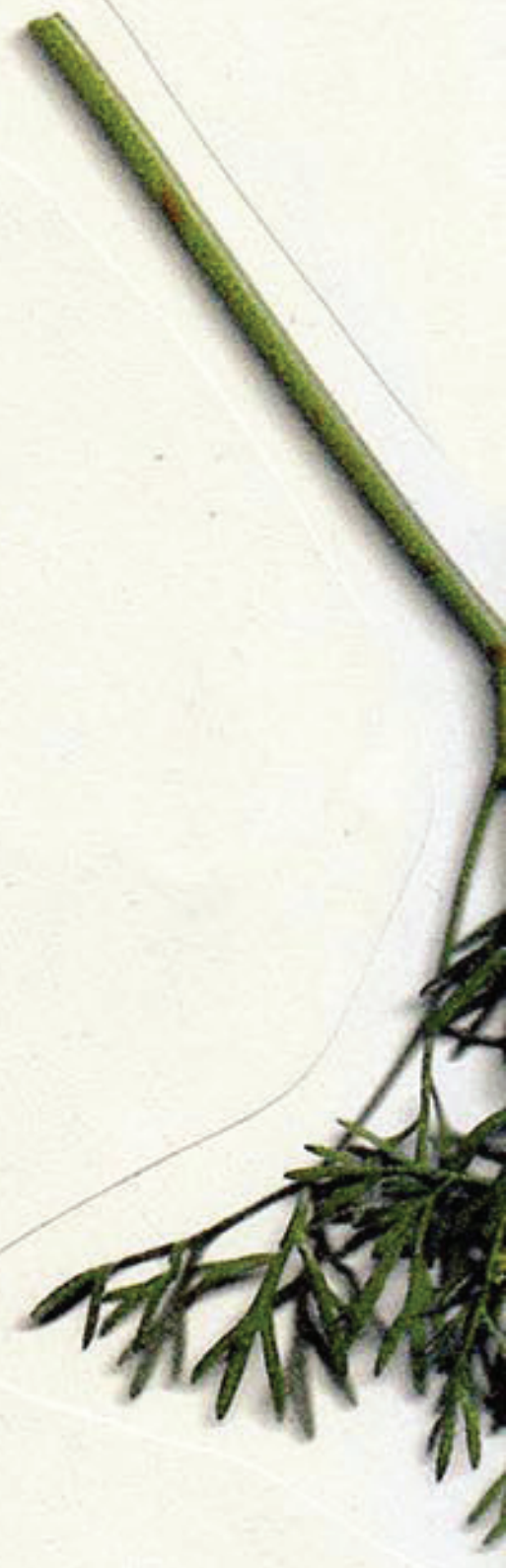

Tomato and Onion Salad

Salt River CAN

- 1 small onion
- 2 or 3 firm tomatoes
- Juice from 1 lemon
- 1 chilli
- Salt and pepper

Chop/dice the onion into small pieces

Pour boiling water over the onions

Chop tomato into diced pieces

Drain the onions

Add the tomato to the onions

Add the lemon juice

Sprinkle with salt and pepper

By Nadia Agherdine

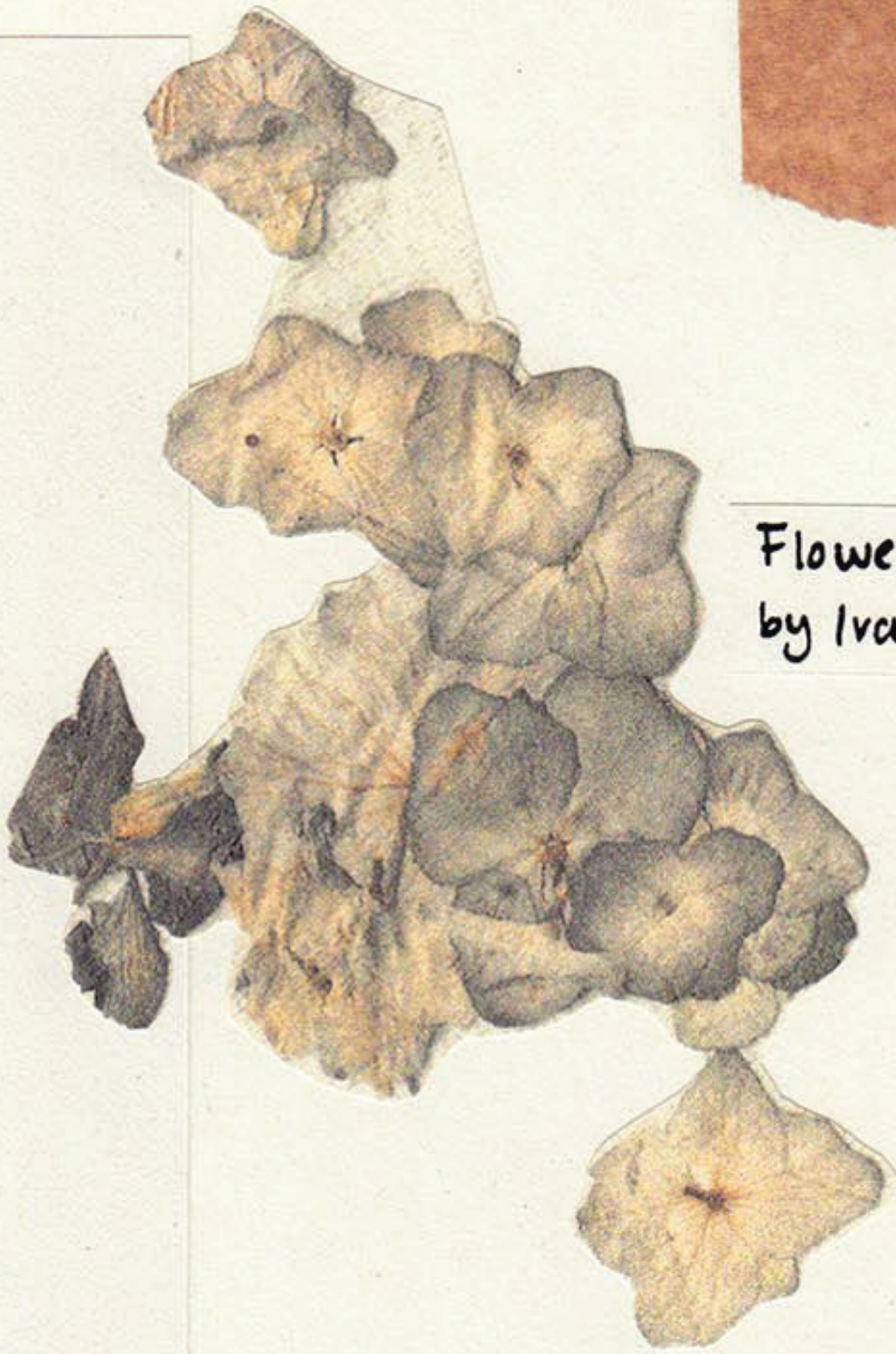

Flowers pressed by Ivan Agherdine

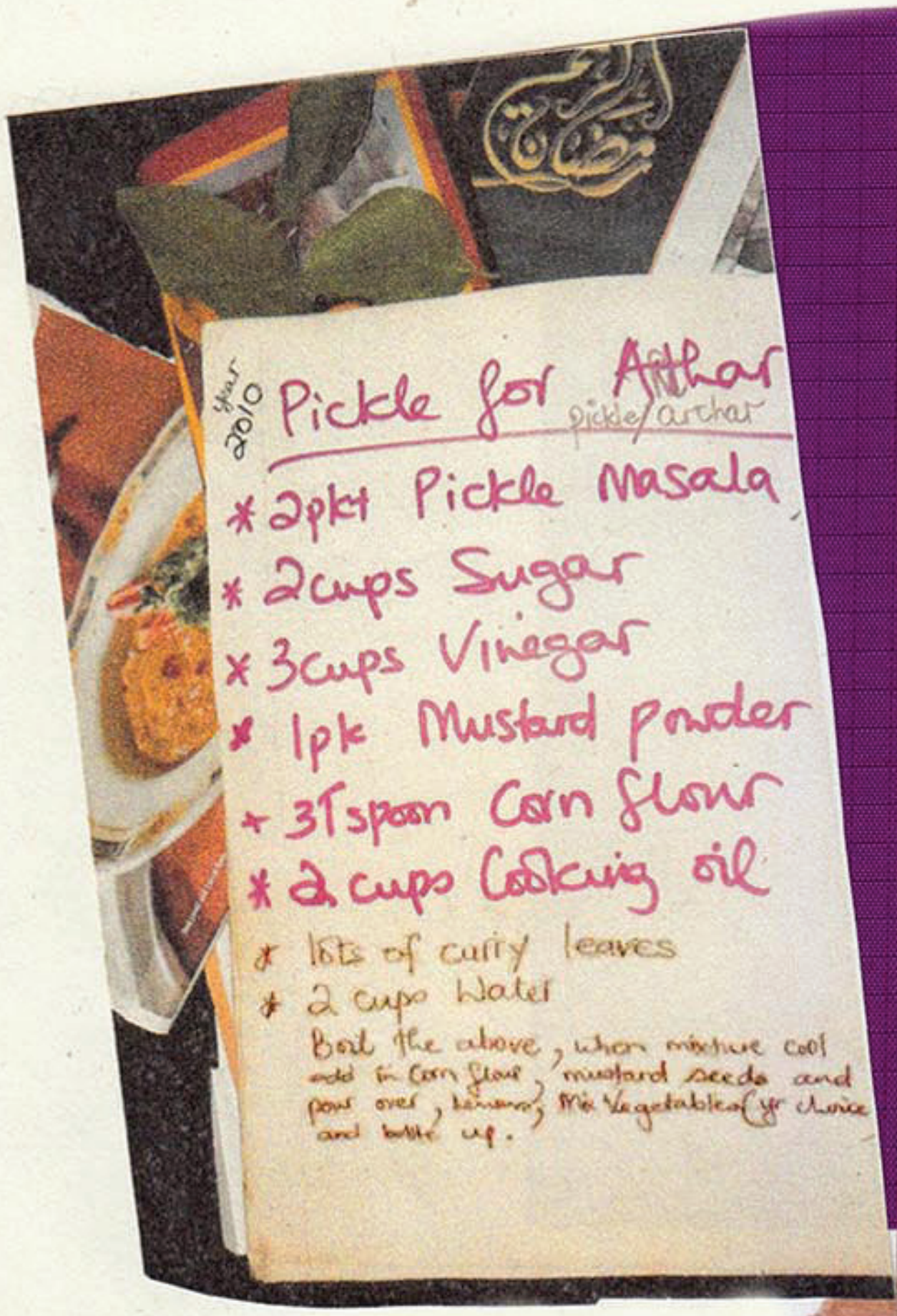

Year 2010

Pickle for Atthar
pickle/archar

* 2pkt Pickle Masala
* 2cups Sugar
* 3cups Vinegar
* 1pk Mustard powder
* 3Tspoon Corn flour
* 2 cups Cooking oil
* lots of curry leaves
* 2 cups Water

Boil the above, when mixture cool add in corn flour, mustard seeds and pour over, lemons, mix vegetables (yr choice) and bottle up.

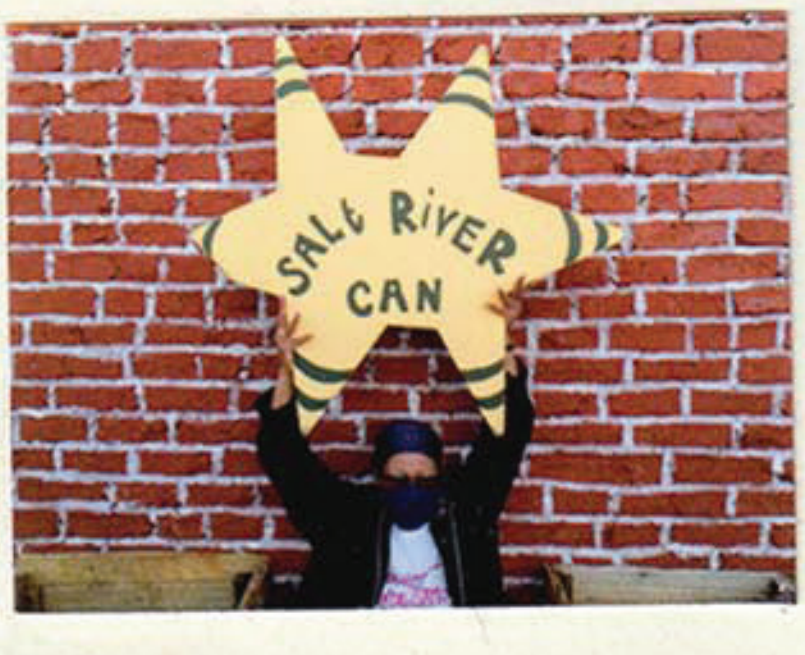

-Nadia Agherdine
Salt River CAN

A recipe for learning together

Co-learning team

The online co-learning sessions were cooked up to strengthen the community action network through deliberate, regular, learning and sharing spaces.

INGREDIENTS

- A group of CAN-do people with a desire to share experiences and stories and learn and reflect together
- A space (either online or in person)
- A commitment to inclusion, collaboration
- A desire to bridge space between practical learning and deepening our personal/political/social consciousness
- The most indispensable ingredient of all good home cooking: love for those you are cooking for — Sophia Loren

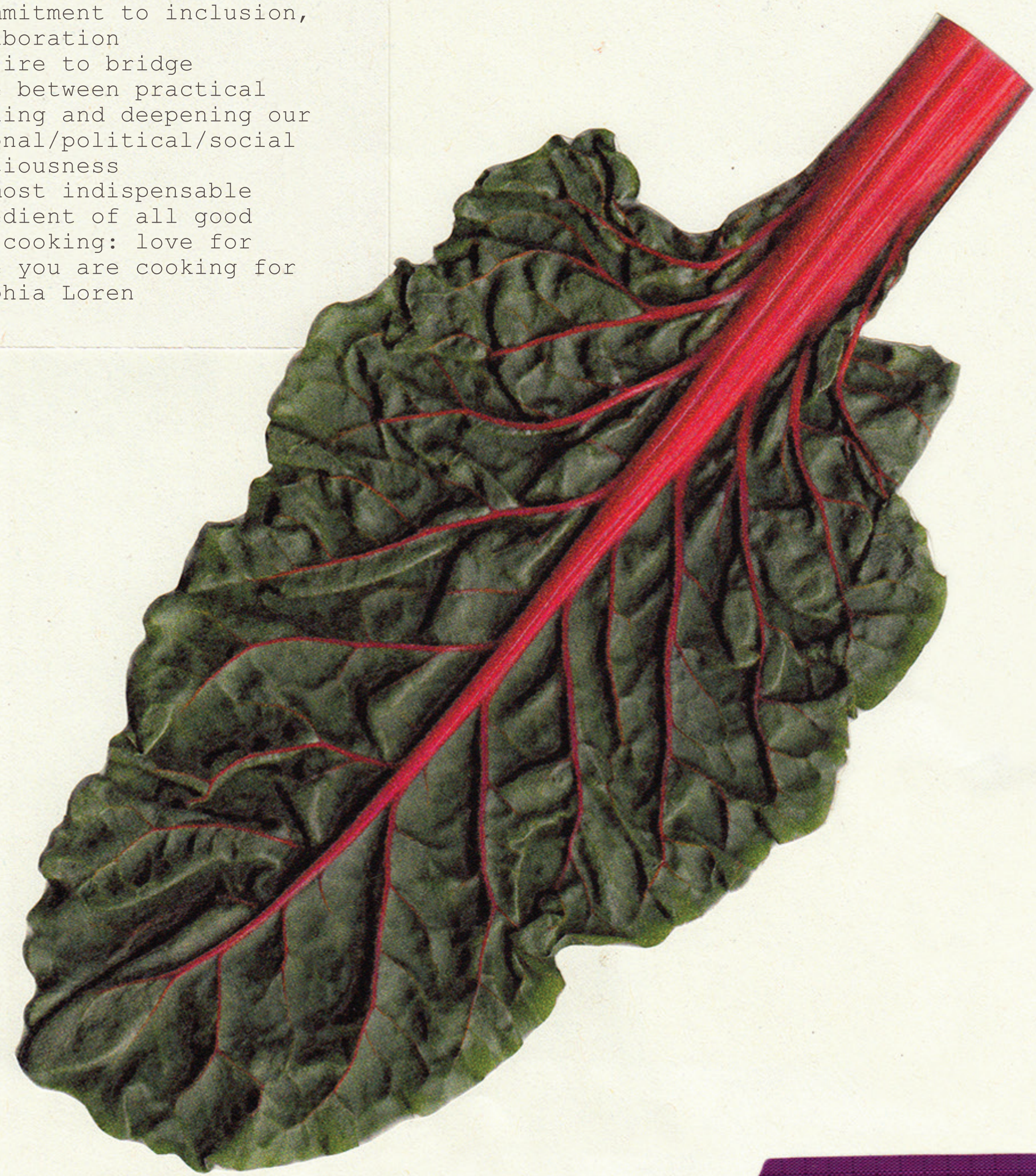

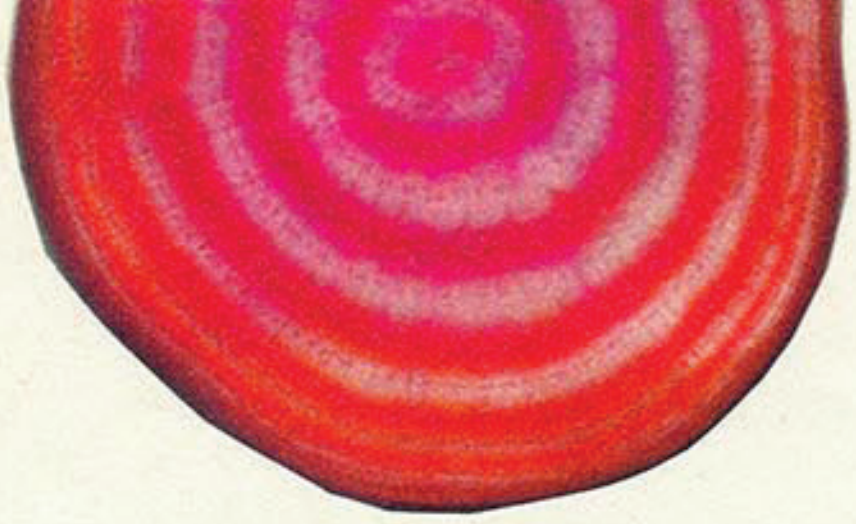

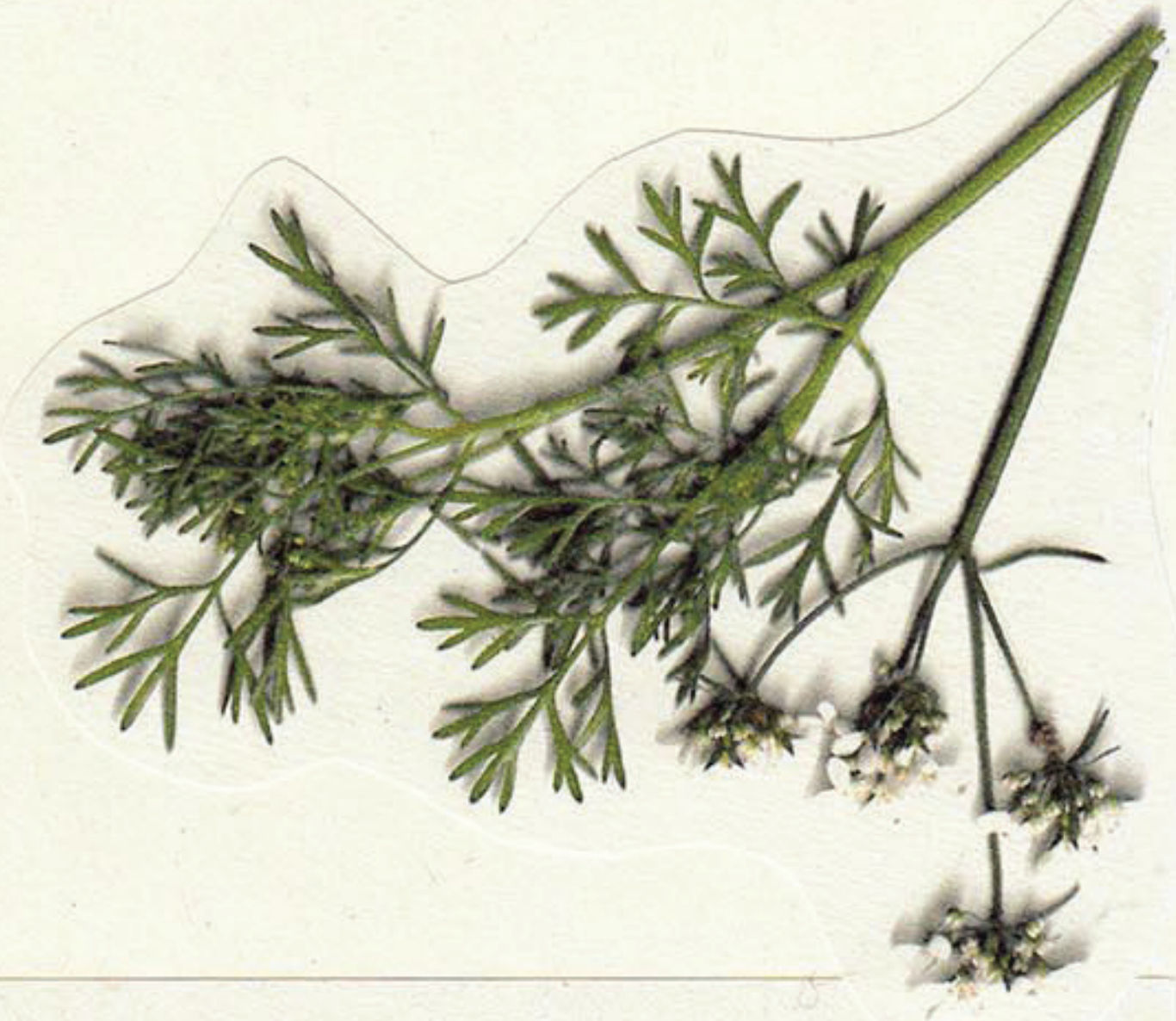

PREPARATION

A dish is only as good as its ingredients. Follow these simple steps *before a co-learning session* to ensure a delicious melange of flavours as well as a unique outcome.

Step 1: Decide on the purpose of the session

We sought input from a variety of CAN members to ensure that there was an agreed upon purpose and direction for the session, clarified expectations and ensured representation of different voices.

Who needs to be involved? Whose voices need to be heard? Who is missing?

We contacted speakers in advance to set the tone and explore what piece of the story they were telling. People were excited by the co-learning nature of the sessions, emphasising that it was less a lecture space, and more about learning together and sharing experiences.

How do we make the session as accessible as possible? E.g. using a combination of Zoom, written contributions, audio recordings, answering key questions etc.

We also offered data and offered to meet earlier to check that all the tech works. This helped reassure people and tried to make people feel more included in the process.

Step 2: Pick a chef

We all know that too many cooks spoil the broth, so ask someone to facilitate the discussion. We had a group of facilitators that we could draw on, one to lead the co-creation and facilitation of each session. They created the virtual holding space, introduced the speakers, gave Zoom directions, and invited different voices into the conversation. We also had a sous chef (process facilitator) who helped with the technical aspects like the creation of break-out rooms etc.

Step 3: Setting ground rules or social contract for meetings

Sustain energy by being punctual for sessions and keeping them to time (most sessions were one hour).

Be inclusive by keeping contributions short (this gives space for others to contribute).

Build solidarity and respect differences. Recognise that there are people from different backgrounds and cultures and politics. The co-learning facilitators tried to find ways to acknowledge and address conflict and differences that arose.

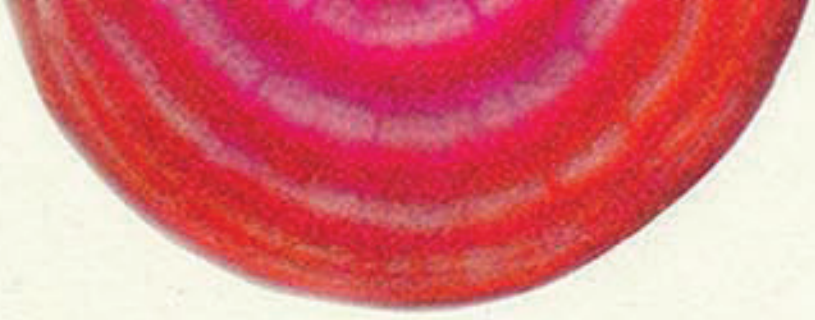

INSTRUCTIONS

Although every session is unique, here are a few things you can do during the session to ensure your session is satisfying and keep participants coming back for more.

Check in

This gives everyone an idea of where the energy of the "room" is. In real life you would get a sense if people's energies are low but with online groups you have to be more intentional. Using prompts is an easy way to do this. For example: one word of how you are feeling; what animal are you feeling like today; how are people feeling 1-10; what woke you up this morning; what song/movie do you feel like today?

Review the session's purpose

This allows you to gently bring people back to the task at hand as one session is never long enough to cover all issues. If something pressing arises during the discussion, ask the group if this is something that needs to be covered today (in which case you shift the agenda) or it is a subject for the next discussion.

Open and hold space gently

We encourage facilitators not to be afraid to name issues that arise and flag that this is exactly what we are trying to learn about. For example, there have been tensions around the difficulty in trying to create community with people from very different class positions. We asked ourselves - How is this tension part of the process? What might we learn from it? Doing this helped de-centre the potential destructive discomfort we feel when confronting tensions, and re-centre that we are people trying to work together in difficult circumstances with a lot of necessary learning as we go.

Close the meeting

Once the meeting is over, it is useful for the facilitator or notetaker to review key points and then ask everyone to do a short check-out. Similar to the check-in, use prompts, for example, what is one thing you have learned? Or a rating system after each session where we rate ourselves (what we brought) rate the session (not if we enjoyed it but what we got out of it) and lastly, we all state what we'd like to be acknowledged for and by.

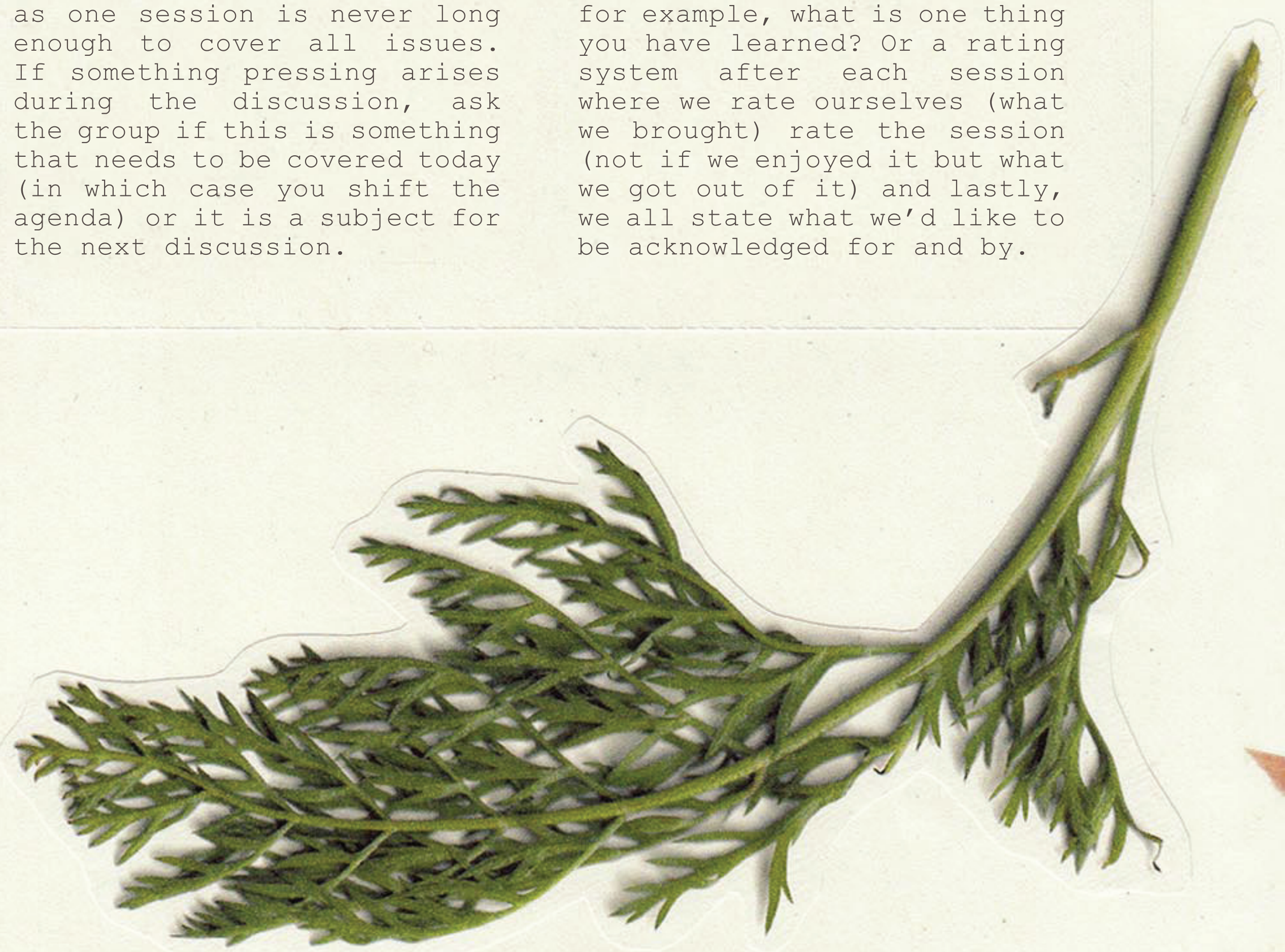

GARNISH

Attention to detail is key to an appetizing dish. After the session, share key notes and follow up actions, share the date for next meeting and the Zoom link, and share any other resources.

NUTRITION FACTS

Community solidarity	99%
Inspiration	94%
Shared frustration	89%
Friendship	86%
Laughter	65%
Tension	24%
Awkward silences	20%
Difficult conversations about power and privilege	55%
2020 Burn-out	5%
Love	100%

Written by Ella Scheepers,
spiced up by Ishtar Lakhani

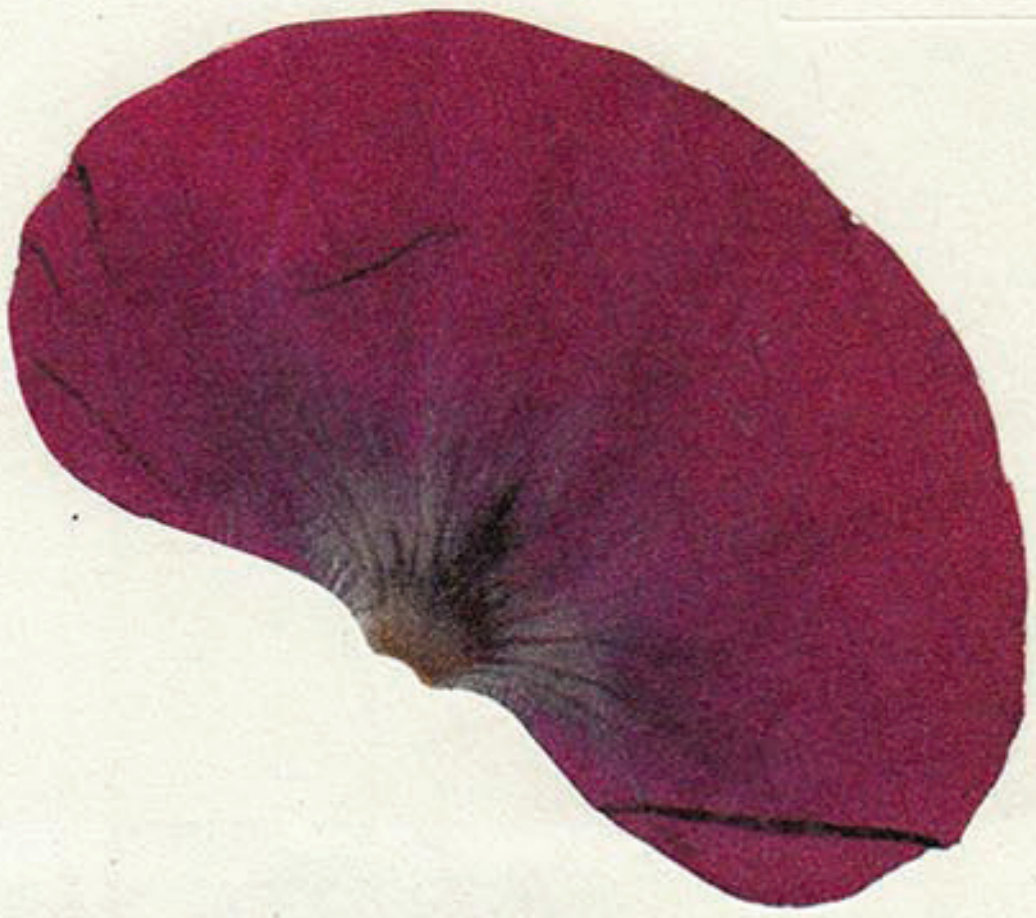

Veggie Steam Bread

Site C CAN

Site C CAN saw that Accountability Labs was looking for partners in hosting community dialogues, and we approached them as we were planning to host one already. They gave us the space to present our own context and we decided to talk about how we hold one another accountable as youth. Migcobo brought veggie steam bread in a cup shape. We had them during tea time or as a pre-lunch snack. They were such a hit that people in the dialogue asked for more. Migcobo had to fetch more from home as she under-estimated how much they would be enjoyed. We ended up having them for lunch as well!

- 1 green pepper, finely chopped
- 1 red pepper, finely chopped
- 1 yellow pepper, finely chopped
- 1 carrot, grated
- 2 cups flour
- 5 grams yeast
- Pinch of salt
- Warm water
- Oil/butter for greasing

Mix together dry ingredients

Add warm water a little bit at a time. Mix after each time with clean hands. Keep adding water until you have a soft dough

Cover with a clean dishcloth and leave it in a warm place for 1 hour to rise

Grease 6 mugs with oil or butter

Add some of the dough to each cup. The cup should be about ¾ full

In a large pot, add 2 cups of water and bring to a simmer

Place the mugs of dough in the pot and put on the lid. Steam for 1 hour

Remove the bread from the mugs and enjoy!

By Migcobo Zandile Sasa

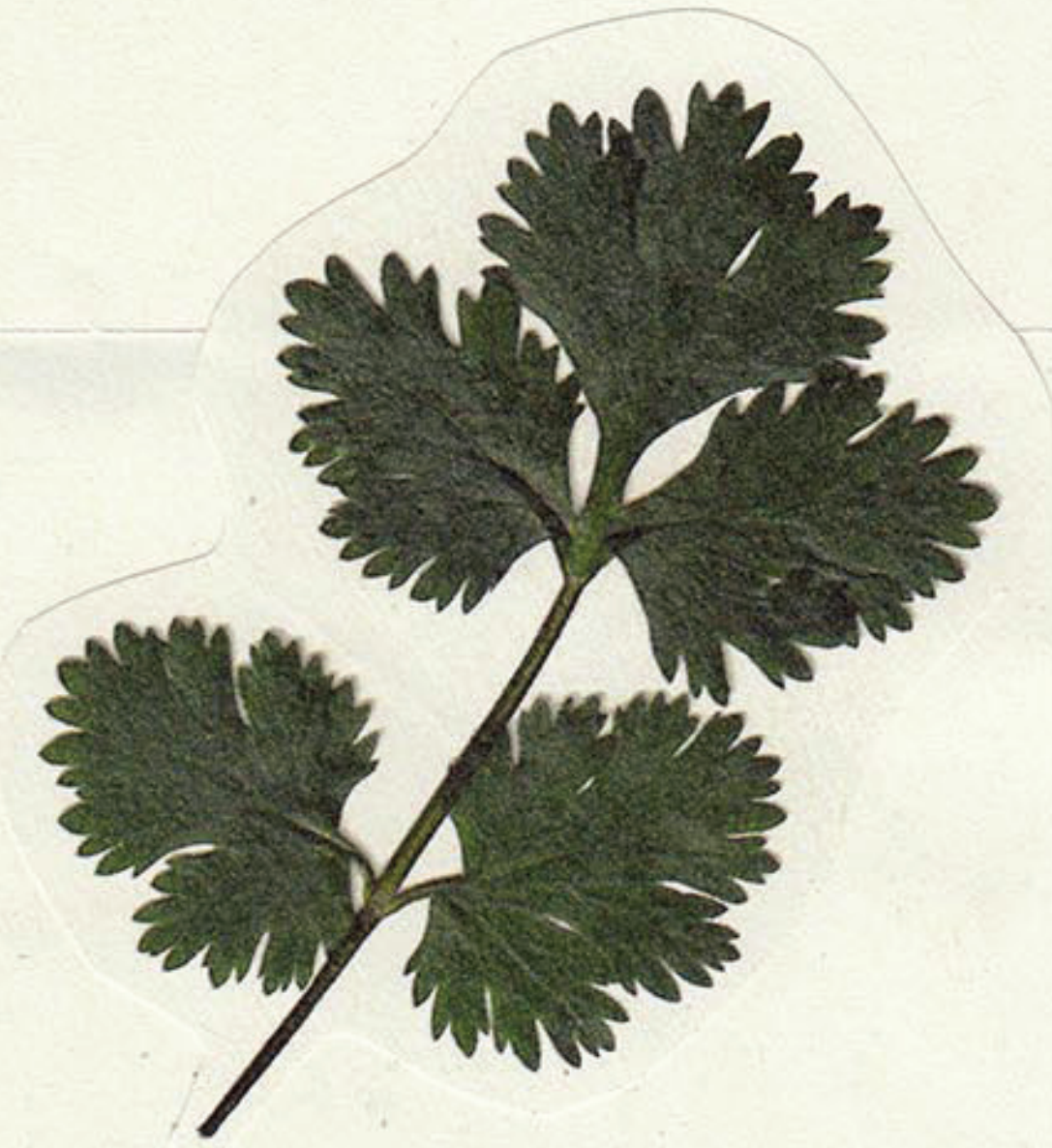

Professors of the street

Health CAN

First published by Daily Maverick as 'Sparks, flames and blazes: Epidemiological and social firefighting for Covid-19' - 24 April '20

At last week's public presentation on South Africa's strategy for tackling the spread of the Covid-19 virus, Professor Salim Abdool Karim made a persuasive argument about the need to identify and stamp out epidemic hotspots.

Demonstrating an admirable ability to communicate epidemiological and clinical information to a lay audience, Professor Karim used the compelling metaphor of small flames to describe in his public presentation on 13 April how one or two cases in a particular community can quickly grow into large fires of uncontrolled community spread. Our testing and tracing strategy is aimed at putting out these small flames before they engulf our health system and communities.

However, as has become heartbreakingly clear in the past three weeks, the consequences of this epidemic are not simply epidemiological. The people of South Africa are suffering not only symptoms of Covid-19, but also, and arguably to an even greater extent, from the symptoms of hunger, joblessness, stigma, and xenophobia. And just like the coronavirus, these pathologies are at risk of spreading through our communities like wildfire.

At the moment, one of our most valuable sources of epidemiological information is the contact tracing pods that have been set up across the country. These are dedicated teams tasked with following each and every positive case reported to the Department of Health.

Information is collected about the person who has tested positive, who they live with and who they have had contact with since becoming infectious. Importantly, this process is also used to inform the department of the challenges that people might face in self-isolating or preventing the spread of the virus while they are sick. In some cases, it also alerts us to the dangers and threats that Covid-19-positive people might face — such as stigma or xenophobia. Through the efforts of the contact-tracing teams, a number of Covid-19-positive people facing stigma and discrimination at home have been moved, along with their families, to safe isolation facilities.

However, even these teams of highly trained, well-equipped professionals can only respond to the needs of confirmed cases and their known contacts. Community-based screening and testing might help us find the epidemiological flames before they spread, but it cannot identify the sparks of unrest and injustice, of fear and anxiety, and poverty and despair, that turn neighbours into strangers and communities into bubbling cauldrons waiting to boil over.

Finding and extinguishing the sparks of stigma, xenophobia, hunger, and poverty cannot be achieved through the collection of data at all. Rather this requires social data, collected and reported by professors of the street, based on real-world experience.

Community Action Networks (CANs) like Cape Town Together, Eastern Cape Together, and more recently Gauteng Together, are some of the community-based groups organising at the local neighbourhood level, and are generating this much-needed social data and community-level intelligence.

Pamela Lindelwa Silwana, a member of the Gugulethu CAN, started cooking for her community two weeks into lockdown because people in Gugulethu were hungry. Soon she and her mother were providing food to an orphanage and an old-age home. The Gugulethu CAN was established to respond to Covid-19, but Pamela says they knew right from the beginning that food was going to be a major problem.

"People would be going home to empty cupboards, and would literally die of hunger, before they got sick from Covid-19," she says. The need for food far exceeded their capacity to produce it, so Pamela and her mom supported a neighbour to reopen a restaurant that had closed due to lockdown as a community kitchen.

"Nobody can stay home to #stopthespread, if they have no food." If government officials were listening to voices like Pamela's, they would know that behaviours will not change until bellies are full – "if people know they are getting help from the government, they will stay home and not panic."

In other contexts, at different times, it is fear and stigma that are the most urgent issues. Late on Saturday night, a frantic phone call from a member of the Philippi East CAN, Siyasanga Vilakati, raised the alarm about a quickly unfolding crisis in her neighbourhood. Siyasanga relayed the story of a woman suspected of having Covid-19, who had been heard shouting, "I don't want to die alone" and reportedly running away from an ambulance. The woman ran to her sister's house, "but her sister's neighbours threatened to burn down the house if she stayed," says Siyasanga, "because they believe she was going to spread the virus."

The woman ran nearly 10km over two days, across three different informal settlements in Philippi, fleeing out of fear of being taken away to die. But everywhere she went, she was not welcomed, partly because she was potentially infectious and partly because she is not South African. Siyasanga says that most people in Philippi don't have a good understanding of how Covid-19 is spread, or how to prevent it, and that this fear breeds discrimination.

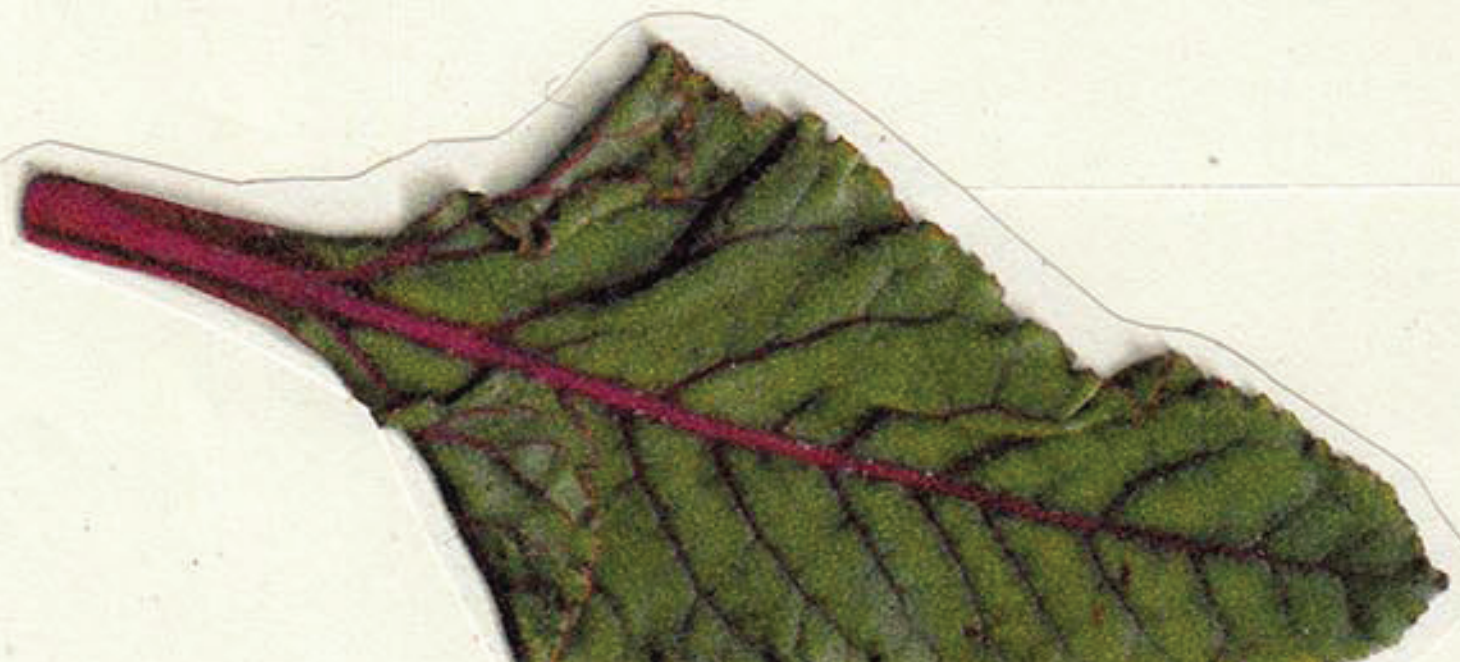

"Covid-19 could easily lead to xenophobic attacks if we don't make sure people are well informed," she adds. The woman turned out not to have Covid-19, but the sobering events of that night demonstrate that fear and stigma are as big a threat to people's lives as the virus itself. "Fear is dangerous," says Siyasanga.

The 13 April press conference focused predominantly on the epidemic trajectory – plotting curves on graphs and talking about how to squash those curves into the ground (or the x-axis). Professor Karim explained that the curve represents cases, or the rise in the number of infections. The truth is though, that those curves represent people – human beings whose lives are closely intertwined with the lives of others, and will invariably be affected by this virus and the country's response to it.

Right now, it is the job of every single government official, politician and bureaucrat to be responsive to the unintended or unexpected consequences of the lockdown, and to minimise its impacts as far as possible. This can only be achieved if we listen to, and take seriously, stories like Pamela's and Siyasanga's.

We can learn so much just by listening to people's experiences. For example, that right now hunger is a much bigger problem than Covid-19 for most people. Or that stigma is bubbling beneath the surface and is about to boil over. The government could address these issues if it just paid a bit more attention to the community-level intelligence being generated by those organising at the neighbourhood level.

While Professor Karim's presentation should be commended for its transparency and lucidity, there was an important voice missing from the panel of clinicians and academics. Where were the professors and epidemiologists of the street, who can speak to the crucial truths of hunger, homelessness and precarity that this pandemic has laid bare? It is imperative that we make space for bottom-up intelligence to inform our response to this pandemic.

By Eleanor Whyle, Manya van Ryneveld, Leanne Brady and Kentse Radebe

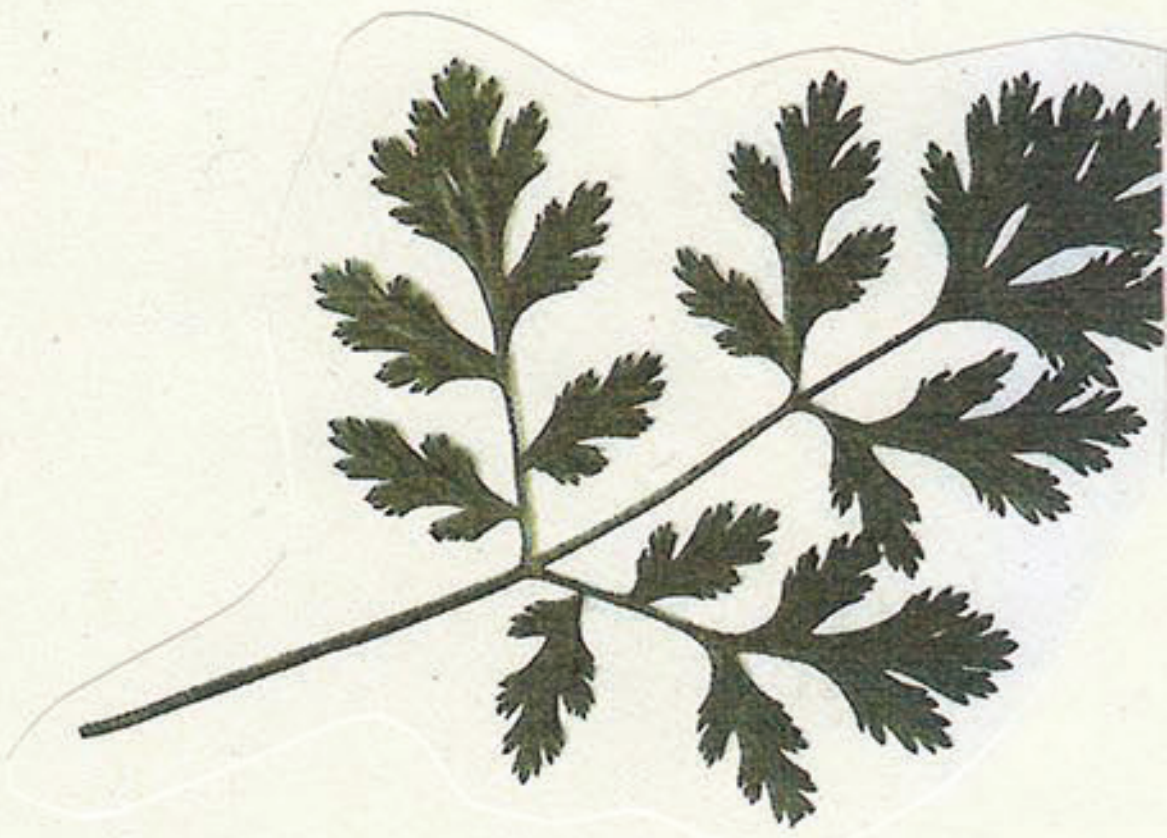

Homemade Bak Brood

Hanover Park CAN

This recipe makes 4 loaves.

- 1 medium potato
- 4tbsp sugar
- 2tsp fine salt
- ½ cup fish oil
- 4 packets instant yeast
- 12 cups cake flour
- lukewarm water
- Sesame seeds/poppy seeds/ any seeds of your choice (optional)

Boil potato until soft. Place in mixing bowl and mash

Add sugar, salt and fish oil. Mix thoroughly

Add 10 cups of flour and yeast. Mix yeast and flour lightly with fingertips

Make a well in the middle and add the lukewarm water. Add enough water for a soft texture

Knead thoroughly until dough becomes little firmer (binded)

Add the remaining cups of flour and mix thoroughly

Oil your bread tins

Divide dough in 4 equal parts and place in oiled tins

Cover the tins with a plastic board or tray (rub little oil on plastic to prevent the dough mixture from sticking to it). Then cover the tins with a warm but light blanket.

Leave to rise until the dough reaches the rim of the baking tin

Put oven on 180°C

When the oven is warm and the dough has risen, sprinkle with seeds of your choice. Place bread in the oven and bake for approximately 20 min or until golden brown

By Yaseen Johaar

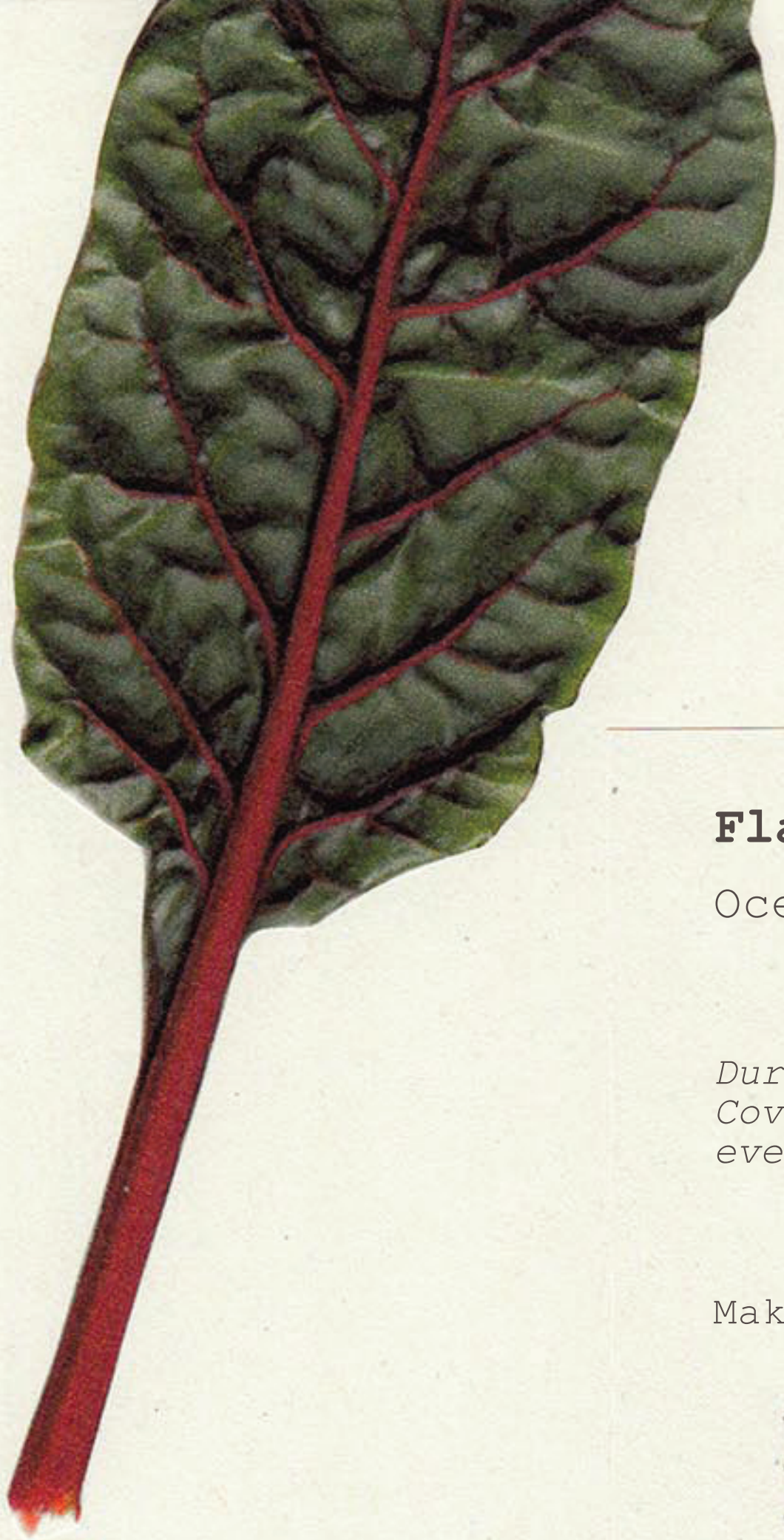

Flapjacks

Ocean View CAN

During the uncertainty of Covid-19, these flapjacks made everything just seem ok.

Makes about 20 Flapjacks

- 2 large eggs
- 500ml milk
- 2¼ cups of self-rising flour

Whisk eggs and milk together

Add flour and mix until lump-free

Heat up a non-stick pan with cooking oil or cooking spray

Pour a spoon of batter and cook for 1-2 minutes on each side

Serve warm with the topping of your choice

By Ricochet van Rensburg, Tonique Andrews and Teagon Klein

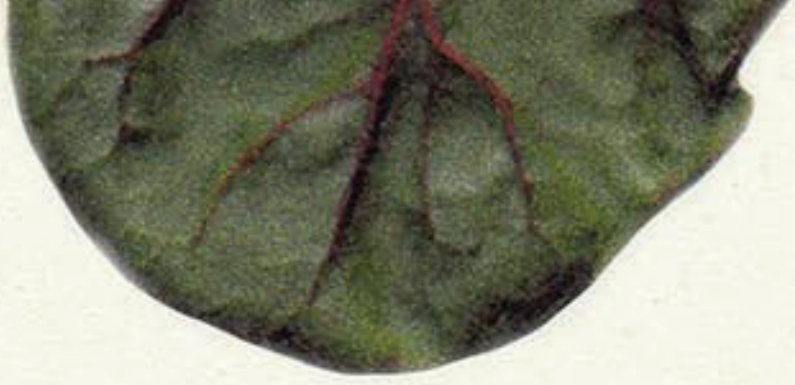

Building networks of trust

Hout Bay CAN

When we began this effort, none of us could have imagined what would be accomplished by choosing to work together! All we knew was that the pandemic was negatively affecting our community, and we chose to do whatever it took to protect people from the impact.

We found each other through individual relationships and past experiences, knowing who had responded in the past when crises hit Hout Bay. We began preparing as soon as we knew COVID-19 was on its way to South Africa, and we became more formalised when the Cape Town Together team helped us set up a WhatsApp Group for the mobilization of volunteers in our neighbour-hood.

None of us are experts in responding to a pandemic, but we each brought valuable experience to the team. We are members of NPOs and community organisations, community influencers, professionals and service providers. We specifically ensured our membership was led by organisations already active in Hout Bay, so that our effort would be sustained beyond this pandemic response. Our common ground is our willingness to volunteer time and energy to support people in and around Hout Bay.

We communicated through WhatsApp, email, and Zoom calls. We built trust by asking rather than telling, getting to understand our fellow CAN-members through the strengths they brought to our team, and choosing to agree to disagree at times, as long as the common purpose for which we had come together was accomplished.

We started out by identifying the needs we saw around us – for food, water and sanitation, and information on COVID-19. Our main aim was to ensure those who were hungry or in need in another way due to the pandemic were able to access what they needed. Working with local community leaders and influencers, through our Facebook page and through word of mouth, we identified the people and families who we could help. We vetted reported needs through trusted community leaders. We developed an online database and tracked who was provided what, when to reduce duplication and ensure equity in distribution.

Communication and organis-ation were vital. By coordinating our efforts, we leveraged our collective resources and maximised our impact. Communication with the local community was essential to understand the real impact, keep up with the latest challenges faced, and address issues quickly. While member organisations functioned cohesively to accomplish the purpose of the CAN, each organisation had autonomy to work as they knew best, which helped to reduce complications and encourage efficiency.

We made sure CAN-members and recipients understood the basics of COVID-19 transmission. Simple, easy to understand guidance was key. Collectively, we developed COVID-safety processes, and adhered to Department of Health mandated protocols to make sure that in all our actions we did not contribute to spreading the virus. We recognized making mistakes is normal, and we adjusted continually, as we learned through doing.

Being part of the broader Cape Town Together (CTT) network was also helpful. Our donors were more willing to give to our CAN because they saw we were well-organised, had longstanding working relationships with the community, and were associated with the wider CTT network. Working through registered and established organisations, with people experienced in community work, and in association with the wider CTT network gave us legitimacy that improved donor trust and supported organised working processes.

Engagement with CTT also enabled us to draw on the experience and expertise of other CANs across the city. We shared the lessons we had learnt and heard from other CANs what was working for them. Knowing other CANs struggled with similar issues to us, that we were in this learning process together, was comforting.

Ultimately, the Hout Bay CAN was able to coordinate the provision of food, information about COVID-19, and water and sanitation support to communities in need across Hout Bay throughout the lockdown. We provided people in all parts of Hout Bay with food through food parcels delivered to individual homes. Through the generous support of individuals and donor organisations such as Courage, our major supporter, we sought to ensure that no one went hungry during lockdown.

This also means that we have established a network of support that we can mobilise in response to other crises. Recently, a fire in Hout Bay destroyed the homes of 2,000 residents. Because there was already a network of responders communicating and collaborating, we were able to respond immediately to ensure that all affected residents had food, water and a safe place to sleep the very next night. Experiences like this demonstrate that we must continue moving forward together. We have the ability, resources, and motivation. Because of our experience with COVID-19, we know how to communicate effectively to maximize these resources, and we know what is possible when we work together.

By Elizabeth Drevlow

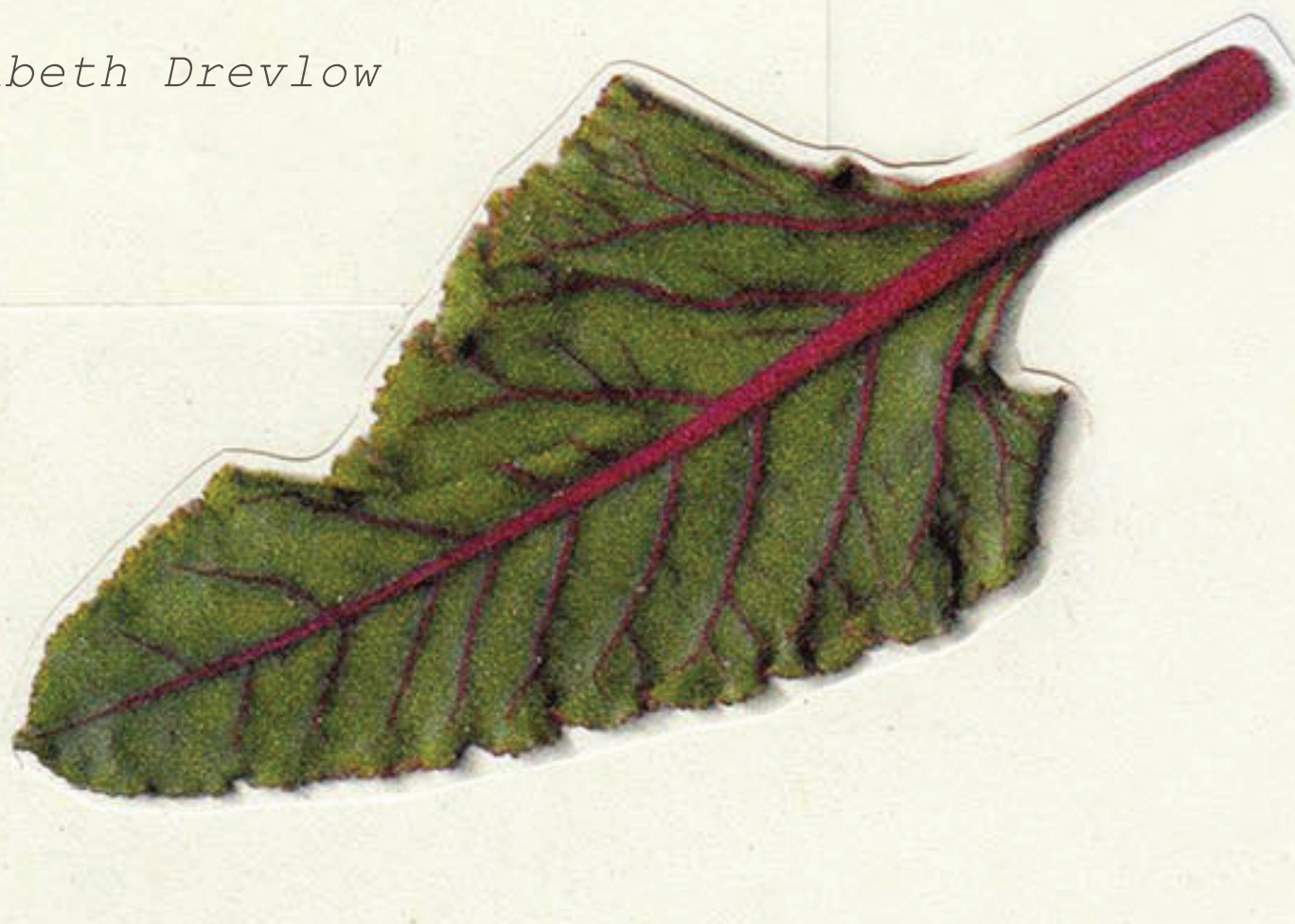

Lemon and Ginger Cordial

Newlands and Philippi
CAN pairing

The most quenching summer drink is Lemonade with Ginger! In the Newlands CAN, we put out a call in our network for anyone who had spare lemons on their trees for us to make lemon and ginger cordial. Bottles of the lemonade are gifted to informal vendors and homeless people so that they can benefit from the vitamin C and a cool drink in the heat of summer.

Before you begin, find five 750 ml used glass bottles. Sterilize them like you would a baby's bottle to ensure they are clean. To do this, boil them in a big pot of clean water on the stove.

- 1 litre freshly squeezed lemon juice (about 24 lemons)
- 1.7 litres water
- 2.5kg sugar
- 4 tsp citric acid
- 2 tsp tartaric acid
- 4 tsp Epsom salts
- 1 tsp salt
- Fresh ginger

Bring the water to the boil on the stove

Remove from the heat and add citric acid, tartaric acid, Epsom salts, salt and sugar

Mix until the sugar is dissolved

Add lemon juice and grated fresh ginger to taste

Now pour juice into the prepared bottles and screw the lids tightly

A bottle goes a long way as you only need very little cordial in a glass of cold water - enjoy!

By Shirley Walters

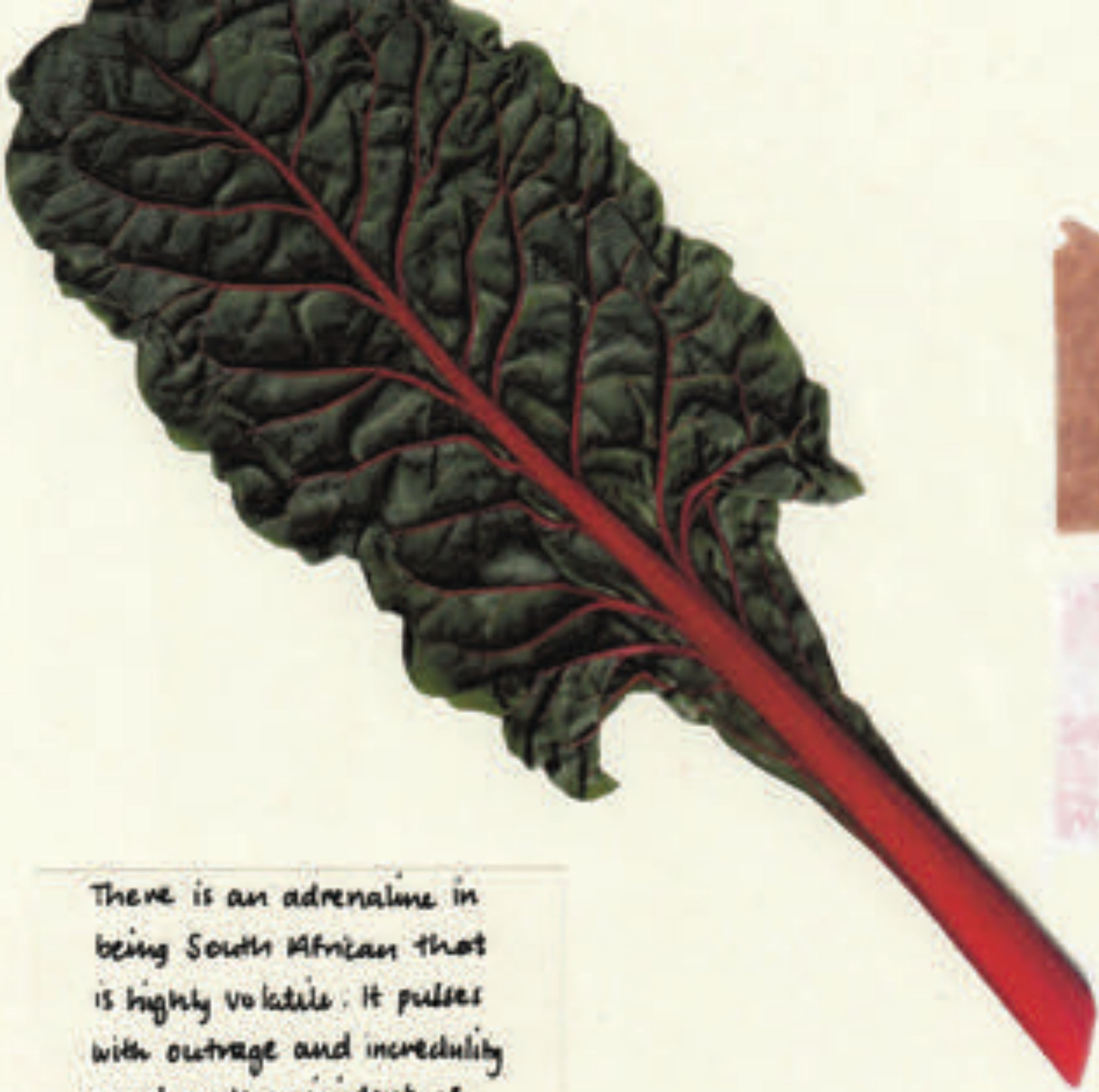

There is an adrenaline in being South African that is highly volatile. It pulses with outrage and incredulity as yet another incident of cruelty, bigotry and stupidity unfolds.

It is miraculous to have this erratic energy, these sparks in the abyss of rage, harnessed into practical action, and this has been my good fortune

- Felicia Kentridge

Girls on bikes

Harare CAN

"You hardly see black women or young girls riding in our neighbourhood - and I wanted to change that"

What did it mean for Harare CAN to get bicycles?

Freedom! We could go anywhere, we didn't need money to take a taxi, and we could just go to Monwabisi Beach near to us. Now that we have bikes, we can go anywhere we want.

Also, during hard lockdown taxis were scarce, so instead of waiting we could just hop on our bikes and go!

We set up the Harare CAN on the 30th March 2020. It started with a WhatsApp group with my neighbours for sharing information and good news. On the 5th of April we started our community kitchen and we've been cooking ever since.

Soon after setting up the CAN we were given bikes from Khaltsha cycles. They were giving bikes to CANs as part of their #HerosOnBikes campaign. I already knew how to ride, because I grew up in the Eastern Cape like a boy - I could ride a horse, I could ride a donkey — and that's where I learnt to cycle. But most of the girls in Harare CAN had never ridden before, so I actually had to teach them how to ride - it was a lot of work, and it took quite a while but, in the end, we managed to get everyone riding.

What was one of your favourite bike memories about the Harare CAN?

We received a voucher from Shoprite, but we had no way of getting to the shops. We decided to borrow crates from our neighbours, strap them to the back of our bikes and go.

When people saw us, they were so amazed! Even the security guard came to take a look, especially because we were girls. We piled sacks of potatoes and oranges into the crates, and we cycled back to Harare from Khayelitsha Mall.

Cycling is such a male-dominated space. Being able to cycle makes me feel equal. Just because I'm a woman doesn't mean I can't cycle long distances, just because I'm a woman doesn't mean I can't lift the bike above my head.

When people see us cycling on street, they get very hyped up. They're like, "yho! It's only girls!" And it's amazing, it's so great! I think it differs - the experience of being a black woman on a bike is different from being a white woman on a bike. When they see a white girl riding, it's not a big deal, because it's a popular sport for white people. But being a black girl on a bike is totally new.

In the end, we don't only use the bikes to transport food to the elderly people who can't come to the soup kitchen, we also use it for our own wellbeing - for exercising and all of those vibes.

No mask no ride
No helmet no ride

Ekuphumleni CAN

Harare CAN

Some reflections on CAN ride from Khayelitsha to Tafelsig

Just seeing everyone come together through cycling was amazing. It wasn't about anything else except riding and enjoying ourselves.

The city has been designed to keep us apart. The apartheid geography is still very much present today. Even though Khayelitsha and Tafelsig are so close together, many people from the Khayelitsha CANs had never been to Tafelsig before, and many people from the Tafelsig CAN had never been to Khayelitsha before. When you cycle there, you realise how close it actually is. Somehow, the bicycle ride broke the fear we had of each other's neighbourhoods.

We ended the ride with Nigeria's best jollof rice from Nana and Obi – and given how much xenophobia there is in South Africa, it was a powerful and symbolic way to end the ride.

With these bike rides, we show that something else is possible. As we cycle across spatial boundaries, we also cycle across race and gender boundaries. We need to show what's possible before we can build something better.

By Khanya Qonqgo and Leanne Brady

Tafelsig CAN

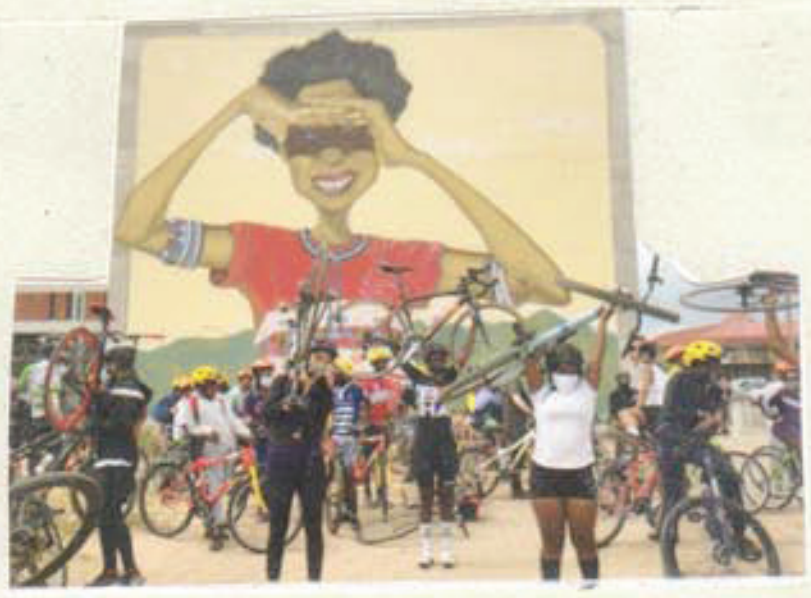

Toffee Apples

Hanover Park CAN

Covid-19 is a difficult time for many. In Hanover Park, the high rates of unemployment and safety issues combined with lockdown restrictions meant that many people felt very alone.

In the struggle to survive there was hardly ever time for anything light or fun. Kids were at home with no space to move around. The effects of the deadly virus, but also of the financial strains, job losses, stress, frustration and lack of movement and interaction, were starting to creep in.

As a CAN we decided that one small way of bringing some joy and happiness to people was to offer toffee apples to the children on Saturday mornings. We usually made between 450 and 500 toffee apples at time, handing them out in the sections of the neighbourhood that struggle the most, including 3 informal settlements.

In times where kids must grow up so fast, adapting to what is happening around them, we need to do what we can to allow them to be kids for just a tiny bit longer.

- 2.5kg sugar
- 2 cups water
- 1½ tsp red dye
- 2 tbsp vinegar
- 450-500 Apples
- 450 - 500 Kebab sticks

Heat all ingredients in a pot. Use a low heat and stir all the time to prevent the sugar from caramelising

Keep stirring until all the sugar is dissolved and the mixture is shiny and smooth

Push kebab stick into apple and dip in syrup mixture

Leave to cool on a baking tray until hard

Enjoy!

By Yaseen Johaar

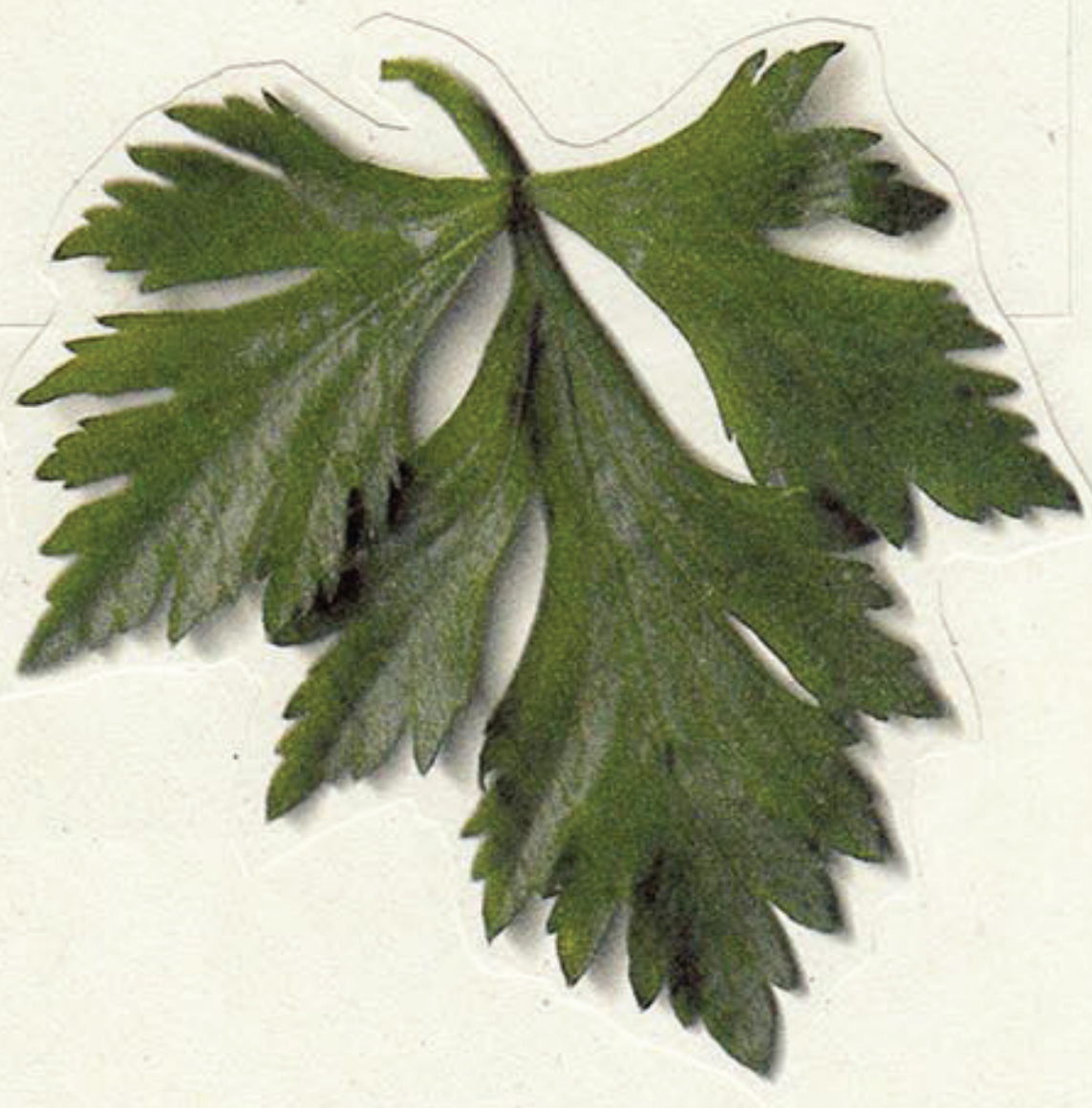

Is Cape Town Together a political threat?

Writers CAN

Originally published in Daily Maverick as "Cape Town Together, a neighbourhood-based network of 170 community action groups" – 26 August '20

Instead of seeing threats to their political agendas, Cape Town's mayor and ward councillors should rather explore the possibilities for collaboration and co-operation with local Community Action Networks to build the city 'back better' in the wake of the pandemic.

What is Cape Town Together?

Cape Town Together (CTT) is a network of self-organising neighbourhood-based teams engaged in rapid, community-led responses to Covid-19. It is not a formal organisation or non-governmental organisation (NGO) and is not aligned to any political party. The network is made up of thousands of people across the city working together to meet the challenges we see in our communities. These neighbourhood hubs are called Community Action Networks (CANs).

Some of us are organisers, some are carers, some have worked in outbreak situations before and some are new to organising in this way. What unites us is that we are all responding to the long-standing health and socioeconomic crises that Covid-19 has exposed and exacerbated in our city.

Although they began as a rapid response to Covid-19, many CANs are starting to grapple with how to build back better, recognising that going back to a pre-Covid "normal" is not a goal we should aspire to, because there was nothing "normal" about the world before Covid-19. In fact, going "back to normal" is one of the worst things that could happen. Instead, we want to move forward by building a better society that is more socially and environmentally just.

The work that we do

Over the last five months, 170 CANs have been set up. There is no "one size fits all", as every neighbourhood is different. Some CANs have set up mask-making and distribution schemes. Others have opened community kitchens that provide hot meals to thousands of people every day, while also being sites for communicating and demonstrating Covid-safe practices, including handwashing, mask-wearing, and physical distancing.

Some CANs have found creative ways to address homelessness in their neighbourhoods by collaborating with NGOs and church groups to secure temporary accommodation and connecting homeless people to employment opportunities. Other CANs have established community care centres for Covid-positive people who are low risk and do not need medical care, but do need a safe, nearby space to self-isolate away from their more vulnerable family members.

Simultaneously, CANs have organised community clean-ups, started food gardens, collected clothing and household items for those affected by evictions and flooding, assisted school children with home-based learning, and put together baby bundles for new mothers sheltering at home at the height of the pandemic.

All of this is important, valuable work. Being a part of a CAN is an opportunity to be part of building and creating something together. The CANs have provided hope, inspiration and solidarity at a time when South Africa's future looked its darkest. Together we have demonstrated the power of collective action – action that will be invaluable in dealing with future pandemics and natural or economic disasters.

All of this has been achieved without any formal support from big donors or the state. Our flexible and flat structure meant that we were able to mobilise with incredible speed despite lockdown restrictions. When formal social welfare schemes were slow to be rolled out, insufficient and difficult to access, the CANs and other civil society bodies were able to mobilise immediately to step into the vacuum.

When people had no money to buy food, and hunger was a more immediate threat than Covid, the CANs got to work. CAN volunteers from across the city found creative ways to resource their efforts from within their CANs and through solidarity between CANs.

This flexibility is our strength. Anybody can join their local CAN, and every CAN decides its own course of action and way of working, and makes decisions about which NGOs, government officials, religious organ-isations, or civil society groups to collaborate with. In fact, many CAN members also work for local NGOs, are members of religious organ-isations, or work in government. This allows the CANs to act as community-based hubs, with the flexibility to do whatever is not being done by more formal actors.

Working alongside the state response

While each CAN is different, each focusing on specific needs in their neighbour-hoods, many CANs have worked alongside, and in support of the state's Covid-19 response programmes. Some CANs have built important, mutually valuable relationships with the state (for example, presenting to hotspot teams, or working with community-based teams, and local ward councillors).

In the early days of the pandemic, when information and education about the virus and important preventative behaviour changes were of the highest priority, the CANs acted as distribution networks for official guidance. CAN members distributed pamphlets to their neighbours at spaza shops and taxi ranks. Community kitchens put up Department of Health posters and adapted these into accessible infographics, quickly becoming hubs for information sharing and demonstration of Covid-safe practices that make sense in resource-scarce settings.

There have been multiple touchpoints between CANs and public servants. There were multiple public co-learning or consultative sessions with CANs and government officials in the early days of the response, including the Premier of the Western Cape, Alan Winde, and the head of the Department of Health, Dr Keith Cloete.

However, despite these promising connections, most CANs have received little to no support from the state. CANs have been unable to access local public infra-structure during this public health emergency. Some CANs have tried in vain to get permission to use local municipal halls for their activities. Others have requested permission to use community halls for community care centres and been denied. These are all public facilities that should be made available for the public good, especially during a crisis.

The current spat between a Bonteheuwel ward councillor and the Bonteheuwel Joint Peace Forum CAN arose because the CAN was granted permission by the facility manager to use a rundown public building for a community care centre, in exchange for a monthly rental. Despite the CAN having already begun major renovations using its own resources and fundraising to make the facility usable, the project is sadly currently on hold.

Work that is deeply political

For all our efforts to work alongside and in support of government programmes, and to build meaningful relationships with public servants, trying to avoid party politics has been a constant challenge.

Cape Town Together is a non-partisan, non-religious and vigorously independent network not beholden to any political party, donor, or organisation. While we continually resist the distraction of party politics, the work we do is, of course, deeply political. As one CAN member put it, in the context of extreme hunger and poverty, "every loaf of bread is political".

In recent weeks, political actors have accused the CAN network of being an NGO that is misleading well-meaning citizens, of encouraging community members to break the law, and of having an opaque, subversive political agenda.

In part, these accusations are a response to some CANs working with civil society organisations to legally contest the unlawful eviction of residents in Empolweni – and ignores that fact that the Western Cape High Court found against the City and in favour of the residents.

In addition, the de-centralised, self-organising nature of Cape Town Together means that there is no way to "steer" the network towards any particular agenda. The network is steered by the needs that emerge, and the CANs are accountable only to their own communities.

The recent misrepresentation not only reflects a misunderstanding of the nature of the network, but also positions the thousands of CAN members leading social and welfare projects in their own neighbourhoods as passive and powerless victims of an opaque and coordinated political agenda.

There is great scope for a multiplication of energies, efforts, and resources, and we invite ward councillors, City officials or any other interested person to meet with their local CAN to explore possibilities for generative collaboration.

The mayor and ward councillors who have regrettably misinterpreted the values, orientation and ways of working of Cape Town Together need to ask themselves why the prospect of hyper-local organising and decision-making in response to Covid-19 and other socioeconomic challenges constitutes such a threat to their political agenda.

Charlene Windvogel, Eleanor Whyle, Elise Malherbe, Erica Inches, Fadia Gamieldien, Glenda Rania, Ian Miller, Isa Jacobson, Leanne Brady, Lynn Abrahams, Manya van Ryneveld, Mboneleli Gqirana, Miki Redelinghuis, Mzikhona Mgedle, Nadia Mayman, Nana Odumuko, Nicola van Schaik, Pamela Nqayi, Pamela Lidelwa Silwana, Paul Crawford, Robyn Liebenberg, Suzanne Zaan Vermeulen, Vanessa Nelson, Marlise Richter, Zelda Lategan

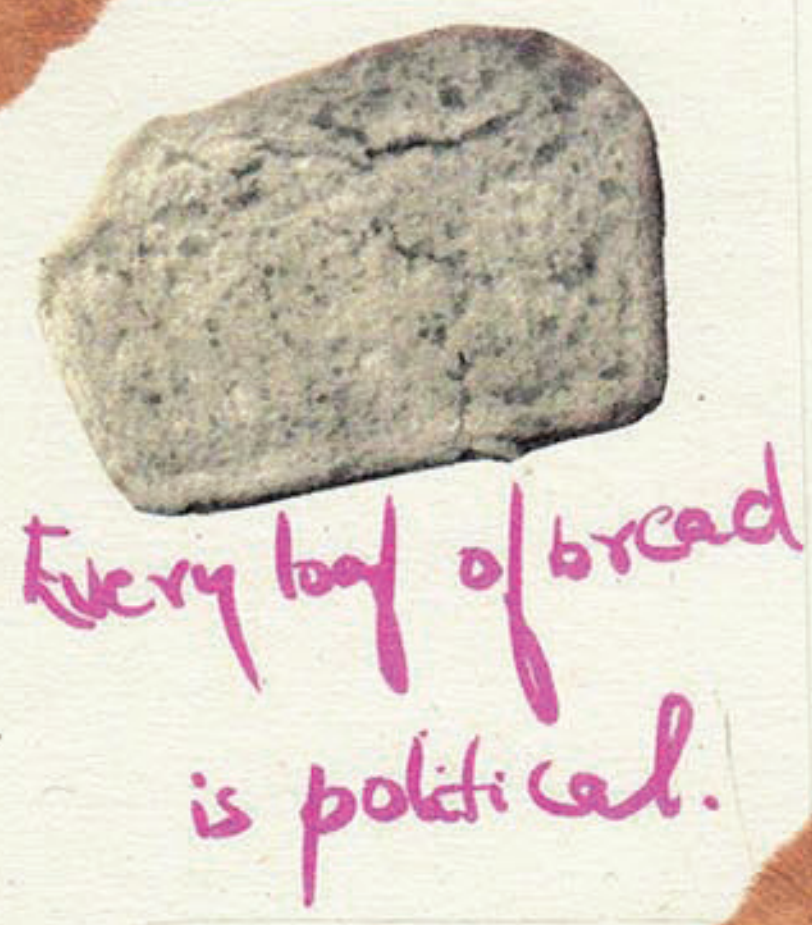

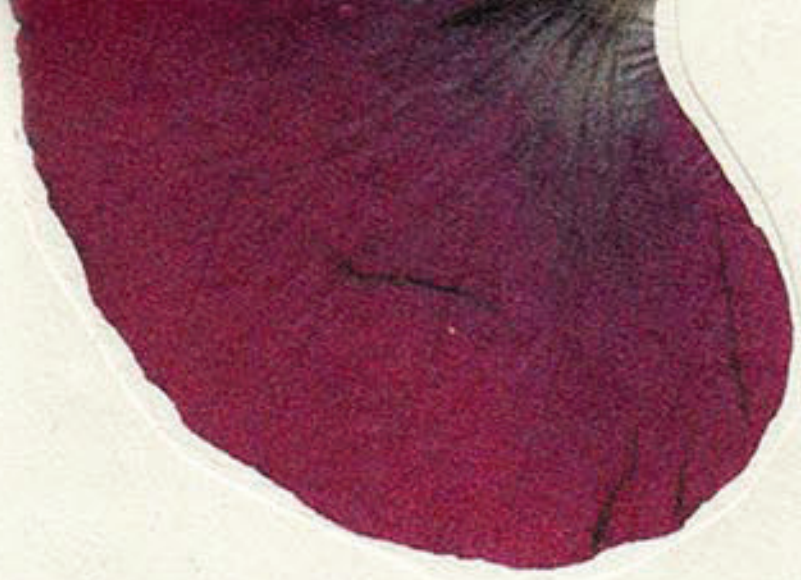

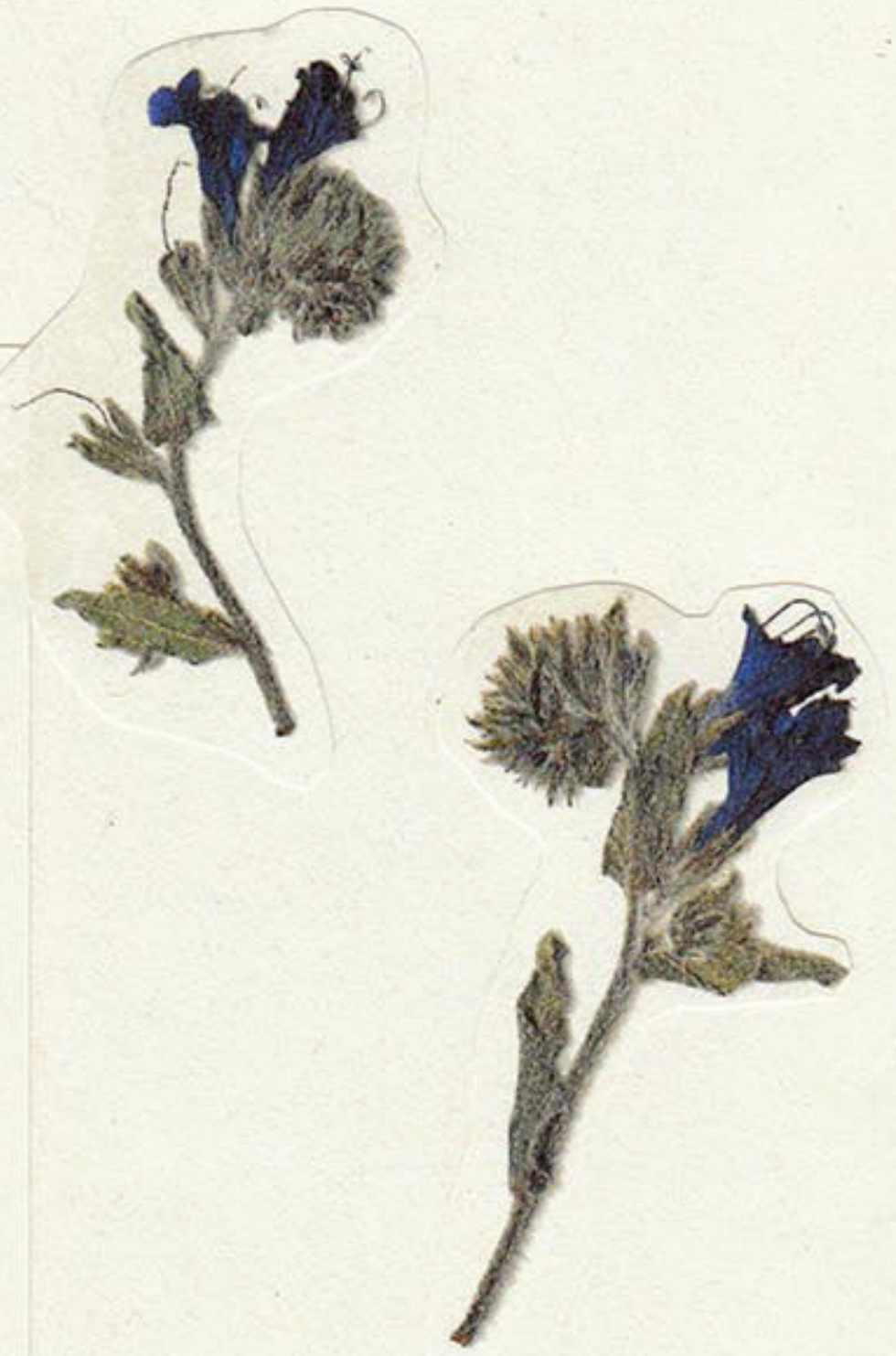

Whyle's choc chip biscuits

Connecting CANs

In the most fraught moments, when emotions run high and sleep is scarce, these biscuits work.

- 140g butter
- 140g treacle sugar
- 110g castor sugar
- 1 egg
- 250g flour
- 3g baking powder
- 4g bicarbonate of soda
- Big pinch of salt
- 180g choc chips

Cream the butter and sugar until a little bit light

Mix in an egg

Sieve dry ingredients and add to the butter mixture

Mix in the chocolate chips

Roll into balls and place on a baking tray

Bake at 160'C for 12-15 minutes - until they are starting to brown on top

Take out the oven and leave to cool before taking them off the oven tray

By Eleanor Whyle

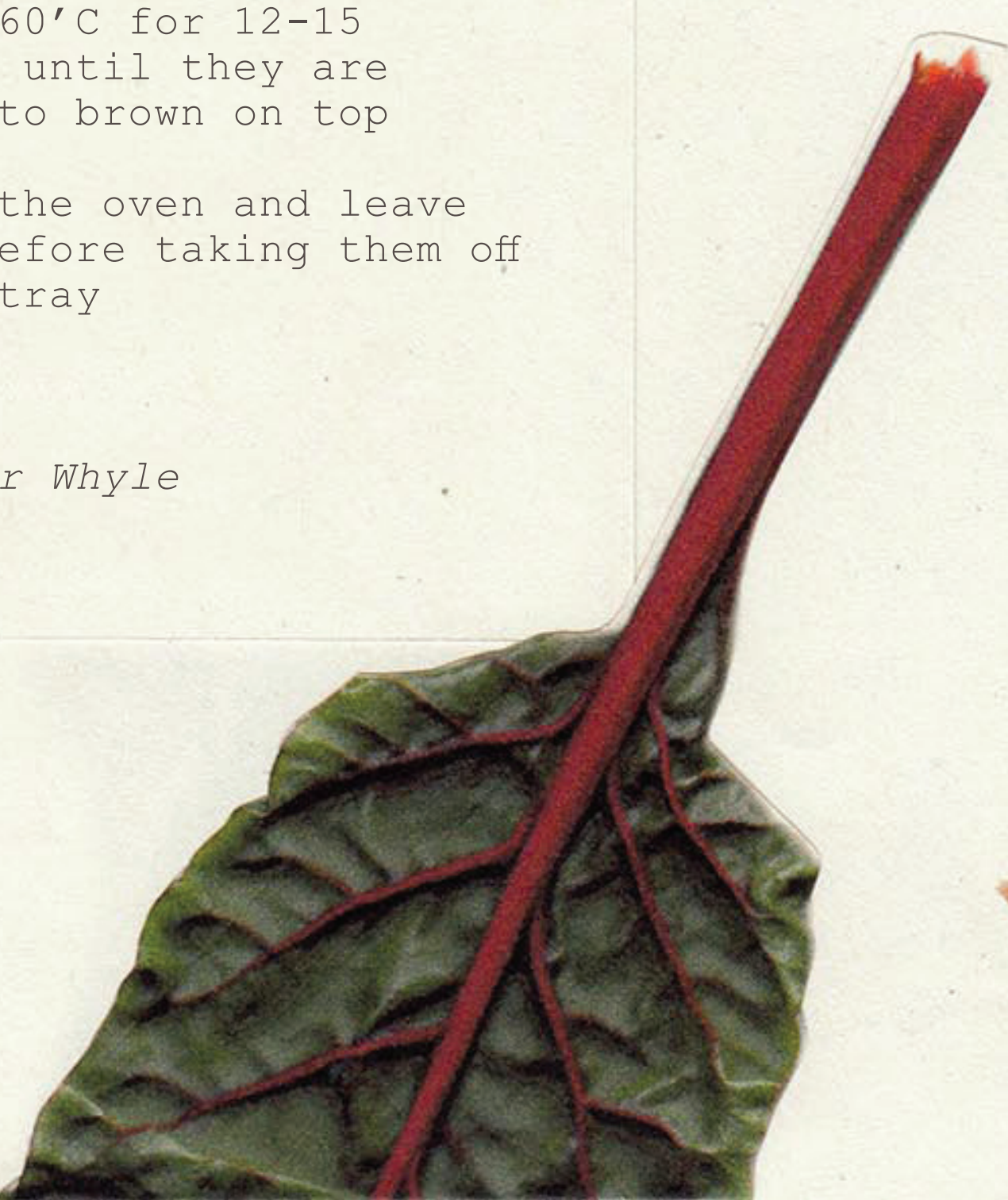

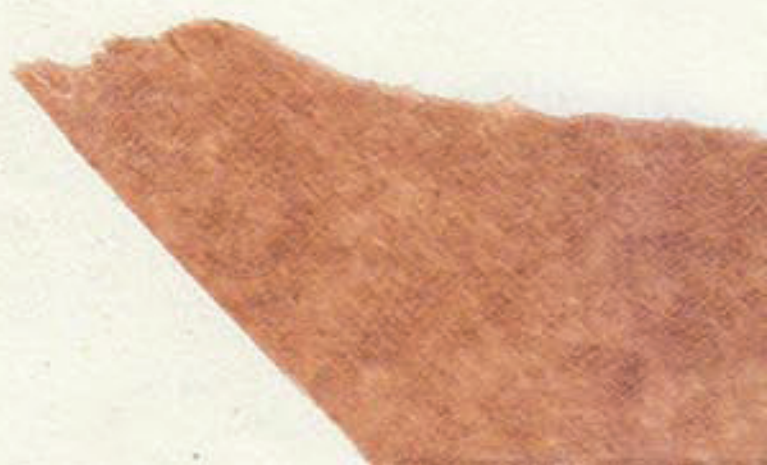

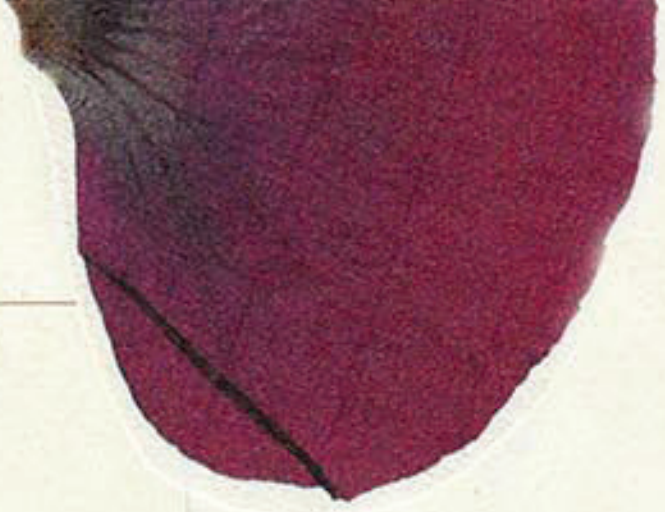

Practising democracy in a pandemic

Newlands CAN

The work of the CANs represents a model of engaged citizenship from which there is much to learn about participation, democracy and solidarity.

The free flow of information – learning together, sharing knowledge and communicating between and beyond our CANs – quickly became a defining feature of the CANs. In Eastern Cape Together, CAN champions in rural districts and small villages quickly began sharing public health messaging, translated into isiXhosa. Government-issued communication in technical English was not only exclusionary but also unhelpful. Democracy is about participation by all the people – if we can't understand, it is very difficult to participate. Through the democratisation of knowledge and information, we ensure that everyone can be an active participant in stopping the spread of Covid-19.

In order to be as safe as possible, the CANs did much of their organising online, using platforms like the Cape Town Together Facebook page and WhatsApp groups to share information and inspiration. This was not information produced by 'high-level experts' with little relevance to the lived ex-periences of many, but rather knowledge co-created by the CANs themselves. Co-learning has been a foundational building block for the network – weekly or monthly meetings have been held where we learn from and get to know one another. CANs used co-learning sessions and inter-personal networks to co-create relevant, timeous information on COVID, gender-based violence, running community kitchens or growing food. In order to make these processes as inclusive and participatory as possible, in a context in which conn-ectivity is unaffordable for many, Cape Town Together launched a data fund through which any CAN member could receive data bundles, and be able to participate actively in online organising.

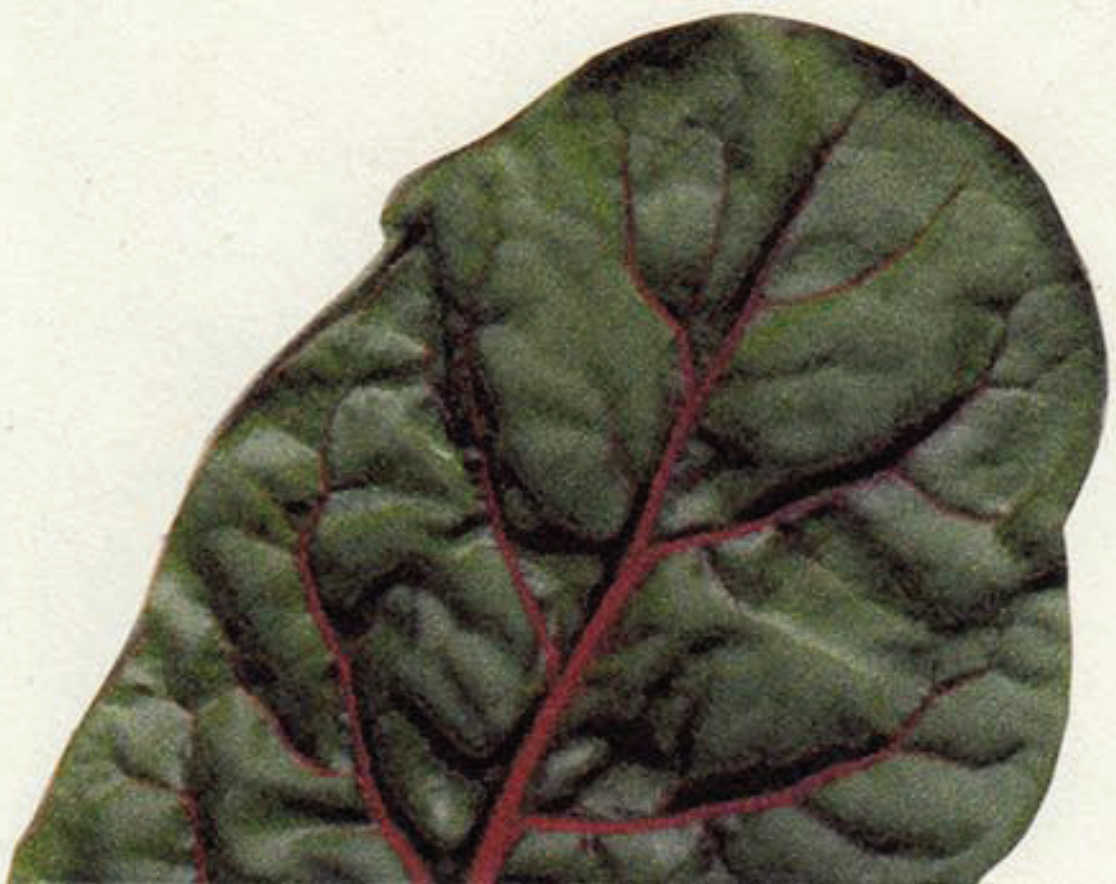

Enabling and encouraging active participation for all was also central to the CANs approach to resource sharing, especially in community kitchens and food distribution schemes. Partnerships between well-resourced CANs and under-resourced CANs were built on mutual trust formed over time. This enabled a power dynamic in which beneficiary CANs are free to make their own decisions about how resources should be used. This dynamic – in which the distinction between bene-ficiary and benefactor is erased, and both are considered active parti-cipants in solidarity – is also extended to those who ate at community kitchens or received food parcels. People who are hungry are not passive recipients of charity, in many cases they made significant contri-butions to growing, making, and distributing food. This reflects a belief that everyone has something to contribute to their CAN, that all forms of labour are valued, and leadership comes in a myriad of forms.

While we reject hierarchies, we embrace new forms of leadership. Within the network our leadership is a feminist leadership, that centres listening, caring, supporting, stepping forward to help, standing together when needed, and stepping back when appropriate. Practising democracy is about building relationships, and fun and laughter are a central part of this process. We have found many playful, caring, open, welcoming ways to encourage broad participation and sustain our energy through difficult times. We strive to recognise and celebrate these forms of leadership as they emerge.

In part, this leadership strategy involves 'moving at the speed of trust'. This means recognising that CAN members are diverse across age, sex, gender, ethnicity, language, religion, race, class, physical ability, and geography, and that differences in perspective are an inevitable part of our work, especially in such an unequal society. Organising virtually together has not always been easy - there have been potholes to navigate. Building trust is critical to acting together to achieve our common goals. We strive to listen openly and carefully, to 'call in' rather than 'call out' and to not take decisions until everyone has been heard and acknowledged.

While the network spans across the city of Cape Town, our work starts at home, in our own neighbourhoods. This model of organising is reminiscent of anti-apartheid organising based on the idea of street committees, going back to the early 1960s – organising street by street towards deep transformation. At its core, democracy is about people participating in making decisions about matters which concern them. Through our participation, we learn what it means to lead, to make decisions collectively, to develop deeper understanding of the collective challenges we face, and new ways of working, organising and listening. We learn to dream of another more just, more equal world. Participatory democracy is about both the means and the ends – how we get to our destination is as important as the arrival!

By Shirley Walters

Common Change: Managing money in mutually accountable ways

Philippi-Newlands CAN partnership

Managing money in community projects is the biggest challenge as it can easily become `a bone over which to fight'. Donors are also reluctant to give if they do not trust how the money will be spent.

The Philippi-Newlands CAN partnership used Common Change to help manage the funds they raised for their food and sanitiser distribution project. Common Change is a platform to help community organisers manage funds democratically. The platform helps you establish a decision-making team and make decisions collectively. "It's useful for decision making – it has the potential for gathering points of view." Common Change does the bookkeeping and sends weekly finance reports, in exchange for 5% of all donations.

We set up a team of 3 Philippi CAN-members and 3 Newlands CAN-members. Anyone can submit a project proposal for consideration. These proposals are then discussed and voted on by the team. Once a decision is made, the funds are distributed accordingly through the Common Change platform. "The collective decision--making process meant that donors could not determine for the recipients what could be done with the money."

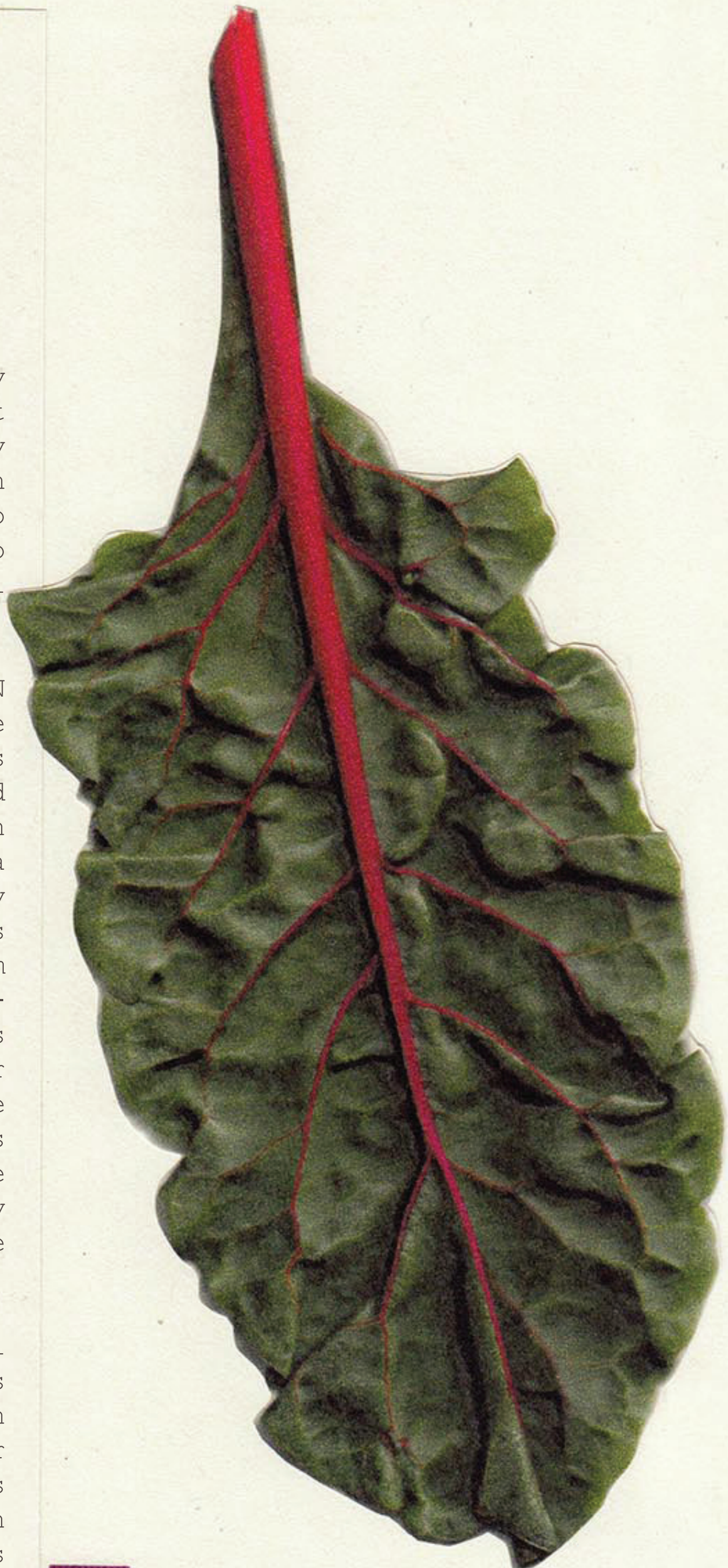

By Mercy Dube, Astrid von Kotze and Shirley Walters

Heavenly Banana and Oat Muffins

Mini Meltdown

- 1½ cups oats
- 1¼ cups milk
- ½ cup of chopped dates
- ½ cup dark brown sugar
- 1 cup mashed ripe bananas
- 2 large eggs
- 2tbsp honey
- 1tbsp oil
- ½ cup flour
- 1tsp baking powder
- ½tsp bicarbonate of soda
- ½tsp salt
- 1tsp cinnamon
- ¾ cup chopped Pecan or peanuts (optional)
- ½ cup raisins (optional)

Preheat the oven to 180°C

Line a standard muffin tin with 12 liners or use silicone muffin cups/tray

Pour the Oats into a large bowl, add the Bicarb, chopped dates, milk and mix

Let this mixture soak for 30 minutes

Add the brown sugar, mashed bananas, eggs, honey and oil to the oats and mix well

Then add the flour, baking powder, cinnamon and salt

Add the optional raisin and nuts

Mix gently

Pour the batter into the prepared muffin tins/moulds

Bake until a toothpick inserted comes out clean (about 30 minutes)

Allow to cool before serving

By Peter and Lesley Wagner

Torched vehicle becomes public art work

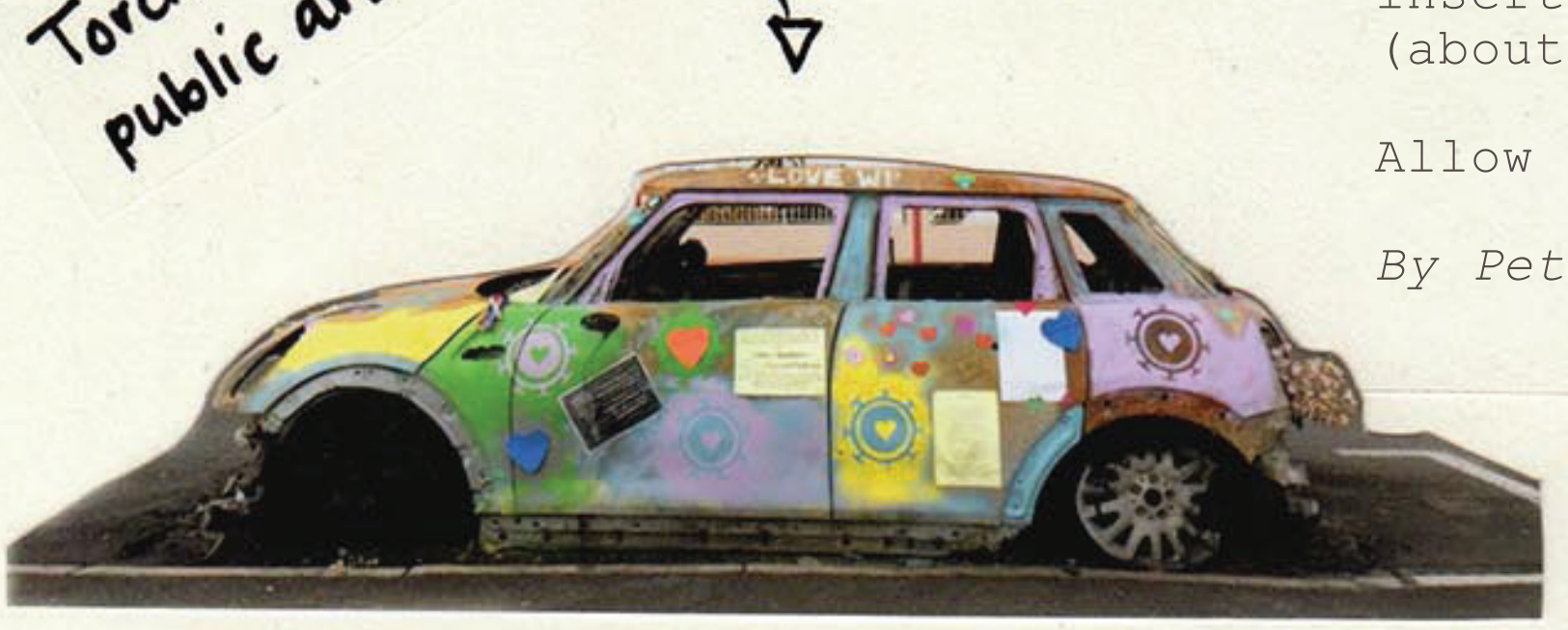

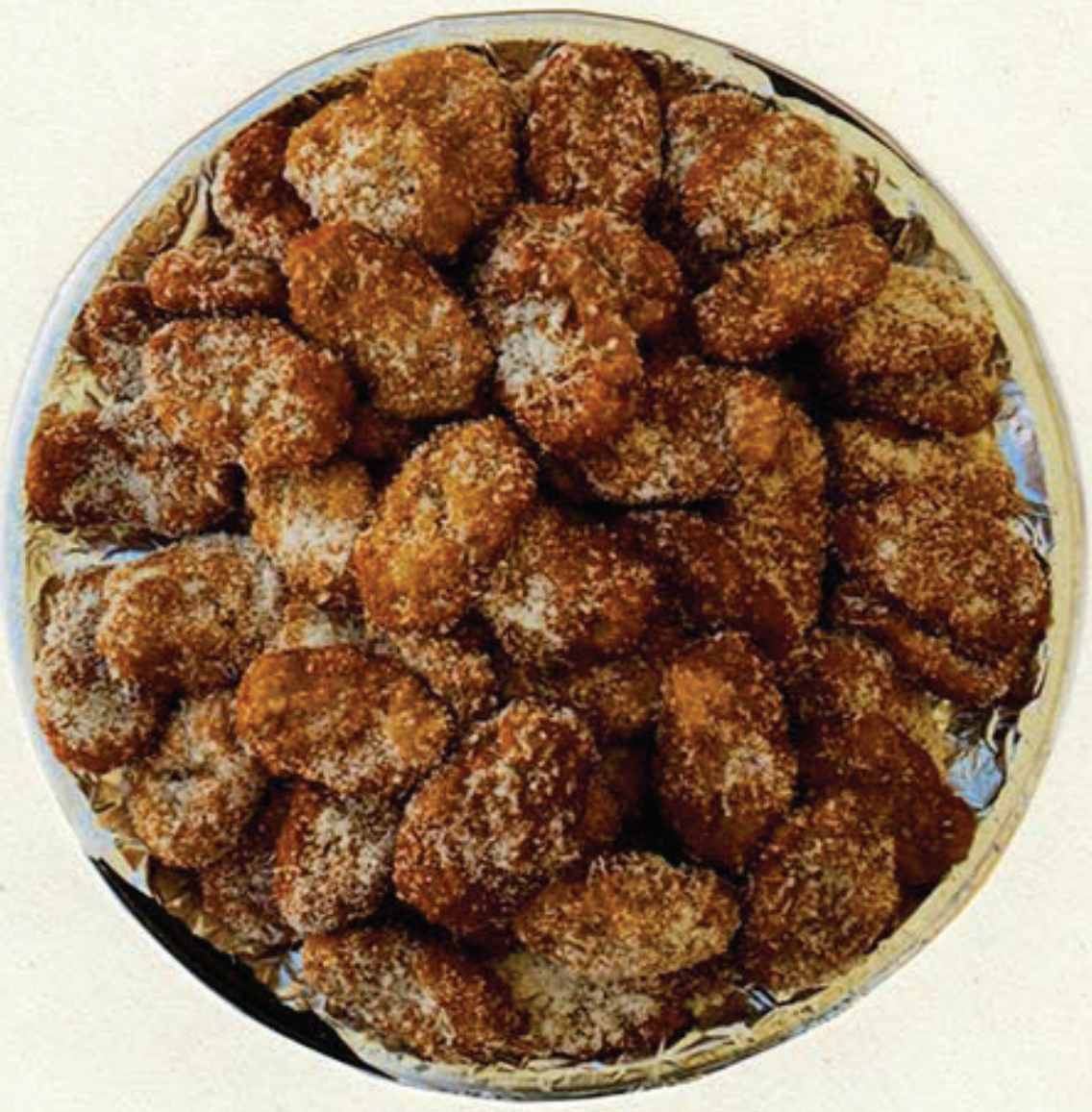

Potato Koesister

Ocean View CAN

Ricochet (Ocean View CAN) always brings a big batch of his mom's koesisters to our face-to-face meetings. These koesisters have sustained many CAN-members through long and difficult conversations, and always bring us back to our smiling, dancing selves.

- 1 large potato, boiled and mashed
- 5 cups all-purpose flour
- 1tsp baking powder
- 1 packet dry yeast
- 1 pinch salt
- 1 tbsp cinnamon
- 1 tbsp ginger
- 2 tbsp sugar
- 1 large egg
- 1½ cups warm water
- 4 tbsp oil
- 2 tbsp
- Oil for deep frying
- Desiccated coconut for sprinkling

Mash potato and add butter

Mix in warm water and oil, then add egg

Add dry ingredients and knead until smooth

Cover with a clean dishcloth and leave to rise in a warm place. The dough should double in size

Roll the dough into balls, about the size of a golf ball. Cover and leave to rise again while you heat up your oil

Deep fry the koesisters until golden brown

While still hot, drizzle with syrup. Roll the koesisters in coconut

By Ricochet Van Rensburg

Postscript: Making sense of magical chaos

In just nine months this wonderfully messy and ungovernable network of people from across the city has had significant impact on the lives of thousands of people living in Cape Town during a time of intense crisis. In doing so, it has demonstrated what is possible when spontaneous, informal eruptions of collective action are enabled and valued - even if only for a moment.

Documenting, reflecting on and learning from this moment has been an important aspect of our work. Dala Kitchen - More than A Cookbook is an output of the Connecting CANs Fellowship, established with the support of the National Research Foundation, South Africa. One of the motivations for establishing the Fellowship and producing creative outputs like this book was to facilitate a space of collective reflection and sensemaking among Connecting CAN members and the wider network.

The learning is not always capturable. Some of it lies in this book, but much of it is intangible - felt rather than known, instinctual rather than conscious. This type of knowledge and learning reflects the hyper-local nature of the CANs, in which many different forms of knowledge, including the expertise of those living in a space who have a deeper understanding of local realities in historical context , is drawn on and legitimised. With this approach comes the possibility of working at scale, without scaling up. We can do justice to localised knowledge and vast contextual differences by working creatively and with autonomy, celebrating the diversity of perspectives rather than trying to force them into one replicable model or standardised policy.

We can also work effectively to meet needs and find pragmatic solutions to immediate problems, without resorting to the usual modes of bureaucratised account-ability or forms of charity insensitive to power. When we prioritise trust, solidarity, interpersonal relationships and horizontal, post-heroic leadership, we are investing in a politics of care where everyone's contributions are valued and recognised. We can break down divisions between benefactors, volunteers and recipients and foreground the common goods that unite and support us all. Whether it be cooking, administrative work, caregiving, communication, emotional labour or fund-raising, the labour involved is recognised and the contribution is valued.

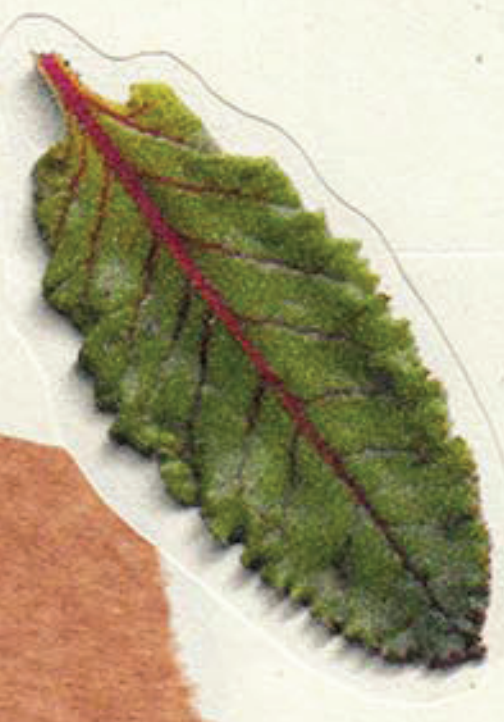

This investment in care stands in stark contrast to the dominant aspects of the state's response to this crisis, which was largely top-down, hospi-centric and out of step with the lived realities of ordinary people. About 15% of people with Covid-19 will need hospital-level care. Nonetheless a coherent and compassionate response must pay attention to the remaining 85%. Whether they have mild symptoms or no symptoms at all, these individuals will have to find a way to protect their families from infection while living in cramped shared spaces with inadequate access to water and sanitation, deal with the economic consequences of being unable to work, face anxiety about being ill and stigma from others, and require care and monitoring to ensure they are able to receive medical care if they need it. As a society, we have a duty of care to the 85%. Yet with the ensuing effects of lockdown - job losses, closure of school feeding schemes and rise in hunger to name a few - Covid-19 exposed the gaping holes that have long existed in the South African state's ability to care for its people.

The power of people caring for each other through a self-organised response stands in stark contradiction with the power of top-down behaviour change interventions or placing blame on individuals, leaving little space for listening, or more equitable redistribution of power. Informal networks of care are deemed a political threat to the status quo and go unrecognised and un-resourced, while top-down, hospi-centric, militarised responses from government are upheld and heavily resourced. The bureaucratic red tape that is enacted to prevent wasteful expenditure becomes an excuse to ignore, or actively suppress the efforts of informal, community-led groups, while enabling health-care systems that funnel public funds into private pockets.

The CANs do not represent a completely new mode of organising - hyper-local, community-led organising is part of South Africa's collective memory. However, the experiences of Cape Town Together, and the awe-inspiring impact of the CANs constitute a powerful argument for a new way of thinking about how we respond to public health crises. Ordinary people caring for each other constitute the backbone of any social health system, during times of crisis and times of peace. Every hot meal served in a community kitchen is also an opportunity to demonstrate realistic Covid-19 safety protocols. A network of 20 000 people across the city directly involved in responding to a public health crisis is a platform for Covid-19 conscientisation that goes well beyond traditional forms of public health messaging.

Far beyond the limited and linear ways in which community engagement is often understood, the CANs have demonstrated not only the valuable contribution to social goals made by ordinary people who devote their time and energy to caring for their neighbours, but also what we could achieve as a society if we centre care instead of capital, put people before profit, and invested in economies of care, particularly in times of crisis. Rather than seeking to frame the efforts of those working to plug the holes in South Africa's social safety net as a threat, the CANs should be understood as a call for a world in which care work is recognised and valued.

The work of the CANs is work, but it is work based on collective care, inclusivity and the fundamental belief that we have a collective right to live in a world free from inequality and injustice, and a collective respons-ibility to take action against it. Far from being a political threat, this is what active citizenship in a democracy should look like. In times of crisis, the role of the state must include supporting, enabling and listening to bottom-up social mobilisation.

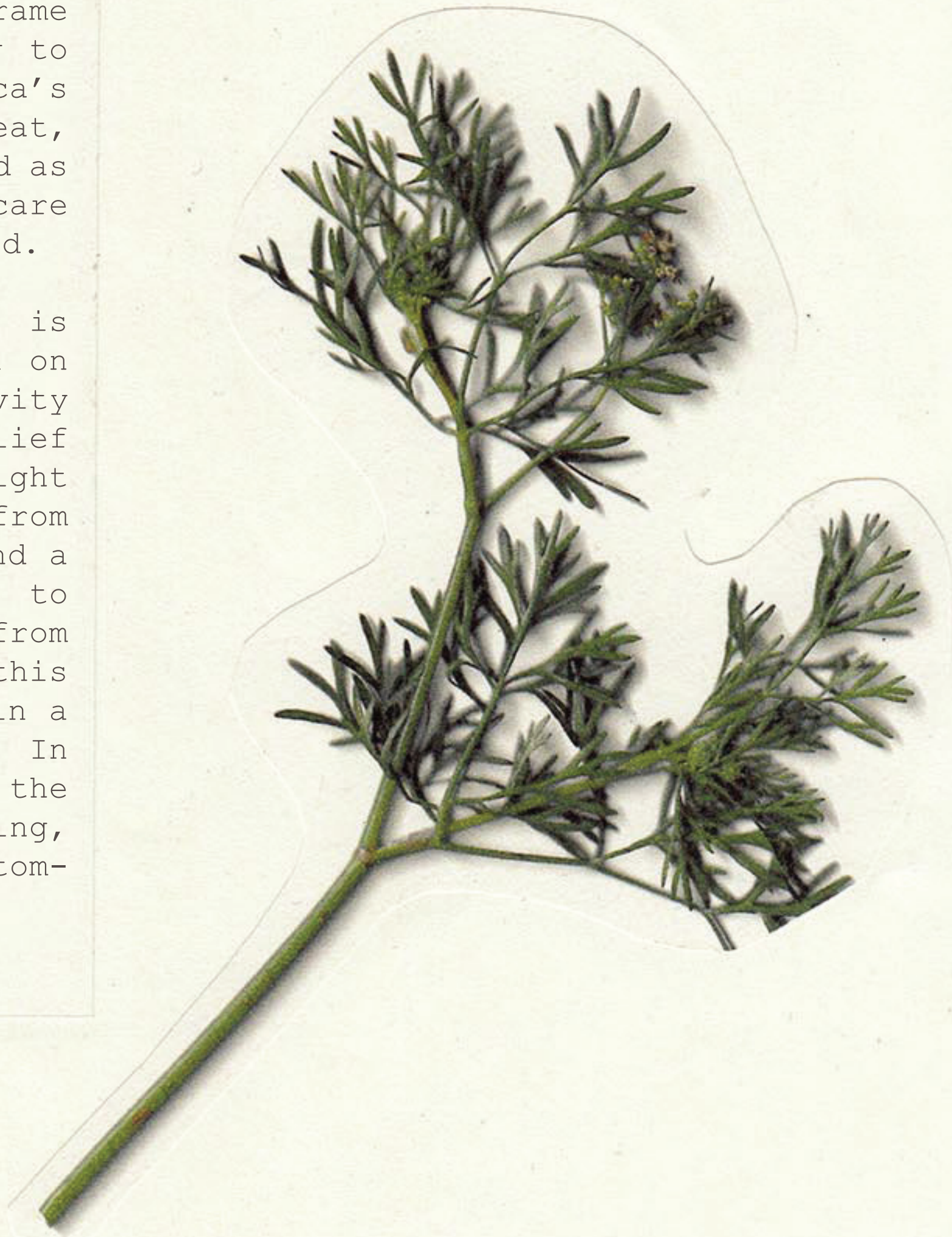

Dala Kitchen

Edited by Eleanor Whyle, Manya van Ryneveld and Leanne Brady

ISBN: 978-0-620-91499-4

Printed in December 2020 in Cape Town, South Africa

Attribution-Non Commercial-Share Alike 4.0 International (CC BY-NC-SA 4.0)

CC-BY-NC-SA This license requires that reusers give credit to the creator. It allows reusers to distribute, remix, adapt, and build upon the material in any medium or format, for noncommercial purposes only. If others modify or adapt the material, they must license the modified material under identical terms.

How to cite this book:

Cape Town Together CAN. Dala Kitchen - More than a cookbook. Edited by Eleanor Whyle, Manya van Ryneveld and Leanne Brady. Cape Town Together CAN, Cape Town, South Africa. ISBN: 978-0-620-91499-4

The Cape Town Together Connecting CAN would also like to acknowledge support from the South African Research Chairs Initiative of the Department of Science and Technology and National Research Foundation of South Africa, and from the University of the Western Cape, South African Medical Research Council Health Services to Systems Research Unit. Any opinion, finding and conclusion or recommendation expressed in this material is that of the Connecting CANs collective and not the funders.

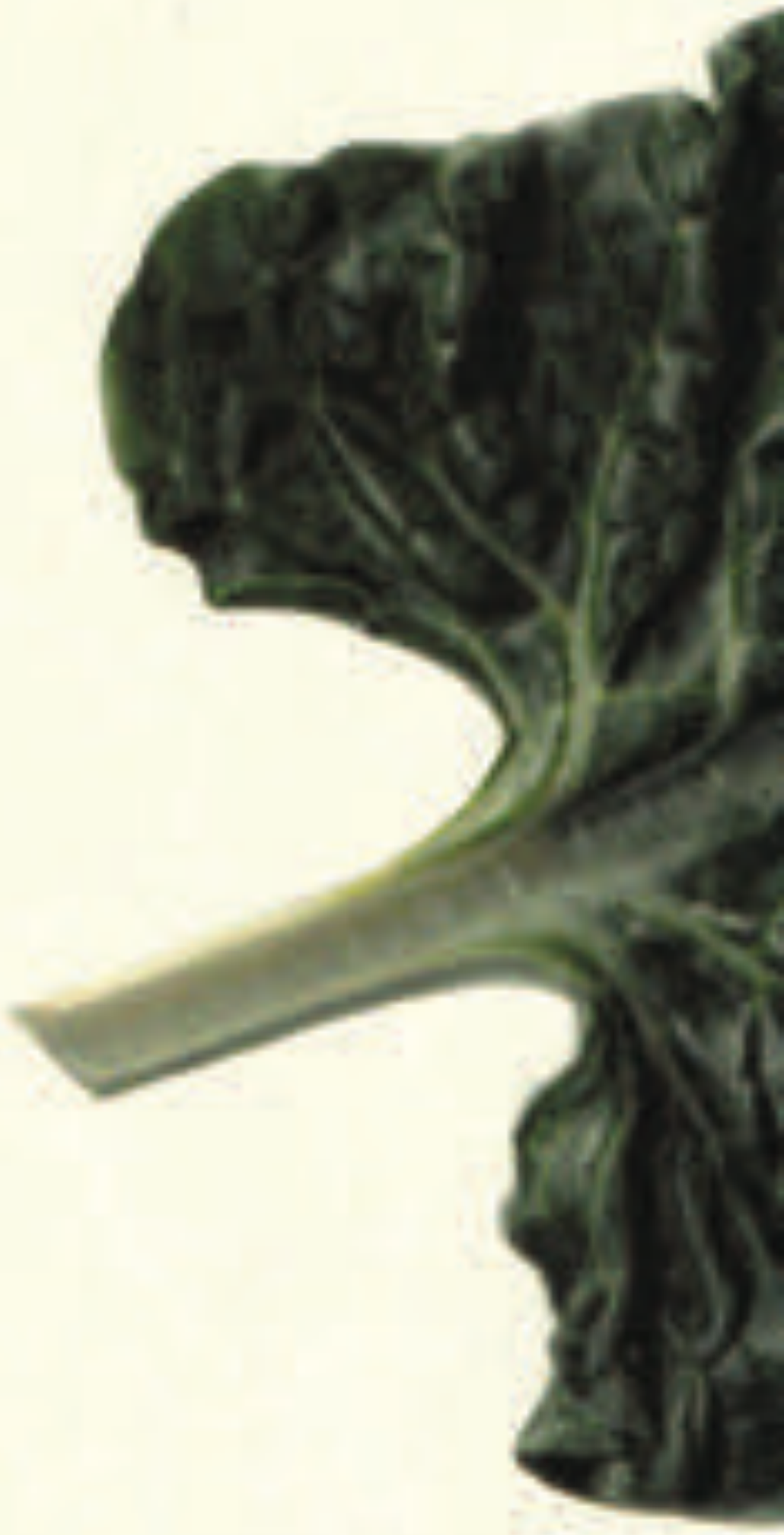

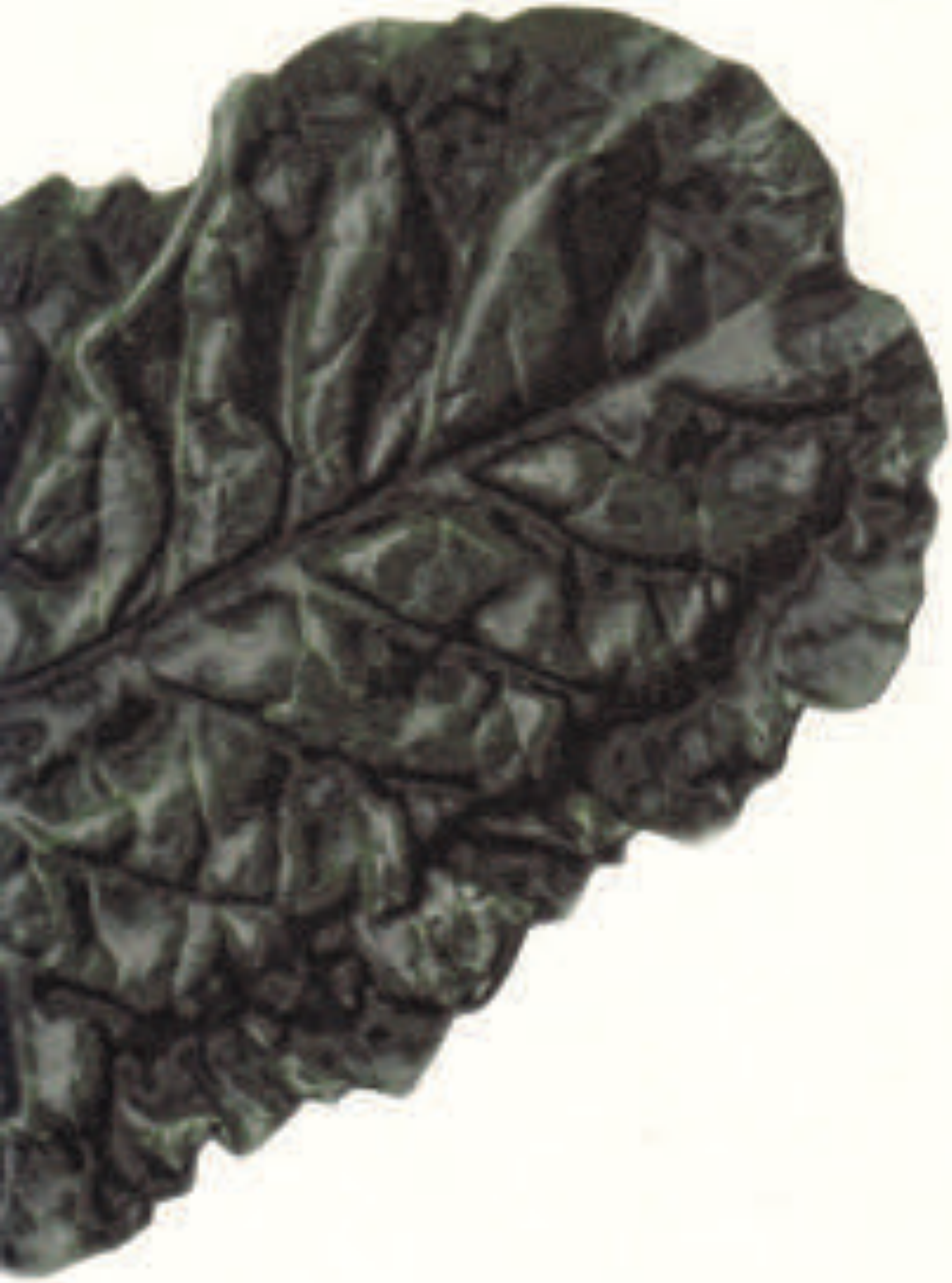

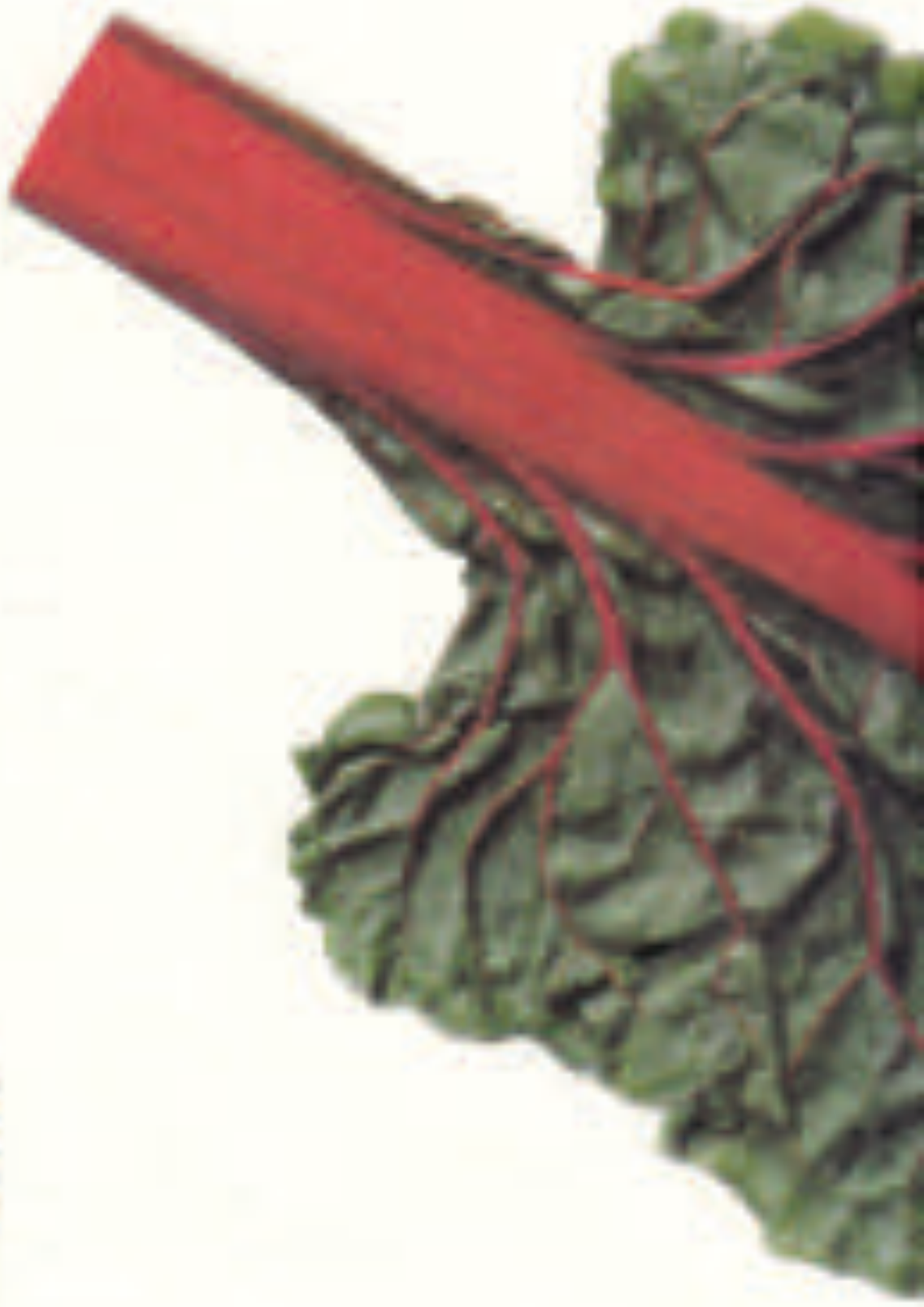

Cape Town Together

ISBN: 978-0-620-91499-4
